# De novo assembly of the Indian blue peacock (*Pavo cristatus*) genome using Oxford Nanopore technology and Illumina sequencing

**DOI:** 10.1093/gigascience/giz038

**Published:** 2019-05-11

**Authors:** Ruby Dhar, Ashikh Seethy, Karthikeyan Pethusamy, Sunil Singh, Vishwajeet Rohil, Kakali Purkayastha, Indrani Mukherjee, Sandeep Goswami, Rakesh Singh, Ankita Raj, Tryambak Srivastava, Sovon Acharya, Balaji Rajashekhar, Subhradip Karmakar

**Affiliations:** 1 Department of Biochemistry, Room 3020, AIIMS - All India Institute of Medical Sciences, Ansari Nagar, New Delhi 110029, India; 2 Vallabhbhai Patel Chest Institute (VPCI), Delhi University, New Delhi 110007, India; 3 Kanpur Zoo, Hastings Ave, Azad Nagar, Nawabganj, Kanpur, Uttar Pradesh 208002, India; 4 Institute of Computer Science, University of Tartu, J. Liivi, Tartu 50409, Estonia; 5 Celixa, 19/1 Sankey Road, Bangalore 560020, India

**Keywords:** peacock, Pavo cristatus, Indian national bird, genome assembly, Oxford Nanopore

## Abstract

**Background:**

The Indian peafowl (*Pavo cristanus*) is native to South Asia and is the national bird of India. Here we present a draft genome sequence of the male blue peacock using Illumina and Oxford Nanopore technology (ONT).

**Results:**

ONT sequencing gave ∼2.3-fold sequencing coverage, whereas Illumina generated 150–base pair paired-end sequence data at 284.6-fold coverage from 5 libraries. Subsequently, we generated a 0.915-gigabase pair de novo assembly of the peacock genome with a scaffold N50 of 0.23 megabase pairs (Mb). We predict that the peacock genome contains 23,153 protein-coding genes and 75.3 Mb (7.33%) of repetitive sequences.

**Conclusions:**

We report a high-quality assembly of the peacock genome using a hybrid approach of sequences generated by both Illumina and ONT. The long-read chemistry generated by ONT was useful for addressing challenges related to de novo assembly, particularly at regions containing repetitive sequences spanning longer than the read length, and which could not be resolved with only short-read–based assembly. Contig assembly of Illumina short reads gave an N50 of 1,639 bases, whereas with ONT, the N50 increased by >9-fold to 14,749 bases. The initial contig assembly based on Illumina sequencing reads alone gave 685,241 contigs. Further scaffolding on assembled contigs using both Illumina and ONT sequencing reads resulted in a final assembly of 15,025 super-scaffolds, with an N50 of ∼0.23 Mb. Ninety-five percent of proteins predicted by homology matched with those in a public repository, verifying the completeness of our assembly. Like other phylogenetic studies of avian conserved genes, we found *P. cristatus* to be most closely related to *Gallus gallus*, followed by *Meleagris gallopavo* and *Anas platyrhynchos*. Compared with the recently published peacock genome assembly, the current, superior, hybrid assembly has greater sequencing depth, fewer non-ATGC sequences, and fewer scaffolds.

## Data Description

### Background

**Figure 1:**
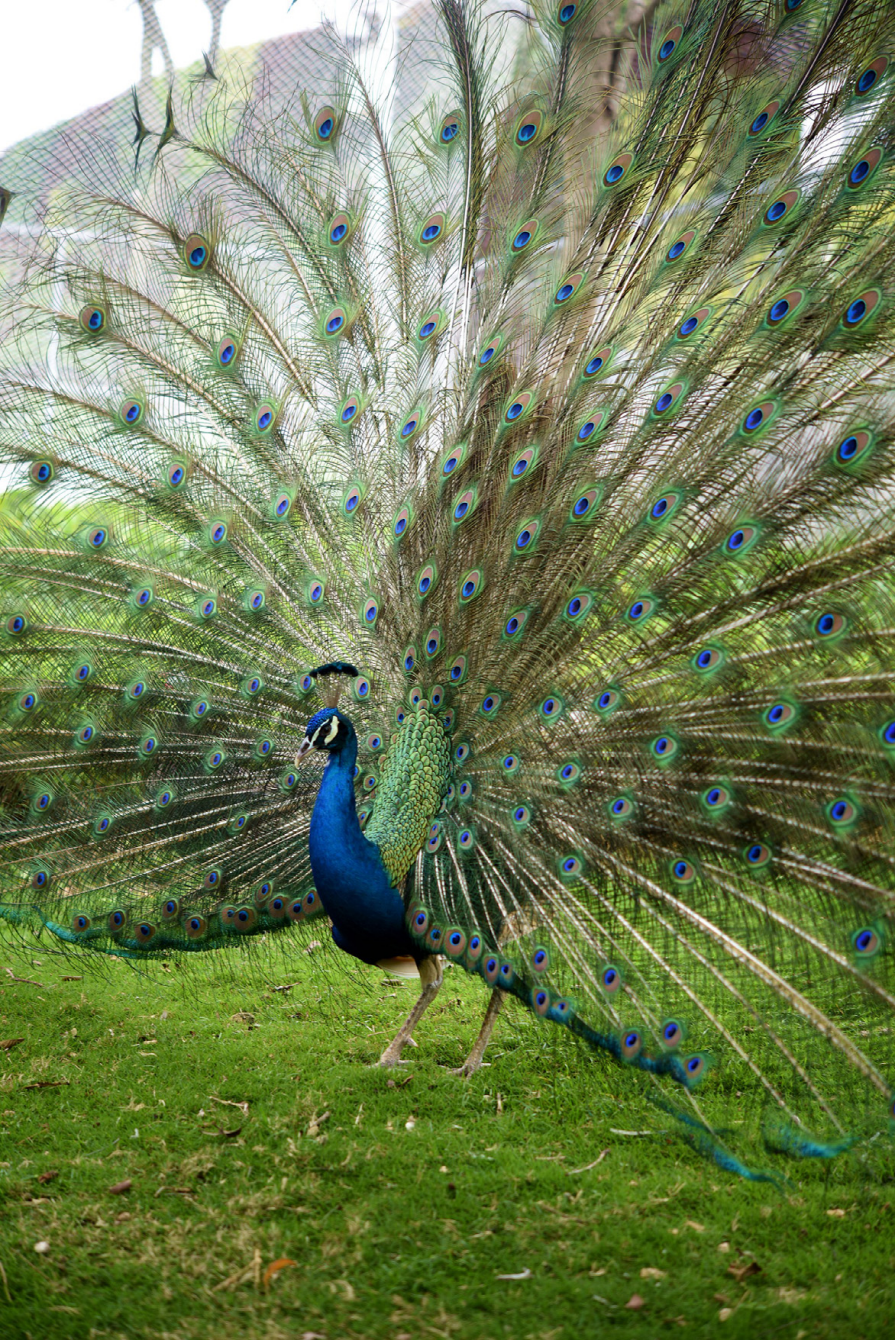
Photograph of the Indian blue peacock (*P. cristatus*).

*Pavo cristatus*, commonly known as the Indian blue peafowl, is native to South Asia. Apart from the wild, they are usually found as park and zoo exhibits or are raised for breeding and conservation purposes [1, 2] (Fig. 1). Peafowl have been widely featured in ancient Indian literature [3] and are closely associated with the life and culture of Southeast Asia, symbolizing beauty, love, grace, and pride [4, 5]. For these reasons, the peafowl—specifically the peacock—was chosen to be the national bird of India in 1963.

Genome sequencing of the avian model organism *Gallus gallus* (the red junglefowl, or chicken) [6] and other avian species [7] has provided novel perspectives on vertebrate genome evolution, such as a better understanding of genome structure and annotating the mammalian genome. Genome studies of *G. gallus* have revealed high conservation within orthologous regions of the human genome [8], thus showing promise as a good candidate for studies on developmental biology, immunology, and vertebrate genome architecture [9, 10].

Despite a wealth of information from existing avian genome-sequencing projects, it remains important to sequence the genomes of other species to add value to avian and vertebrate genomics. Here, we use Oxford Nanopore technology (ONT) to sequence a bird genome for the first time. The long reads generated from this sequencing technology were helpful during the de novo assembly of this genome, especially in the guanine-cytosine–rich repeat regions, which invariably pose serious challenges. By comparing this genome with those of other birds, we will understand more about the uniqueness of the peacock genome; the development of this species, its complex plumage pigmentation, and sexual dimorphism; and its evolutionary relationships with other birds. Characterization of genes and their specific functions will facilitate better understanding of the peafowl species. By comparing proteins between the peacock, chicken, and *Meleagris gallopavo* (domestic turkey), conserved domains and functional annotations may be revealed.

## Methods

### Sample collection and extraction of DNA

A blood sample was collected from an Indian male peacock (Fig. 1) at Kanpur Zoo, Kanpur, Uttar Pradesh, India, after the necessary ethical and institutional approvals were obtained.

DNA from blood was prepared for sequencing as follows: first, 200 µl of blood was added to a 1.5-ml microcentrifuge tube containing ∼20 µl of proteinase K solution, and briefly mixed. Cell lysis buffer (200 µl) was added to the tube, which was mixed by vortexing for 10 seconds, then incubated at 56°C for 10 minutes. Then, 250 µl of binding buffer (BBA) was added to the tube, which was mixed by vortexing again for 10 seconds. The contents of the tube were added to a ReliaPrep (Promega, Madison, WI, USA) binding column, which had been placed into an empty collection tube, then capped and placed in a refrigerated microcentrifuge. The binding column and tube were then centrifuged for 1 minute at 12,000 rpm and flow-through was discarded. The binding column was placed into a fresh collection tube, 500 µl of column wash solution was added, and then centrifuged for 3 minutes at maximum speed, again discarding flow-through. Column washing was repeated 3 times. Columns were then placed in a clean, nuclease-free 1.5-ml microcentrifuge tube. Nuclease-free water (100 µl) was then added to the column and centrifuged for 1 minute more at maximum speed before discarding the column and saving the elute.

The concentration and purity of the extracted DNA was evaluated using a Nanodrop 2000 spectrophotometer (Thermo Fisher Scientific, Waltham, MA, USA) and a Qubit fluorometer (Thermo Fisher Scientific), and integrity was checked on 0.8% agarose gel. The DNA sample was aliquoted for library preparation on 2 different platforms: Illumina HiSeq 4000 (Illumina, San Diego, CA, USA) and Oxford Nanopore Technology (Oxford, UK) MinION sequencing platform. The genome sequencing was performed by Genotypic Technology, Bengaluru, India, in accordance with standard protocols.

### Library preparation and sequencing

#### Paired-end library preparation and sequencing

Whole-genome sequencing libraries were prepared with an Illumina-compatible NEXTflex DNA sequencing kit (BIOO Scientific, Austin, TX, USA). Approximately 1 μg of genomic DNA was sheared using a Covaris S2 sonicator (Covaris, Woburn, MA, USA) to generate fragment sizes of ∼300–600 base pairs (bp). The fragment size distribution was checked using an Agilent 2200 TapeStation system with D1000 DNA screen tapes and reagents (Agilent Technologies, Palo Alto, CA, USA), and subsequently purified using HighPrep magnetic beads (Magbio Genomics Inc, Gaithersburg, MD, USA). The purified fragments were end-repaired, adenylated, and ligated to Illumina multiplex barcode adaptors, in accordance with the NEXTflex DNA sequencing kit protocol (BIOO Scientific, Austin, TX, USA).

The adapter-ligated DNA was purified with HighPrep beads (MagBio Genomics, Inc, Gaithersburg, MD, USA), then size selected on 2% low-melting agarose gel, and cleaned using a MinElute column (Qiagen, Hilden , Germany). The resulting fragments were amplified for 10 cycles of polymerase chain reaction (PCR) using the Illumina-compatible primers provided in the NEXTFlex DNA sequencing kit. The final PCR product (sequencing library) was purified with HighPrep beads, followed by a library quality control check. The Illumina-compatible sequencing library was initially quantified using a Qubit fluorometer (Thermo Fisher Scientific), and fragment size distribution was analyzed on an Agilent TapeStation. Finally, the sequencing library was quantified by quantitative PCR (qPCR) using the Kapa Library Quantification Kit (Kapa Biosystems, Wilmington, MA, USA). The qPCR-quantified library was sequenced on an Illumina sequencer for 150-bp paired-end (PE) chemistry.

For each sample, the Illumina-compatible sequencing library had a fragment size range of 275–425 bp for PE short inserts and 350–650 bp for PE long inserts. Because the combined adapter size was ∼120 bp, the effective user-defined insert size was 155–305 and 230–530 bp for PE short inserts and PE long inserts, respectively. Libraries were sequenced using the Illumina HiSeq platform [11] with 150 PE chemistry.

#### Mate-pair library preparation and sequencing

The mate-pair sequencing library was prepared using the Illumina-compatible NextEra Mate Pair Sample Preparation Kit (Illumina Inc., Austin, TX, USA). Approximately 4 µg of genomic DNA was simultaneously fragmented and tagged with mate-pair adapters in a transposon-based tagmentation step. Tagmented DNA was then purified using AMPure XP magnetic beads (Beckman Coulter Life Sciences, Indianapolis, IN, USA), followed by strand displacement to fill gaps in the tagmented DNA. Strand-displaced DNA was further purified with AMPure XP beads before size-selecting fragments of 3–5, 5–7, and 7–10 kilobase pairs (kb) on low-melting agarose gel. The fragments were circularized in an overnight blunt-end intra-molecular ligation step, which resulted in circularization of DNA with the insert mate-pair adapter junction. Circularized DNA was sheared using a Covaris S220 sonicator (Covaris, Woburn, MA, USA) to generate approximate fragment sizes of 300–1000 bp. The sheared DNA was purified to collect the mate-pair junction-positive fragments using Dynabeads M-280 streptavidin magnetic beads (Thermo Fisher Scientific). The purified fragments were end-repaired, adenylated, and ligated to Illumina multiplex barcode adaptors, in accordance with the NextEra Mate Pair Sample Preparation Kit protocol.

The adapter-ligated DNA was then amplified for 15 cycles of PCR using Illumina-compatible primers. The final PCR product (sequencing library) was purified with AMPure XP beads, followed by a library quality control check. The Illumina-compatible sequencing library was initially quantified using a Qubit fluorometer (Thermo Fisher Scientific), and its fragment size distribution was analyzed with an Agilent TapeStation. Finally, the sequencing library was accurately quantified by qPCR using the Kapa Library Quantification Kit (Kapa Biosystems). The qPCR-quantified libraries were pooled in equimolar amounts to create a final multiplexed library pool for sequencing on an Illumina sequencer.

#### ONT MinION library preparation and sequencing

Genomic DNA (1.5 μg) was end-repaired using the NEBnext Ultra II End Repair kit (New England Biolabs, Ipswich, MA, USA) and cleaned up with 1x volume of AMPure beads (Beckmann Coulter, USA). Adapter ligations were performed for 20 minutes using NEB blunt/TA ligase (New England Biolabs). The library mixtures were cleaned up using 0.4X AmPure beads (Beckmann Coulter) and eluted in 25 μl of elution buffer. The eluted library was used for sequencing. Whole-genome libraries were prepared using the ligation sequencing SQK-LSK108 Oxford Nanopore sequencing kit (ONT). Sequencing was performed on a MinION Mk1b (ONT) using SpotON flow cell (FLO-MIN106) in a 48-hour sequencing protocol on MinKNOW (version 1.1.20, ONT).

### Raw data quality control and processing

#### Illumina raw data: quality control and processing

Illumina reads were de-multiplexed using bcl2fastq (Illumina). Raw genomic library data generated by Illumina was quality-checked using FastQC (FastQC, RRID:SCR_014583) [12]. PE Illumina reads were processed for clipping adapter and low-quality bases using a customized script that retains a minimum of 70% bases/reads with Phred score (Q ≥ 30 in each base position) with a read length of 50 bp. Mate-pair libraries were trimmed for adapter sequences and low-quality bases, trimming from the 3-end using the PLATANUS internal trimmer (Platanus version 1.2.4, RRID:SCR_015531) [13].

#### ONT reads: base calling and processing

Raw data were base-called with the cloud-based Metrichor workflow 2D Basecalling plus Barcoding (Metrichor version 2.43.1, ONT, Oxford, UK [14]. ONT reads were processed using Poretools [15] to convert fast5 files to fasta format. The 2D reads or 1D high-quality reads were selected for further assembly.

### De novo genome assembly and genome size estimation

**Figure 2:**
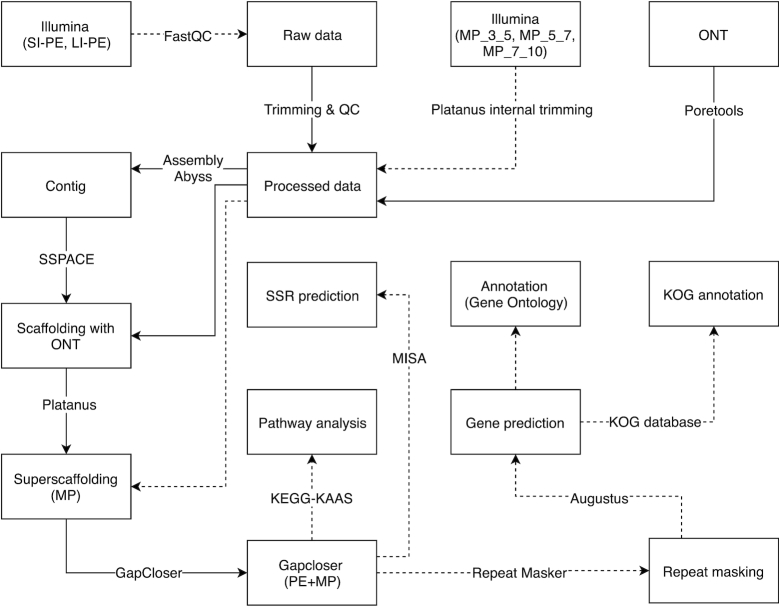
Detailed workflow for de novo whole-genome assembly and annotation. LI-PE: long-insert paired-end; QC: quality control; SI-PE: short-insert paired-end.

Quality-checked ONT reads were error-corrected using Illumina PE reads. For error correction, the Illumina PE reads were aligned to the ONT reads using BWA aligner (BWA, RRID:SCR_010910) [16]. PE reads were assembled using Abyss (ABySS, RRID:SCR_010709) [17], followed by contig extension using ONT reads using SSPACE-LongRead [18]. Super-scaffolding of the assembled scaffold was performed using SSPACE (SSPACE, RRID:SCR_005056) [19] and PLATANUS on the ONT and mate-pair data. A final draft genome resulted after gap closure using GAPCLOSER (GapCloser, RRID:SCR_015026) [20] and the PLATANUS gap_close tool, with Illumina data. The genome size was estimated with a k-mer distribution plot using JELLYFISH (Jellyfish, RRID:SCR_005491) [21]. The assembly and annotation workflow is shown in Fig. 2.

### Identification of repetitive elements and simple sequence repeat markers

Repetitive elements, retrotransposons, and DNA transposons were identified in the draft genome and hard-masked by using reference genomic repeats of *G. gallus* using Repeatmasker (RRID:SCR_012954) [22]. Final assembled scaffolds were analyzed to identify simple sequence repeats (SSRs). SSRs, such as di-, tri-, tetra-, penta-, and hexa-nucleotide repeats in the genome, were identified using MISA (version 1.0.0) [23].

### Annotation of the draft genome

Gene models were predicted on a hard-masked draft genome using AUGUSTUS (RRID:SCR_008417) [24], with *G. gallus* as a reference model. Predicted proteins were annotated using BLASTP (RRID:SCR_001010) [25] against the National Center for Biotechnology Information Non-redundant (NCBI NR) database, with default parameters at an E-value cutoff of 1E–5.

Predicted proteins were searched against the Kyoto Encyclopedia of Genes and Genomes’ Automatic Annotation Server (KEGG-KAAS) for pathway analysis [26]. *G. gallus, M. gallopavo, Taeniopygia guttata* (zebra finch), and *Falco peregrinus* (peregrine falcon) were used as reference organisms for pathway identification. Eukaryotic orthologous groups (KOGs) [27] were predicted using a homology-based approach.

### Prediction of protein domains

Predicted proteins from peacock, chicken, and turkey, with sequence lengths >100 amino acids, were considered for protein domain analysis. All protein-coding sequences from each organism were searched against the Pfam-A database using Pfam scan [28].

### Identification of avian protein families

A total of 748,544 protein sequences from 49 avian species (including peacock proteins from this study) and others were downloaded from the Avian Phylogenomics Project [29, 30]. Sequences with lengths >100 amino acids from all the avian genomes were selected and concatenated to a single fasta file. These sequences were clustered using CD-HIT [31], with 70% alignment coverage for the shorter sequences, with a length difference cutoff of 0.7. Single-copy gene family orthologs present across all avian species, and not clustered peacock proteins, were annotated.

### Phylogenetic tree construction

To construct a phylogenetic tree, we considered single-copy gene clusters present as single copies in all the avian species analyzed. These protein sequences from each species were concatenated and further aligned using the multiple sequence alignment tool Clustalw [32]. Poorly aligned positions and divergent regions were removed using Gblock [33]. Sequences in fasta format were converted to phylip format using Phylip [34]. Phylogenetic trees were constructed using IQ-TREE (version 1.5.6) [35]. The parameters used to construct the phylogenetic tree were ultrafast bootstrap (UFBoot, using the –bb option of 1,000 replicates), and a standard substitution model (–st AA –m TEST), and alrt 1000 –nt AUTO was given to generate the tree. Trees generated from IQ-TREE were visualized using FigTree [36], and the branch-support values were recorded from the output “.treefile”. For better visualization, trees were modified under the “Trees” section, and increasing order nodes were applied.

### Genome conservation analysis

Draft chromosome visualizations were constructed by aligning the assembled peacock genome against that for *G. gallus* using the Chromosomer tool [37]. The reordered, assembled genome was aligned to the chicken genome using LAST aligner [38], with NEAR (finding short-and-strong [near-identical] similarities) parameters to allow for substitution and gap frequencies, leading to the identification of orthologs. For visualization, these query-mapped regions were filtered for >1% of the maximum length using Circos [39].

## Results

### Genome sequencing assessment

Five libraries were generated from 150-bp paired-end (PE) Illumina sequences. Short-insert reads (489,114,747) represented genome coverage of 146.7×, and 302,884,819 long-insert reads represented ∼90.9× coverage, with a total coverage of 237.6×. Sequencing of 3 mate pairs of 3–5, 5–7, and 7–10 kb yielded 72,915,033, 47,440,144, and 36,464,628 reads, respectively, with an approximate coverage of 21.9×, 14.2×, and 10.9×, respectively, and a grand total of 156 million mate-pair reads representing 47× coverage.

ONT was used to generate 366,323 long reads, having 2,398,560,283 bp and coverage of 2.3×. The complete genome was sequenced to a depth of ∼287×, using both Illumina and ONT platforms (Table 1). Coverage was based on the assumption that the peacock genome is 1 gigabase (Gb) in size.

**Table 1:** Raw data statistics of peacock genome reads generated by Illumina HiSeq and ONT

Sample	Platform	Library and chemistry	No. of reads	Coverage	Sequence Read Archive ID
SO_6221_SKPea2016_SI	HiSeq	PE-SI (150 * 2)	489,114,747	146.73	SUB3108018, SAMN07739105
SO_6221_SKPea2016_LI	HiSeq	PE-LI (150 * 2)	302,884,819	90.87	SUB3108017, SAMN07739104
SO_6221_FPL_3_5KB	HiSeq	MP (150 * 2)	72,915,033	21.87	SUB3107930, SAMN07739101
SO_6221_FPL_5_7KB	HiSeq	MP (150 * 2)	47,440,144	14.23	SUB3108015, SAMN07739102
SO_6221_FPL_7_10KB	HiSeq	MP (150 * 2)	36,464,628	10.94	SUB3108016, SAMN07739103
SO_6221_NP	ONT	5–341,124	366,323	2.3	SUB3108020, SAMN07739107

Abbreviations: KB, kilobases; LI, long insert; MP, mate-pair; PE, paired-end; SI, short insert.

### Genome assembly

The first assembly was based on Illumina reads only, using the Abyss *de novo* assembler, which resulted in a genome size of ∼932 megabase pairs (Mb) and an N50 of 1,639 bp. Contig extension was performed using ONT-generated reads, which gave scaffolds with an N50 of 14,748 bp. SSPACE and PLATANUS were used to super-scaffold the assembled scaffold with mate-pair libraries, which generated a genome size of ∼916 Mb and an N50 of 168,140 bp. Finally, gaps were closed using GAPCLOSER with mate-pair and PE long-insert libraries, which generated a draft genome size of 1.02 Gb.

The draft genome assembly of *P. cristatus* comprises 179,346-bp scaffolds, with an N50 of 189,886 bp with 37 scaffolds, having a sequence length ≥1 Mb. Contigs >5,000 bp in length covered a genome of ∼0.915 Mb, with an N50 of 0.23 Mb. In the assembled genome, there were ∼0.4% non-ATGC (adenine, cytosine, guanine, thymine) characters (Table 2).

**Table 2:** De novo assembly statistics of the peacock genome

Description	Contigs	ONT scaffolds	Super-scaffolds	GapClosed	>1,000 kb	>5,000 kb
Contigs	685,241	281,272	179,346	179,332	34,178	15,025
Maximum length	49,159	251,510	2390,121	2,488,982	2,488,982	2,488,982
Minimum length	300	5	265	265	1,000	5,000
Mean length	1,360	3,250	5,111	5,729		
Total length	932,162,464	914,363,908	916,720,956	1,027,510,962	954,449,349	915,342,012
Length ≥ 100 bp	685,241	281,271	179,346	179,332	34,178	15,025
Length ≥ 200 bp	685,241	281,271	179,346	179,332	34,178	15,025
Length ≥ 500 bp	616,120	186,433	93,727	93,718	34,178	15,025
Length ≥ 1 kb	363,428	104,479	34,168	34,178	34,178	15,025
Length ≥ 10 kb	1,591	24,748	9,249	10,310	10,310	10,310
Length ≥ 1 Mb	0	0	27	37	37	37
Non-ATGC No.	350,325	42,696,911	49,169,831	4,043,129	4,040,790	3,986,487
Non-ATGC percentage	0.038	4.67	5.36	0.393	0.423	0.436
N50 value	1,639	14,748	168,140	190,304	218,023	232,312

### Repetitive genome elements and SSR markers

It was estimated that 75 Mb (7.33%) of the peacock genome consisted of repeat sequences (Table S1). Approximately 56 Mb (5.5%) of class I retrotransposons were identified (long interspersed nuclear elements [LINEs], 4.7%; short interspersed nuclear elements [SINEs], 0.08%; and total long terminal repeat elements, 0.72%). Subsequently, 7,277,390 bp (0.71%) class II DNA transposons and 467,719 (0.05%) unclassified elements were identified (Table S1). The median percentages of LINEs, SINEs, long terminal repeats, DNA, unknown, and total masked bases of other avian birds were 3.94, 0.11, 1.31, 0.22, 0.85, and 6.93, respectively (Table S2). A total of 399,493 SSRs were obtained from the peacock genome assembly. The largest fraction of SSRs identified were mononucleotides (60.04%), followed by tetranucleotides (25.99%), dinucleotides (8.51%), trinucleotides (4.31%), pentanucleotides (1.03%), and hexanucleotides (0.13%). Among these SSRs, A (49.2%) and T (44.9%) accounted for 94.1% of the mononucleotide repeats. AT (23.8%), TA (16.5%), TG (13.7%), AC (10.6%), and CA (10.3%) accounted for 75% of the dinucleotide repeats, whereas TTG (9.9%), AAT (9.6%), AAC (9.4%), TTA (7.1%), ATT (4.5%), TAA (3.5%), CAA (3.1%), and GGA (2.7%) accounted for 49.7% of the trinucleotide repeats (Table S3).

### Gene prediction and annotation

**Figure 3:**
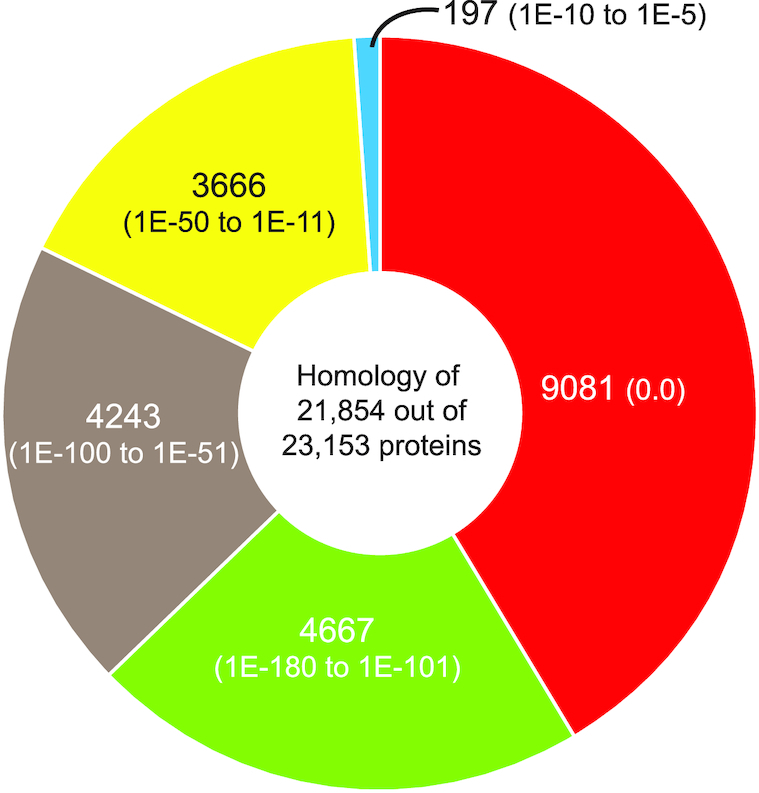
Peacock proteins showing homology. Pie chart showing significant similarity scores of peacock proteins against the NCBI NR database. The pie chart colors are grouped based on the E-value scores from most significant E-value of 0.0 (red) going clockwise to least significant of ∼1E–5 (blue).

A total of 23,153 proteins were predicted from the assembled draft peacock genome using AUGUSTUS. Of these, 21,854 (94.4%) predicted proteins showed homology to other sequences from the NCBI NR database (Fig. 3). The top 4 organisms with which peacock proteins showed homology were *G. gallus* (11,398 proteins), *M. gallopavo* (4,059 proteins), *Amazona aestiva* (blue-fronted Amazon parrot; 1,352 proteins), and *Anas platyrhynchos* (mallard duck; 849 proteins). Detailed annotations of all proteins are available in Table S4.

Gene ontology (GO) descriptions were assigned for 18,294 (79%) peacock proteins. Of these, 14,489 proteins were identified as having molecular function, 11,678 as biological processes, and 13,735 proteins as cellular components (Table S4).

A total of 4,091 (17.7%) peacock proteins had pathway information from the KEGG database (Table S5), whereas 20,937 (90.4%) peacock proteins were similar to KOG annotations (Table S6). When peacock proteins were searched against human proteins, gene family expansions were found in cell morphogenesis, neuronal projection and development, and GTPases (Table S7 and Fig. S3).

### Analysis of avian protein families

From a total of 748,544 protein sequences from 49 avian species, 653,497 protein sequences were found to have a length of ≥100 amino acids (Table S8A). On the basis of their level of identity, CD-HIT clustered the proteins into 114,121 gene clusters. Of these, 68 highly homologous gene clusters were present as single copies in all 49 avian species (Table S8B and S8C). We also observed 13,860 peacock protein clusters that were not clustered with other avian species (Table S8D).

### Phylogenetic analysis

**Figure 4:**
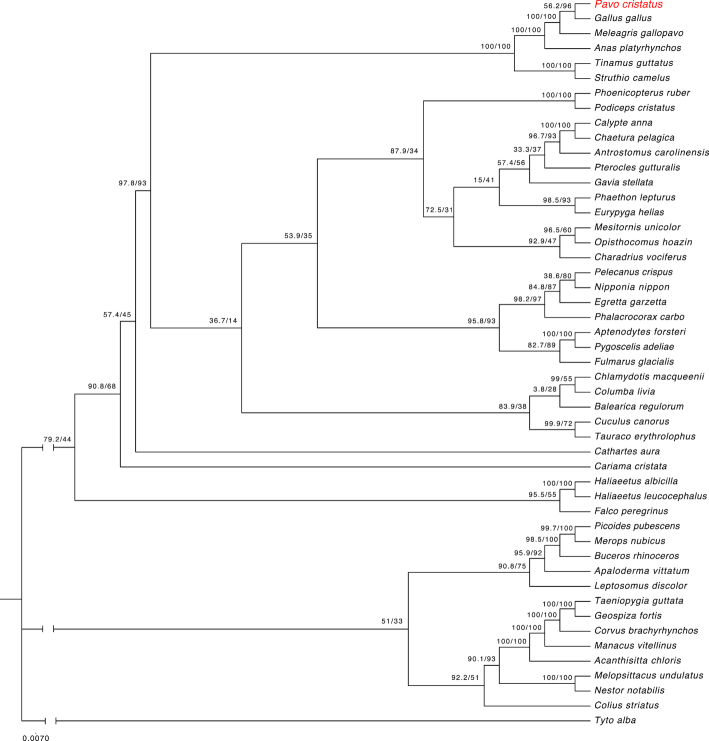
Phylogenetic tree generated from homologous proteins from 49 different avian species.

Phylogenetic analysis of 48 avian species and peacock proteins showed *P. cristatus* to be clustered in a clade with *G. gallus, M. gallopavo, A. platyrhynchos, Tinamus guttatus* (white-throated tinamou), and *Struthio camelus* (ostrich). This is the largest clade, with 6 species, having bootstrap support of 100. All species within this clade, except the mallard duck, are flightless or low-flying birds. Bootstrap support between *P. cristatus* and *G. gallus* was 96, followed by *M. gallopavo*, with bootstrap support of 100 (Fig. 4).

### Comparison with other species and databases

**Figure 5:**
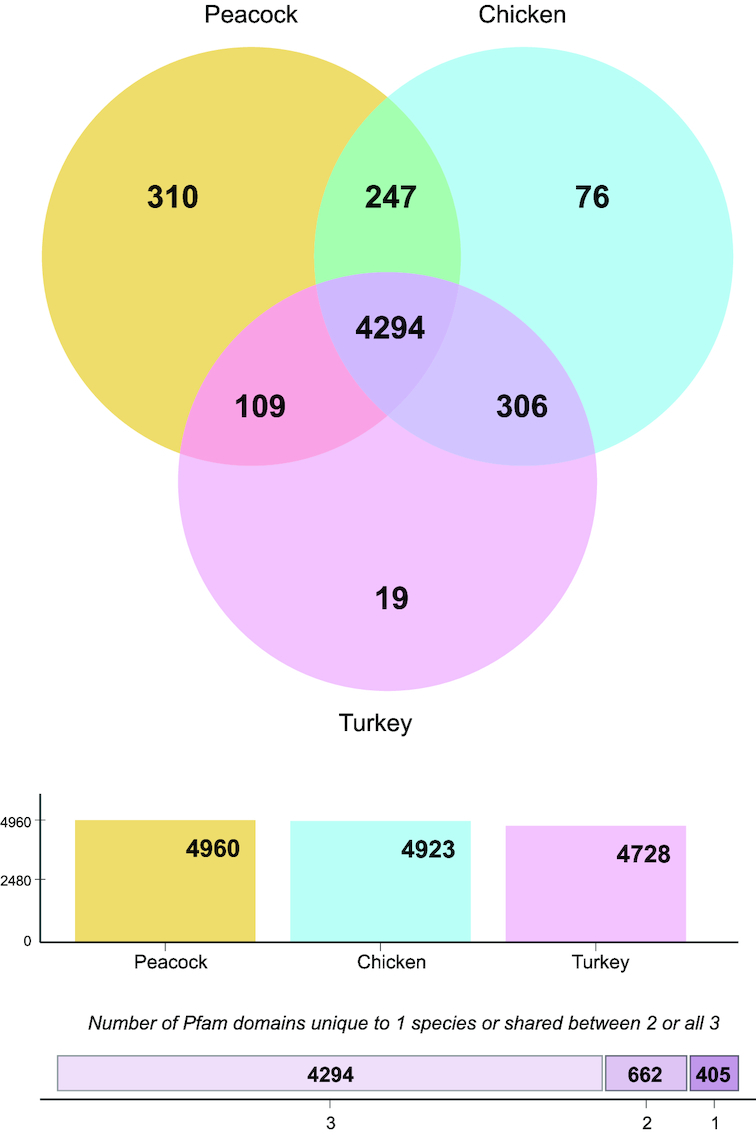
Venn diagram showing common and absent Pfam domains between peacock, chicken, and turkey proteins.

Searching Pfam for conserved protein domains between the predicted proteins from peacock, chicken, and turkey revealed that ∼81% of domains were common to these 3 species (Fig. 5, Table S9). Compared with the total number of protein family (Pfam) domains from these 3 species, 92.52%, 91.83%, and 88.19% Pfam domains were present in peacock, chicken, and turkey, respectively, but 310, 76, and 19 Pfam domains were absent between the species comparisons, respectively (Table S9H).

**Figure 6:**
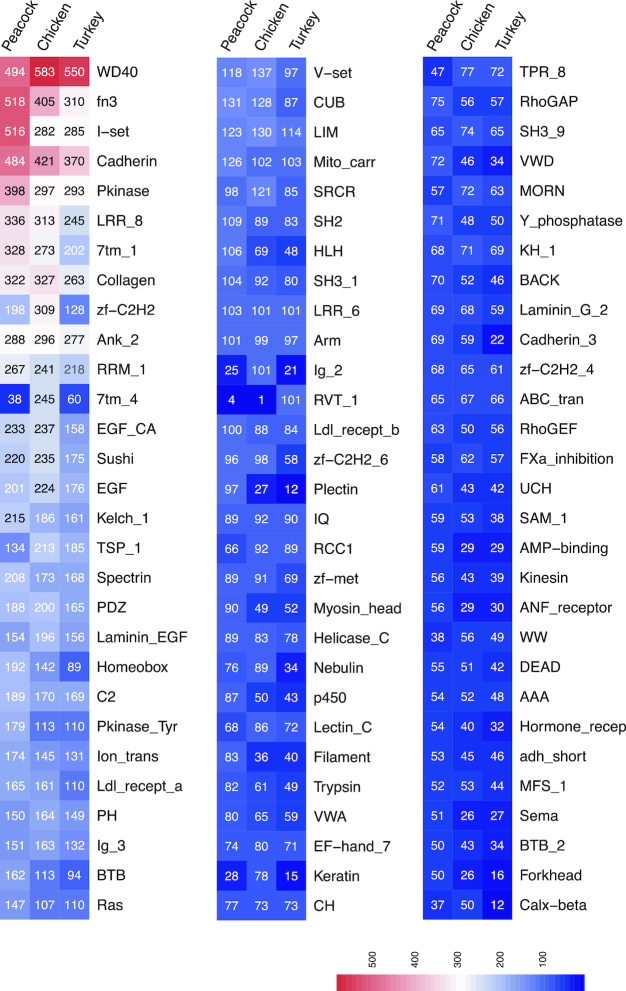
Heat map showing Pfam domains distributed in peacock, chicken, or turkey species. The number represents the Pfam domain count predicted from the protein sequences. Pfam domains of 50 and above identified in any 1 of the species are compared in the heat map.

There were 15,470 (78%), 12,794 (85%), and 11,745 (85%) of the peacock, chicken, and turkey proteins found to match with Pfam domains, respectively (Table S9). Domain comparisons between these species showed gene family expansions such as kinases, zinc finger proteins, GTPases, and others, in any 1 of the species (Fig. 6).

**Figure 7:**
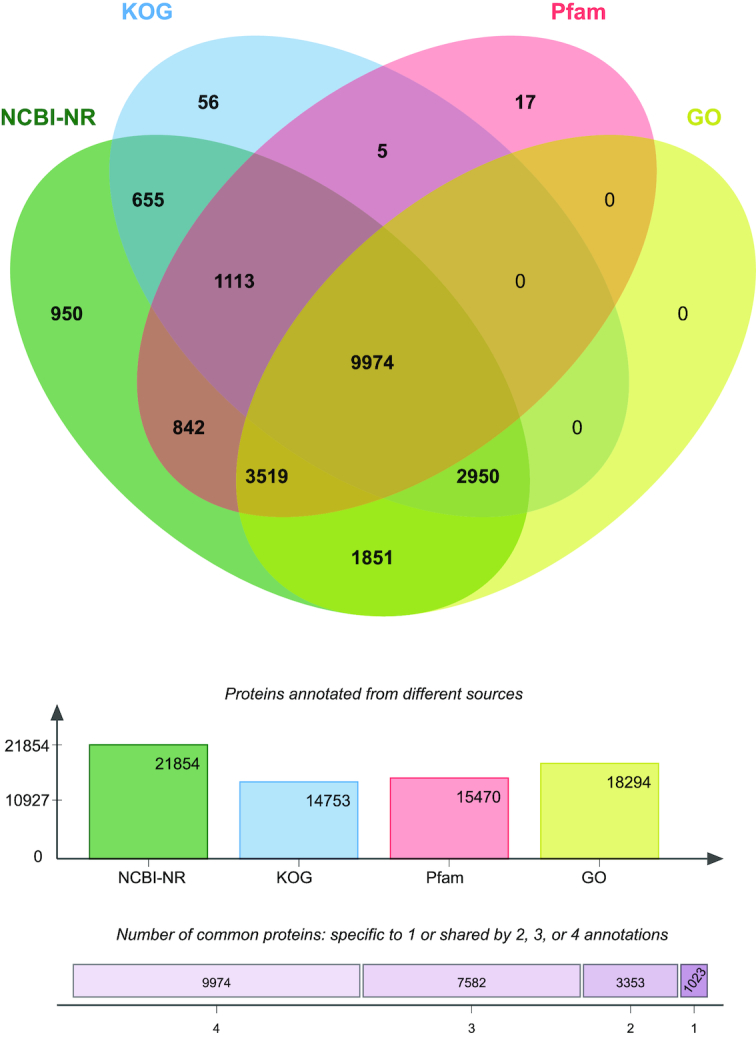
Venn diagram showing peacock proteins with significant homology to the NCBI NR database, the EuKaryotic Orthologous Groups (KOG) database, and Pfam and GO ontologies.

**Figure 8:**
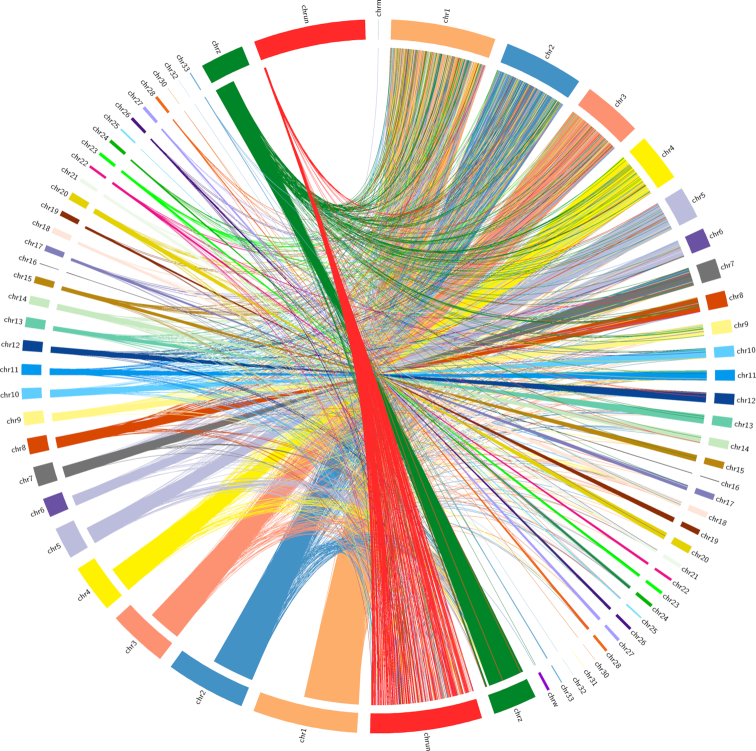
Circular image of the assembled peacock genome, aligned against the *G. gallus* genome. The right side of the image represents the reference chicken genome; left side represents the peacock genome.

A total of 9,974 peacock proteins were annotated in all 4 databases (NCBI NR, KOG, Pfam, and GO) (Fig. 7). When reordered for the generation of pseudo-chromosomes, 597 Mb of the assembled peacock genome was reordered peacock genome compared with the 1.21-Gb masked chicken genome [40] (Fig. 8).

Approximately 60 different avian species have been sequenced using various sequencing technologies (Table S10). The depth of these sequences varies, from as low as 6× to as high as 390× coverage. These results, which were obtained using different bioinformatics methods to assemble the sequencing data, are measured as scaffold N50; i.e., from 30 kb to 14 Mb.

## Discussion and Conclusions

In recent years, there has been a rapid surge in the de novo genome sequence assembly of diverse species [41]. This surge has largely been driven by a more affordable cost per base sequencing, and the development of smarter algorithms that have been refined and equipped to handle large datasets [42–44]. The challenge for newer genome analysis pipelines is to generate assemblies with lower contig numbers and longer contigs per genome. To achieve this, technologies that generate longer reads or greater read depths are very helpful [45], but the use of combinations of different sequencing technologies also plays a significant role in improving genome assemblies [46] (Table S10). Libraries generated using >1 type of chemistry have been found to generate superior assemblies [47] and have been shown to reduce the number of scaffolds—even with very low coverage. Thus, we need to consider combinations of sequencing technologies, along with the use of different bioinformatics software programs, to obtain assemblies with fewer scaffolds, or which are closer to chromosome-level sequencing [48].

Compared with other avian genomes [49], the sequencing depth of 290× that we achieved for the peacock is one of the highest. The final draft peacock genome assembly resulted in an N50 of 0.23 Mb. Including 2.3× of reads generated by ONT helped to improve the assembly by reducing the number of scaffolds by 26.2% and increasing the scaffold and contig N50s by ∼50.7% and 115%, respectively.

The draft assembly contained <0.4% unknown nucleotides, which is very low for a draft assembly. Our hybrid peacock assembly outperforms the currently available draft peacock assembly (Table S11) by sequencing 6 different libraries, including long reads from ONT, and 2.1-fold increased sequencing data generation. Greater sequencing depth and the use of multiple libraries enabled us to obtain a better assembly with 6.6-fold fewer scaffolds and an improvement in N50 length by 9.1-fold. The longest scaffold in our assembly is 8.7-fold longer than in the previously published draft assembly and has a 5-fold lower percentage of non-ATGC. Thus, for the first time in avian genomics, we have demonstrated how low-cost, third-generation sequencing data generated by ONT can help to improve draft genome assembly. Assemblies with longer scaffolds will further help us to understand more about organisms with structurally complex genomic regions, repeat elements, and isoforms [39].

Our confidence in the peacock proteins predicted from our assembly was strengthened when we discovered that ∼95% of them showed significant homology to various genomic features from different databases (Fig. 7). Based on proteins conserved across the avian species, our phylogenetic analysis revealed that the peacock is most closely related to the chicken, followed by turkey and duck. This concurs with previous data based on mitochondrial phylogeny [50]. Thus, our genome sequence provides further insight into the peacock's genetic lineage and evolution with respect to other avian species. The estimated median divergence time of *P. cristatus* from *G. gallus* is ∼35 million years ago, whereas the divergence time estimated between *G. gallus* and *M. gallopavo* is ∼37 million years ago [51]. The large gap between peacock and other avians may be attributed to the non-availability of other avian genome sequences. The gap may be closed by sequencing other avian species.

Among the vertebrates, it has been observed that variations in transposable elements (TEs) between avians are very low [52] (Table S8). The genome complexities of a species are influenced by the TEs, which are believed to play a crucial role [53]. In this peacock genome assembly, inclusion of ONT long-read sequences has substantially improved the assembly, thus helping to resolve repetitive regions across the genome. The roles of TEs in the development and evolution of the peacock should be further explored.

Information about the peacock genome will be valued, and may be explored, by avian enthusiasts to further understand the avian world. Although not yet critically endangered in India, the wild peafowl population is declining because of massive deforestation, habitat loss [54], and increased poaching for their meat and feathers. Our *P. cristatus* genome sequencing initiative is valuable not only from a conservational viewpoint but also to preserve the history and heritage that is associated with this bird, which has a strong hold on the national psyche.

## Availability of supporting data and materials

The datasets supporting the results of this article are available on the study website [55] and the *GigaScience* GigaDB repository [56].

Raw reads (Illumina and ONT) are available in the Sequence Read Archive database, and the whole-genome shotgun project has been deposited at GenBank under Sequence Read Archive submission ID SUB3108024, Bioproject PRJNA413288, and biosamples SUB3108018/SAMN07739105: SKPea2016_SI, SUB3108017/SAMN07739104: SKPea2016_LI, SUB3107930/SAMN07739101: FPL_3_5KB, SUB3108015/SAMN07739102: FPL_5_7KB, SUB3108016/SAMN07739103: FPL_7_10KB, and SUB3108020/SAMN07739107: FPL_Nano (Table 1).

The de novo genome assembly can be accessed under SUB4504869/SAMN07739105.

## Additional files

**Figure S1:** Number of proteins showing similarity to protein family (Pfam) domains.

**Figure S2:** Distribution of top 10 GOs in biological process, cellular component, and molecular function categories represented as a pie chart.

**Figure S3:** Comparison of peacock and human protein orthologs and their GO annotations, represented as a word cloud to show significant ontology descriptors.

Geneids were converted to Ensembl Ids using G: Convert [57] and GOs were evaluated using GO: Summaries [58].

**Table S1:** Statistical analysis of repeat masking for the peacock genome.

**Table S2:** Statistical analysis of repeats in the peacock genome compared with other bird species.

**Table S3A:** Overview of simple sequence repeats (SSRs).

**Table S3B:** Simple sequence repeats (SSRs) identified in the peacock genome.

**Table S4:** Protein database annotations for predicted peacock genes, using BLAST software.

**Table S5:** Kyoto Encyclopedia of Genes and Genomes (KEGG) annotations for all peacock proteins.

**Table S6:** EuKaryotic Orthologous Groups (KOG) annotations for all peacock proteins.

**Table S7:** Peacock proteins showing remote orthologous sequence relationships with human proteins, using BLAST software.

**Table S8A:** Count of proteins in different bird species.

**Table S8B:** Orthology, clustering, and annotation of proteins from different bird species.

**Table S8C:** Single-copy orthologous proteins present in all bird species.

**Table S8D:** Proteins uniquely present in peacock.

**Table S9A-G:** Protein family (Pfam) protein domains identified in peacock, chicken, and turkey.

**Table S10:** Raw sequencing data, total scaffolds with N50 obtained for different avian species.

**Table S11:** Comparison of 2 different peacock assemblies.

GIGA-D-18-00280_Original_Submission.pdf

GIGA-D-18-00280_Revision_1.pdf

GIGA-D-18-00280_Revision_2.pdf

GIGA-D-18-00280_Revision_3.pdf

GIGA-D-18-00280_Revision_4.pdf

Response_to_Reviewer_Comments_Original_Submission.pdf

Response_to_Reviewer_Comments_Revision_1.pdf

Response_to_Reviewer_Comments_Revision_2.pdf

Response_to_Reviewer_Comments_Revision_3.pdf

Reviewer_1_Report_Original_Submission -- Matthew Greenwold 8/24/2018 Reviewed

Reviewer_1_Report_Revision_1 -- Matthew Greenwold 10/18/2018 Reviewed

Reviewer_2_Report_Original_Submission -- Daniel Ence 8/27/2018 Reviewed

Reviewer_2_Report_Revision_1 -- Daniel Ence 10/22/2018 Reviewed

Reviewer_2_Report_Revision_2 -- Daniel Ence 1/15/2019 Reviewed

Supplemental Files

## Abbreviations

ATGC: adenine, cytosine, guanine, thymine; bp: base pairs; Gb: gigabase pairs; kb: kilobase pairs; KEGG: Kyoto Encyclopedia of Genes and Genomes; KOG: eukaryotic orthologous groups; LINE: long interspersed nuclear element; Mb: megabase pairs; NCBI NR: National Center for Biotechnology Information Non-redundant; ONT: Oxford Nanopore technology; PCR: polymerase chain reaction; PE: paired end; Pfam: protein family; qPCR: quantitative polymerase chain reaction; SINE: short interspersed nuclear element; SSR: simple sequence repeat; TE: transposable element.

## Competing interests

The authors declare that they have no competing interests.

## Funding

R.D. provided partial funding. This work was also supported by Estonian Research Council ETAG grants IUT 34–4 to B.R.

## Authors’ contributions

R.D., A.S., and K.P. performed the wet lab experiments; R.D. designed the work plan, experiments, and logistics; S.S., V.R., K.P., S.G., I.M., and A.R. assisted with the work; R.S. provided bird samples; B.R. and S.K. analyzed and interpreted the data and drafted the manuscript. S.K. oversaw the whole project. All authors read and approved the final version of the manuscript.
