# Supplementary material for: De novo assembly of the Indian blue peacock (Pavo cristatus) genome using Oxford Nanopore technology and Illumina sequencing
Source: Gigascience. 2019 May 11;8(5):giz038. doi: 10.1093/gigascience/giz038 (PMC6511069; doi:10.1093/gigascience/giz038)
Supplement: GIGA-D-18-00280_Original_Submission.pdf [file giz038_giga-d-18-00280_original_submission.pdf]

GigaScience

De novo genome assembly of Indian Blue Peacock (Pavo cristatus) from Oxford Nanopore and Illumina sequencing reads

--Manuscript Draft--

**Manuscript Number:**	GIGA-D-18-00280
**Full Title:**	De novo genome assembly of Indian Blue Peacock (Pavo cristatus) from Oxford Nanopore and Illumina sequencing reads
**Article Type:**	Data Note
**Funding Information:**	
**Abstract:**	Background<br><br>Pavo cristatus the Indian blue peacock are geographically found distributed in natural habitats of South Asia. Peacock has been described among the bird species as one of the most elegant, majestic and beautiful bird (Fig. 1). Since prehistoric times they have been described or depicted in Indian culture and has been adopted as the national bird of India. Its length varies from 92-125 centimeter (without train), weighing about 4-8 Kilograms and lives up to 20 years in the wild. The avian species have been very important in the fields of phylogenetics, developmental studies, sexual reproduction and speciation. Avian genomics have contributed immensely towards understanding the vertebrate genome evolution. Here we present the first draft genome sequence of P. cristatus, yet another important bird species to further add values and gain insight into avian genomics.<br><br>Findings<br><br>For the first time in avian genomics, long reads using Oxford Nanopore technology have been used for the whole genome assembly. We sequenced different DNA insert size libraries from Illumina and long read Nanopore technologies from the peacock DNA. We performed de novo genome assembly by integrating the reads from Illumina short insert, long insert, multiple mate-pair reads along with Nanopore long reads using multiple genome improvement tools. A draft of the peacock genome of about 0.915 Gigabases (Gb) with a N50 of 0.23 Megabases (Mb) was assembled. Annotations with other avian species, protein families, KEGG were performed for functional understanding by in-silico approaches. Proteins were compared against Chicken, Turkey and Human to obtain evolutionary similarities and uniqueness of the Pavo species.<br><br>Conclusions<br><br>Our most important findings from the genome sequence of P. cristatus is to decipher the different gene families and to understand their role in body pattern development and other features that truly makes this bird unique. The genome sequence also gives insights on its genetic lineage and evolution with relation to other avian members. Several hypothesis and theories have been discussed with respect to sexual selection; now with the understanding of the genome sequence, some of these evolutionary theories will be better understood. The genome will also support future studies on population genetics and breeding for species conservation as well as in understanding its evolutionary ecology and sexual dimorphism. The comparative genomics with other avian species and specifically with Gallus gallus (Chicken) and Meleagris gallopavo (Turkey) have shown insights into the gene families and their conserved domains. Pavo proteins were also compared with human to understand the functional components that were conserved after the speciation split.
**Corresponding Author:**	Subhradip Karmakar, PhD<br>All India Institute of Medical Sciences<br>New Delhi, Delhi INDIA
**Corresponding Author Secondary Information:**	

Powered by Editorial Manager® and ProduXion Manager® from Aries Systems Corporation

Corresponding Author's Institution:	All India Institute of Medical Sciences
Corresponding Author's Secondary Institution:	
First Author:	Subhradip Karmakar, PhD
First Author Secondary Information:	
Order of Authors:	Subhradip Karmakar, PhD
	Ruby Dhar
	Ashikh Seethy
	Karthikeyan Pethusamy
	Vishwajeet Rohil
	Sunil Singh
	Kakali Purkayastha
	Sandeep Goswami
	Rakesh Singh
	Indrani Mukherjee
	Ankita Raj
	Tryambak Srivastava
	Sovon Acharya
	Balaji Rajashekhar
Order of Authors Secondary Information:	
Additional Information:	
Question	Response
Are you submitting this manuscript to a special series or article collection?	No
**Experimental design and statistics**<br><br>Full details of the experimental design and statistical methods used should be given in the Methods section, as detailed in our Minimum Standards Reporting Checklist. Information essential to interpreting the data presented should be made available in the figure legends.<br><br>Have you included all the information requested in your manuscript?	Yes
**Resources**<br><br>A description of all resources used, including antibodies, cell lines, animals	Yes

Powered by Editorial Manager® and ProduXion Manager® from Aries Systems Corporation

and software tools, with enough information to allow them to be uniquely identified, should be included in the Methods section. Authors are strongly encouraged to cite Research Resource Identifiers (RRIDs) for antibodies, model organisms and tools, where possible.<br><br>Have you included the information requested as detailed in our Minimum Standards Reporting Checklist?	
**Availability of data and materials**<br><br>All datasets and code on which the conclusions of the paper rely must be either included in your submission or deposited in publicly available repositories (where available and ethically appropriate), referencing such data using a unique identifier in the references and in the "Availability of Data and Materials" section of your manuscript.<br><br>Have you have met the above requirement as detailed in our Minimum Standards Reporting Checklist?	Yes

Powered by Editorial Manager® and ProduXion Manager® from Aries Systems Corporation

Manuscript
Click here to download Manuscript Final Manucript_Karmakar et al _29th July 2018.docx

De novo genome assembly of Indian Blue Peacock (*Pavo cristatus*), from Oxford Nanopore and Illumina sequencing reads

Authors: Ruby Dhar[1,], Ashikh Seethy[1], Karthikeyan Pethusamy[1,] Vishwajeet Rohil[2], Sunil Singh[1], Kakali Purkayastha[2], Sandeep Goswami[1], Rakesh Singh[3], Indrani Mukherjee[1], Ankita Raj[1], Tryambak Srivastava[1], Sovon Acharya[1], Balaji Rajashekhar[4,]* and Subhradip Karmakar[1,]*

Affiliation: [1]Department of Biochemistry, AIIMS, New Delhi, India. [2] Vallabhbhai Patel Chest Institute (VPCI), New Delhi, India. [3]Kanpur Zoo, Kanpur, India.[4] Institute of Computer Science, University of Tartu, 50409 Tartu, Estonia.

***Corresponding Authors email:** subhradip.k@aiims.edu, balaji@ut.ee

Running Title: De novo Genome Assembly of the Peacock Bird

Key words: Peacock, *Pavo cristatus*, Indian National Bird, Genome Assembly, Oxford Nanopore.

Abstract

Background

Pavo cristatus the Indian blue peacock are geographically found distributed in natural habitats of South Asia. Peacock has been described among the bird species as one of the most elegant, majestic and beautiful bird (Fig. 1). Since prehistoric times they have been described or depicted in Indian culture and has been adopted as the national bird of India. Its length varies from 92-125 centimeter (without train), weighing about 4-8 Kilograms and lives up to 20 years in the wild. The avian species have been very important in the fields of phylogenetics, developmental studies, sexual reproduction and speciation. Avian genomics have contributed immensely towards understanding the vertebrate genome evolution. Here we present the first draft genome sequence of P. *cristatus*, yet another important bird species to further add values and gain insight into avian genomics.

Findings

For the first time in avian genomics, long reads using Oxford Nanopore technology have been used for the whole genome assembly. We sequenced different DNA insert size libraries from Illumina and long read Nanopore technologies from the peacock DNA. We performed *de novo* genome assembly by integrating the reads from Illumina short insert, long insert, multiple mate-pair reads along with Nanopore long reads using multiple genome improvement tools. A draft of the peacock genome of about 0.915 Gigabases (Gb) with a N50 of 0.23 Megabases (Mb) was assembled. Annotations with other avian species, protein families, KEGG were performed for functional understanding by insilico approaches. Proteins were compared against Chicken, Turkey and Human to obtain evolutionary similarities and uniqueness of the *Pavo* species.

Conclusions

Our most important findings from the genome sequence of *P. cristatus* is to decipher the different gene families and to understand their role in body pattern development and other features that truly makes this bird unique. The genome sequence also gives insights on its genetic lineage and evolution with relation to other avian members. Several hypothesis and theories have been discussed with respect to sexual selection; now with the understanding of the genome sequence, some of these evolutionary theories will be better understood. The genome will also support future studies on population genetics and breeding for species conservation as well as in understanding its evolutionary ecology and sexual dimorphism. The comparative genomics with other avian species and specifically with *Gallus gallus* (Chicken) and *Meleagris gallopavo* (Turkey) have shown insights into the gene families and their conserved domains. *Pavo* proteins were also compared with human to understand the functional components that were conserved after the speciation split.

Introduction

Pavo cristatus commonly known as the blue Indian peacock obtained the status of National Bird of India in 1963. Peacocks have been distributed widely in Asian countries. The bird is very popular as it symbolizes beauty, love, grace and pride (Gadagkar, R; Kushwaha, et al.). It has been referred in ancient literatures of India and has been found closely associated with the life and culture of the peoples from South East Asia and particularly India (Kadgaonkar, SB). Peacocks are native to South Asia but have been introduced into many other countries usually as exhibits in park, zoos and also large number of aviculturists raise and breed these species as pets (Brickle, N; Jackson, C).

International Chicken Genome Sequencing Consortium sequenced the *Gallus gallus* genome in 2004, and this laid the foundation for study of avian genomics. A decade later, the avian genome consortium assembled 48 genomes of wide variety of avian species to understand the evolutionary relationships of modern birds (Zhang, G., et al.). Raw sequencing data for each species were generated at from coverage of 6X for zebra finch to a maximum 160X for budgerigar. The genome size varied from 1.04 to 1.26 Gb (http://avian.genomics.cn/en/). The studies on different bird species have provided a new perspective on vertebrate genome evolution. These genomes have also helped in improving the annotation of mammalian genomes. There are several distinguishing as well as unique features between chicken and human genome including genome size which is of one third of humans, conserved synteny blocks complete absence of active short interspersed nucleotide elements (SINE) to mention a few (International Chicken Genome sequencing consortium).

Despite the wealth of information from avian genomes sequencing projects, it is very important to genome sequence other new species to add value into aves and vertebrate

genomics. For the first time in bird genomics, Oxford Nanopore technology has been used to sequence a bird genome in this present study. The long read chemistry will help in better genome assembly of the TEs and repeat rich. The peacock genome will aid in understanding about the uniqueness of this birds genome in comparison to other bird species. Comparative genomes will help in understanding the development of this species, sexual selection and its evolutionary relationships with other birds. The characterization of the genes involved in sex determination could provide relevant information for the selective breeding of the peafowl populations. We have unraveled some of the genomic signatures and thus have reported unique gene pools of this bird by performing comparative genomics. Further different data types will improve the assembly and gene/genome characterization will help to address the sexual selection theory and key answers relevant to the evolution of this bird.

Materials and methods

Sample collection and extraction of DNA

The whole blood of male peacock was collected from Kanpur zoo, India after obtaining the necessary ethical and institutional approval. 20µl of Proteinase K (PK) solution was taken into a 1.5ml micro centrifuge tube. 200µl of blood was added and briefly mixed. 200µl of cell lysis buffer was added to the tube, mixed by vortexing for 10seconds; incubated at 56°C for 10minutes. ReliaPrep™ Binding Column was placed into an empty collection tube. 250µl of Binding Buffer (BBA) was added, capped the tube, and mixed by vortexing for 10 seconds with a vortex mixer. Contents of the tube were added to the ReliaPrep™ binding column, capped and placed in a refrigerated micro centrifuge. These were then centrifuged for 1 minute at maximum speed and flow through was discarded. Binding column was placed into a fresh collection tube. 500µl of column wash solution was added to the column and

centrifuged for 3 minutes at maximum speed; Flow through was again discarded. Column washing is repeated thrice. Columns were then placed in a nuclease free clean 1.5ml micro centrifuge tube. 100 µl of Nuclease-Free Water was then added to the column and centrifuged for an additional 1 minute at maximum speed. Column was discarded and the elute was saved. The concentration and purity of the extracted DNA was evaluated using Nanodrop Spectrophotometer (Thermo Scientific) and Qubit flurometer and integrity was checked on a 0.8% agarose gel. The DNA sample was aliquoted for library preparation on two different platforms: Illumina HiSeq4000 and Oxford Nanopore Technologies (ONT).

HiSeq Paired-End library preparation and sequencing

Whole genome sequencing (WGS) libraries were prepared with Illumina-compatible NEXTflex DNA sequencing kit (BIOO Scientific, Austin, Texas, U.S.A.). Briefly, approx. 1 µg of genomic DNA was sheared using Covaris S2 sonicator (Covaris, Woburn, Massachusetts, USA) to generate approx. fragment size distribution from 300 to 600 basepair (bp). The fragment size distribution was checked on Agilent 2200 Tape Station with D1000 DNA screen tapes and reagents (Agilent Technologies, Palo Alto, CA, USA) and subsequently purified using HighPrep magnetic beads (Magbio Genomics Inc, USA). The purified fragments were end-repaired, adenylated and ligated to Illumina multiplex barcode adaptors as per NEXTflex DNA sequencing kit protocol (BIOO Scientific, Austin, Texas, USA).

The adapter-ligated DNA was purified with HighPrep beads (MagBio Genomics, Inc, Gaithersburg, Maryland, USA) and then size selected on 2% low melting agarose gel and cleaned using MinElute column (QIAGEN). The resultant fragments were amplified for 10 cycles of PCR using Illumina-compatible primers provided in the NEXTFlex DNA

sequencing kit. The final PCR product (sequencing library) was purified with HighPrep beads, followed by library quality control check. The Illumina-compatible sequencing library was initially quantified by Qubit fluorometer (Thermo Fisher Scientific, MA, USA) and its fragment size distribution was analyzed on Agilent TapeStation. Finally, the sequencing library was accurately quantified by quantitative PCR using Kapa Library Quantification Kit (Kapa Biosystems, Wilmington, MA, USA). The qPCR-quantified library was subjected to sequencing on an Illumina sequencer for 150 bp paired-end chemistry.

The Illumina-compatible sequencing library for the samples has a fragment size range between 275 to 425 bp for Paired-End Short Insert (PE-SI) and 350 bp to 650bp for Paired-End Long Insert (PE-LI). As the combined adapter size is approximately 120bp, the effective user-defined insert size is 155 to 305 bp and 230 to 530 bp for PE-SI and PE-LI respectively. Libraries were sequencing in Illumina HiSeq platform with 150*2 chemistry. The short reads of Paired-End Short Insert (PE-SI), Paired-End Long Insert (PE-LI) and Mate-Pair (MP) from Illumina HiSeq platform.

Mate-Pair library preparation and sequencing

Mate Pair sequencing library was prepared with Illumina-compatible Nextera Mate Pair Sample Preparation Kit (Illumina Inc., Austin, TX, U.S.A.). Briefly, approx. 4 ug of genomic DNA was simultaneously fragmented and tagged with Mate Pair adapters in a Transposon based Tagmentation step. Tagmented DNA was then purified using AMPure XP Magnetic beads (Beckman Coulter Life Sciences, Indianapolis, IN, U.S.A.) followed by Strand Displacement to fill gaps in the Tagmented DNA. Strand displaced DNA was further purified with AMPure XP beads before size-selecting the 3-5 Kilobases (Kb), 5-7 Kb & 7-10 Kb

fragments on low melting agarose gel. The fragments were circularized in an overnight blunt-end intra-molecular ligation step, which will result in circularization of DNA with the insert flanked mate pair adapter junction.

The circularized DNA was sheared using Covaris S220 sonicator (Covaris, Woburn, Massachusetts, USA) to generate approx. fragment size distribution from 300 bp to 1000 bp. The sheared DNA was purified to collect the Mate pair junction positive fragments using Dynabeads M-280 Streptavidin Magnetic beads (Thermo Fisher Scientific, Waltham, MA, U.S.A.). The purified fragments were end-repaired, adenylated and ligated to Illumina multiplex barcode adaptors as per Nextera Mate Pair Sample Preparation Kit protocol.

The adapter-ligated DNA was then amplified for 15 cycles of PCR using Illumina-compatible primers. The final PCR product (sequencing library) was purified with AMPure XP beads, followed by library quality control check. The Illumina compatible sequencing library was initially quantified by Qubit fluorometer (Thermo Fisher Scientific, MA, USA) and its fragment size distribution was analyzed on Agilent TapeStation. Finally, the sequencing library was accurately quantified by quantitative PCR using Kapa Library Quantification Kit (Kapa Biosystems, Wilmington, MA, USA). The qPCR quantified libraries were pooled in equimolar amounts to create a final multiplexed library pool for sequencing on an Illumina sequencer.

Nanopore MinION library preparation and sequencing

Genomic DNA (1.5µg) was end-repaired (NEBnext ultra II end repair kit, New England Biolabs, MA, USA), cleaned up with 1x AmPure beads (Beckmann Coulter,USA). Adapter ligation were performed for 20 minutes using NEB blunt/ TA ligase (New England Biolabs,

MA, USA). Library mix were cleaned up using 0.4X AmPure beads (Beckmann Coulter, USA) and eluted in 25 µl of elution buffer. Eluted Library were used for sequencing. Whole genome library were prepared by using ligation sequencing kit SQK-LSK108-Oxford Nanopore Technology (ONT) from Oxford Nanopore Technology. Sequencing were performed on MinION Mk1b (Oxford Nanopore Technologies, Oxford, UK) using SpotON flow cell (FLO-MIN106) in a 48hr sequencing protocol on MinKNOW 1.1.20 from ONT.

Illumina raw data QC and processing

The Illumina reads were de-multiplexed using Illumina bcl2fastq. The Illumina generated raw data for genomic libraries was quality checked using FastQC (Andrews, S., 2010). The paired-end Illumina reads were processed for clipping the adapter and low-quality bases using customized script which retains minimum 70% bases/reads with Phred score (Q≥30 in each base position) with a read length of 50 bp. The MP libraries were trimmed for adapter and low-quality base trimming from the 3'-end using PLATANUS internal trimmer (Kajitani, R., et al.).

Nanopore reads base calling and processing

Base calling was performed using Metrichor V.2.43.1 is a cloud based analysis tool provided by Oxford Nanopore Technology software suite. The Nanopore reads were processed using Poretools (Loman, NJ., et al.) for converting fast5 files to fasta format. For further quantification and analysis the 2D reads or 1D high quality reads were selected for further assembly.

***De novo* genome assembly and genome size estimation**

The quality checked Nanopore reads were error-corrected using Illumina PE reads. For error-correction the Illumina PE-reads were aligned to the Nanopore reads by using BWA aligner (Li, H., et al.). The paired-end reads were assembled using Abyss (Birol. I., et al.) followed by contig extension using Nanopore reads using SSPACE-LongRead (Boetzer, M., et al.). Super scaffolding of the assembled scaffold was performed using SSPACE (Boetzer, M., et al.) and PLATANUS using the Nanopore and Matepair data. Final draft genome resulted after gap closure by GAPCLOSER and PLATANUS gap_close tool using Illumina data. The genome size was estimated using a k-mer distribution plot using JELLYFISH (Marcais, G., et al.). The repetitive elements were identified in the final assembled draft genome using Repeat Masker tool. The draft genome was hardmasked by using reference genomic repeats of *G. gallus*. The assembly and annotation workflow overview has been represented as Figure 2.

Simple sequence repeats prediction

Final assembled scaffolds were analysed for Simple Sequence Repeats (SSR) identification. SSRs like the di, tri, tetra, penta and hexa-nucleotide repeats in the genome were obtained using MISA (Version 1.0.0).

Genome prediction and annotation

Gene models was predicted on a hard masked draft genome, where the repetitive elements in the draft genome were masked using genomic repeats of *G. gallus* with Repeatmasker tool and further genes were predicted using AUGUSTUS with *G. gallus* as a reference model. The predicted proteins were annotated by using BLASTP (Altshul, S., et al.) against all Avian sequences downloaded from UniProt Protein Database.

Pathway Analysis of the draft genome

The predicted proteins were searched against the KEGG-KAAS server (Moriya, Y., et al.) for pathway analysis. *G. gallus* (chicken), *Meleagris gallopavo* (turkey), *Taeniopygia guttata* (zebra finch), *Falco peregrinus* (peregrine falcon) were used as reference organism for pathway identification. The EuKaryotic Orthologous Groups (KOGs) were predicted using homology based approach.

Mitochondrial genome assembly and annotation

The generated scaffolds from the draft assembly were aligned against the *P. cristatus* mitochondria genome and the mapped reads were filtered and stitched using ABACUS software using the same reference (Zhou, TC., et al.). Further gap closure were performed with 3-7kb MP reads to generate an complete assembled mitochondrial genome. MITOS (Bernt, M., et al.) was used for gene annotation. Circular plot generated using GenomeVx (http://wolfe.ucd.ie/GenomeVx/) representing the localization of the gene in the assembled mitochondrial genome.

Phylogenetic tree construction

The assembled Peacock mitochondrial genome was searched against 695 avian mitochondrial genomes downloaded from NCBI. Based on the Blast-N homology results (with query coverage> 100, subject coverage > 95, % identity >85 and with 1% gaps allowed in the sequences). 51 mitochondrial genome sequences along with our assembled mitochondrial genome were filtered. Multiple sequence alignment with default parameters were performed using MUSCLE global sequence aligner. Phylogenetic trees were constructed using IQ-TREE version 1.5.6 (www.iqtree.org). The parameters used for phylogenetic tree construction were ultrafast boostrap (UFBoot, using the –bb option of 1000 replicates), and a standard substitution model (-m MFP) was given for tree generation. The generated trees

from IQ-TREE tool were visualized using Figtree (http://tree.bio.ed.ac.uk/software/figtree/) and the Brach-support values were recorded from the output ".treefile". The trees were modified for better visualization under Trees section increasing order nodes were applied.

Protein domain analysis

Predicted proteins from Peacock, Chicken and Turkey with sequence length greater than 100 amino acids were considered for protein domain analysis. All the protein sequences from each organism were searched against Pfam-A database using Pfam scan for protein domain identification.

Avian protein families

The protein sequences of 48 avian genomes was downloaded from the link http://avian.genomics.cn/en/jsp/database.shtml apart from the predicted proteins of the draft genome. Sequences greater than 100 amino acids from all the avian genomes were filtered and concatenated to a single fasta file. These sequences were clustered using CD-HIT (Fu, L., et al.) with 90% alignment coverage for the shorter sequence with a length difference cutoff of 0.9. The single copy ortholog gene family present across all organisms and genes unique to peacock were filtered and annotated.

Genome conservation analysis

The assembled draft genome was aligned against the *G. gallus* genome using Chromosomer tool. Draft chromosomes were constructed based on alignment between fragments (scaffolds) using a reference genome. These reordered assembled genome was aligned against the Chicken genome using LAST aligner with NEAR (finding short-and-strong (near-identical) similarities.) parameter allowing for substitution and gap frequencies leading to the

identification of orthologs. These query mapped regions were filtered with greater than 1% of the maximum length for visualization using Circos.

Results

DNA Sequencing data

Five libraries of 150 bp paired-end from Illumina HiSeq technology were generated. The short-insert reads of 489,114,747 accounted to genome coverage of 146.7X and long-insert reads of 302,884,819 sequences was about 90.9X coverage with a total coverage of 236X. Sequencing of three mate-pairs of 3-5Kb, 5-7Kb of and 7-10Kb yielded 72,915,033, 47,440,144 and 36,464,628 reads respectively with an approximate coverage of 21.9, 14.2 and 10.9 respectively, with a total of 156 million reads of 47X coverage. Oxford Nanopore technology was used to generate 366,323 long reads having of 2,398,560,283 bp with coverage of 2.3X. The complete sequencing was generated to a depth of ~287X from Illumina and Nanopore platforms. The coverage was based on assuming the peacock genome size of about 1 Gb (Table S1).

Genome assembly

The assembly was performed on Illumina reads with Abyss *de novo* assembler that resulted in ~932 Mb (mega base) of genome with an N50 of 1639 bp. The extension of the contigs were performed with Nanopore reads which generated scaffolds with N50 of 14,748 bp. Super scaffolding of the assembled scaffold was performed using SSPACE and PLATANUS with MP libraries that generated ~916 Mb genome with the N50 value of 168,140bp. The final gap closer was executed using GAPCLOSER program with MP and PE-LI libraries which generated a draft genome of 1.02 GB (giga base). The draft genome assembly of *Pavo*

cristatus consists of 179,346bp scaffolds, with a N50 of 189,886bp with 37 scaffolds having sequence length >=1Mbp. Contigs above 5000 bp have covered a genome of ~0.915 Mb with N50 0.23 Mb. In the assembled genome there were ~0.4% of non ATGC characters (Table 1).

Complete mitochondrial genome of 16699 bp was obatained. Total of 22 tRNA, tow rRNA and 13 protein coding genes were identified in the assembled genome (Fig. 3a). A 100% similarity was observed with the preiously published Peacocks mitochondrial genome (Fig. S1a and S1b).

Transposable Elements (TE)

In the bird genome a total of 75,315,566 bp (7.3% of the genome) was predicted to have 5.5% of Retroelements (with SINEs 0.08% and LINEs 4.71%), 6.25 % total interspersed repeats with 0.84 % simple repeats and 0.21% low complexity regions. The DNA transposons identified in the genome was 0.71% (Table S2).

Protein coding gene annotation

A total of 23,153 proteins were predicted in the assembled draft genome using AUGUSTUS. Among them 95% predicted genes were annotated against the other Aves proteins. The 21,854 annotated proteins showed top similarity to species *Gallus gallus* (Chicken) with 11,398 proteins, *Meleagris gallopavo* (Common turkey) with 4059 proteins, *Amazona aestiva* (Blue-fronted Amazon parrot) with 1352 proteins and *Anas platyrhynchos* (Mallard) (*Anas boschas*) with 849 proteins. Thirteen species had about 100 to 400 annotated proteins. The remaining proteins were in the range of 1 to <100 proteins in about 62 species. From the

annotations a total of 13,161 proteins showed similarity to uncharacterized protein annotation and some of the over represented proteins were Tyrosine-protein kinase, Sulfotransferase, Phosphoinositide phospholipase C, Tetraspanin, Phospholipid-transporting ATPase, Olfactory receptor, Polypeptide N-acetylgalactosaminyltransferase, Transporter, Keratin, Hexosyltransferase, Protein Wnt, Kinesin-like protein, Gap junction protein, Claudin, POU domain protein, Sodium/hydrogen exchanger, Phospholipid-transporting ATPase, Histone-lysine N-methyltransferase and others (Table S3). The gene ontology annotations showed to have Gene Ontology (GO) descriptions for 18,295 proteins. Among them, 14,490 proteins have Molecular Function; 11,679 have Biological Process and 13,736 proteins have Cellular Component as functional categories.

About 17.7% of proteins were found to have pathway information against the KEGG database (Table S4). Some of the overrepresented annotations were Kinases like MAPK (mitogen-activated protein kinase); JNK (c-Jun N-terminal kinase); RAF (RAF proto-oncogene serine/threonine-protein kinase); AKT (RAC serine/threonine-protein kinase); protein kinases and different GTPases.

Proteins searched against the KOG annotations showed a total of 20,937 proteins having annotations. Among them, the most abundant annotations include Zn-finger, transmembrane receptor, ubiquitin ligase, Leucine rich repeat, Cadherin repeats, Serine/threonine protein kinase, Collagens, Ankryin repeat, Fibrillins, Voltage-gated Ca2+ channels and Hormone receptors (Table S5). The peacock proteins when searched against the human proteins showed expansions in ontologies for cell morphogenesis, neuronal projection and development and GTPases (Table S10 and Fig. S4).

Simple sequence repeats

A total of 399,493 SSRs were identified from the peacock genome assembly. The largest fraction of SSRs identified were mononucleotide (60.04%), followed by tetra nucleotide (26%), di nucleotide (8.51%), tri nucleotide (4.31%), penta nucleotide (1.03%) and finally hexa nucleotide (0.13%). Among the SSRs identified, A (49.2%) and T (44.9%) accounted for 94.1% of the mono-nucleotide repeats. AT (23.8%), TA (16.5%), TG(13.7%), AC(10.6%) and CA (10.32%) accounted for 75% of the di-nucleotide repeats. while TTG (9.9%), AAT (9.6%), AAC (9.4%), TTA (7.1%), ATT (4.5%), TAA (3.5%), CAA (3.1%) and GGA (2.69%) accounted for 49.7% of the tri-nucleotide repeats (Table S6).

Avian protein family analysis

A total of 748,544 protein sequences from 49 avian species have 653,497 protein sequences of length above 100 amino acids (Table S7A). A total of 240,853 gene clusters were generated of which 41 gene clusters had single copy orthologs in all avian species (Table S7B and Table S7C). With the above stringent cutoff we observed 15,913 gene clusters were unique to peacock species.

Phylogeny and Genome comparisons

The phylogeny of 51 mitochondrial genome sequences along with peacock genome showed a clade consisting of *Pavo* species and *Gallus* (red junglefowl, Sri Lankan junglefowl, grey junglefowl, grey junglefowl), Bambusicola (Mountain Bamboo-partridge, Chinese bamboo partridge) and Francolinus (Chinese francolin). It can be observed many species were distributed in two different clades where 34 species were found in one clade and five species in other clade. Some of the endemic or native bird species like *Arborophila ardens*, *Acryllium vulturinum* and *Numida meleagris* were found as clear outgroup of species in this study (Fig. 3b).

Predicted proteins from Peacock, Chicken and Turkey were searched for protein domain analysis. 81% of the Pfams were common among the three species. About 94%, 98.4% and 99.7% predicted Pfam domains were identified in Peacock, Chicken and Turkey respectively. There were 255, 69 and 14 Pfam domains found to be unique in the species mentioned above respectively (Fig. 4).

The assembled Peacock genome was reordered for pseudo chromosomes generation against the simple repeat masked Chicken genome (1.21 GB, Warren, WC., et al.) using Chromosomer which generated a overall reordered Peacock genome of about 597MB. The right side of the image represents the reference genome and left side of the image represents the Peacock genome (Fig. 5).

Conclusions

Third generation sequencing in avian genomics where long reads having the substantiality to improve genome assembly will benefit understanding the organisms in the structurally complex regions having repeat elements and isoforms in the genome (Goodwin, S., et al.). Using a combination of short reads of different insert sizes as well as mate pair reads generated from Illumina technology along with long reads from Oxford Nanopore, we obtained an improved assembly and a draft genome of the Indian Blue Peacock (*Pavo cristatus*). In comparison to other avian genomes (Zhang, G., et al.), the current 290X sequencing depth obtained from our study is one of the highest. With a N50 of 0.23Mb, we presented here a reasonably reliable draft genome for the peacock species. The inclusion of Nanopore reads 366,323 for scaffolding followed by subsequent gap-closing using Illumina data led to a 26.2% reduction in the number of scaffolds and a 50.65% and 115% increase in the scaffold and contig N50, respectively. With only 2.3X of long reads, a significant improvement in the assembly was observed. On the contrary, the assembly contained less

than 0.4% of unknown nucleotides, which is very low for a draft assembly. With the addition of more long reads along with transcriptomic sequencing along with scaffolding and/or gap closure tools, further improvement in the assembly can be achieved.

Peacock seems to defy the Darwinian laws of natural selection. These concern were raised by no other than Darwin himself. Hence, he proposed the theory of the sexual selection where the female can choose for a male with a certain phenotypic feature such as brilliant color or a long tail (Burgess, S.). Peacock’s brilliantly colored long tail feathers seems to evolve at the cost of finding its female partner thereby contributing its beneficial genes, even at the cost of making itself venerable to predators. A female peafowl in turn tends to choose the mate with the largest and decorated plumage, which indirectly reflects its healthiness and capacity to wade off potential competitors thereby selecting the most suitable male. Peacocks beautiful feathers with it all its artistry surely provides it with an advantage to impress the females (Dakin, R., et al.). Understanding the formation of beautiful feathers from the genomic context will help in resolving several evolutionary theories on sexual selection that have been discussed on this species.

Pigment particles are embedded into the newly grown peacock feathers during the molting season which seems to absorb light of selective wavelength there by imparting to the color of the plumage (Mercedes Foster, Rennee Riedler, et al.) Pigment morphogenesis in *Gallus* has been reported by a process of melanoblasts migration and colonization into feather bud where they differentiate to produce the pigment, melanin (Kelsh, RN., et al.). The molecular mechanisms that control the pigment cell migration have been narrowed down to proteins Kit1 and FGFs which maintain the melanoblasts migration to feathers. Kit and FGF proteins have also been identified in our current study (Table S3). Understanding of these proteins in the patterned formation will help decode the pigment pattern morphology in peacocks. These pigmented patterns play a role in communication, choice of mate and in some species it can help in camouflage (Burgess, S)

It has been observed that the variations in the genome size among bird species are very low (Table S8). The genome complexities of a species are influenced by the Transposable elements (TE) that are believed to play a crucial role (Kapsuta, A., et al.). The long read sequences have significantly helped in resolving the TEs in genome quality and assembly. Peacock genome comparisons with Turkey and Chicken have showed closeness to the Chicken species. The mitochondrial phylogeny also revealed similar findings. Homology searches have shown several important gene family expansions such as Kinases, Zn finger proteins, GTPases and others. Their roles in biology, development and evolution of the Peacocks have to be further explored.

To summarize, we have assembled the *Pavo* genome using Illumina and Oxford nanopore technology. The genome information can be valued and explored by avian enthusiasts to further understand about this bird. Though not critically endangered yet, in India, peafowl population is surely at a decline in the wild due to massive deforestation and habitat loss. Thus is further compounded by increased poaching for meat and feathers. Our genome sequencing initiative of *Pavo cristatus* is not just only from a conservational viewpoint, but also to preserve a heritage associated with this bird that runs through centuries and that bears a strong attachment to the national psyche.

Availability of supporting data

Supplementary data contains, read statistics, annotation, repeats identification, orthology analysis, mitochondria assembly and annotation. Figures, Gene ontology. DNA and library preparation protocols.

Raw Data in SRA

Raw reads (Illumina and Nanopore) are available in the Sequence Read Archive (SRA), and the Whole Genome Shotgun project has been deposited at GenBank under SRA Submission ID: SUB3108024, Bioproject: PRJNA413288 and Biosamples SAMN07739105 : SKPea2016_SI, SAMN07739104 : SKPea2016_LI, SAMN07739101 : FPL_3_5KB, SAMN07739102 : FPL_5_7KB, SAMN07739103 : FPL_7_10KB and SAMN07739107 : FPL_Nano.

Competing interests

The author(s) declare that they have no competing interests.

Authors contributions

RD, AS, KP performed wet lab experiments; RD designed work plan, experiments and logistics; SS, VR, KP SG IM and AR assisted with the work; RS provided samples from bird; BR, SK performed data analysis and interpretation; SK, BR, RD drafted the manuscript and SK overseen the whole project.

Acknowledgements

Department of Biochemistry, AIIMS, New Delhi for providing space and infrastructure to carry on the work. RD for providing partial funding. Kritika Tripathi for inputs with manuscript editing. Raja and Sudha from Genotypic Technology Pvt. Ltd for providing sequencing services and also Deepti, Shalini, Nagu, Abhi and Manish. BR acknowledges IUT 34-4.

Tables

Table 1. *De novo* assembly statistics of the Peacock genome.

Description	Contigs	Nanopore Scaffold	Super Scaffolds	GapClosed	>1000 Kb	>5000 Kb
Contigs	685241	281272	179346	179346	34181	15026
Maximum Length	49159	251510	2390121	2488982	2488982	2488982
Minimum Length	300	5	265	265	1000	5000
Average Length	1360	3250	5111	5729	-	-
Total Length	932162464	914363908	916720956	1027551907	954483822	915373606
Length >= 100 bp	685241	281271	179346	179346	34181	15026
Length >= 200 bp	685241	281271	179346	179346	34181	15026
Length >= 500 bp	616120	186433	93727	93727	34181	15026
Length >= 1 Kbp	363428	104479	34168	34181	34181	15026
Length >= 10 Kbp	1591	24748	9249	10311	10311	10311
Length >= 1 Mbp	0	0	27	37	37	37
Non-ATGC #	350325	42696911	49169831	4034372	4032567	3978757
Non-ATGC %	0.038	4.67	5.364	0.393	0.422	0.435
N50 value	1639	14748	168140	189886	218023	232312

Figure legend

Figure 1. The beautiful and charismatic photo of Indian blue peacock (*Pavo cristatus)* bird.

Figure 2. Detailed workflow for *de novo* genome assembly and annotation.

Figure 3a. Circular representation of Peacock mitochondrial genome with genes predicted.

Figure 3b. Phylogenetic tree generated from mitochondrial genome from 52 different avian species.

Figure 4. Circular image of the assembled peacock genome aligned against the *G. gallus* genome using Chromosomer tool. Draft chromosomes were generated by similarity between scaffolds which were arranged on the reference chicken genome. Circos was used for visualization.

Figure 5. Venn diagram showing common and unique Protein family domains (Pfam) between Peacock, Chicken and Turkey proteins.

References:

Gadagkar, R., 2003. Is the peacock merely beautiful or also honest?. Current Science, 85(7), pp.1012-1020.

Kushwaha, S., and Kumar, A. 2016. A Review on Indian Peafowl (*Pavo cristatus*) Linnaeus, 1758. Journal of Wildlife and Research, 4, 42-59.

Kadgaonkar, Shivendra B. 1993. The peacock in ancient Indian art and literature. Bulletin of the Deccan College Research Institute, vol. 53, pp. 95–115. JSTOR, www.jstor.org/stable/42936434.

Brickle, N. 2002. Habitat use, predicted distribution and conservation of green peafowl (*Pavo muticus*) in Dak Lak Province, Vietnam. Biological Conservation, 105: 189-197.

Jackson, C. 2006. Peacock. London: Reaktion Books Ltd.

Zhang, G., Jarvis, E. D., and Gilbert, M. T. P. 2014. A flock of genomes. Science 346, 1308–1309.

International Chicken Genome Sequencing Consortium. 2004. Sequence and comparative analysis of the chicken genome provide unique perspectives on vertebrate evolution. Nature, 432(7018), 695.

Kajitani, R., Toshimoto, K., Noguchi, H., Toyoda, A., Ogura, Y., Okuno, M., Yabana, M., Harada, M., Nagayasu, E., Maruyama, H. and Kohara, Y., 2014. Efficient de novo assembly of highly heterozygous genomes from whole-genome shotgun short reads. Genome research, 24(8), pp.1384-1395.

Loman, N. J. and Quinlan, A. R. 2014. Poretools: a toolkit for analyzing nanopore sequence data. Bioinformatics, 30(23), 3399-3401.

Li H. and Durbin R. 2009. Fast and accurate short read alignment with Burrows-Wheeler Transform. Bioinformatics, 25:1754-60.

Birol, I., Jackman, S.D., Nielsen, C.B., Qian, J.Q., Varhol, R., Stazyk, G., Morin, R.D., Zhao, Y., Hirst, M., Schein, J.E. and Horsman, D.E., 2009. De novo transcriptome assembly with ABySS. Bioinformatics, 25(21), pp.2872-2877.

Boetzer, Marten, and Walter Pirovano. 2014. SSPACE-LongRead: scaffolding bacterial draft genomes using long read sequence information. BMC bioinformatics 15.1: 211

Boetzer, M., Henkel, C.V., Jansen, H.J., Butler, D. and Pirovano, W., 2010. Scaffolding pre-assembled contigs using SSPACE. Bioinformatics, 27(4), pp.578-579.

Marcais, G and Kingsford, C. 2011. A fast, lock-free approach for efficient parallel counting of occurrences of k-mers. Bioinformatics 27(6): 764-770.

Altschul, S.F., Gish, W., Miller, W., Myers, E.W. and Lipman, D.J., 1990. Basic local alignment search tool. Journal of molecular biology, 215(3), pp.403-410.

Moriya, Y., Itoh, M., Okuda, S., Yoshizawa, A.C. and Kanehisa, M., 2007. KAAS: an automatic genome annotation and pathway reconstruction server. Nucleic acids research, 35(suppl_2), pp.W182-W185.

Zhou, T.C., Sha, T., Irwin, D.M. and Zhang, Y.P., 2015. Complete mitochondrial genome of the Indian peafowl (*Pavo cristatus*), with phylogenetic analysis in phasianidae. Mitochondrial DNA, 26(6), pp.912-913.

Bernt, M., Donath, A., Jühling, F., Externbrink, F., Florentz, C., Fritzsch, G., Pütz, J., Middendorf, M. and Stadler, P.F., 2013. MITOS: improved de novo metazoan mitochondrial genome annotation. Molecular phylogenetics and evolution, 69(2), pp.313-319.

Fu, L., Niu, B., Zhu, Z., Wu, S. and Li, W., 2012. CD-HIT: accelerated for clustering the next-generation sequencing data. Bioinformatics, 28(23), pp.3150-3152.

Warren, W.C., Hillier, L.W., Tomlinson, C., Minx, P., Kremitzki, M., Graves, T., Markovic, C., Bouk, N., Pruitt, K.D., Thibaud-Nissen, F. and Schneider, V., 2016. A new chicken

genome assembly provides insight into avian genome structure. G3: Genes, Genomes, Genetics, pp.g3-116.

Goodwin, S., McPherson, J.D. and McCombie, W.R., 2016. Coming of age: ten years of next-generation sequencing technologies. Nature Reviews Genetics, 17(6), p.333.

Zhang, G., Li, C., Li, Q., Li, B., Larkin, D.M., Lee, C., Storz, J.F., Antunes, A., Greenwold, M.J., Meredith, R.W. and Ödeen, A., 2014. Comparative genomics reveals insights into avian genome evolution and adaptation. Science, 346(6215), pp.1311-1320.

Burgess, S., 2001. The beauty of the peacock tail and the problems with the theory of sexual selection. Journal of Creation, 15(2), pp.94-102..

Dakin, R. 2008. The role of the visual train ornament in the courtship of peafowl, *Pavo cristatus*. Masters Abstracts International, 47/03: 97.

Mercedes S Foster 1975. The overlap of molting and breeding in some birds. The Condor 77:304-314.

Renee Riedler, Christel Pesme, James Druzik, Molly Gleeson, Ellen Pearlstein The Journal of the American Institute of Conservation 2014

Kelsh, R.N., Harris, M.L., Colanesi, S. and Erickson, C.A., 2009. Stripes and belly-spots—a review of pigment cell morphogenesis in vertebrates. Seminars in cell & developmental biology. Vol. 20, No. 1, pp. 90-104.

Kapusta, A. and Suh, A., 2017. Evolution of bird genomes—a transposon's- eye view. Annals of the New York Academy of Sciences, 1389(1), pp.164-185.

Webservers:

FastQC : http://www.bioinformatics.babraham.ac.uk/projects/fastqc/

AUGUSTUS : http://augustus.gobics.de/

PLATANUS : http://platanus.bio.titech.ac.jp/

ABACUS : http://abacas.sourceforge.net/Manual.html

MISA: http://pgrc.ipk-gatersleben.de/misa/

REPEATMASKER : http://www.repeatmasker.org/

KOGs : https://genome.jgi.doe.gov/portal/help/kogbrowser.jsf

KAAS : http://www.genome.jp/tools/kaas/

MITOS : http://mitos.bioinf.uni-leipzig.de/

MUSCLE : https://www.drive5.com/muscle/

PFAMSCAN : https://www.ebi.ac.uk/seqdb/confluence/display/THD/PfamScan

LAST : http://last.cbrc.jp/

CHROMOSOMER : https://github.com/gtamazian/chromosomer

CIRCOS : http://circos.ca/

Manuscript

Click here to download Manuscript Resubmisison_ Manucript_Karmakar et al _29 th July 2018.docx

De novo genome assembly of Indian Blue Peacock (*Pavo cristatus*), from Oxford Nanopore and Illumina sequencing reads

Authors: Ruby Dhar[1,], Ashikh Seethy[1], Karthikeyan Pethusamy[1,] Vishwajeet Rohil[2], Sunil Singh[1], Kakali Purkayastha[2], Sandeep Goswami[1], Rakesh Singh[3], Indrani Mukherjee[1], Ankita Raj[1], Tryambak Srivastava[1], Sovon Acharya[1], Balaji Rajashekhar[4,]* and Subhradip Karmakar[1,]*

Affiliation: [1]Department of Biochemistry, AIIMS, New Delhi, India. [2] Vallabhbhai Patel Chest Institute (VPCI), New Delhi, India. [3]Kanpur Zoo, Kanpur, India.[4] Institute of Computer Science, University of Tartu, 50409 Tartu, Estonia.

***Corresponding Authors email:** subhradip.k@aiims.edu, balaji@ut.ee

Running Title: De novo Genome Assembly of the Peacock Bird

Key words: Peacock, *Pavo cristatus*, Indian National Bird, Genome Assembly, Oxford Nanopore.

Abstract

Background

Pavo cristatus the Indian blue peacock are geographically found distributed in natural habitats of South Asia. Peacock has been described among the bird species as one of the most elegant, majestic and beautiful bird (Fig. 1). Since prehistoric times they have been described in Indian culture and has been adopted as the national bird of India. Its length varies from 92-125 centimeter (without train), weighing about 4-8 Kilograms and lives up to 20 years in the wild. The avian species have been very important in the fields of phylogenetics, developmental studies, sexual reproduction and speciation. Avian genomics have contributed immensely towards understanding the vertebrate genome evolution. Here we present the first draft genome sequence of P. *cristatus*, yet another important bird species to further add values and gain insight into avian genomics.

Findings

For the first time in avian genomics, long reads using Oxford Nanopore technology have been used for the whole genome assembly. We sequenced different DNA insert size libraries from Illumina and long read Nanopore technologies from the peacock DNA. We performed *de novo* genome assembly by integrating the reads from Illumina short insert, long insert, multiple mate-pair reads along with Nanopore long reads using multiple genome improvement tools. A draft of the peacock genome of about 0.915 Gigabases (Gb) with a N50 of 0.23 Megabases (Mb) was assembled. Annotations with other avian species, protein families, KEGG were performed for functional understanding by insilico approaches. Proteins were compared against Chicken, Turkey and Human to obtain evolutionary similarities and uniqueness of the *Pavo* species.

Conclusions

Our most important findings from the genome sequence of *P. cristatus* is to decipher the different gene families and to understand their role in body pattern development and other

features that truly makes this bird unique. The genome sequence also gives insights on its genetic lineage and evolution with relation to other avian members. Several hypothesis and theories have been discussed with respect to sexual selection; now with the understanding of the genome sequence, some of these evolutionary theories will be better understood. The genome will also support future studies on population genetics and breeding for species conservation as well as in understanding its evolutionary ecology and sexual dimorphism. The comparative genomics with other avian species and specifically with *Gallus gallus* (Chicken) and *Meleagris gallopavo* (Turkey) have shown insights into the gene families and their conserved domains. *Pavo* proteins were also compared with human to understand the functional components that were conserved after the speciation split.

Introduction

Pavo cristatus commonly known as the blue Indian peacock obtained the status of National Bird of India in 1963. Peacocks have been distributed widely in Asian countries. The bird is very popular as it symbolizes beauty, love, grace and pride (Gadagkar, R; Kushwaha, et al.). It has been referred in ancient literatures of India and has been found closely associated with the life and culture of the peoples from South East Asia and particularly India (Kadgaonkar, SB). Peacocks are native to South Asia but have been introduced into many other countries usually as exhibits in park, zoos and also large number of aviculturists raise and breed these species as pets (Brickle, N; Jackson, C).

International Chicken Genome Sequencing Consortium sequenced the *Gallus gallus* genome in 2004, and this laid the foundation for study of avian genomics. A decade later, the avian genome consortium assembled 48 genomes of wide variety of avian species to understand the evolutionary relationships of modern birds (Zhang, G., et al.). Raw sequencing data for each species were generated at from coverage of 6X for zebra finch to a maximum 160X for budgerigar. The genome size varied from 1.04 to 1.26 Gb (http://avian.genomics.cn/en/). The studies on different bird species have provided a new perspective on vertebrate genome evolution. These genomes have also helped in improving the annotation of mammalian genomes. There are several distinguishing as well as unique features between chicken and human genome including genome size which is of one third of humans, conserved synteny blocks complete absence of active short interspersed nucleotide elements (SINE) to mention a few (International Chicken Genome sequencing consortium).

Despite the wealth of information from avian genomes sequencing projects, it is very important to genome sequence other new species to add value into aves and vertebrate genomics. For the

first time in bird genomics, Oxford Nanopore technology has been used to sequence a bird genome in this present study. The long read chemistry will help in better genome assembly of the TEs and repeat rich. The peacock genome will aid in understanding about the uniqueness of this birds genome in comparison to other bird species. Comparative genomes will help in understanding the development of this species, sexual selection and its evolutionary relationships with other birds. The characterization of the genes involved in sex determination could provide relevant information for the selective breeding of the peafowl populations. We have unraveled some of the genomic signatures and thus have reported unique gene pools of this bird by performing comparative genomics. Further different data types will improve the assembly and gene/genome characterization will help to address the sexual selection theory and key answers relevant to the evolution of this bird.

Materials and methods

Sample collection and extraction of DNA

The whole blood of male peacock was collected from Kanpur zoo, India after obtaining the necessary ethical and institutional approval. 20µl of Proteinase K (PK) solution was taken into a 1.5ml micro centrifuge tube. 200µl of blood was added and briefly mixed. 200µl of cell lysis buffer was added to the tube, mixed by vortexing for 10seconds; incubated at 56°C for 10minutes. ReliaPrep™ Binding Column was placed into an empty collection tube. 250µl of Binding Buffer (BBA) was added, capped the tube, and mixed by vortexing for 10 seconds with a vortex mixer. Contents of the tube were added to the ReliaPrep™ binding column, capped and placed in a refrigerated micro centrifuge. These were then centrifuged for 1 minute at maximum speed and flow through was discarded. Binding column was placed into a fresh collection tube. 500µl of column wash solution was added to the column and centrifuged for 3

minutes at maximum speed; Flow through was again discarded. Column washing is repeated thrice. Columns were then placed in a nuclease free clean 1.5ml micro centrifuge tube. 100 µl of Nuclease-Free Water was then added to the column and centrifuged for an additional 1 minute at maximum speed. Column was discarded and the elute was saved. The concentration and purity of the extracted DNA was evaluated using Nanodrop Spectrophotometer (Thermo Scientific) and Qubit flurometer and integrity was checked on a 0.8% agarose gel. The DNA sample was aliquoted for library preparation on two different platforms: Illumina HiSeq4000 and Oxford Nanopore Technologies (ONT).

HiSeq Paired-End library preparation and sequencing

Whole genome sequencing (WGS) libraries were prepared with Illumina-compatible NEXTflex DNA sequencing kit (BIOO Scientific, Austin, Texas, U.S.A.). Briefly, approx. 1 µg of genomic DNA was sheared using Covaris S2 sonicator (Covaris, Woburn, Massachusetts, USA) to generate approx. fragment size distribution from 300 to 600 basepair (bp). The fragment size distribution was checked on Agilent 2200 Tape Station with D1000 DNA screen tapes and reagents (Agilent Technologies, Palo Alto, CA, USA) and subsequently purified using HighPrep magnetic beads (Magbio Genomics Inc, USA). The purified fragments were end-repaired, adenylated and ligated to Illumina multiplex barcode adaptors as per NEXTflex DNA sequencing kit protocol (BIOO Scientific, Austin, Texas, USA).

The adapter-ligated DNA was purified with HighPrep beads (MagBio Genomics, Inc, Gaithersburg, Maryland, USA) and then size selected on 2% low melting agarose gel and cleaned using MinElute column (QIAGEN). The resultant fragments were amplified for 10 cycles of PCR using Illumina-compatible primers provided in the NEXTFlex DNA sequencing kit. The final PCR product (sequencing library) was purified with HighPrep beads, followed

by library quality control check. The Illumina-compatible sequencing library was initially quantified by Qubit fluorometer (Thermo Fisher Scientific, MA, USA) and its fragment size distribution was analyzed on Agilent TapeStation. Finally, the sequencing library was accurately quantified by quantitative PCR using Kapa Library Quantification Kit (Kapa Biosystems, Wilmington, MA, USA). The qPCR-quantified library was subjected to sequencing on an Illumina sequencer for 150 bp paired-end chemistry.

The Illumina-compatible sequencing library for the samples has a fragment size range between 275 to 425 bp for Paired-End Short Insert (PE-SI) and 350 bp to 650bp for Paired-End Long Insert (PE-LI). As the combined adapter size is approximately 120bp, the effective user-defined insert size is 155 to 305 bp and 230 to 530 bp for PE-SI and PE-LI respectively. Libraries were sequencing in Illumina HiSeq platform with 150*2 chemistry. The short reads of Paired-End Short Insert (PE-SI), Paired-End Long Insert (PE-LI) and Mate-Pair (MP) from Illumina HiSeq platform.

Mate-Pair library preparation and sequencing

Mate Pair sequencing library was prepared with Illumina-compatible Nextera Mate Pair Sample Preparation Kit (Illumina Inc., Austin, TX, U.S.A.). Briefly, approx. 4 ug of genomic DNA was simultaneously fragmented and tagged with Mate Pair adapters in a Transposon based Tagmentation step. Tagmented DNA was then purified using AMPure XP Magnetic beads (Beckman Coulter Life Sciences, Indianapolis, IN, U.S.A.) followed by Strand Displacement to fill gaps in the Tagmented DNA. Strand displaced DNA was further purified with AMPure XP beads before size-selecting the 3-5 Kilobases (Kb), 5-7 Kb & 7-10 Kb fragments on low melting agarose gel. The fragments were circularized in an overnight blunt-

end intra-molecular ligation step, which will result in circularization of DNA with the insert flanked mate pair adapter junction.

The circularized DNA was sheared using Covaris S220 sonicator (Covaris, Woburn, Massachusetts, USA) to generate approx. fragment size distribution from 300 bp to 1000 bp. The sheared DNA was purified to collect the Mate pair junction positive fragments using Dynabeads M-280 Streptavidin Magnetic beads (Thermo Fisher Scientific, Waltham, MA, U.S.A.). The purified fragments were end-repaired, adenylated and ligated to Illumina multiplex barcode adaptors as per Nextera Mate Pair Sample Preparation Kit protocol.

The adapter-ligated DNA was then amplified for 15 cycles of PCR using Illumina-compatible primers. The final PCR product (sequencing library) was purified with AMPure XP beads, followed by library quality control check. The Illumina compatible sequencing library was initially quantified by Qubit fluorometer (Thermo Fisher Scientific, MA, USA) and its fragment size distribution was analyzed on Agilent TapeStation. Finally, the sequencing library was accurately quantified by quantitative PCR using Kapa Library Quantification Kit (Kapa Biosystems, Wilmington, MA, USA). The qPCR quantified libraries were pooled in equimolar amounts to create a final multiplexed library pool for sequencing on an Illumina sequencer.

Nanopore MinION library preparation and sequencing

Genomic DNA (1.5µg) was end-repaired (NEBnext ultra II end repair kit, New England Biolabs, MA, USA), cleaned up with 1x AmPure beads (Beckmann Coulter,USA). Adapter ligation were performed for 20 minutes using NEB blunt/ TA ligase (New England Biolabs, MA, USA). Library mix were cleaned up using 0.4X AmPure beads (Beckmann Coulter, USA) and eluted in 25 µl of elution buffer. Eluted Library were used for sequencing. Whole genome

library were prepared by using ligation sequencing kit SQK-LSK108-Oxford Nanopore Technology (ONT) from Oxford Nanopore Technology. Sequencing were performed on MinION Mk1b (Oxford Nanopore Technologies, Oxford, UK) using SpotON flow cell (FLO-MIN106) in a 48hr sequencing protocol on MinKNOW 1.1.20 from ONT.

Illumina raw data QC and processing

The Illumina reads were de-multiplexed using Illumina bcl2fastq. The Illumina generated raw data for genomic libraries was quality checked using FastQC (Andrews, S., 2010). The paired-end Illumina reads were processed for clipping the adapter and low-quality bases using customized script which retains minimum 70% bases/reads with Phred score (Q≥30 in each base position) with a read length of 50 bp. The MP libraries were trimmed for adapter and low-quality base trimming from the 3'-end using PLATANUS internal trimmer (Kajitani, R., et al.).

Nanopore reads base calling and processing

Base calling was performed using Metrichor V.2.43.1 is a cloud based analysis tool provided by Oxford Nanopore Technology software suite. The Nanopore reads were processed using Poretools (Loman, NJ., et al.) for converting fast5 files to fasta format. For further quantification and analysis the 2D reads or 1D high quality reads were selected for further assembly.

***De novo* genome assembly and genome size estimation**

The quality checked Nanopore reads were error-corrected using Illumina PE reads. For error-correction the Illumina PE-reads were aligned to the Nanopore reads by using BWA aligner (Li, H., et al.). The paired-end reads were assembled using Abyss (Birol. I., et al.) followed by contig extension using Nanopore reads using SSPACE-LongRead (Boetzer, M., et al.). Super

scaffolding of the assembled scaffold was performed using SSPACE (Boetzer, M., et al.) and PLATANUS using the Nanopore and Matepair data. Final draft genome resulted after gap closure by GAPCLOSER and PLATANUS gap_close tool using Illumina data. The genome size was estimated using a k-mer distribution plot using JELLYFISH (Marcais, G., et al.). The repetitive elements were identified in the final assembled draft genome using Repeat Masker tool. The draft genome was hardmasked by using reference genomic repeats of *G. gallus*. The assembly and annotation workflow overview has been represented as Figure 2.

Simple sequence repeats prediction

Final assembled scaffolds were analysed for Simple Sequence Repeats (SSR) identification. SSRs like the di, tri, tetra, penta and hexa-nucleotide repeats in the genome were obtained using MISA (Version 1.0.0).

Genome prediction and annotation

Gene models was predicted on a hard masked draft genome, where the repetitive elements in the draft genome were masked using genomic repeats of *G. gallus* with Repeatmasker tool and further genes were predicted using AUGUSTUS with *G. gallus* as a reference model. The predicted proteins were annotated by using BLASTP (Altshul, S., et al.) against all Avian sequences downloaded from UniProt Protein Database.

Pathway Analysis of the draft genome

The predicted proteins were searched against the KEGG-KAAS server (Moriya, Y., et al.) for pathway analysis. *G. gallus* (chicken), *Meleagris gallopavo* (turkey), *Taeniopygia guttata* (zebra finch), *Falco peregrinus* (peregrine falcon) were used as reference organism for

pathway identification. The EuKaryotic Orthologous Groups (KOGs) were predicted using homology based approach.

Mitochondrial genome assembly and annotation

The generated scaffolds from the draft assembly were aligned against the *P. cristatus* mitochondria genome and the mapped reads were filtered and stitched using ABACUS software using the same reference (Zhou, TC., et al.). Further gap closure were performed with 3-7kb MP reads to generate an complete assembled mitochondrial genome. MITOS (Bernt, M., et al.) was used for gene annotation. Circular plot generated using GenomeVx (http://wolfe.ucd.ie/GenomeVx/) representing the localization of the gene in the assembled mitochondrial genome.

Phylogenetic tree construction

The assembled Peacock mitochondrial genome was searched against 695 avian mitochondrial genomes downloaded from NCBI. Based on the Blast-N homology results (with query coverage> 100, subject coverage > 95, % identity >85 and with 1% gaps allowed in the sequences). 51 mitochondrial genome sequences along with our assembled mitochondrial genome were filtered. Multiple sequence alignment with default parameters were performed using MUSCLE global sequence aligner. Phylogenetic trees were constructed using IQ-TREE version 1.5.6 (www.iqtree.org). The parameters used for phylogenetic tree construction were ultrafast boostrap (UFBoot, using the –bb option of 1000 replicates), and a standard substitution model (-m MFP) was given for tree generation. The generated trees from IQ-TREE tool were visualized using Figtree (http://tree.bio.ed.ac.uk/software/figtree/) and the Brach-support values were recorded from the output ".treefile". The trees were modified for better visualization under Trees section increasing order nodes were applied.

Protein domain analysis

Predicted proteins from Peacock, Chicken and Turkey with sequence length greater than 100 amino acids were considered for protein domain analysis. All the protein sequences from each organism were searched against Pfam-A database using Pfam scan for protein domain identification.

Avian protein families

The protein sequences of 48 avian genomes was downloaded from the link http://avian.genomics.cn/en/jsp/database.shtml apart from the predicted proteins of the draft genome. Sequences greater than 100 amino acids from all the avian genomes were filtered and concatenated to a single fasta file. These sequences were clustered using CD-HIT (Fu, L., et al.) with 90% alignment coverage for the shorter sequence with a length difference cutoff of 0.9. The single copy ortholog gene family present across all organisms and genes unique to peacock were filtered and annotated.

Genome conservation analysis

The assembled draft genome was aligned against the *G. gallus* genome using Chromosomer tool. Draft chromosomes were constructed based on alignment between fragments (scaffolds) using a reference genome. These reordered assembled genome was aligned against the Chicken genome using LAST aligner with NEAR (finding short-and-strong (near-identical) similarities.) parameter allowing for substitution and gap frequencies leading to the identification of orthologs. These query mapped regions were filtered with greater than 1% of the maximum length for visualization using Circos.

Results

DNA Sequencing data

Five libraries of 150 bp paired-end from Illumina HiSeq technology were generated. The short-insert reads of 489,114,747 accounted to genome coverage of 146.7X and long-insert reads of 302,884,819 sequences was about 90.9X coverage with a total coverage of 236X. Sequencing of three mate-pairs of 3-5Kb, 5-7Kb of and 7-10Kb yielded 72,915,033, 47,440,144 and 36,464,628 reads respectively with an approximate coverage of 21.9, 14.2 and 10.9 respectively, with a total of 156 million reads of 47X coverage. Oxford Nanopore technology was used to generate 366,323 long reads having of 2,398,560,283 bp with coverage of 2.3X. The complete sequencing was generated to a depth of ~287X from Illumina and Nanopore platforms. The coverage was based on assuming the peacock genome size of about 1 Gb (Table S1).

Genome assembly

The assembly was performed on Illumina reads with Abyss *de novo* assembler that resulted in ~932 Mb (mega base) of genome with an N50 of 1639 bp. The extension of the contigs were performed with Nanopore reads which generated scaffolds with N50 of 14,748 bp. Super scaffolding of the assembled scaffold was performed using SSPACE and PLATANUS with MP libraries that generated ~916 Mb genome with the N50 value of 168,140bp. The final gap closer was executed using GAPCLOSER program with MP and PE-LI libraries which generated a draft genome of 1.02 GB (giga base). The draft genome assembly of *Pavo cristatus* consists of 179,346bp scaffolds, with a N50 of 189,886bp with 37 scaffolds having sequence length >=1Mbp. Contigs above 5000 bp have covered a genome of ~0.915 Mb with N50 0.23 Mb. In the assembled genome there were ~0.4% of non ATGC characters (Table 1).

Complete mitochondrial genome of 16699 bp was obatained. Total of 22 tRNA, tow rRNA and 13 protein coding genes were identified in the assembled genome (Fig. 3a). A 100% similarity was observed with the preiously published Peacocks mitochondrial genome (Fig. S1a and S1b).

Transposable Elements (TE)

In the bird genome a total of 75,315,566 bp (7.3% of the genome) was predicted to have 5.5% of Retroelements (with SINEs 0.08% and LINEs 4.71%), 6.25 % total interspersed repeats with 0.84 % simple repeats and 0.21% low complexity regions. The DNA transposons identified in the genome was 0.71% (Table S2).

Protein coding gene annotation

A total of 23,153 proteins were predicted in the assembled draft genome using AUGUSTUS. Among them 95% predicted genes were annotated against the other Aves proteins. The 21,854 annotated proteins showed top similarity to species *Gallus gallus* (Chicken) with 11,398 proteins, *Meleagris gallopavo* (Common turkey) with 4059 proteins, *Amazona aestiva* (Blue-fronted Amazon parrot) with 1352 proteins and *Anas platyrhynchos* (Mallard) (*Anas boschas*) with 849 proteins. Thirteen species had about 100 to 400 annotated proteins. The remaining proteins were in the range of 1 to <100 proteins in about 62 species. From the annotations a total of 13,161 proteins showed similarity to uncharacterized protein annotation and some of the over represented proteins were Tyrosine-protein kinase, Sulfotransferase, Phosphoinositide phospholipase C, Tetraspanin, Phospholipid-transporting ATPase, Olfactory receptor, Polypeptide N-acetylgalactosaminyltransferase, Transporter, Keratin, Hexosyltransferase, Protein Wnt, Kinesin-like protein, Gap junction protein, Claudin, POU domain protein,

Sodium/hydrogen exchanger, Phospholipid-transporting ATPase, Histone-lysine N-methyltransferase and others (Table S3). The gene ontology annotations showed to have Gene Ontology (GO) descriptions for 18,295 proteins. Among them, 14,490 proteins have Molecular Function; 11,679 have Biological Process and 13,736 proteins have Cellular Component as functional categories.

About 17.7% of proteins were found to have pathway information against the KEGG database (Table S4). Some of the overrepresented annotations were Kinases like MAPK (mitogen-activated protein kinase); JNK (c-Jun N-terminal kinase); RAF (RAF proto-oncogene serine/threonine-protein kinase); AKT (RAC serine/threonine-protein kinase); protein kinases and different GTPases.

Proteins searched against the KOG annotations showed a total of 20,937 proteins having annotations. Among them, the most abundant annotations include Zn-finger, transmembrane receptor, ubiquitin ligase, Leucine rich repeat, Cadherin repeats, Serine/threonine protein kinase, Collagens, Ankryin repeat, Fibrillins, Voltage-gated Ca2+ channels and Hormone receptors (Table S5). The peacock proteins when searched against the human proteins showed expansions in ontologies for cell morphogenesis, neuronal projection and development and GTPases (Table S10 and Fig. S4).

Simple sequence repeats

A total of 399,493 SSRs were identified from the peacock genome assembly. The largest fraction of SSRs identified were mononucleotide (60.04%), followed by tetra nucleotide (26%), di nucleotide (8.51%), tri nucleotide (4.31%), penta nucleotide (1.03%) and finally hexa nucleotide (0.13%). Among the SSRs identified, A (49.2%) and T (44.9%) accounted for 94.1% of the mono-nucleotide repeats. AT (23.8%), TA (16.5%), TG(13.7%), AC(10.6%) and

CA (10.32%) accounted for 75% of the di-nucleotide repeats. while TTG (9.9%), AAT (9.6%), AAC (9.4%), TTA (7.1%), ATT (4.5%), TAA (3.5%), CAA (3.1%) and GGA (2.69%) accounted for 49.7% of the tri-nucleotide repeats (Table S6).

Avian protein family analysis

A total of 748,544 protein sequences from 49 avian species have 653,497 protein sequences of length above 100 amino acids (Table S7A). A total of 240,853 gene clusters were generated of which 41 gene clusters had single copy orthologs in all avian species (Table S7B and Table S7C). With the above stringent cutoff we observed 15,913 gene clusters were unique to peacock species.

Phylogeny and Genome comparisons

The phylogeny of 51 mitochondrial genome sequences along with peacock genome showed a clade consisting of *Pavo* species and *Gallus* (red junglefowl, Sri Lankan junglefowl, grey junglefowl, grey junglefowl), Bambusicola (Mountain Bamboo-partridge, Chinese bamboo partridge) and Francolinus (Chinese francolin). It can be observed many species were distributed in two different clades where 34 species were found in one clade and five species in other clade. Some of the endemic or native bird species like *Arborophila ardens*, *Acryllium vulturinum* and *Numida meleagris* were found as clear outgroup of species in this study (Fig. 3b).

Predicted proteins from Peacock, Chicken and Turkey were searched for protein domain analysis. 81% of the Pfams were common among the three species. About 94%, 98.4% and 99.7% predicted Pfam domains were identified in Peacock, Chicken and Turkey respectively. There were 255, 69 and 14 Pfam domains found to be unique in the species mentioned above respectively (Fig. 4).

The assembled Peacock genome was reordered for pseudo chromosomes generation against the simple repeat masked Chicken genome (1.21 GB, Warren, WC., et al.) using Chromosomer which generated a overall reordered Peacock genome of about 597MB. The right side of the image represents the reference genome and left side of the image represents the Peacock genome (Fig. 5).

Conclusions

Third generation sequencing in avian genomics where long reads having the substantiality to improve genome assembly will benefit understanding the organisms in the structurally complex regions having repeat elements and isoforms in the genome (Goodwin, S., et al.). Using a combination of short reads of different insert sizes as well as mate pair reads generated from Illumina technology along with long reads from Oxford Nanopore, we obtained an improved assembly and a draft genome of the Indian Blue Peacock (*Pavo cristatus*). In comparison to other avian genomes (Zhang, G., et al.), the current 290X sequencing depth obtained from our study is one of the highest. With a N50 of 0.23Mb, we presented here a reasonably reliable draft genome for the peacock species. The inclusion of Nanopore reads 366,323 for scaffolding followed by subsequent gap-closing using Illumina data led to a 26.2% reduction in the number of scaffolds and a 50.65% and 115% increase in the scaffold and contig N50, respectively. With only 2.3X of long reads, a significant improvement in the assembly was observed. On the contrary, the assembly contained less than 0.4% of unknown nucleotides, which is very low for a draft assembly. With the addition of more long reads along with transcriptomic sequencing along with scaffolding and/or gap closure tools, further improvement in the assembly can be achieved.

Peacock seems to defy the Darwinian laws of natural selection. These concern were raised by no other than Darwin himself. Hence, he proposed the theory of the sexual selection where the female can choose for a male with a certain phenotypic feature such as brilliant color or a long tail (Burgess, S.). Peacock's brilliantly colored long tail feathers seems to evolve at the cost of finding its female partner thereby contributing its beneficial genes, even at the cost of making itself venerable to predators. A female peafowl in turn tends to choose the mate with the largest and decorated plumage, which indirectly reflects its healthiness and capacity to wade off potential competitors thereby selecting the most suitable male. Peacocks beautiful feathers with it all its artistry surely provides it with an advantage to impress the females (Dakin, R., et al.). Understanding the formation of beautiful feathers from the genomic context will help in resolving several evolutionary theories on sexual selection that have been discussed on this species.

Pigment particles are embedded into the newly grown peacock feathers during the molting season which seems to absorb light of selective wavelength there by imparting to the color of the plumage (Mercedes Foster, Rennee Riedler, et al.) Pigment morphogenesis in *Gallus* has been reported by a process of melanoblasts migration and colonization into feather bud where they differentiate to produce the pigment, melanin (Kelsh, RN., et al.). The molecular mechanisms that control the pigment cell migration have been narrowed down to proteins Kit1 and FGFs which maintain the melanoblasts migration to feathers. Kit and FGF proteins have also been identified in our current study (Table S3). Understanding of these proteins in the patterned formation will help decode the pigment pattern morphology in peacocks. These pigmented patterns play a role in communication, choice of mate and in some species it can help in camouflage (Burgess, S)

It has been observed that the variations in the genome size among bird species are very low (Table S8). The genome complexities of a species are influenced by the Transposable elements (TE) that are believed to play a crucial role (Kapsuta, A., et al.). The long read sequences have significantly helped in resolving the TEs in genome quality and assembly. Peacock genome comparisons with Turkey and Chicken have showed closeness to the Chicken species. The

mitochondrial phylogeny also revealed similar findings. Homology searches have shown several important gene family expansions such as Kinases, Zn finger proteins, GTPases and others. Their roles in biology, development and evolution of the Peacocks have to be further explored.

To summarize, we have assembled the *Pavo* genome using Illumina and Oxford nanopore technology. The genome information can be valued and explored by avian enthusiasts to further understand about this bird. Though not critically endangered yet, in India, peafowl population is surely at a decline in the wild due to massive deforestation and habitat loss. Thus is further compounded by increased poaching for meat and feathers. Our genome sequencing initiative of *Pavo cristatus* is not just only from a conservational viewpoint, but also to preserve a heritage associated with this bird that runs through centuries and that bears a strong attachment to the national psyche.

Availability of supporting data

Supplementary data contains, read statistics, annotation, repeats identification, orthology analysis, mitochondria assembly and annotation. Figures, Gene ontology. DNA and library preparation protocols.

Raw Data in SRA

Raw reads (Illumina and Nanopore) are available in the Sequence Read Archive (SRA), and the Whole Genome Shotgun project has been deposited at GenBank under SRA Submission ID: SUB3108024, Bioproject: PRJNA413288 and Biosamples SAMN07739105 : SKPea2016_SI, SAMN07739104 : SKPea2016_LI, SAMN07739101 : FPL_3_5KB, SAMN07739102 : FPL_5_7KB, SAMN07739103 : FPL_7_10KB and SAMN07739107 : FPL_Nano.

Competing interests

The author(s) declare that they have no competing interests.

Authors contributions

RD, AS, KP performed wet lab experiments; RD designed work plan, experiments and logistics; SS, VR, KP SG IM and AR assisted with the work; RS provided samples from bird; BR, SK performed data analysis and interpretation; SK, BR, RD drafted the manuscript and SK overseen the whole project.

Acknowledgements

Department of Biochemistry, AIIMS, New Delhi for providing space and infrastructure to carry on the work. RD for providing partial funding. Kritika Tripathi for inputs with manuscript editing. Raja and Sudha from Genotypic Technology Pvt. Ltd for providing sequencing services and also Deepti, Shalini, Nagu, Abhi and Manish. BR acknowledges IUT 34-4.

Tables

Table 1. *De novo* assembly statistics of the Peacock genome.

Description	Contigs	Nanopore Scaffold	Super Scaffolds	GapClosed	>1000 Kb	>5000 Kb
Contigs	685241	281272	179346	179346	34181	15026
Maximum Length	49159	251510	2390121	2488982	2488982	2488982
Minimum Length	300	5	265	265	1000	5000
Average Length	1360	3250	5111	5729	-	-
Total Length	932162464	914363908	916720956	1027551907	954483822	915373606
Length >= 100 bp	685241	281271	179346	179346	34181	15026
Length >= 200 bp	685241	281271	179346	179346	34181	15026
Length >= 500 bp	616120	186433	93727	93727	34181	15026
Length >= 1 Kbp	363428	104479	34168	34181	34181	15026
Length >= 10 Kbp	1591	24748	9249	10311	10311	10311
Length >= 1 Mbp	0	0	27	37	37	37
Non-ATGC #	350325	42696911	49169831	4034372	4032567	3978757
Non-ATGC %	0.038	4.67	5.364	0.393	0.422	0.435
N50 value	1639	14748	168140	189886	218023	232312

Figure legend

Figure 1. The beautiful and charismatic photo of Indian blue peacock (*Pavo cristatus)* bird.

Figure 2. Detailed workflow for *de novo* genome assembly and annotation.

Figure 3a. Circular representation of Peacock mitochondrial genome with genes predicted.

Figure 3b. Phylogenetic tree generated from mitochondrial genome from 52 different avian species.

Figure 4. Circular image of the assembled peacock genome aligned against the *G. gallus* genome using Chromosomer tool. Draft chromosomes were generated by similarity between scaffolds which were arranged on the reference chicken genome. Circos was used for visualization.

Figure 5. Venn diagram showing common and unique Protein family domains (Pfam) between Peacock, Chicken and Turkey proteins.

References:

Gadagkar, R., 2003. Is the peacock merely beautiful or also honest?. Current Science, 85(7), pp.1012-1020.

Kushwaha, S., and Kumar, A. 2016. A Review on Indian Peafowl (*Pavo cristatus*) Linnaeus, 1758. Journal of Wildlife and Research, 4, 42-59.

Kadgaonkar, Shivendra B. 1993. The peacock in ancient Indian art and literature. Bulletin of the Deccan College Research Institute, vol. 53, pp. 95–115. JSTOR, www.jstor.org/stable/42936434.

Brickle, N. 2002. Habitat use, predicted distribution and conservation of green peafowl (*Pavo muticus*) in Dak Lak Province, Vietnam. Biological Conservation, 105: 189-197.

Jackson, C. 2006. Peacock. London: Reaktion Books Ltd.

Zhang, G., Jarvis, E. D., and Gilbert, M. T. P. 2014. A flock of genomes. Science 346, 1308–1309.

International Chicken Genome Sequencing Consortium. 2004. Sequence and comparative analysis of the chicken genome provide unique perspectives on vertebrate evolution. Nature, 432(7018), 695.

Kajitani, R., Toshimoto, K., Noguchi, H., Toyoda, A., Ogura, Y., Okuno, M., Yabana, M., Harada, M., Nagayasu, E., Maruyama, H. and Kohara, Y., 2014. Efficient de novo assembly of highly heterozygous genomes from whole-genome shotgun short reads. Genome research, 24(8), pp.1384-1395.

Loman, N. J. and Quinlan, A. R. 2014. Poretools: a toolkit for analyzing nanopore sequence data. Bioinformatics, 30(23), 3399-3401.

Li H. and Durbin R. 2009. Fast and accurate short read alignment with Burrows-Wheeler Transform. Bioinformatics, 25:1754-60.

Birol, I., Jackman, S.D., Nielsen, C.B., Qian, J.Q., Varhol, R., Stazyk, G., Morin, R.D., Zhao, Y., Hirst, M., Schein, J.E. and Horsman, D.E., 2009. De novo transcriptome assembly with ABySS. Bioinformatics, 25(21), pp.2872-2877.

Boetzer, Marten, and Walter Pirovano. 2014. SSPACE-LongRead: scaffolding bacterial draft genomes using long read sequence information. BMC bioinformatics 15.1: 211

Boetzer, M., Henkel, C.V., Jansen, H.J., Butler, D. and Pirovano, W., 2010. Scaffolding pre-assembled contigs using SSPACE. Bioinformatics, 27(4), pp.578-579.

Marcais, G and Kingsford, C. 2011. A fast, lock-free approach for efficient parallel counting of occurrences of k-mers. Bioinformatics 27(6): 764-770.

Altschul, S.F., Gish, W., Miller, W., Myers, E.W. and Lipman, D.J., 1990. Basic local alignment search tool. Journal of molecular biology, 215(3), pp.403-410.

Moriya, Y., Itoh, M., Okuda, S., Yoshizawa, A.C. and Kanehisa, M., 2007. KAAS: an automatic genome annotation and pathway reconstruction server. Nucleic acids research, 35(suppl_2), pp.W182-W185.

Zhou, T.C., Sha, T., Irwin, D.M. and Zhang, Y.P., 2015. Complete mitochondrial genome of the Indian peafowl (*Pavo cristatus*), with phylogenetic analysis in phasianidae. Mitochondrial DNA, 26(6), pp.912-913.

Bernt, M., Donath, A., Jühling, F., Externbrink, F., Florentz, C., Fritzsch, G., Pütz, J., Middendorf, M. and Stadler, P.F., 2013. MITOS: improved de novo metazoan mitochondrial genome annotation. Molecular phylogenetics and evolution, 69(2), pp.313-319.

Fu, L., Niu, B., Zhu, Z., Wu, S. and Li, W., 2012. CD-HIT: accelerated for clustering the next-generation sequencing data. Bioinformatics, 28(23), pp.3150-3152.

Warren, W.C., Hillier, L.W., Tomlinson, C., Minx, P., Kremitzki, M., Graves, T., Markovic, C., Bouk, N., Pruitt, K.D., Thibaud-Nissen, F. and Schneider, V., 2016. A new chicken genome

assembly provides insight into avian genome structure. G3: Genes, Genomes, Genetics, pp.g3-116.

Goodwin, S., McPherson, J.D. and McCombie, W.R., 2016. Coming of age: ten years of next-generation sequencing technologies. Nature Reviews Genetics, 17(6), p.333.

Zhang, G., Li, C., Li, Q., Li, B., Larkin, D.M., Lee, C., Storz, J.F., Antunes, A., Greenwold, M.J., Meredith, R.W. and Ödeen, A., 2014. Comparative genomics reveals insights into avian genome evolution and adaptation. Science, 346(6215), pp.1311-1320.

Burgess, S., 2001. The beauty of the peacock tail and the problems with the theory of sexual selection. Journal of Creation, 15(2), pp.94-102..

Dakin, R. 2008. The role of the visual train ornament in the courtship of peafowl, *Pavo cristatus*. Masters Abstracts International, 47/03: 97.

Mercedes S Foster 1975. The overlap of molting and breeding in some birds. The Condor 77:304-314.

Renee Riedler, Christel Pesme, James Druzik, Molly Gleeson, Ellen Pearlstein The Journal of the American Institute of Conservation 2014

Kelsh, R.N., Harris, M.L., Colanesi, S. and Erickson, C.A., 2009. Stripes and belly-spots—a review of pigment cell morphogenesis in vertebrates. Seminars in cell & developmental biology. Vol. 20, No. 1, pp. 90-104.

Kapusta, A. and Suh, A., 2017. Evolution of bird genomes—a transposon's- eye view. Annals of the New York Academy of Sciences, 1389(1), pp.164-185.

Webservers:

FastQC : http://www.bioinformatics.babraham.ac.uk/projects/fastqc/

AUGUSTUS : http://augustus.gobics.de/

PLATANUS : http://platanus.bio.titech.ac.jp/

ABACUS : http://abacas.sourceforge.net/Manual.html

MISA: http://pgrc.ipk-gatersleben.de/misa/

REPEATMASKER : http://www.repeatmasker.org/

KOGs : https://genome.jgi.doe.gov/portal/help/kogbrowser.jsf

KAAS : http://www.genome.jp/tools/kaas/

MITOS : http://mitos.bioinf.uni-leipzig.de/

MUSCLE : https://www.drive5.com/muscle/

PFAMSCAN : https://www.ebi.ac.uk/seqdb/confluence/display/THD/PfamScan

LAST : http://last.cbrc.jp/

CHROMOSOMER : https://github.com/gtamazian/chromosomer

CIRCOS : http://circos.ca/

Figure 1

Click here to download Figure Fig 1. Peacock.jpg

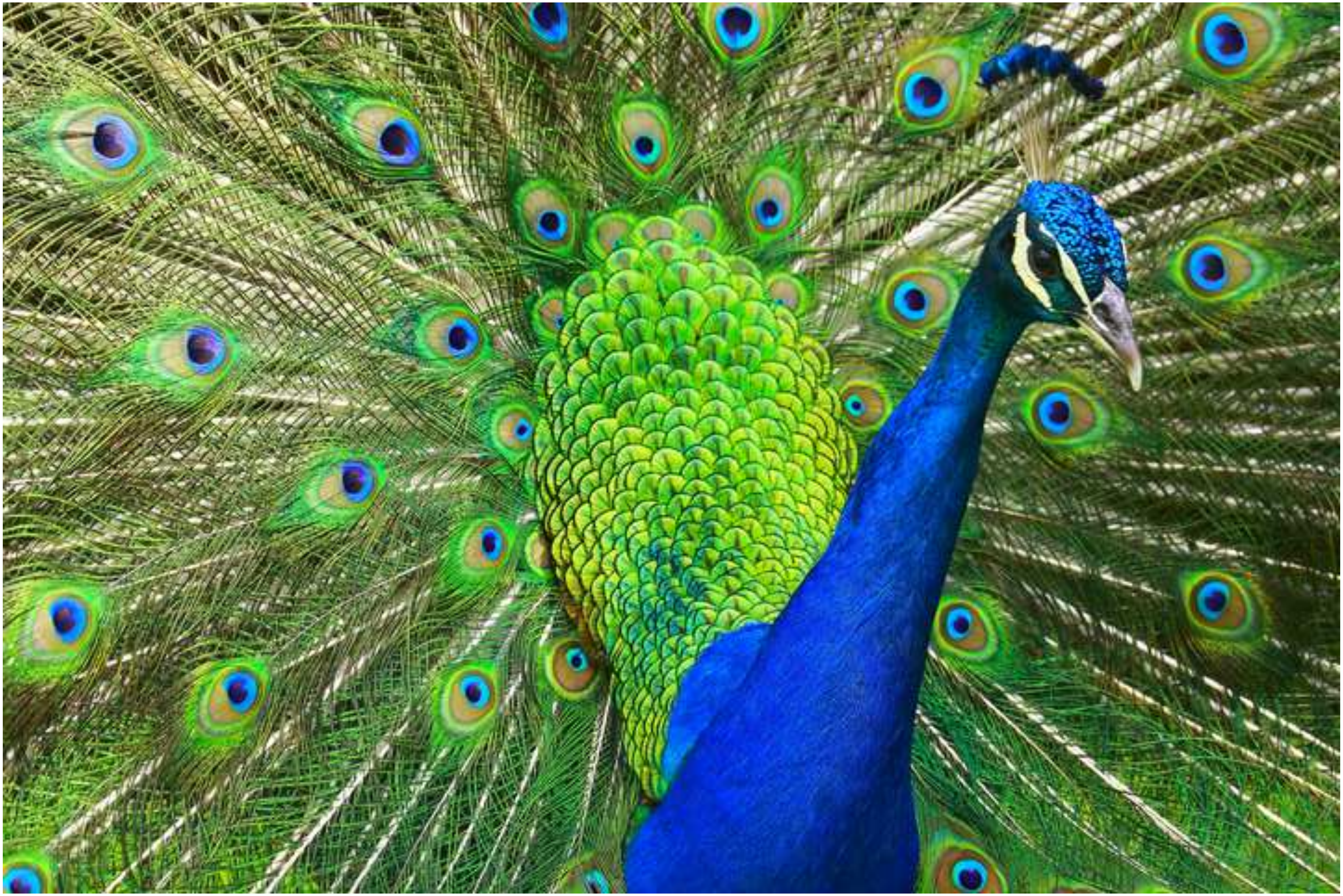

 Click here to download Figure Fig 2. Workflow.jpeg

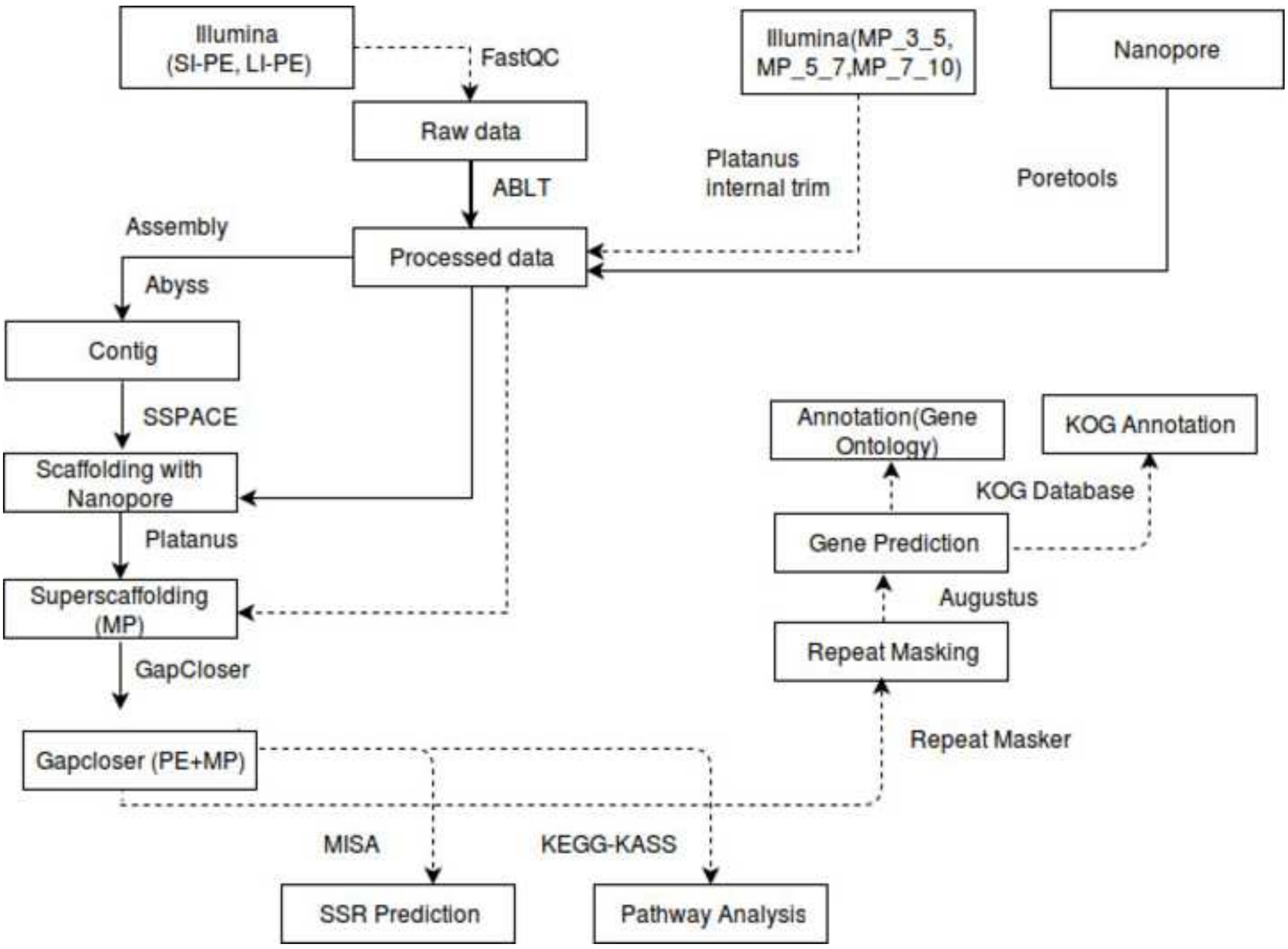

Figure 3a

Click here to download Figure Fig 3a. Mitochondria genome with genes.pdf

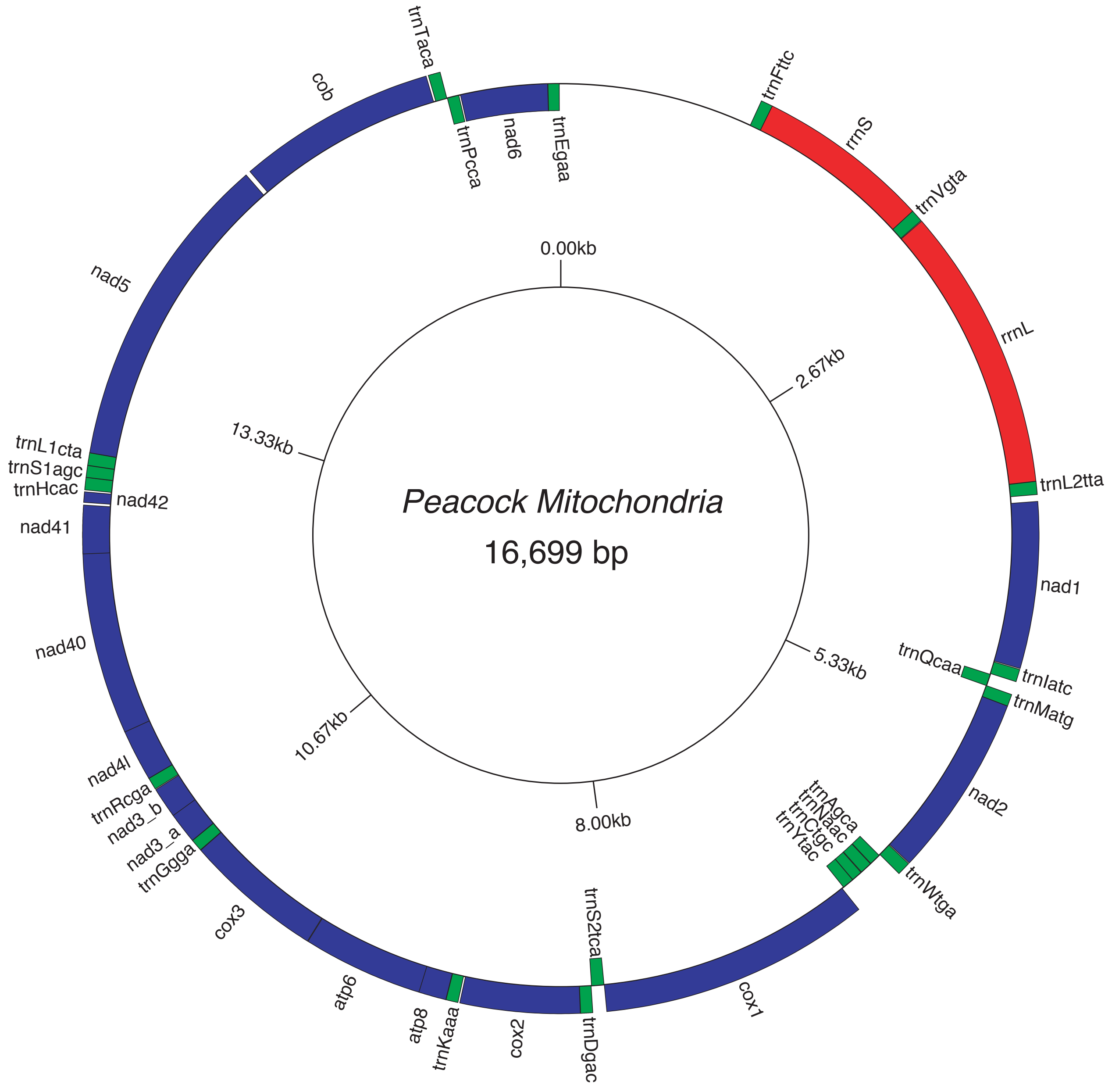

Figure 3b

Click here to download Figure Fig 3b. Birds mitochodria phylogeny.pdf

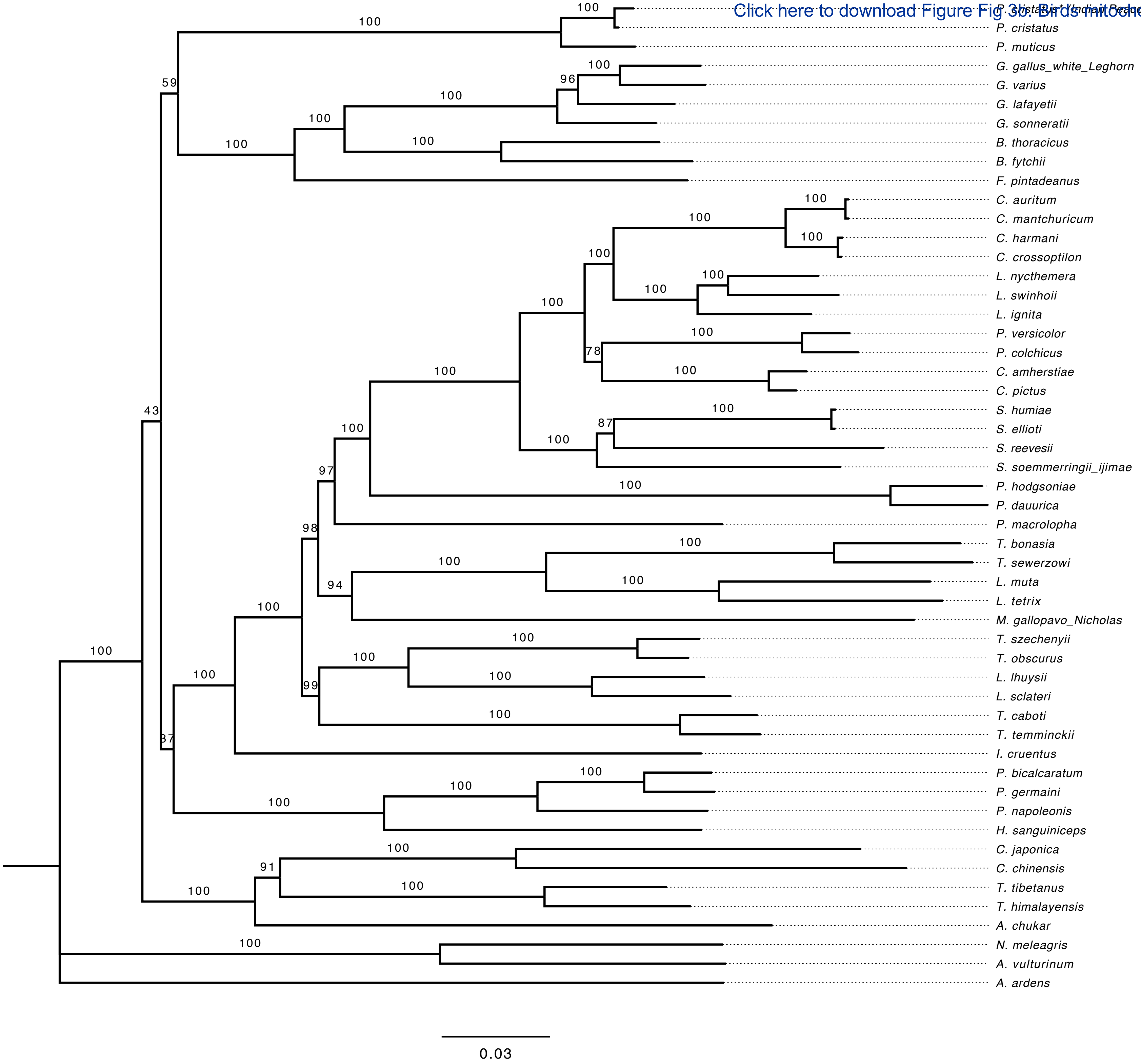

Figure 4

Click here to download Figure Fig 4. Pfam domains common with chicken and turkey.png

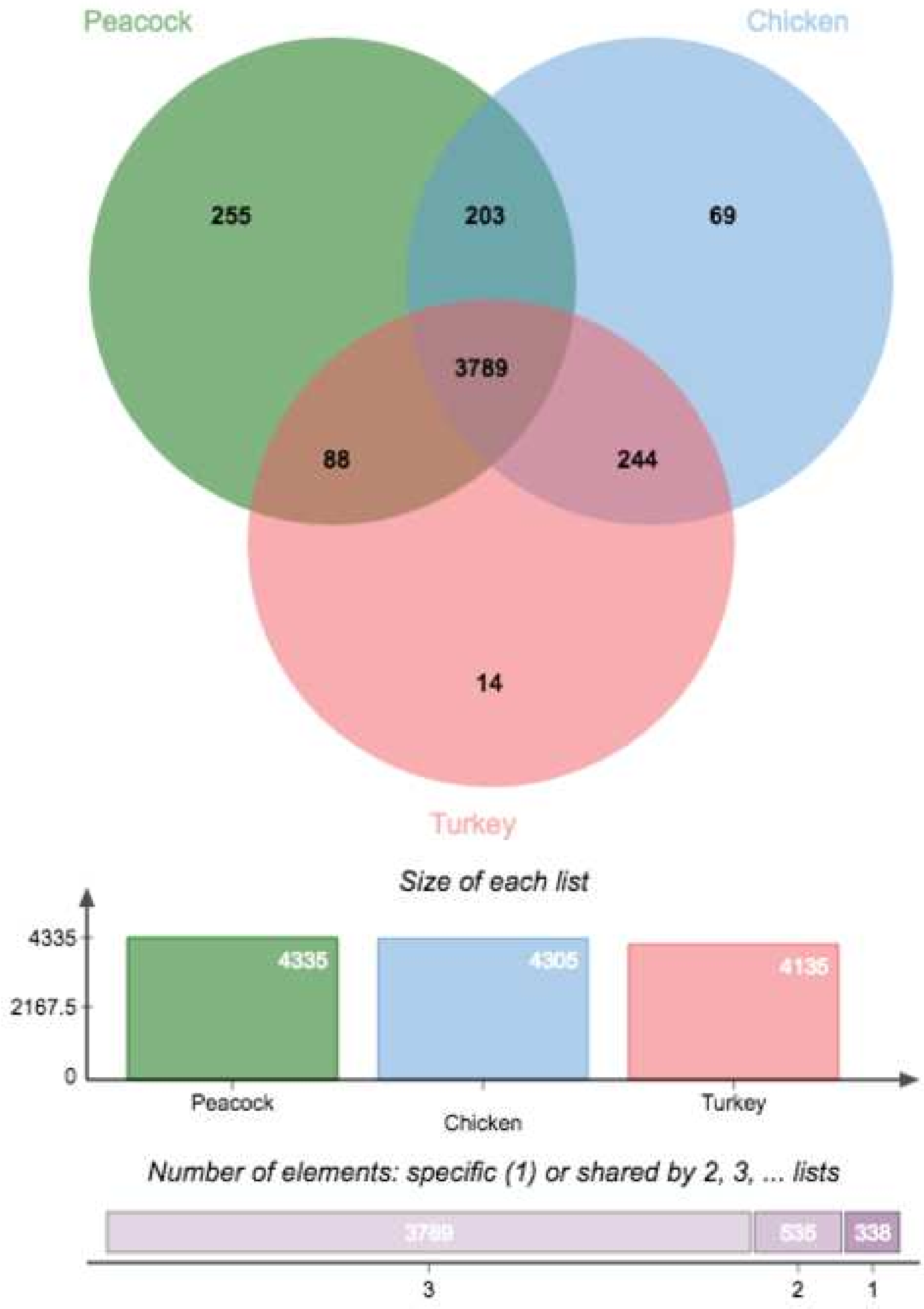

Figure 5

Click here to download Figure Fig 5. Peacock vs Gallus circos.png

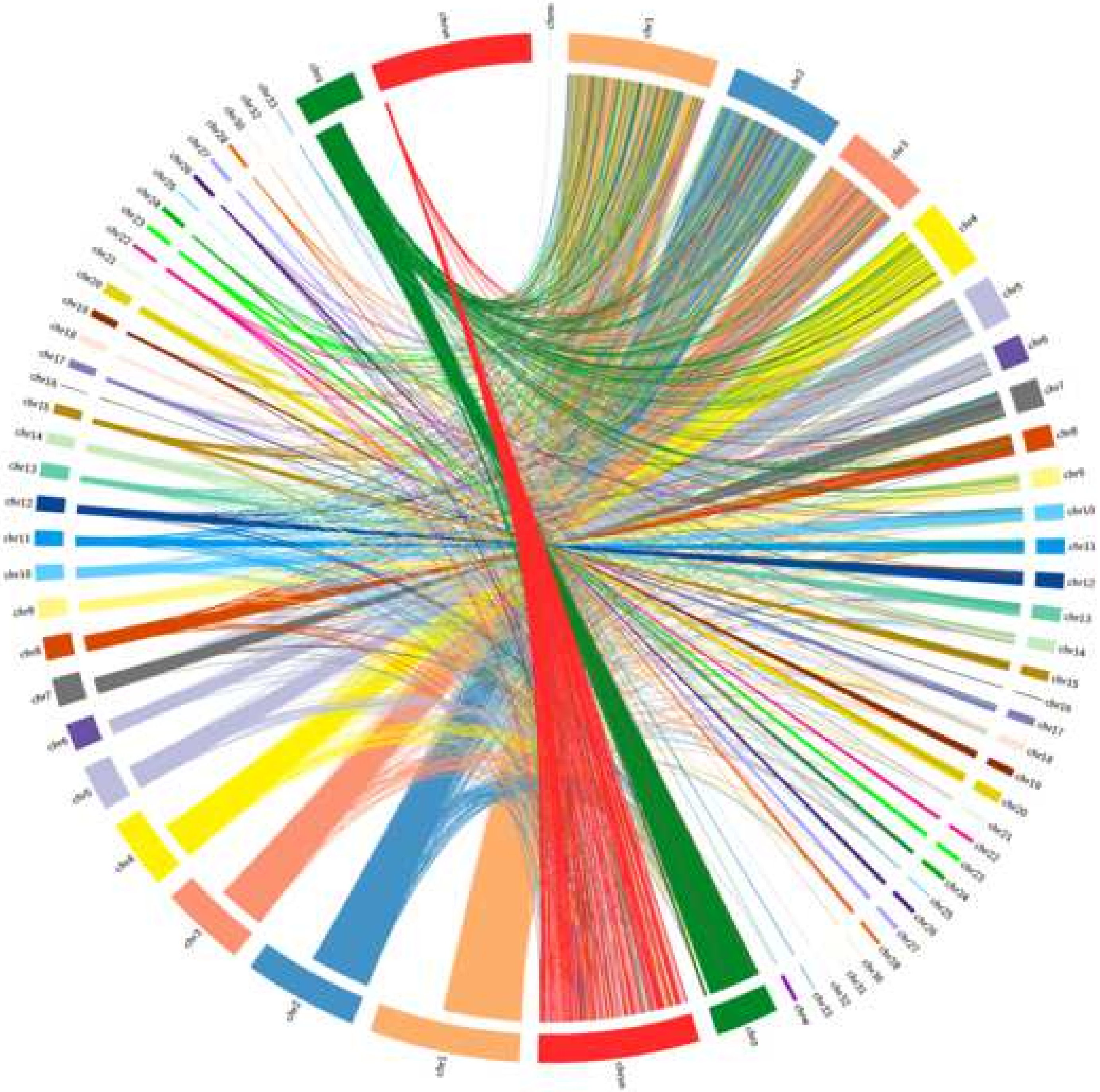

Click here to access/download
Supplementary Material
Supplementary_Notes_Mitochondria.docx

Click here to access/download
Supplementary Material
Description of all the tables and figures.docx

Click here to access/download
Supplementary Material
Table_S1_ReadStats_Table_S2_TEs.xlsx

Table S3 Gene annotations

Click here to access/download
Supplementary Material
Table_S3_Gene_annotation.xlsx

Table S4 KEGG annotations

Click here to access/download
Supplementary Material
Table_S4_KEGG_annotation.xlsx

Table S5 KOG annotations

Click here to access/download
Supplementary Material
Table_S5_KOG_annotation.xlsx

Table S6 Blast against Human proteins

Click here to access/download
Supplementary Material
Table_S6_BlastVsHumanProteins.xlsx

Table S7 SSR analysis

Click here to access/download
Supplementary Material
Table_S7_SSR.xlsx

Table S8 Orthology

Click here to access/download
Supplementary Material
Table_S8_Orthology.xlsx

Table S9 Mitochondria species description

Click here to access/download
Supplementary Material
Table_S9_Species_for_mitochondriaxlsx.xlsx

Table S10 Pfam annotations

Click here to access/download
Supplementary Material
Table_S10_Pfam_Analysis.xlsx
