# Supplementary material for: De novo assembly of the Indian blue peacock (Pavo cristatus) genome using Oxford Nanopore technology and Illumina sequencing
Source: Gigascience. 2019 May 11;8(5):giz038. doi: 10.1093/gigascience/giz038 (PMC6511069; doi:10.1093/gigascience/giz038)
Supplement: GIGA-D-18-00280_Revision_1.pdf [file giz038_giga-d-18-00280_revision_1.pdf]

GigaScience

De novo genome assembly of the Indian Blue Peacock (Pavo cristatus), from Oxford Nanopore and Illumina sequencing

--Manuscript Draft--

Manuscript Number:	GIGA-D-18-00280R1
Full Title:	De novo genome assembly of the Indian Blue Peacock (Pavo cristatus), from Oxford Nanopore and Illumina sequencing
Article Type:	Data Note
Funding Information:	
Abstract:	Background<br>Pavo cristatus, the Indian blue peacock are geographically found distributed in natural habitats of South Asia. The peacock has been described as one of the most elegant, majestic, and beautiful bird species. Since prehistoric times they have been described in Indian culture and has been adopted as the national bird of India. Its length varies from 92-125 centimeter (without train), weighing about 4-8 Kilograms and lives up to 20 years in the wild. This avian species have been very important in the fields of phylogenetics, developmental studies, sexual reproduction and speciation. The individuals of avian genomics have contributed immensely towards understanding the vertebrate genome evolution. Here we present the first draft genome sequence of P. cristatus, yet another important and popular bird species to further add values and gain insight into avian genomics.<br><br>Findings<br>For the first time in avian genomics, Oxford Nanopore technologies (ONT) have been used for the whole genome assembly. Along with the above sequencing technology we have sequenced different DNA insert size libraries from Illumina technology for the peacock DNA. We performed de novo genome assembly by integrating the reads from Illumina short insert, long insert, multiple mate-pair reads along with Oxford Nanopore long reads using multiple genome improvement tools. A draft of the peacock genome of about 0.915 Gigabases (Gb) with a N50 of 0.23 Megabases (Mb) was assembled. Annotations with other avian species, protein families, KEGG were performed for functional understanding by insilico approaches. Proteins were compared against Chicken, Turkey and Human to obtain evolutionary similarities and uniqueness of the Pavo species.<br><br>Conclusions<br>Our study is the first report of a high quality draft genome of P. cristatus using a hybrid assembly generated from Illumina sequencing reads and long reads from ONT. The long read chemistry was found to be useful in addressing challenges related to de novo assembly particularly at regions containing repetitive sequences that span longer than the read length and which cannot be resolved using short read based assembly alone. miniION based ONT offers an affordable and reliable platform to achieve this. Observation from our study showed a significant improvement in genome assembly with fewer gaps and a reliable N50 when used together with Illumina reads. Further a comparative genomics with Gallus gallus (Chicken) and Meleagris gallopavo (Turkey) have shown insights into the gene families and their conserved domains. Peacock proteins were also compared with human proteins to understand the functional components that were conserved after the speciation split. Further, the phylogentic tree on the conserved genes from the avian species showed a grouping amongst the clade of birds based on their ability to fly.
Corresponding Author:	Subhradip Karmakar, PhD<br>All India Institute of Medical Sciences<br>New Delhi, Delhi INDIA
Corresponding Author Secondary Information:	
Corresponding Author's Institution:	All India Institute of Medical Sciences
Corresponding Author's Secondary	

Powered by Editorial Manager® and ProduXion Manager® from Aries Systems Corporation

**Institution:**	
**First Author:**	Subhradip Karmakar, PhD
**First Author Secondary Information:**	
**Order of Authors:**	Subhradip Karmakar, PhD
	Ruby Dhar
	Ashikh Seethy
	Karthikeyan Pethusamy
	Vishwajeet Rohil
	Sunil Singh
	Kakali Purkayastha
	Sandeep Goswami
	Rakesh Singh
	Indrani Mukherjee
	Ankita Raj
	Tryambak Srivastava
	Sovon Acharya
	Balaji Rajashekhar
**Order of Authors Secondary Information:**	
**Response to Reviewers:**	Reply to reviewer's comments (see also attached response letter).<br><br>We thank the editors and the reviewer for reviewing our manuscript titled " De-novo genome assembly of Indian Blue peacock (Pavo Cristatus) Oxford Nanopore and Illumina sequencing . Following reviewers comments and suggestions, we have modified the manuscript incorporating all the necessary changes. Additional figures are incorporated as per the reviewer suggestions while non relevant items are removed. File containing a point by point reply to reviewers questions was also attached. We also uploaded the raw data at NCBI SRA as requested by one of the reviewer.<br><br>1: "of the Indian Blue Peacock"<br>Response: The above has been changed<br><br>3: [remove "reads", unnecessary]<br>Response: Reads have been removed from the title<br><br>26-27: "are native to South Asia"<br>Response: The authors accepted this suggestion and the same has been modified in the article.<br><br>27-28: "The peacock has been described as one of the most elegant, majestic, and beautiful bird species."<br>Response: The authors thank the reviewers for suggesting this change and as per their expert suggestions, the above sentence has been included in the article.<br><br>38,40: standardizing how you refer to Oxford Nanopore sequencing would be helpful. "Oxford Nanopore technology" vs. "long read Nanopore technologies"<br>Response: Authors thank the reviewer for raising the concern and we have now used "Oxford Nanopore technology" in the article. Further a detail discussion of how ONT (Oxford Nanopore technology) long read chemistry was helpful to improve the genome assembly is discussed in the conclusion section. The authors want to humbly state that a hybrid approach of genome assembly using short reads along with long reads seems to improve genome quality that otherwise might not be achieved using just only one of

Powered by Editorial Manager® and ProduXion Manager® from Aries Systems Corporation

	the type. This could be due to repetitive elements in the genome.<br><br>60: comparing peacock proteins to human seems like much less informative than comparisons to chick and turkey.<br>Response: When we submitted the manuscript under research category previously one of the reviewers suggested to do a comparison of peacock proteins to human. Hence we included the results of comparison against human proteins. In this present manuscript, we have included the comparisons to Chicken and Turkey.<br><br>73-84: I'm not certain that the review of avian genomics is helpful. This could be condensed to a couple of sentences with appropriate citations.<br>Response: Authors agree with the reviewer's suggestion. This section has been condensed and references have been included.<br><br>89-90: "The long read chemistry …" I think this sentence is supposed to end with "repeat rich regions of the genome".<br>Response: Authors agree with the reviewer's suggestion. This has been modified in the manuscript as per reviewers comment.<br><br>90-91: remove, redundant with the rest of the paragraph or replace as the first sentence in the paragraph<br>Response: Authors thanks the reviewers for his comments and this has been corrected in the manuscript.<br><br>91-92: "Comparative genomics"<br>Response: This has been modified.<br><br>93-95: How will knowledge of the sex determination genes aid in selective breeding? I'm not certain this<br>Response: Authors agree with the reviewers suggestions and taking into duly consideration of their concern, this sentence has been modified in the updated manuscript.<br><br>97: "should improve"<br>Response: Authors assure the reviewer that this has been duly considered and modified in the manuscript<br><br>106: "10 seconds" (add space)<br>Response: Space has been added.<br><br>107: "10 minutes" (add space)<br>Response: Space has been added.<br><br>108: It's not clear whether the Binding Buffer was added to the collection tube with the ReliaPrep column or the tube that contained the sample mixture.<br>Response: Authors confirmed the working protocol from the concerned investigators and concluded that binding buffer was added to the collection tube and the entire sample preparation was carried out strictly adhering to the manufacturers protocol. The same has been incorporated in the text.<br><br>126: approximate not approx.. Abbreviation is unnecessary here.<br>Response: Abbreviation has been removed from the manuscript<br><br>205-206: This is a run on sentence. Should end with "Metrichor V.2.43.1" followed by a citation or URL for the software.<br>Response: The authors agree with the concern raised and sentence has been modified with URL included.<br><br>224-227: This section should be simplified to one sentence and combined with the prior paragraph.<br>Response: The paragraph is merged and modified.

Powered by Editorial Manager® and ProduXion Manager® from Aries Systems Corporation

229-234: Citations needed for repeatmasker tool, augustus, and Uniprot protein database. Half of this paragraph is a repeat of a prior section and could be combined there.
Response: The paragraph is merged and references have been included.

253: URL or citation needed
Response: URL included.

257: I think you mean “selected” here, not “filtered”. To my understanding, “filtered” implies exclusion.
Response: The authors agree with the reviewer that the original sentence was misleading and as per their valued suggestions necessary correction has been made in the text. We thank the reviewer for this.

269: Citation needed for Pfam
Response: Citation is included.

282-284: This should be one sentence: “Draft chromosomes were constructed by aligning the assembled draft genome against the G. gallus with the Chromosomer tool” with a citation or URL for the Chromosomer tool.
Response: The authors agree with the reviewer on this and sentences are now merged as per their recommendation. Further URL for the tool is included in the text for readers.

304-315: Citations needed for Abyss, SSPACE, PLATANUS, GAPCLOSER tools.
Response: The tools have already been cited in 214-218.

318-320: Citation needed for the previously published peacock mitogenome.
Response: Authors want to state that information /data on Peacock mitogenome has not been included in thus present manuscript .

322-326: This section needs a thorough rewrite for clarity.
Response: Authors have seriously taken the positive feedback of the reviewers comments and this section has been rewritten and one more table has been included for comparison with other bird species for better clarity. We thank the reviewers for this critical suggestion.

329-334: This data could be easier presented in a table. The very few homologous genes identified with blast hits between the peacock and parrot and mallard genomes suggest that a too stringent blast search was used.
Response: Authors agree with the reviewers comment. New figures and tables have been included in the manuscript.

334: “Thirteen species had about 100-400 annotated proteins”. This is a misstatement of these results. The authors did not annotated genes in the other bird genomes. They identified homologous genes using a very stringent requirement of similarity. Again, this data would be better presented as a table or figure, ideally as a histogram with the various bird species binned by the number of blast hits. identified between each species and the genes from the peacock genome.
Response: The authors agree on this and appreciate the reviewers concerns. This section have been rewritten and modified in this updated manuscript. The significant results are represented as pie and venn chart, histograms with complete details in tables.

337: “overrepresented” It isn’t clear what criteria or method was used for overrepresentation here.
Response: The authors want to state that this was based on the count, now this section is modified.

346-350: The interpretation of the “overrepresented” categories here isn’t clear either.
Response: This section is modified.

374: If the majority of peacock genes (15K out of 23K) clustered by themselves (ie found no homolog in any of the 49 avian proteomes used here), then probably too

	stringent a blast search or clustering criteria were used for this analysis to be generally useful. This is supported by the fact that clustering the 750K protein sequences resulted in ~250K gene cluster, or about 3 genes per cluster. An alternate interpretation is that a large number of those 15K unique peacock genes are mis-annotations of some kind, and the reason they have no known homology is that they do not represent actual genic sequences. This is supported by the fact that a very low percentage of the annotated peacock genes were found to have Pfam domains (4335 out of 23000 or ~19% of annotated genes with a Pfam domain, see Fig. 1 in Holt and Yandell, 2011).<br>Response: The authors understood the reviewers concerns and addressed the necessary changes in the reviewed manuscript. These have been modified and the new figures have been included. We used CD-Hit to cluster the proteins with 70% similarity, we have tried different similarity cut-offs and below 70% CD-hit showed errors in clustering hence we had to report results at the above mentioned cutoff. CD-Hit clusters the sequences assuming there will be 70% continuous sequence similarity. If there are mutations between the sequences like substitutions, insertions, deletions this will fall outside the cluster. The approach of this method was to work on those proteins that are present in all bird genomes and make a phylogeny on the conserved pool of orthologs. Blast similarity approaches will yield different results but we may end up with shorter orthologs and the results may be completely different. In this article we present CD-Hit based clustering approach to instead of BLAST approach to avoid false positives clustering.<br><br>393-395: This sentence is hard to follow.<br>Response: The authors agree with the reviewers suggestions and hence the sentence is modified.<br><br>398: I don't think you can say that this assembly is "improved" if it is the first published assembly for this species.<br>Response: Your statement of understanding is correct, hence the word improved have been removed from the sentence.<br><br>410-432: The last sentence in this paragraph is missing a period. There should be some analysis of the Kit and FGF proteins that the authors point to here. Are they conserved or divergent from chicken, from guinea fowl? The fact that they are present in the genome isn't surprising or notable, since large number of proteins share homology across large taxonomic distances. The first paragraph here, which discusses sexual selection is too long, and needs to be reduced to one or two sentences to highlight the peacock's historic role in the development of the theory of sexual selection.<br>Response: This paragraph have been removed since the literature talks about some other proteins and we have to investigate more about all these proteins and the transcriptome data will be better to reveal more about the coloration in the peacock bird.<br><br>437: "closeness" is hard to interpret here.<br>Response: This has been modified and made more clear for the readers. Authors want to thank reviewer for their suggestions.<br><br>445: citation for population decline and conservation status of the Indian peafowl population.<br>Response: The citation have been provided in the manuscript.
**Additional Information:**	
**Question**	**Response**
Are you submitting this manuscript to a special series or article collection?	No
**Experimental design and statistics**<br><br>Full details of the experimental design and	Yes

statistical methods used should be given in the Methods section, as detailed in our Minimum Standards Reporting Checklist. Information essential to interpreting the data presented should be made available in the figure legends.<br><br>Have you included all the information requested in your manuscript?	
**Resources**<br><br>A description of all resources used, including antibodies, cell lines, animals and software tools, with enough information to allow them to be uniquely identified, should be included in the Methods section. Authors are strongly encouraged to cite Research Resource Identifiers (RRIDs) for antibodies, model organisms and tools, where possible.<br><br>Have you included the information requested as detailed in our Minimum Standards Reporting Checklist?	Yes
**Availability of data and materials**<br><br>All datasets and code on which the conclusions of the paper rely must be either included in your submission or deposited in publicly available repositories (where available and ethically appropriate), referencing such data using a unique identifier in the references and in the "Availability of Data and Materials" section of your manuscript.<br><br>Have you have met the above requirement as detailed in our Minimum Standards Reporting Checklist?	Yes

Powered by Editorial Manager® and ProduXion Manager® from Aries Systems Corporation

Manuscript
Click here to view linked References
Click here to access/download;Manuscript;To be uploaded in GigaScience_FInal version_30th Sept 2018.docx

De novo genome assembly of the Indian Blue Peacock (*Pavo cristatus*), from Oxford Nanopore and Illumina sequencing

Authors: Ruby Dhar[1], Ashikh Seethy[1], Karthikeyan Pethusamy[1,] Vishwajeet Rohil[2], Sunil Singh[1], Kakali Purkayastha[2], Sandeep Goswami[1], Rakesh Singh[3], Indrani Mukherjee[1,] Ankita Raj[1], Tryambak Srivastava[1], Sovon Acharya[1], Balaji Rajashekhar[4,5,]* and Subhradip Karmakar[1,]*

Affiliation: [1]Department of Biochemistry, AIIMS, New Delhi, India. [2] Vallabhbhai Patel Chest Institute (VPCI), New Delhi, India. [3]Kanpur Zoo, Kanpur, India. [4]Genotypic Technology Pvt. Ltd., Bangalore, India. and [5]Institute of Computer Science, University of Tartu, 50409 Tartu, Estonia

*Corresponding Authors email: balaji@ut.ee, subhradip.k@aiims.edu,

Running Title: *De novo* Genome Assembly of the Peacock Bird

Key words: Peacock, *Pavo cristatus*, Indian National Bird, Genome Assembly, Oxford Nanopore.

Abstract

Background

Pavo cristatus, the Indian blue peacock are geographically found distributed in natural habitats of South Asia. The peacock has been described as one of the most elegant, majestic, and beautiful bird species. Since prehistoric times they have been described in Indian culture and has been adopted as the national bird of India. Its length varies from 92-125 centimeter (without train), weighing about 4-8 Kilograms and lives up to 20 years in the wild. This avian species have been very important in the fields of phylogenetics, developmental studies, sexual reproduction and speciation. The individuals of avian genomics have contributed immensely towards understanding the vertebrate genome evolution. Here we present the first draft genome sequence of *P. cristatus*, yet another important and popular bird species to further add values and gain insight into avian genomics.

Findings

For the first time in avian genomics, Oxford Nanopore technologies (ONT) have been used for the whole genome assembly. Along with the above sequencing technology we have sequenced different DNA insert size libraries from Illumina technology for the peacock DNA. We performed *de novo* genome assembly by integrating the reads from Illumina short insert, long insert, multiple mate-pair reads along with Oxford Nanopore long reads using multiple genome improvement tools. A draft of the peacock genome of about 0.915 Gigabases (Gb) with a N50 of 0.23 Megabases (Mb) was assembled. Annotations with other avian species, protein families, KEGG were performed for functional understanding by insilico approaches. Proteins were compared against Chicken, Turkey and Human to obtain evolutionary similarities and uniqueness of the *Pavo* species.

Conclusions

Our study is the first report of a high quality draft genome of *P. cristatus* using a hybrid assembly generated from Illumina sequencing reads and long reads from ONT. The long read chemistry was found to be useful in addressing challenges related to *de novo* assembly particularly at regions containing repetitive sequences that span longer than the read length and which cannot be resolved using short read based assembly alone. miniION based ONT offers an affordable and reliable platform to achieve this. Observation from our study showed a significant improvement in genome assembly with fewer gaps and a reliable N50 when used together with Illumina reads. Further a comparative genomics with *Gallus gallus* (Chicken) and *Meleagris gallopavo* (Turkey) have shown insights into the gene families and their conserved domains. Peacock proteins were also compared with human proteins to understand the functional components that were conserved after the speciation split. Further, the phylogentic tree on the conserved genes from the avian species showed a grouping amongst the clade of birds based on their ability to fly.

Introduction

Pavo cristatus commonly known as the Indian blue peacock are native to South Asian countries. Due to their popularity as a beautiful bird, they have been introduced into many countries. They are usually found as exhibits in park, zoos and also large number of aviculturists raise and breed these species as pets (Brickle 2002; Jackson 2006). The peacock bird is very popular as it symbolizes beauty, love, grace and pride (Gadagkar 2003; Kushwaha et al. 2016) (Fig. 1). It has been referred in ancient literatures of India and has been found closely associated with the life and culture of the peoples from South East Asia and particularly India (Kadgaonkar 1993). Due to reasons above the peacock obtained the status of National Bird of India in 1963.

The avian genomics began with the sequencing of the model organism the *Gallus gallus* species (Chicken) (Hillier et al. 2004). A decade after *Gallus* sequencing, the avian genome consortium assembled 48 genomes of wide variety of avian species (Zhang et al. 2014). The genome sequencing of different avian species have provided a novel perspective on vertebrate genome evolution and better understanding of the annotation of mammalian genomic regions. The model organism *Gallus* in comparison to human genome have revealed extremely high level of conservations within the orthologous regions (Bejerano et al. 2004), thus promising of being a good candidate for studies of developmental biology, Immunology and vertebrate genome architecture (Burt 2007; Furlong 2005).

Despite the wealth of information from avian genomes sequencing projects, it is very important to genome sequence other new species to add value into aves and vertebrate genomics. For the first time in avian genomics, Oxford Nanopore technology (ONT or Nanopore) has been used to sequence a bird genome presented in this study. The long reads

sequencing will help in improving genome assembly where repeat rich regions challenge the assembly of the genome. Comparative genomics with other birds will help in understanding the uniqueness of peacock genome, development of this species, sexual selection and its evolutionary relationships with other birds. The characterization of the genes and to associate these with function will provide better understanding of the peafowl species. We have unraveled some of the genomic signatures and thus have reported unique gene pools of this bird by performing comparative genomics.

Materials and methods

Sample collection and extraction of DNA

The whole blood of male peacock was collected from Kanpur zoo, India after obtaining the necessary ethical and institutional approval. 20µl of Proteinase K (PK) solution was taken into a 1.5ml micro centrifuge tube. 200µl of blood was added and briefly mixed. 200µl of cell lysis buffer was added to the tube, mixed by vortexing for 10 seconds; incubated at 56°C for 10 minutes. ReliaPrep™ Binding Column was placed into an empty collection tube. 250µl of Binding Buffer (BBA) was added to the tube, and mixed by vortexing for 10 seconds with a vortex mixer. Contents of the tube were added to the ReliaPrep™ binding column, capped and placed in a refrigerated micro centrifuge. These were then centrifuged for 1 minute at maximum speed and flow through was discarded. Binding column was placed into a fresh collection tube. 500µl of column wash solution was added to the column and centrifuged for 3 minutes at maximum speed; Flow through was again discarded. Column washing is repeated thrice. Columns were then placed in a nuclease free clean 1.5ml micro centrifuge tube. 100 µl of Nuclease-Free Water was then added to the column and centrifuged for an additional 1 minute at maximum speed. Column was discarded and elute was saved. The concentration and purity of the extracted DNA was evaluated using Nanodrop

Spectrophotometer (Thermo Scientific) and Qubit flurometer and integrity was checked on a 0.8% agarose gel. The DNA sample was aliquoted for library preparation on two different platforms: Illumina HiSeq4000 and Oxford Nanopore Technologies (ONT).

HiSeq Paired-End library preparation and sequencing

Whole genome sequencing (WGS) libraries were prepared with Illumina-compatible NEXTflex DNA sequencing kit (BIOO Scientific, Austin, Texas, U.S.A.). Approximately 1 μg of genomic DNA was sheared using Covaris S2 sonicator (Covaris, Woburn, Massachusetts, USA) to generate approximate fragment size distribution from 300 to 600 basepair (bp). The fragment size distribution was checked on Agilent 2200 Tape Station with D1000 DNA screen tapes and reagents (Agilent Technologies, Palo Alto, CA, USA) and subsequently purified using HighPrep magnetic beads (Magbio Genomics Inc, USA). The purified fragments were end-repaired, adenylated and ligated to Illumina multiplex barcode adaptors as per NEXTflex DNA sequencing kit protocol (BIOO Scientific, Austin, Texas, USA).

The adapter-ligated DNA was purified with HighPrep beads (MagBio Genomics, Inc, Gaithersburg, Maryland, USA) and then size selected on 2% low melting agarose gel and cleaned using MinElute column (QIAGEN). The resultant fragments were amplified for 10 cycles of PCR using Illumina-compatible primers provided in the NEXTFlex DNA sequencing kit. The final PCR product (sequencing library) was purified with HighPrep beads, followed by library quality control check. The Illumina-compatible sequencing library was initially quantified by Qubit fluorometer (Thermo Fisher Scientific, MA, USA) and its fragment size distribution was analyzed on Agilent TapeStation. Finally, the sequencing library was accurately quantified by quantitative PCR using Kapa Library Quantification Kit

(Kapa Biosystems, Wilmington, MA, USA). The qPCR-quantified library was subjected to sequencing on an Illumina sequencer for 150 bp paired-end chemistry.

The Illumina-compatible sequencing library for the samples has a fragment size range between 275 to 425 bp for Paired-End Short Insert (PE-SI) and 350 bp to 650bp for Paired-End Long Insert (PE-LI). As the combined adapter size is approximately 120bp, the effective user-defined insert size is 155 to 305 bp and 230 to 530 bp for PE-SI and PE-LI respectively. Libraries were sequenced in Illumina HiSeq platform with 150 PE chemistry.

Mate-Pair library preparation and sequencing

Mate Pair sequencing library was prepared with Illumina-compatible Nextera Mate Pair Sample Preparation Kit (Illumina Inc., Austin, TX, U.S.A.). Approximately 4 ug of genomic DNA was simultaneously fragmented and tagged with Mate Pair adapters in a Transposon based Tagmentation step. Tagmented DNA was then purified using AMPure XP Magnetic beads (Beckman Coulter Life Sciences, Indianapolis, IN, U.S.A.) followed by Strand Displacement to fill gaps in the Tagmented DNA. Strand displaced DNA was further purified with AMPure XP beads before size-selecting the 3-5 Kilobases (Kb), 5-7 Kb & 7-10 Kb fragments on low melting agarose gel. The fragments were circularized in an overnight blunt-end intra-molecular ligation step, which will result in circularization of DNA with the insert mate pair adapter junction. The circularized DNA was sheared using Covaris S220 sonicator (Covaris, Woburn, Massachusetts, USA) to generate approximate fragment size distribution from 300 bp to 1000 bp. The sheared DNA was purified to collect the Mate pair junction positive fragments using Dynabeads M-280 Streptavidin Magnetic beads (Thermo Fisher Scientific, Waltham, MA, U.S.A.). The purified fragments were end-repaired, adenylated and ligated to Illumina multiplex barcode adaptors as per Nextera Mate Pair Sample Preparation

Kit protocol.

The adapter-ligated DNA was then amplified for 15 cycles of PCR using Illumina-compatible primers. The final PCR product (sequencing library) was purified with AMPure XP beads, followed by library quality control check. The Illumina compatible sequencing library was initially quantified by Qubit fluorometer (Thermo Fisher Scientific, MA, USA) and its fragment size distribution was analyzed on Agilent TapeStation. Finally, the sequencing library was accurately quantified by quantitative PCR using Kapa Library Quantification Kit (Kapa Biosystems, Wilmington, MA, USA). The qPCR quantified libraries were pooled in equimolar amounts to create a final multiplexed library pool for sequencing on an Illumina sequencer.

Oxford Nanopore MinION library preparation and sequencing

Genomic DNA (1.5µg) was end-repaired (NEBnext ultra II end repair kit, New England Biolabs, MA, USA), cleaned up with 1x AmPure beads (Beckmann Coulter,USA). Adapter ligation were performed for 20 minutes using NEB blunt/ TA ligase (New England Biolabs, MA, USA). Library mix were cleaned up using 0.4X AmPure beads (Beckmann Coulter, USA) and eluted in 25 µl of elution buffer. Eluted Library were used for sequencing. Whole genome library were prepared by using ligation sequencing SQK-LSK108 Oxford Nanopore sequencing kit (ONT, Oxford, UK). Sequencing were performed on MinION Mk1b (ONT, Oxford, UK) using SpotON flow cell (FLO-MIN106) in a 48hr sequencing protocol on MinKNOW (1.1.20 from ONT).

Illumina raw data quality control and processing

The Illumina reads were de-multiplexed using Illumina bcl2fastq. The Illumina generated raw data for genomic libraries was quality checked using FastQC (http://www.bioinformatics.babraham.ac.uk/projects/fastqc/) (Andrews, S., 2010). The paired-end Illumina reads were processed for clipping the adapter and low-quality bases using customized script which retains minimum 70% bases/reads with Phred score (Q≥30 in each base position) with a read length of 50 bp. The MP libraries were trimmed for adapter and low-quality base trimming from the 3'-end using PLATANUS internal trimmer (http://platanus.bio.titech.ac.jp/) (Kajitani et al. 2014).

Oxford Nanopore reads base calling and processing

The raw data were then base-called with the cloud-based Metrichor workflow 2D Basecalling plus Barcoding by Metrichor (V.2.43.1 from ONT, https://nanoporetech.com/products/metrichor). The Oxford Nanopore reads were processed using Poretools (Loman et al. 2014) for converting fast5 files to fasta format. For further quantification and analysis the 2D reads or 1D high quality reads were selected for further assembly.

***De novo* genome assembly and genome size estimation**

The quality checked Oxford Nanopore reads were error-corrected using Illumina PE reads. For error-correction the Illumina PE-reads were aligned to the Nanopore reads by using BWA aligner (Li et al. 2009). The paired-end reads were assembled using Abyss (Birol.et al. 2009) followed by contig extension using Oxford Nanopore reads using SSPACE-LongRead

(Boetzer et al. 2014). Super scaffolding of the assembled scaffold was performed using SSPACE (Boetzer et al. 2010) and PLATANUS (http://platanus.bio.titech.ac.jp/) using the Oxford Nanopore and Matepair data. Final draft genome resulted after gap closure by GAPCLOSER (http://sourceforge.net/projects/soapdenovo2/files/GapCloser/) and PLATANUS gap_close tool (http://platanus.bio.titech.ac.jp/) using Illumina data. The genome size was estimated using a k-mer distribution plot using JELLYFISH (Marcais et al. 2011). The assembly and annotation workflow has been represented in Figure 2.

Identification of repetitive elements and SSR markers

Repetitive elements, retrotransposons and DNA transposons were identified in the draft genome and was hardmasked by using reference genomic repeats of *G. gallus* using Repeatmasker tool (www.repeatmasker.org/). Final assembled scaffolds were analysed for Simple Sequence Repeats (SSR) identification. SSRs like the di, tri, tetra, penta and hexa-nucleotide repeats in the genome were obtained using MISA (Version 1.0.0) (http://pgrc.ipk-gatersleben.de/misa/).

Annotation of the draft genome

Gene models were predicted on a hard masked draft genome and further genes were predicted using AUGUSTUS (http://augustus.gobics.de/) with *G. gallus* (red junglefowl the chicken) as a reference model. The predicted proteins were annotated by using BLASTP (Altshul et al. 1990) against the NCBI NR (non-redundant) database with default parameters at E-value cutoff of 1E-5.

The predicted proteins were searched against the KEGG-KAAS server (http://www.genome.jp/tools/kaas/) for pathway analysis (Moriya et al. 2007). *G. gallus*, *M. gallopavo* (turkey), *Taeniopygia guttata* (zebra finch), *Falco peregrinus* (peregrine falcon)

were used as reference organism for pathway identification. The EuKaryotic Orthologous Groups (KOGs) (https://genome.jgi.doe.gov/portal/help/kogbrowser.jsf) were predicted using homology based approach.

Prediction of protein domains

Predicted proteins from Peacock, Chicken and Turkey with sequence length greater than 100 amino acids were considered for protein domain analysis. All the protein sequences from each organism were searched against Pfam-A database (http://pfam.sanger.ac.uk/) using Pfam scan (https://www.ebi.ac.uk/seqdb/confluence/display/THD/PfamScan) for protein domain identification.

Identification of avian protein families

A total of 748,544 protein sequences from 49 avian species (including peacock proteins from this study) and others were downloaded from http://avian.genomics.cn/en/jsp/database.shtml. Sequences greater than 100 amino acids from all the avian genomes were selected and concatenated to a single fasta file. These sequences were clustered using CD-HIT (Fu et al. 2012) with 70% alignment coverage for the shorter sequence with a length difference cutoff of 0.7. The single copy ortholog gene family present across all organisms and genes unique to peacock were filtered and annotated.

Phylogenetic tree construction

Gene clusters containing proteins in all the avian species were selected for phylogentic analysis. These protein sequences from each species were concatenated and were aligned by multiple sequence alignment tool Clustalw (http://www.clustal.org/clustal2). The poorly aligned positions and divergent regions were removed using Gblock tool

(http://molevol.cmima.csic.es/castresana/Gblocks.html). The fasta format sequences were converted to phylip format using Phylip tool (http://evolution.genetics.washington.edu/phylip/getme-new1.html). Phylogenetic trees were constructed using IQ-TREE version 1.5.6 (www.iqtree.org). The parameters used for phylogenetic tree construction were ultrafast boostrap (UFBoot, using the –bb option of 1000 replicates), and a standard substitution model (-st AA –m TEST) and alrt 1000 -nt AUTO was given for tree generation. The generated trees from IQ-TREE tool were visualized using Figtree (http://tree.bio.ed.ac.uk/software/figtree/) and the Brach-support values were recorded from the output ".treefile". The trees were modified for better visualization under Trees section increasing order nodes were applied.

Genome conservation analysis

Draft chromosome visualizations were constructed by aligning the assembled peacock genome against the *G. gallus* with the Chromosomer tool (https://github.com/gtamazian/chromosomer). The reordered assembled genome was aligned against the Chicken genome using LAST aligner (http://last.cbrc.jp/) with NEAR (finding short-and-strong (near-identical) similarities.) parameter allowing for substitution and gap frequencies leading to the identification of orthologs. These query-mapped regions were filtered with a greater than 1% of the maximum length for visualization using Circos (http://circos.ca/).

Results

Genome sequencing assessment

A total of five libraries from Illumina HiSeq technology of 150 bp paired-end were generated. The short-insert reads of 489,114,747 accounted to genome coverage of 146.7X

and long-insert reads of 302,884,819 sequences was about 90.9X coverage with a total coverage of 236X. Sequencing of three mate-pairs of 3-5Kb, 5-7Kb of and 7-10Kb yielded 72,915,033, 47,440,144 and 36,464,628 reads respectively with an approximate coverage of 21.9X, 14.2X and 10.9X respectively, with a grand total of 156 million mate-pair reads of 47X coverage. Oxford Nanopore technology was used to generate 366,323 long reads having of 2,398,560,283 bp with coverage of 2.3X. The complete genome sequencing was generated to a depth of ~287X from both Illumina and Oxford Nanopore platforms. The coverage was based on assuming the peacock genome size of about 1 Gb (Table S1).

Genome assembly

The first assembly was performed on Illumina reads with Abyss *de novo* assembler that resulted in ~932 Mb (mega base) of genome with an N50 of 1639 bp. The extension of the contigs were performed with Oxford Nanopore reads which generated scaffolds with N50 of 14,748 bp. Super scaffolding of the assembled scaffold was performed using SSPACE and PLATANUS with MP libraries that generated ~916 Mb genome with the N50 value of 168,140bp. The final gap closer was executed using GAPCLOSER program with MP and PE-LI libraries which generated a draft genome of 1.02 GB (giga base). The draft genome assembly of *Pavo cristatus* consists of 179,346bp scaffolds, with a N50 of 189,886bp with 37 scaffolds having sequence length >=1Mbp. Contigs above 5000 bp have covered a genome of ~0.915 Mb with N50 0.23 Mb. In the assembled genome there were ~0.4% of non-ATGC characters (Table 1).

Repetitive genome elements and SSR markers

A total of 75,315,566 bp (7.33%) of the peacock genome was estimated to consist of repeat sequences (Table S2a). In the genome about 56,511,635 bp (5.5%) of retrotransposons (class

I) were identified as the NON-LTR elements (LINEs (4.7%), SINEs (0.08%)) and LTR elements (0.72%). Then the DNA transposons (class II) of 7,277,390 bp (0.71%) and unclassified elements of about 467,719 (0.05%) were identified (Table S2A). Other avian birds have shown the median percentages of LINEs, SINEs, LTR, DNA, Unknown and total masked bases were of 3.94, 0.11, 1.31, 0.22, 0.85 and 6.93 respectively (Table S2B).

A total of 399,493 SSRs were obtained from the peacock genome assembly. The largest fraction of SSRs identified were mono-nucleotide (60.04%), followed by tetra-nucleotide (26%), di-nucleotide (8.51%), tri-nucleotide (4.31%), penta-nucleotide (1.03%) and finally hexa-nucleotide (0.13%). Among the SSRs identified, A (49.2%) and T (44.9%) accounted for 94.1% of the mono-nucleotide repeats. AT (23.8%), TA (16.5%), TG (13.7%), AC (10.6%) and CA (10.32%) accounted for 75% of the di-nucleotide repeats. while TTG (9.9%), AAT (9.6%), AAC (9.4%), TTA (7.1%), ATT (4.5%), TAA (3.5%), CAA (3.1%) and GGA (2.69%) accounted for 49.7% of the tri-nucleotide repeats (Table S3).

Gene prediction and annotation

A total of 23,153 proteins were predicted from the assembled draft genome using AUGUSTUS. Among them 21,854 (94.4%) predicted proteins showed homology to other sequences from the NCBI NR database (Fig. 3). The top three organisms where the peacock proteins showed homology belonged to the *G. gallus* with 11,398 proteins, *M. gallopavo* with 4059 proteins, *Amazona aestiva* (Blue-fronted Amazon parrot) with 1352 proteins and *Anas platyrhynchos* (Mallard) with 849 proteins. The detail annotations of all the proteins are available in Table S4.

Significant gene Ontology (GO) descriptions were assigned for 18,294 (79%) proteins.

Among them, 14,489 proteins have Molecular Function; 11,678 have Biological Process and 13,735 proteins have Cellular Component as functional categories (Table S4 and Fig. S3). About 4091 (17.7%) of unique proteins were found to have pathway information from the KEGG database (Table S5). Proteins searched against the KOG annotations showed a total of 20,937 proteins having annotations (Table S6). Against the human proteins the peacock proteins showed expansions in ontologies for cell morphogenesis, neuronal projection and development and GTPases (Table S7 and Fig. S4).

Analysis of avian protein families

A total of 748,544 protein sequences from 49 avian species have 653,497 protein sequences of length above 100 amino acids (Table S8A). A total of 114,121 gene clusters were generated of which 68 gene clusters had single copy orthologs present in all the 49 avian species (Table S8B and Table S8C). With the stringent cutoff 13,860 clusters unique to peacock species were observed (Table S8D).

Phylogenetic analysis

The phylogenetic analysis of 48 avian species along with peacock genome showed clustering of the *P. cristatus* species in a clade of *G. gallus* (chicken), *M. gallopavo* (turkey), *A. platyrhynchos* (mallard the duck), *Tinamus guttatus* (white-throated tinamou) and *Struthio camelus* (ostrich). This is the largest clade with six species of having a bootstrap support of a 100. In the aforementioned clade leaving the mallard species all belong to flightless or low flying birds. The bootstrap support between *P. cristatus* and *G. gallus* were 96, followed by *M. gallopavo* of 100 bootstrap support (Fig. 4).

Comparison with other species

Predicted proteins from peacock, chicken and turkey when searched for the conserved Pfam protein domains showed about 81% of the domains that were common among these three species (Fig. 5, Table S9). In comparison with the total unique Pfam domains from all the three species, 94%, 98.4% and 99.7% Pfam domains were present in peacock, chicken and turkey respectively. There were 255, 69 and 14 Pfam domains unique in the aforementioned species respectively (Table S9H).

There are 78% (15470), 85% (12794) and 85% (11745) of the peacock, chicken and turkey proteins respectively found to contain Pfam domains (Table S9). The assembled peacock genome when reordered for pseudo chromosomes generation against the masked 1.21GB chicken genome (Warren et al. 2016) showed a 597MB reordered peacock genome (Fig. 6).

Conclusions

Using a combination of short reads of different insert sizes as well as mate pair reads generated from Illumina technology along with long reads from Oxford Nanopore, we obtained a draft genome of the Indian Blue Peacock. In comparison with other avian genomes (Zhang et al. 2014), the current 290X sequencing depth obtained from our study is one of the highest. The draft genome assembly generated have an N50 of 0.23MB. The inclusion of Oxford Nanopore reads for scaffolding followed by subsequent gap-closing using Illumina sequencing data led to a 26.2% reduction in the number of scaffolds and about 50.7% and 115% increase in the scaffold and contig N50 statistics, respectively. On the contrary, the assembly contained less than 0.4% of unknown nucleotides, which is very low for a draft assembly. Thus with 2.3X coverage of Oxford Nanopore reads, a significant improvement in the assembly was observed. Thus we have shown how the low-cost third generation sequencing data from Oxford Nanopore was used for the first time in avian genomics for de novo assembly and have yielded substantiality improved the final draft genome. This will

further benefit in understanding the organisms in the structurally complex regions having repeat elements and isoforms in the genome (Goodwin et al. 2016).

Comparisons of the genome features of Peacock with other species and databases have shown about 95% homology (Fig. 7). With an enhancement in the sequencing coverage from long reads based platforms with transcriptomic sequencing aided by scaffolding and/or gap closure tools, further improvement in the assembly can be achieved. These improvements in the genome with help to understand the role of these unique proteins and other features that truly makes this bird unique. The genome sequence also gives insights on its genetic lineage and evolution with relation to other avian members. The estimated median divergence time of *P. cristatus* from *G. gallus* is of about 35 million years ago (MYA) while between *G. gallus* and and *M. gallopavo* is about 37 MYA (http://www.timetree.org/). The huge gap is due to non-availability of genome sequences from other avians, which can be reduced by sequencing other avian species. Several hypothesis and evolutionary theories with respect to sexual selection, population genetics, developmental biology or immunology can be better understood with the help of other avian genome sequencing. Among the vertebrates, it has been observed that the variations in TEs among avians are very low (Sotero-Caio et al. 2017) (Table S8). The genome complexities of a species are influenced by the Transposable elements (TE) that are believed to play a crucial role (Kapsuta et al. 2017). In this peacock genome assembly inclusion of Oxford Nanopore sequencing have significantly improved the assembly thus helping in resolving the repetitive regions in genome quality and assembly. Homology searches have shown several important gene family expansions such as Kinases, Zn finger proteins, GTPases and others (Fig. 8). Their roles in biology, development and evolution of the peacocks need to be further explored.

One of the most important task will be to characterize the genes involved in the coloration of the tail feather plumage in *P. cristatus* (Roulin et al. 2013). The peacock feathers have played a significant role in the mating and sexual selection. Peacock seems to defy the Darwinian laws of natural selection. These concern were raised by no other than Darwin himself. Hence, he proposed the theory of the sexual selection where the female can choose for a male with a certain phenotypic feature such as brilliant color or a long tail (Burgess 2001). Peacock's brilliantly colored long tail feathers seems to evolve at the cost of finding its female partner thereby contributing its beneficial genes, even at the cost of making itself vulnerable to predators. A female peafowl in turn tends to choose the mate with the largest and decorated plumage, which indirectly reflects its healthiness and capacity to wade off potential competitors. Thus understanding the formation of beautiful feathers from the genomic context will help in resolving several evolutionary theories on sexual selection that have been discussed on this species.

The genome information can be valued and explored by avian enthusiasts to further understand about the peacock Though not critically endangered yet, in India, peafowl population is surely at a declining trend in the wild due to massive deforestation and habitat loss (Ramesh et al. 2009). These are further compounded by increased poaching for meat and feathers of peacock bird. Our genome sequencing initiative of *Pavo cristatus* is not just only from a conservational viewpoint, but also to preserve a heritage associated with this bird that runs through centuries and that bears a strong attachment to the national psyche.

Availability of supporting data

Supplementary data contains, read statistics, annotation, repeats identification, orthology analysis, assembly and annotation. Figures, Gene ontology and annotations. Additional data will be available from https://biit.cs.ut.ee/supplementary/peacock/

Raw Data and genome assembly in SRA

Raw reads (Illumina and Oxford Nanopore) are available in the Sequence Read Archive (SRA), and the Whole Genome Shotgun project has been deposited at GenBank under SRA Submission ID: SUB3108024, Bioproject: PRJNA413288 and Biosamples SUB3108018/SAMN07739105 : SKPea2016_SI, SUB3108017/SAMN07739104 : SKPea2016_LI, SUB3107930/SAMN07739101 : FPL_3_5KB, SUB3108015/SAMN07739102 : FPL_5_7KB, SUB3108016/SAMN07739103 : FPL_7_10KB and SUB3108020/SAMN07739107 : FPL_Nano. The *de novo* genome assembly can be accessed under SUB4504869/ SAMN07739105.

Competing interests

The author(s) declare that they have no competing interests.

Authors contributions

RD, AS, KP performed wet lab experiments; RD designed work plan, experiments and logistics; SS, VR, KP SG IM and AR assisted with the work; RS provided samples from bird; BR, SK performed data analysis and interpretation; BR, SK drafted the manuscript and SK overseen the whole project.

Acknowledgements

Department of Biochemistry, AIIMS, New Delhi for providing space and infrastructure to carry on the work. RD for providing partial funding. Raja, Sudha and their team Deepti, Shalini, Nagu, Abhi and Manish from Genotypic Technology Pvt. Ltd for providing sequencing services. BR acknowledges IUT 34-4.

Tables

Table 1. *De novo* assembly statistics of the peacock genome.

Description	Contigs	Nanopore Scaffold	Super Scaffolds	GapClosed	>1000 Kb	>5000 Kb
Contigs	685,241	281,272	179,346	179,332	34,178	15,025
Maximum Length	49,159	251,510	2,390,121	2,488,982	2,488,982	2,488,982
Minimum Length	300	5	265	265	1000	5000
Average Length	1360	3250	5111	5729	-	-
Total Length	932,162,464	914,363,908	916,720,956	1,027,510,962	954,449,349	915,342,012
Length >= 100 bp	685,241	281,271	179,346	179,332	34,178	15,025
Length >= 200 bp	685,241	281,271	179,346	179,332	34,178	15,025
Length >= 500 bp	616,120	186,433	93,727	93,718	34,178	15,025
Length >= 1 Kbp	363,428	104,479	34,168	34,178	34,178	15,025
Length >= 10 Kbp	1591	24,748	9249	10,310	10,310	10,310
Length >= 1 Mbp	0	0	27	37	37	37
Non-ATGC #	350,325	42,696,911	49,169,831	4,043,129	4,040,790	3,986,487
Non-ATGC %	0.038	4.67	5.36	0.393	0.423	0.436
N50 value	1639	14,748	168,140	190,304	218,023	232,312

Figure legend

Figure 1. The beautiful and charismatic photo of Indian blue peacock (*Pavo cristatus)* bird.

Figure 2. Detailed workflow for *de novo* whole genome assembly and annotation.

Figure 3. Peacock proteins showing homology**.** Pie chart showing significant similarity scores of peacock proteins against the NR database.

Figure 4. Phylogenetic tree generated from homologous proteins from 49 different avian species. Birds on dotted line are low flying or non-flying birds. Solid line represents flying birds.

Figure 5. Venn diagram showing common and unique Protein family domains (Pfam) between Peacock, Chicken and Turkey proteins.

Figure 6. Circular image of the assembled peacock genome aligned against the *G. gallus* genome using Chromosomer tool. Draft chromosomes were generated by similarity between scaffolds that were arranged on the reference chicken genome. Circos was used for visualization. The right side of the image represents the reference chicken genome and left side of the image represents the Peacock genome.

Figure 7. Venn diagram showing peacock proteins showing significant homology to NR database, KOG, Pfam and GO ontologies.

Figure 8. Heatmap showing protein family (Pfam) distributed in peacock, chicken or turkey species where each row contains maximum of 50 Pfam domains.

References:

1. Brickle, N. 2002. Habitat use, predicted distribution and conservation of green peafowl (*Pavo muticus*) in Dak Lak Province, Vietnam. Biological Conservation, 105: 189-197.

2. Jackson, C. 2006. Peacock. London: Reaktion Books Ltd.

3. Gadagkar, R., 2003. Is the peacock merely beautiful or also honest?. Current Science, 85(7), pp.1012-1020.

4. Kushwaha, S., and Kumar, A. 2016. A Review on Indian Peafowl (*Pavo cristatus*) Linnaeus, 1758. Journal of Wildlife and Research, 4, 42-59.

5. Kadgaonkar, Shivendra B. 1993. The peacock in ancient Indian art and literature. Bulletin of the Deccan College Research Institute, vol. 53, pp. 95–115. JSTOR, www.jstor.org/stable/42936434.

6. Hillier LW, Miller W, Birney E, Warren W, International Chicken Genome Sequencing Consortium et al. 2004 Sequence and comparative analysis of the chicken genome provide unique perspectives on vertebrate evolution. Nature 432:695–716

7. Zhang, G., Jarvis, E. D., and Gilbert, M. T. P. 2014. A flock of genomes. Science 346, 1308–1309.

8. Bejerano, G., Pheasant, M., Makunin, I., Stephen, S., Kent, W.J., Mattick, J.S. and Haussler, D., 2004. Ultraconserved elements in the human genome. Science, 304(5675), pp.1321-1325.

9. Furlong, R.F., 2005. Insights into vertebrate evolution from the chicken genome sequence. Genome biology, 6(2), p.207.

10. Kajitani, R., Toshimoto, K., Noguchi, H., Toyoda, A., Ogura, Y., Okuno, M., Yabana, M., Harada, M., Nagayasu, E., Maruyama, H. and Kohara, Y., 2014. Efficient de novo assembly of highly heterozygous genomes from whole-genome shotgun short reads. Genome research, 24(8), pp.1384-1395.

11. Loman, N. J. and Quinlan, A. R. 2014. Poretools: a toolkit for analyzing nanopore sequence data. Bioinformatics, 30(23), 3399-3401.

12. Li H. and Durbin R. 2009. Fast and accurate short read alignment with Burrows-Wheeler Transform. Bioinformatics, 25:1754-60.

13. Birol, I., Jackman, S.D., Nielsen, C.B., Qian, J.Q., Varhol, R., Stazyk, G., Morin, R.D., Zhao, Y., Hirst, M., Schein, J.E. and Horsman, D.E., 2009. De novo transcriptome assembly with ABySS. Bioinformatics, 25(21), pp.2872-2877.

14. Boetzer, Marten, and Walter Pirovano. 2014. SSPACE-LongRead: scaffolding bacterial draft genomes using long read sequence information. BMC bioinformatics 15.1: 211

15. Boetzer, M., Henkel, C.V., Jansen, H.J., Butler, D. and Pirovano, W., 2010. Scaffolding pre-assembled contigs using SSPACE. Bioinformatics, 27(4), pp.578-579.

16. Marcais, G and Kingsford, C. 2011. A fast, lock-free approach for efficient parallel counting of occurrences of k-mers. Bioinformatics 27(6): 764-770.

17. Altschul, S.F., Gish, W., Miller, W., Myers, E.W. and Lipman, D.J., 1990. Basic local alignment search tool. Journal of molecular biology, 215(3), pp.403-410.

18. Moriya, Y., Itoh, M., Okuda, S., Yoshizawa, A.C. and Kanehisa, M., 2007. KAAS: an automatic genome annotation and pathway reconstruction server. Nucleic acids research, 35(suppl_2), pp.W182-W185.

19. Fu, L., Niu, B., Zhu, Z., Wu, S. and Li, W., 2012. CD-HIT: accelerated for clustering the next-generation sequencing data. Bioinformatics, 28(23), pp.3150-3152.

20. Warren, W.C., Hillier, L.W., Tomlinson, C., Minx, P., Kremitzki, M., Graves, T., Markovic, C., Bouk, N., Pruitt, K.D., Thibaud-Nissen, F. and Schneider, V., 2016. A new chicken genome assembly provides insight into avian genome structure. G3: Genes, Genomes, Genetics, pp.g3-116.

21. Zhang, G., Li, C., Li, Q., Li, B., Larkin, D.M., Lee, C., Storz, J.F., Antunes, A., Greenwold, M.J., Meredith, R.W. and Ödeen, A., 2014. Comparative genomics reveals insights into avian genome evolution and adaptation. Science, 346(6215), pp.1311-1320.

22. Goodwin, S., McPherson, J.D. and McCombie, W.R., 2016. Coming of age: ten years of next-generation sequencing technologies. Nature Reviews Genetics, 17(6), p.333.

23. Sotero-Caio, C.G., Platt, R.N., Suh, A. and Ray, D.A., 2017. Evolution and diversity of transposable elements in vertebrate genomes. Genome biology and evolution, 9(1), pp.161-177.

24. Kapusta, A. and Suh, A., 2017. Evolution of bird genomes—a transposon's- eye view. Annals of the New York Academy of Sciences, 1389(1), pp.164-185.

25. Roulin, A. and Ducrest, A.L., 2013, June. Genetics of colouration in birds. In Seminars in cell & developmental biology (Vol. 24, No. 6-7, pp. 594-608). Academic Press.

26. Ramesh, K. and McGowan, P., 2009. On the current status of Indian peafowl Pavo cristatus (Aves: Galliformes: Phasianidae): keeping the common species common. Journal of Threatened Taxa, 1(2), pp.106-108.

Figure 1

Click here to access/download;Figure;Fig 1. Peacock bird.jpg

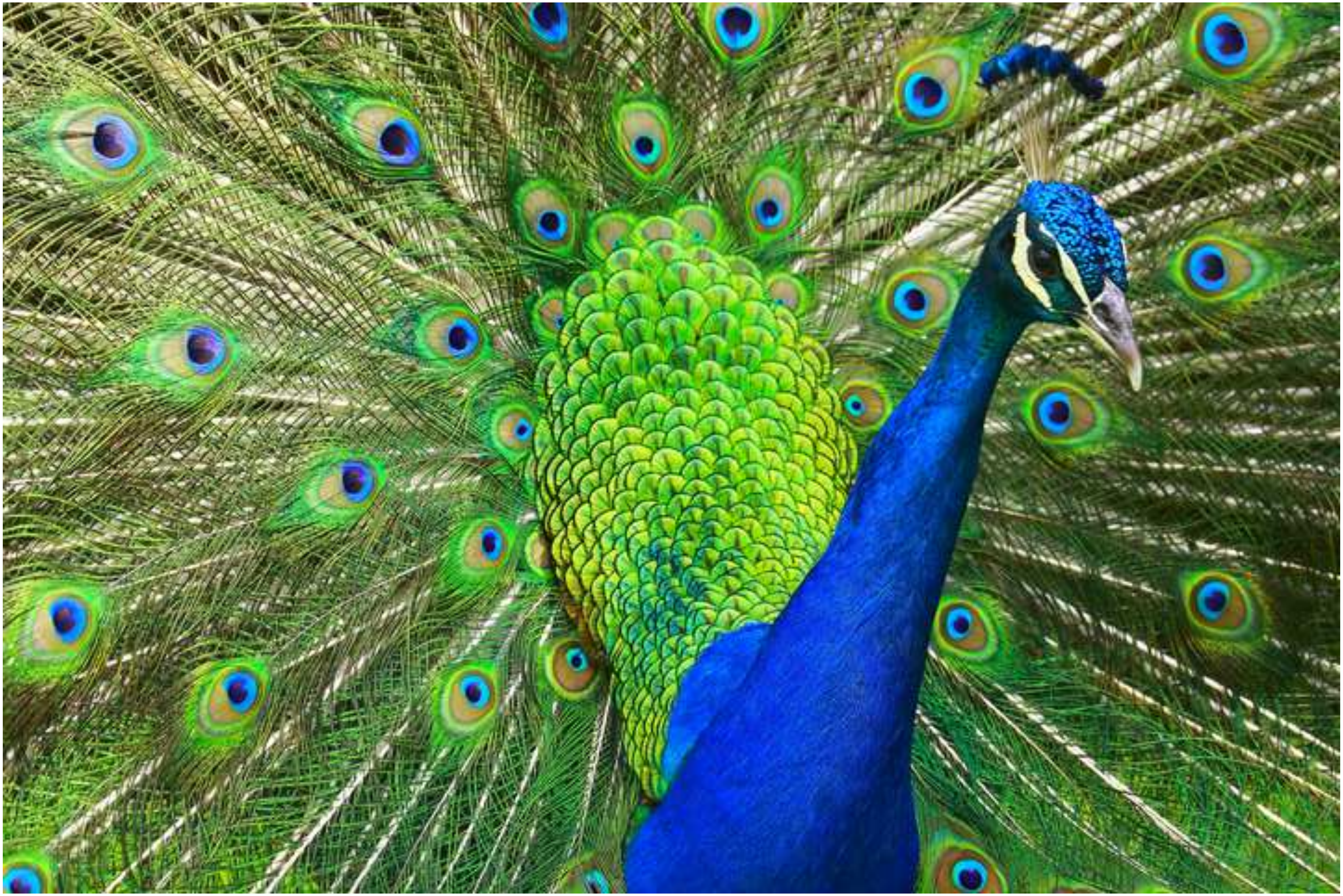

Figure 2

Click here to access/download;Figure;Fig 2. Workflow.jpeg

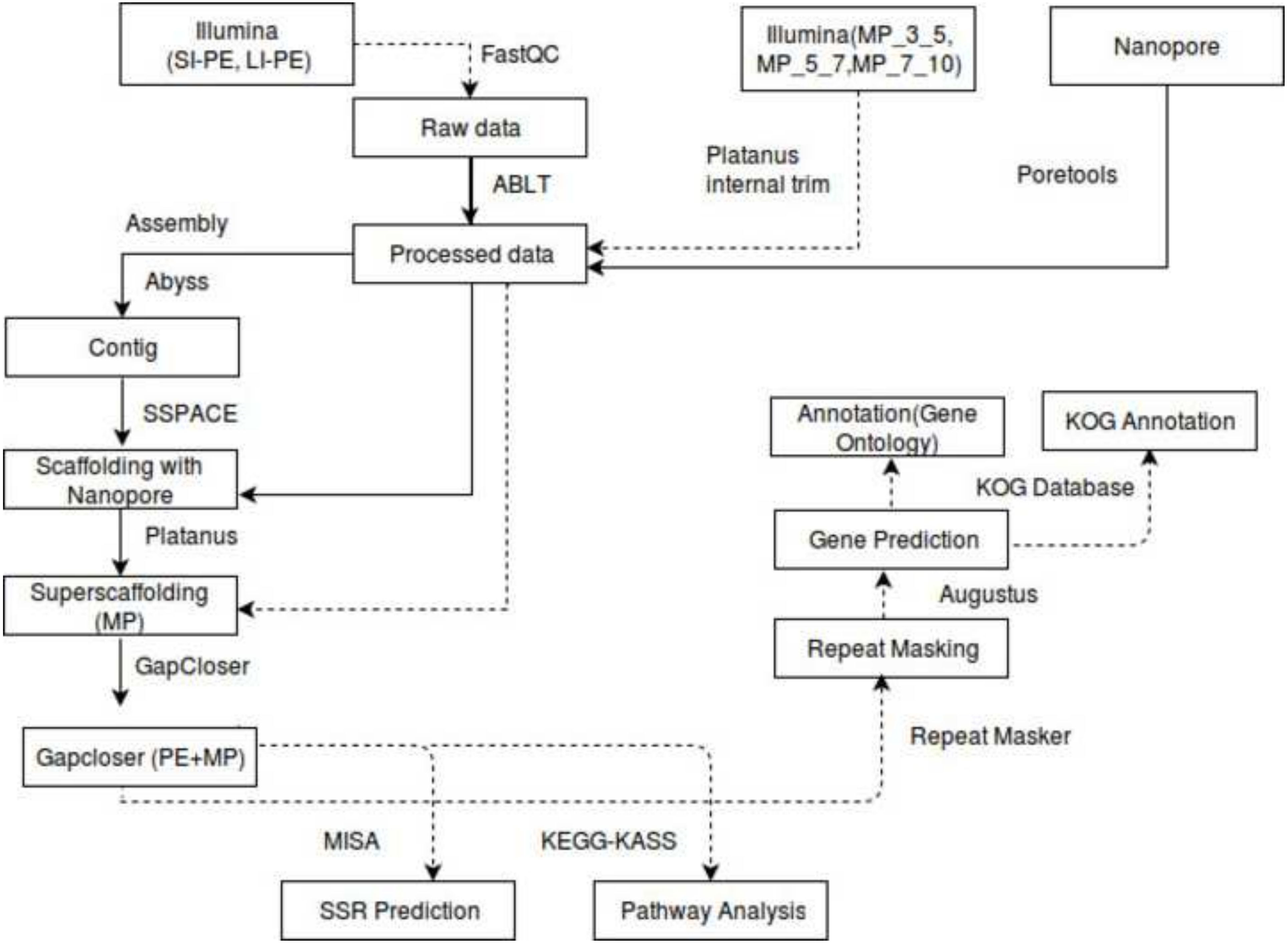

Figure 3

Click here to access/download;Figure;Fig 3 Similarity scores against the Uniprot database.pdf

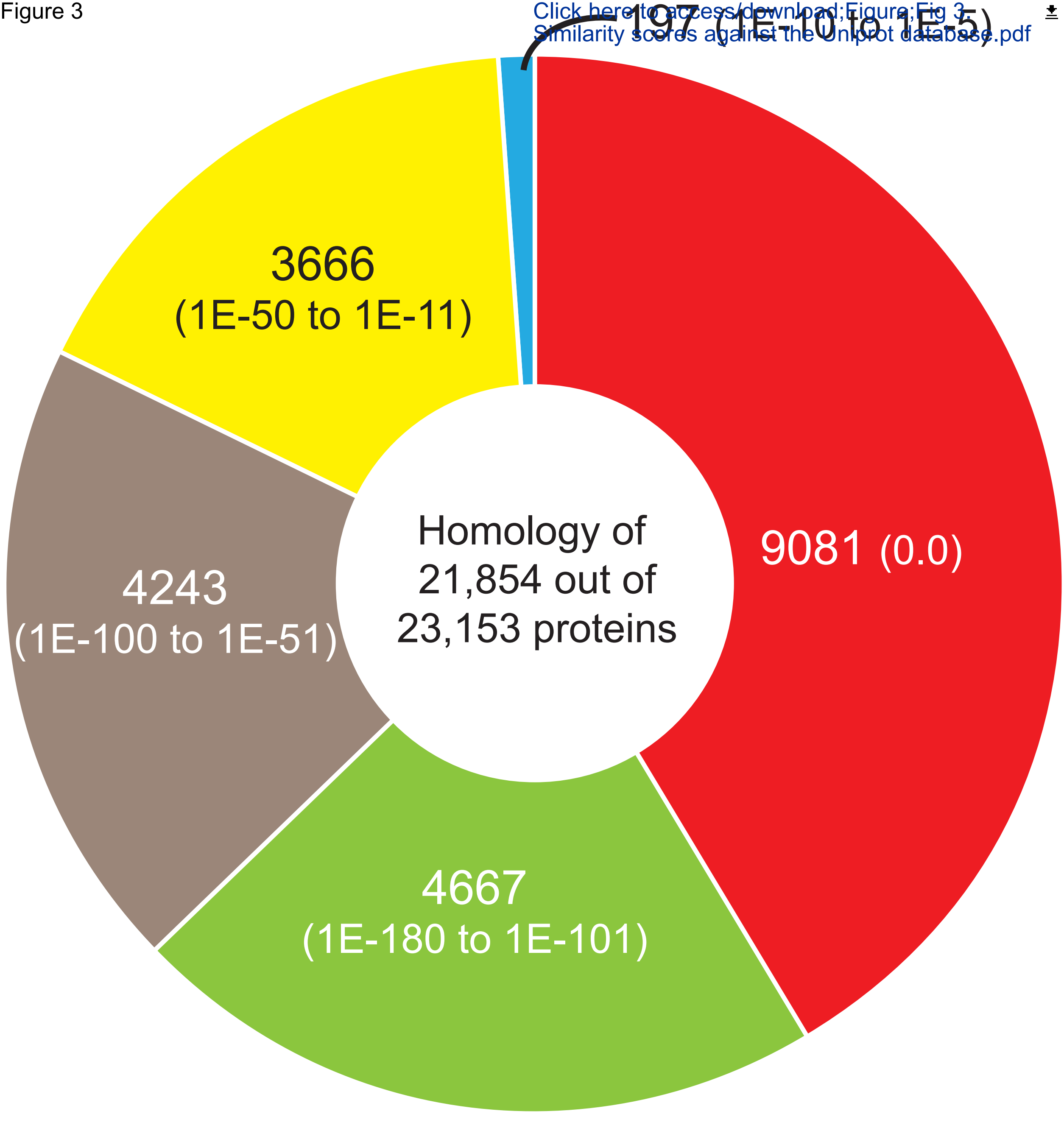

Figure 4

Click here to access/download;Figure;Fig 4. Phylogeny of avian species.pdf

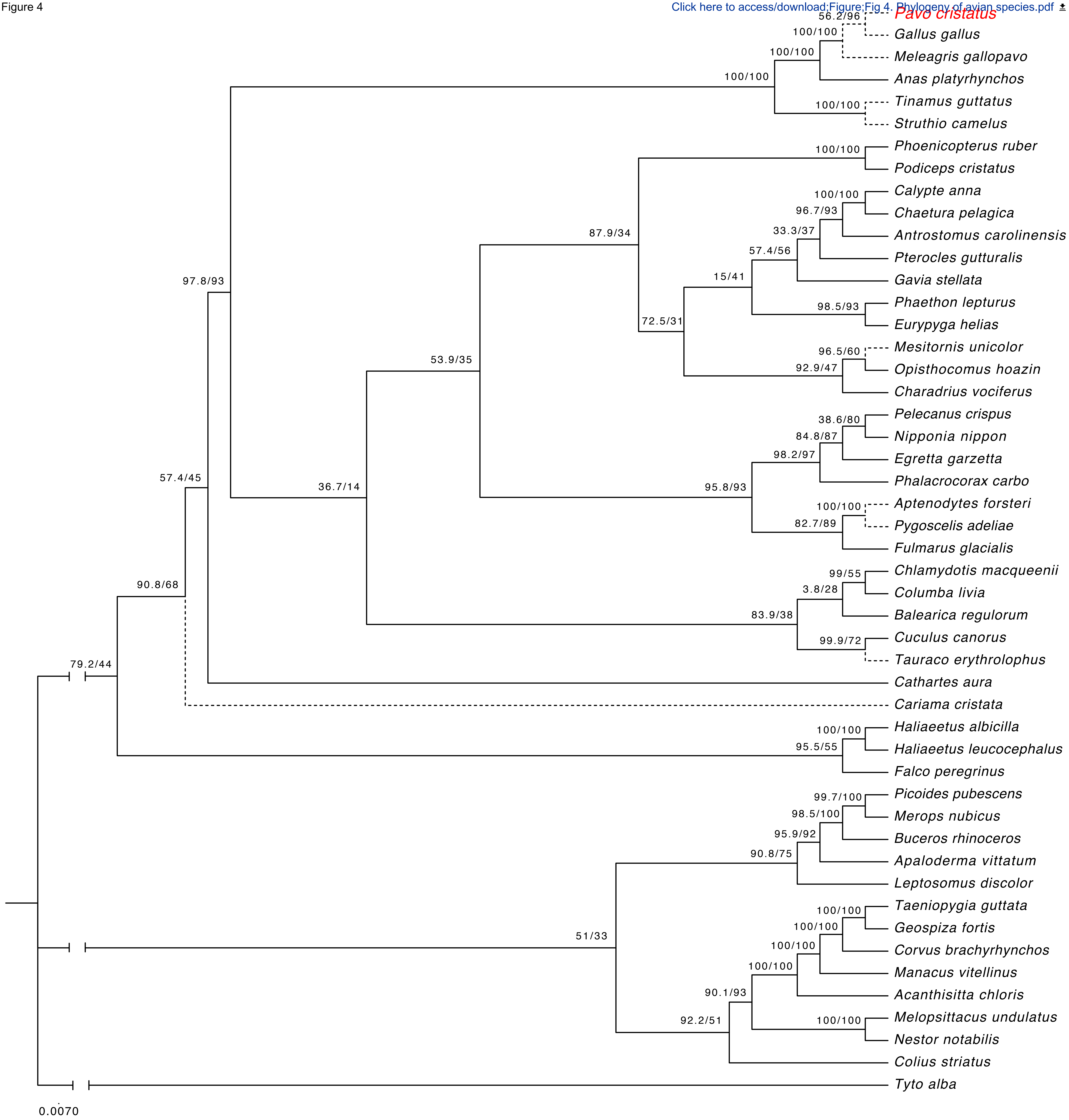

Figure 5

Click here to access/download;Figure;Fig 5. Unique Pfam domains common with chicken

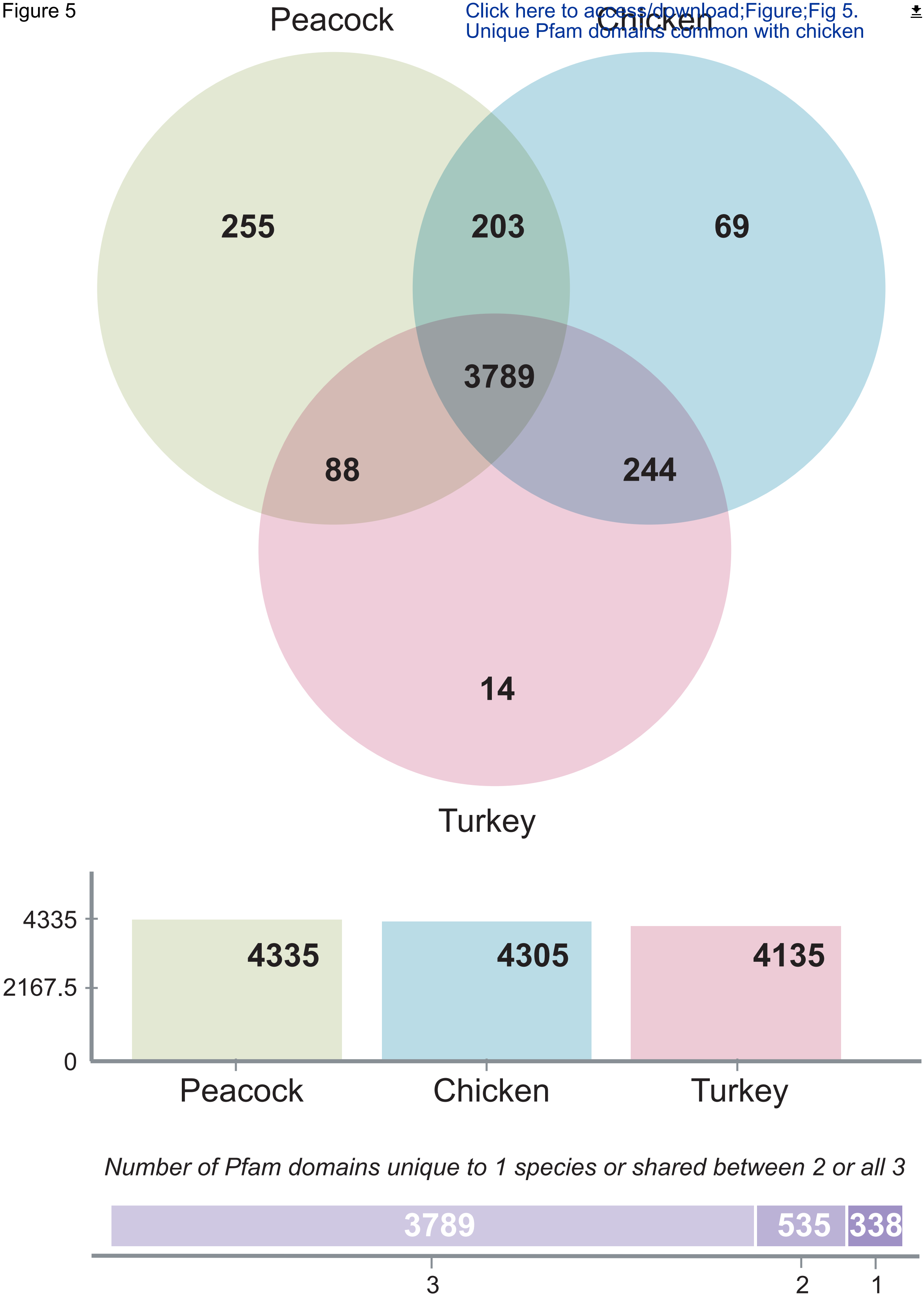

Figure 6

Click here to access/download;Figure;Fig 6. Peacock scaffolds against Gallus circular synteny.png

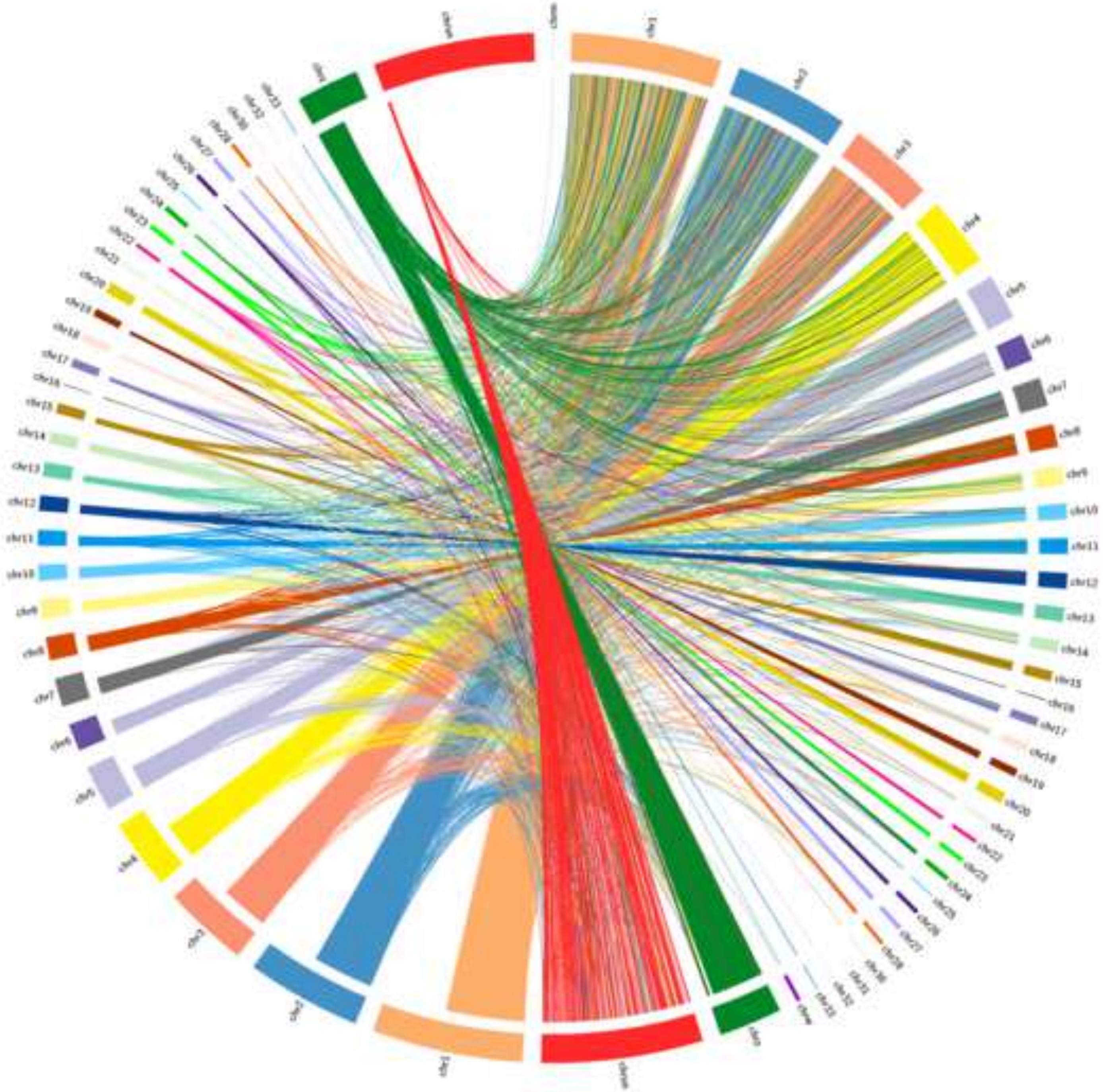

Figure 7

Click here to access/download;Figure;Fig 7. Venn of annotations.pdf

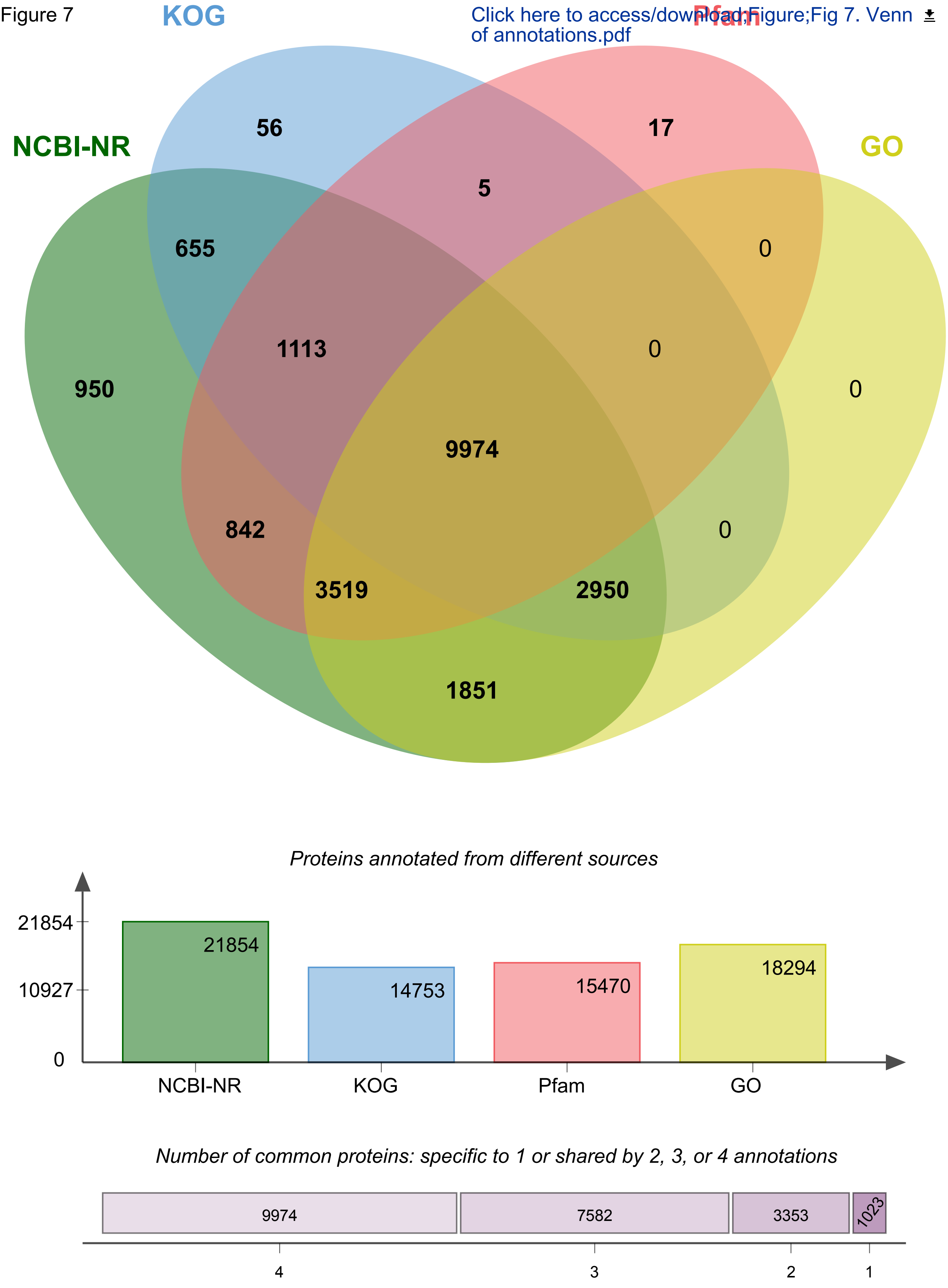

Figure 8

Click here to access/download;Figure;Fig 8. Heatmap

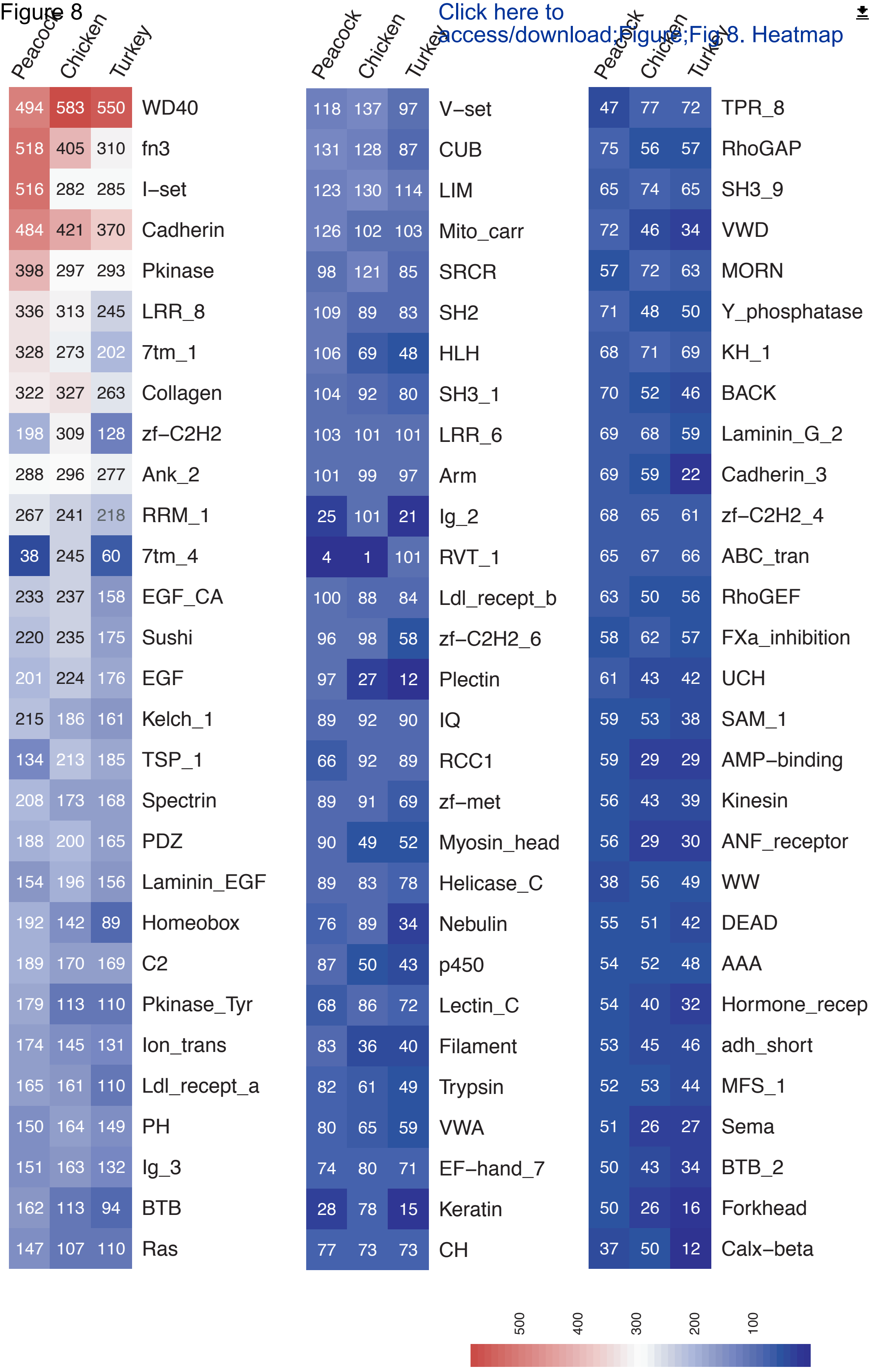

Click here to access/download
Supplementary Material
Supplementary_Description of all the tables and figures.docx

Supplementary Table S1 and S2

Click here to access/download
Supplementary Material
Table_S1_ReadStats_Table_S2_TEs.xlsx

Supplementary Table S3

Click here to access/download
Supplementary Material
Table_S3_Repeats.xlsx

Supplementary Table S4

Click here to access/download
Supplementary Material
Table_S4_Gene_annotations_of_peacock_proteins.xlsx

Supplementary Table S5

Click here to access/download
Supplementary Material
Table_S5_KEGG_annotation.xlsx

Supplementary Table S6

Click here to access/download
Supplementary Material
Table_S6_KOG_annotation.xlsx

Supplementary Table S7

Click here to access/download
Supplementary Material
Table_S7_BlastVsHumanProteins.xlsx

Supplementary Table S8

Click here to access/download
Supplementary Material
Table_S8_Orthologous_proteins

Supplementary Table S9

Click here to access/download
Supplementary Material
Table_S9_Pfam_Analysis.xlsx

Supplementary Fig. S1

Click here to access/download
Supplementary Material
Fig S1. Proteins showing similarity to Pfam domains.pdf

Supplementary Fig. S2

Click here to access/download
Supplementary Material
Fig S2. Gene Ontololgy of top 10 WGS.png

Supplementary Fig. S3

Click here to access/download
Supplementary Material
Fig S3.Peacock vs Human_GO.pdf

Response to reviewer comments

Click here to access/download

Supplementary Material

Reviewed_comments_GigaScience_Final_upload_30Sept2018.docx
