# Supplementary material for: De novo assembly of the Indian blue peacock (Pavo cristatus) genome using Oxford Nanopore technology and Illumina sequencing
Source: Gigascience. 2019 May 11;8(5):giz038. doi: 10.1093/gigascience/giz038 (PMC6511069; doi:10.1093/gigascience/giz038)
Supplement: GIGA-D-18-00280_Revision_2.pdf [file giz038_giga-d-18-00280_revision_2.pdf]

GigaScience

De novo genome assembly of the Indian Blue Peacock (Pavo cristatus), from Oxford Nanopore and Illumina sequencing

--Manuscript Draft--

**Manuscript Number:**	GIGA-D-18-00280R2
**Full Title:**	De novo genome assembly of the Indian Blue Peacock (Pavo cristatus), from Oxford Nanopore and Illumina sequencing
**Article Type:**	Data Note
**Funding Information:**	
**Abstract:**	Background<br>Pavo cristatus, the Indian peafowl are located in natural habitats of South Asia. The male blue peacock bird is known for its elegance, majestic looks and beauty. Since prehistoric times they have been described in Indian culture and has been adopted as the national bird of India. The findings from avian genomics have contributed immensely toward understanding the vertebrate genome evolution. Genome sequencing of the birds performed until recently have been generated by using Sanger, 454, Illumina or Pacbio based next generation sequencing technologies. In this study, we present the first draft genome sequence of the peacock using Illumina and Oxford Nanopore technologies (ONT).<br><br>Findings<br>For the first time in avian genomics, sequencing from ONT has been used for the whole genome assembly. ONT sequencing resulted in approximately 2.3-fold sequencing coverage, whereas Illumina generated 150 bp paired-end sequence data at 284.6-fold sequencing coverage from five libraries. Subsequently, we generated de novo genome assembly of the peacock genome with a 0.915 Gigabases (Gb) with a scaffold N50 of 0.23 Megabases (Mb). We also predicted that the peacock genome contains 23,153 protein-coding genes and 75,315,566 bp (7.33%) of repetitive sequences.<br><br>Conclusions<br>We report a high-quality genome assembly of the peacock using a hybrid assembly generated from Illumina and ONT sequencing platforms. Long read chemistry generated from ONT was found to be useful in addressing challenges related to de novo assembly particularly at regions containing repetitive sequences that span longer than the read length, and which cannot be resolved using only short-read-based assembly. The contig assembly on the short reads from Illumina resulted in an N50 of 1639 bases, whereas using 2.3x coverage from ONT increased the N50 by nine fold to 14,749 bases. The initial contig assembly based on Illumina sequencing reads alone resulted in total of 685,241 contigs. Further scaffolding on assembled contigs using both Illumina and ONT sequencing reads resulted in a final assembly having 15,025 super scaffolds with a N50 of about 0.23 Mb. The reliability of our genome assembly was verified with the fact that 95% of proteins predicted by homology were matched to those submitted in public repository. Further, the phylogentic tree on the conserved genes from the avian species showed P. cristatus being grouped with G. gallus, M. gallopavo and A. platyrhynchos (mallard the duck).
**Corresponding Author:**	Subhradip Karmakar, PhD<br>All India Institute of Medical Sciences<br>New Delhi, Delhi INDIA
**Corresponding Author Secondary Information:**	
**Corresponding Author's Institution:**	All India Institute of Medical Sciences
**Corresponding Author's Secondary Institution:**	
**First Author:**	Ruby Dhar

Powered by Editorial Manager® and ProduXion Manager® from Aries Systems Corporation

**First Author Secondary Information:**	
**Order of Authors:**	Ruby Dhar
	Ashikh Seethy
	Karthikeyan Pethusamy
	Vishwajeet Rohil
	Sunil Singh
	Kakali Purkayastha
	Sandeep Goswami
	Rakesh Singh
	Indrani Mukherjee
	Ankita Raj
	Tryambak Srivastava
	Sovon Acharya
	Balaji Rajashekhar
	Subhradip Karmakar, PhD
**Order of Authors Secondary Information:**	
**Response to Reviewers:**	Dear Dr. Scott,<br>We have addressed all the reviewer's comments and have made significant additional revisions as required by you and both the reviewers. We have removed content on the sexual selection from the manuscript. Below are point-by-point response for the queries raised by the reviewers.<br><br>1.Abstract: Page 2, 12-13: "Its length …" The level of detail in this sentence is inappropriate for an abstract, and it should be removed.<br><br>Reply: the sentence below is completely removed from abstract<br>"Its length varies from 92-125 centimeter (without train), weighing about 4-8 Kilograms and lives up to 20 years in the wild."<br><br>2.Page 3, 15: "Observation from our study showed…", rewrite as "Our study showed…"<br><br>Reply: The sentence has been corrected as suggested.<br><br>3.Page 3, 19: "Further a comparative genomics …" this sentence is grammatically incorrect<br><br>Reply: this sentence is modified to "Further predicted peacock proteins when compared with"<br><br>4.Page 3, 32: "amongst the clade of birds based on their ability to fly". I think you should just indicate the clade with which Pavo was grouped.<br><br>Reply: The sentence is modified to "Further, the phylogentic tree on the conserved genes from the avian species showed the P. cristatus amongst in the clade of G. gallus, M. gallopavo and A. platyrhynchos (mallard),"<br><br>5.Page 4, 27: "The avian genomics began …" This paragraph is still too much introduction and too general to be helpful for the paper. The phrase "The avian genomics" is grammatically incorrect.

Reply: The first 2 sentences are completely removed now the new paragraph starts as "The genome sequencing of the model organism Gallus gallus species (Chicken) (Hillier et al. 2004) and wide variety of avian species (Zhang et al. 2014). have provided a novel perspective on vertebrate genome evolution in better understanding number of distinct characteristics and the annotation of mammalian genomic regions."

6.Page 4, 55: "aves" I think this should be italicized.

Reply: In other published articles "aves" is used as "Aves". To keep in standard format we have changed to Aves without italics.

7.Page 5, 10-15: "We have unraveled …" I don't think that these are demonstrated in the results ("genomic signatures", "gene pools"). I think that "gene pools" is used incorrectly here.

Reply: The sentence is changed from "We have unraveled some of the genomic signatures and thus have reported unique gene pools of this bird by performing comparative genomics."
to
"The protein comparisons between the peacock, chicken and turkey will reveal proteins, conserved domains and functional annotations common and absent between the species."

8.Page 9, 32-33: "The raw data were then base-called …" This reads like it is directly following the MinION library preparation and sequencing section, which it doesn't. It should either be re-written to fix this or the paragraphs should be re-ordered.

Reply: To resolve the issues the paragraphs are arranged under two new broad sections
Library preparation and sequencing
Raw data quality control and processing

9.Page 10, 41-46: "Gene models were predicted on a hard masked draft genome and further genes were predicted using AUGUSTUS" This sounds like gene models were predicted twice (once on the hard-masked genome and once using AUGUSTUS).

Reply: This sentence was corrected to
"Gene models were predicted on a hard masked draft genome using AUGUSTUS"

10.Page 14, 59: "Significant gene Ontology (GO)" Significance in this context implies statistical significance, but no statistical tests are presented. Throughout this section, sometimes results are presented as percentages or counts inconsistently.

Reply: Now the paragraph starts from Gene ontology. In the whole section the total protein numbers (% in brackets) are mentioned. This has been represented uniformly in this paragraph.

11.Page 15, 4-5: The meaning of the phrase "unique proteins" is unclear here, since you're just talking about the set of predicted proteins from the peacock genome.

Reply: The unique protein is changed to "peacock specific proteins" "absent between" or "not clustered with other species" in the entire manuscript

12.Page 15, 12-13: "showed expansions in ontologies" should be "showed expansions in GO categories".

Reply: The sentence has been modified as suggested.

Powered by Editorial Manager® and ProduXion Manager® from Aries Systems Corporation

13.Page 15, 12-13: Fig. S4 appears to be missing from the attachments in this document. I don't know why Table S7 is referenced here. Table S7 doesn't have GO terms or any other functional annotation information.

Reply: Fig. S4 was removed from the manuscript after previous revision. We have removed the Fig. S4 from the manuscript.

14.Page 15, 29-32: "With the stringent cutoff" This makes it sound like there were two cutoffs ¬– a stringent one and a lenient one. This result (13,860 genes unique to peacock) still seems to point to over-prediction in the peacock genes than actual unique genes.

Reply: The cutoff parameters for clustering were 70% alignment coverage and length difference of 0.7. With above cutoff we obtained 13860 clusters not clustering with other avian proteins. This could be due to the sensitivity of the CD-Hit tool to identify highly conserved proteins in avian species. BLAST similarity and further clustering them may result in less number of unique proteins. This will allow short sequences clustered with complete long sequences resulting in false positive results. Due to very low coverage of sequencing of some avian species which may have resulted in incomplete ORF predictions.

15.Page 17, 24-25: The timetree URL isn't the correct way to reference the tool. http://www.timetree.org/faqs#q7

Reply: The following reference have been included in the references section of the manuscript
Kumar S, Stecher G, Suleski M, Hedges SB (2017) TimeTree: A Resource for Timelines, Timetrees, and Divergence Times. Mol Biol Evol 34 (7): 1812-1819

16.Page 17, 51-52: It isn't clear which methods or results section Fig. 8 is connected to. These results should be addressed before the Conclusions section of the paper.

Reply: This sentence has been moved to the results sections under Pfam.
"The domain comparisons between the species showed gene family expansions such as Kinases, Zn finger proteins, GTPases and others in either one of the aforementioned species (Fig. 6)."
The other figure order and also legends have been modified

17.Page 18, 5-6: This paragraph needs to be shortened to one or two sentences pointing out the importance of tail feathers in the biology of the peacock and relevant literature regarding genetic control of plumage that might inform future studies. The discussion of sexual selection is irrelevant to the results presented in this paper. This point has been repeatedly addressed by reviewers in the past two rounds of revision.

Reply: The following paragraph regarding sexual selection have been removed from the manuscript,
One of the most important task will be to characterize the genes involved in the coloration of the tail feather plumage in P. cristatus (Roulin et al. 2013). The peacock feathers have played a significant role in the mating and sexual selection. Peacock seems to defy the Darwinian laws of natural selection. These concern were raised by no other than Darwin himself. Hence, he proposed the theory of the sexual selection where the female can choose for a male with a certain phenotypic feature such as brilliant color or a long tail (Burgess 2001). Peacock's brilliantly colored long tail feathers seems to evolve at the cost of finding its female partner thereby contributing its beneficial genes, even at the cost of making itself vulnerable to predators. A female peafowl in turn tends to choose the mate with the largest and decorated plumage, which indirectly reflects its healthiness and capacity to wade off potential competitors. Thus understanding the formation of beautiful feathers from the genomic context will help in resolving several evolutionary theories on sexual selection that have been discussed on this species.
And is modified into

Powered by Editorial Manager® and ProduXion Manager® from Aries Systems Corporation

The section is now reduced to three new sentences.

18.Page 18, 36-37: “peacock Though” missing period here.

Reply: The period has been included between the sentences.

19.Page 18, 44: suggest replacing “just” with “valuable” here.

Reply: The alternative work have been replaced in the manuscript

20.Page 22, 14-15: I don’t think indicating the flight status of birds is helpful in Fig. 4.

Reply: The figure is modified, the flightless and low flying have been removed from the figure and the figure legend.

Additional notes from letter
Your manuscript "De novo genome assembly of the Indian Blue Peacock (Pavo cristatus), from Oxford Nanopore and Illumina sequencing" (GIGA-D-18-00280R1) has been re-reviewed by our reviewers. Although it is of interest, we are unable to consider it for publication in its current form as significant additional revisions are required. The reviewers have raised a number of points which we believe would improve the manuscript and may allow a revised version to be published in GigaScience so we are giving you one final chance to address these otherwise we cannot keep considering this paper. It is a shame you ignored some of the previously raised significant revisions that need to be made and have been brought up before, specifically the irrelevant discussion of Darwin and sexual selection. In the final version these and the many other speculative discussions need to be removed to just focus on the data and its validation (including the comparisons of the builds of the many bird genomes currently available).

Reply : We have completely removed sections on Darwin and sexual selection. Significant additional revisions as suggested have been made and the details of each correction are described above.

Please include a point-by-point within the 'Response to Reviewers' box in the submission system. Please ensure you describe additional experiments that were carried out and include a detailed rebuttal of any criticisms or requested revisions that you disagreed with. Please also ensure that your revised manuscript conforms to the journal style, which can be found in the Instructions for Authors on the journal homepage.

Reply : https://academic.oup.com/gigascience/pages/instructions_to_authors

The due date for submitting the revised version of your article is 20 Jan 2019.

Reviewer #1: The manuscript entitled "De novo assembly of Indian Blue Peacock (Pavo cristatus), from Oxford Nanopore and Illumina sequencing" details the results from sequencing and assembling the peacock genome. The manuscript is very much improved and should be ready for publication with only minor revisions.

I think this manuscript lacks one very important point. How does this hybrid assembly compare to other avian genome assemblies? For example, the turkey genome used two different genome sequencers while the original chicken genome made use of Sanger sequencing. Furthermore, many of the 48 bird genomes (Jarvis et al.; Zhang et al, 2014) only used Illumina sequencing at different sequencing depths. I think a comparison between these builds (N50, etc.) should be included in this manuscript. This will aid future researchers who are trying to decide the best sequencing strategy for their favorite bird/organism.

Reply :

	The abstract and introduction contain several awkward sentences that impede the reader's understanding. For example, the second to last sentence (lines19-22) of the Abstract Background needs to be rewritten.<br><br>Reply : We have changed the second last sentence in the Abstract Background section.<br><br><br>Reviewer #2: The manuscript is much improved over prior versions, but still needs significant revisions.<br><br>1) The abstract includes too much detail about the general biology of the peacock and can be shortened for clarity and to focus on the results of the manuscript.<br><br>Reply : We have modified our abstract for clarity and have aligned with the other accepted articles in giga science. The biology is completely removed and we have focusedon the key results and the importance of Nanopore long reads.<br><br>2) Citations are not numbered in the text and in some cases do not cite the tool or resource correctly (see my note about timetree.org)<br><br>Reply : The citations are numbered in the text, the timetree.org reference is now correctly cited.<br><br><br>3) A supplementary figure (Fig. S4) is missing from the text and the table referenced at the same point of the many script doesn't contain relevant data.<br><br>Reply : This has been corrected, see above point 13 for details.<br><br><br>4) The Conclusions section includes a largely irrelevant section about sexual selection that needs to be removed.<br><br>Reply : Sections related to sexual selection has been completely removed from the manuscript.<br><br><br>5) The Conclusions includes a first reference of a figure that doesn't seem to be referenced in the Methods or Results sections.<br><br>Reply : This figures is now referenced in results section "Comparison with other species and databases" in the last paragraph as Fig. 7.
**Additional Information:**	
**Question**	**Response**
Are you submitting this manuscript to a special series or article collection?	No
**Experimental design and statistics**<br><br><br>Full details of the experimental design and statistical methods used should be given in the Methods section, as detailed in our Minimum Standards Reporting Checklist. Information essential to interpreting the data presented should be made available in the figure legends.	Yes

Powered by Editorial Manager® and ProduXion Manager® from Aries Systems Corporation

Have you included all the information requested in your manuscript?	
**Resources**<br><br>A description of all resources used, including antibodies, cell lines, animals and software tools, with enough information to allow them to be uniquely identified, should be included in the Methods section. Authors are strongly encouraged to cite Research Resource Identifiers (RRIDs) for antibodies, model organisms and tools, where possible.<br><br>Have you included the information requested as detailed in our Minimum Standards Reporting Checklist?	Yes
**Availability of data and materials**<br><br>All datasets and code on which the conclusions of the paper rely must be either included in your submission or deposited in publicly available repositories (where available and ethically appropriate), referencing such data using a unique identifier in the references and in the "Availability of Data and Materials" section of your manuscript.<br><br>Have you have met the above requirement as detailed in our Minimum Standards Reporting Checklist?	Yes

Manuscript
Click here to view linked References
Click here to access/download;Manuscript;Manuscript_revision_23Dec2018.

De novo genome assembly of the Indian Blue Peacock (*Pavo cristatus*), from Oxford Nanopore and Illumina sequencing

Authors: Ruby Dhar[1], Ashikh Seethy[1], Karthikeyan Pethusamy[1], Vishwajeet Rohil[2], Sunil Singh[1], Kakali Purkayastha[2], Sandeep Goswami[1], Rakesh Singh[3], Indrani Mukherjee[1], Ankita Raj[1], Tryambak Srivastava[1], Sovon Acharya[1], Balaji Rajashekhar[4,5,]* and Subhradip Karmakar[1,]*

Affiliation: [1]Department of Biochemistry, AIIMS, New Delhi, India. [2]Vallabhbhai Patel Chest Institute (VPCI), New Delhi, India. [3]Kanpur Zoo, Kanpur, India. [4]Genotypic Technology Pvt. Ltd., Bangalore, India. and [5]Institute of Computer Science, University of Tartu, 50409 Tartu, Estonia

*Corresponding Authors email: balaji@ut.ee, subhradip.k@aiims.edu

Running Title: *De novo* Genome Assembly of the Peacock Bird

Key words: Peacock, *Pavo cristatus*, Indian National Bird, Genome Assembly, Oxford Nanopore.

Abstract

Background

Pavo cristatus, the Indian peafowl are located in natural habitats of South Asia. The male blue peacock bird is known for its elegance, majestic looks and beauty. Since prehistoric times they have been described in Indian culture and has been adopted as the national bird of India. The findings from avian genomics have contributed immensely toward understanding the vertebrate genome evolution. Genome sequencing of the birds performed until recently have been generated by using Sanger, 454, Illumina or Pacbio based next generation sequencing technologies. In this study, we present the first draft genome sequence of the peacock using Illumina and Oxford Nanopore technologies (ONT).

Findings

For the first time in avian genomics, sequencing from ONT has been used for the whole genome assembly. ONT sequencing resulted in approximately 2.3-fold sequencing coverage, whereas Illumina generated 150 bp paired-end sequence data at 284.6-fold sequencing coverage from five libraries. Subsequently, we generated *de novo* genome assembly of the peacock genome with a 0.915 Gigabases (Gb) with a scaffold N50 of 0.23 Megabases (Mb). We also predicted that the peacock genome contains 23,153 protein-coding genes and 75,315,566 bp (7.33%) of repetitive sequences.

Conclusions

We report a high-quality genome assembly of the peacock using a hybrid assembly generated from Illumina and ONT sequencing platforms. Long read chemistry generated from ONT was found to be useful in addressing challenges related to *de novo* assembly particularly at regions containing repetitive sequences that span longer than the read length, and which

cannot be resolved using only short-read-based assembly. The contig assembly on the short reads from Illumina resulted in an N50 of 1639 bases, whereas using 2.3x coverage from ONT increased the N50 by nine fold to 14,749 bases. The initial contig assembly based on Illumina sequencing reads alone resulted in total of 685,241 contigs. Further scaffolding on assembled contigs using both Illumina and ONT sequencing reads resulted in a final assembly having 15,025 super scaffolds with a N50 of about 0.23 Mb. The reliability of our genome assembly was verified with the fact that 95% of proteins predicted by homology were matched to those submitted in public repository. Further, the phylogentic tree on the conserved genes from the avian species showed *P. cristatus* being grouped with *G. gallus, M. gallopavo* and *A. platyrhynchos* (mallard the duck).

Introduction

Pavo cristatus commonly known as the Indian blue peafowl are native to South Asian countries. Apart from the wild, they are usually found as exhibits in park and zoo, besides being raised for breeding and conservation purposes [1, 2] (Fig. 1). The peacock has been widely referred in ancient Indian literatures. They have been found to be closely associated with the life and culture of the people from South East Asia, symbolizing beauty, love, grace and pride [3, 4]. Owing to these, the peacock obtained the status as the National Bird of India in 1963.

Genome sequencing of the avian model organism *Gallus gallus* (chicken) [6], as well as variety of other avian species [7] have provided a novel perspective on vertebrate genome evolution. This enabled us to understand the genome structure better and annotate the mammalian genome. Genome studies of *Gallus gallus* with respect to the human have revealed an extremely high level of conservation within the orthologous regions [8].

Despite the wealth of information from the existing avian genome sequencing projects, it is still important to sequence genome of other new species to add value, both into avian and vertebrate genomics. For the first time in avian genomics, Oxford Nanopore technology (ONT or Nanopore) has been used to sequence a bird genome presented in this study. Long reads have been helpful during the *de-novo* assembly of the genome especially in the GC rich repeat regions which invariably poses serious challenges in genome construction. Comparative genomics with other birds will help in understanding the uniqueness of peacock genome, development of this species, complex plumage pigmentation, sexual dimorphism and its evolutionary relationships with other birds. The characterization of the genes and association with specific function will provide better understanding of the peafowl species. The protein comparisons among the peacock, chicken and turkey will reveal proteins,

conserved domains and functional annotations that are common and absent among these species.

Materials and methods

Sample collection and extraction of DNA

The whole blood of male peacock was collected from Kanpur zoo, India after obtaining the necessary ethical and institutional approval. Approximately, 20 µl of proteinase K (PK) solution was taken into a 1.5 ml microcentrifuge tube, 200 µl of blood was added and briefly mixed. Furthermore, 200 µl of cell lysis buffer was added to the tube, mixed by vortexing for 10 seconds, incubated at 56°C for 10 minutes. ReliaPrep™ Binding Column was placed into an empty collection tube. Furthermore, 250 µl of Binding Buffer (BBA) was added to the tube, and mixed by vortexing for 10 seconds with a vortex mixer. Contents of the tube were added to the ReliaPrep™ binding column, capped and placed in a refrigerated microcentrifuge. These were then centrifuged for 1 minute at maximum speed and flow through was discarded. Binding column was placed into a fresh collection tube. In addition, 500 µl of column wash solution was added to the column and centrifuged for 3 minutes at maximum speed; flow through was again discarded. Column washing is repeated thrice. Columns were then placed in a nuclease free clean 1.5 ml microcentrifuge tube. Furthermore, 100 µl of Nuclease-Free Water was then added to the column and centrifuged for an additional 1 minute at maximum speed. Column was discarded and elute was saved. The concentration and purity of the extracted DNA was evaluated using Nanodrop Spectrophotometer (Thermo Scientific) and Qubit flurometer and integrity was checked on a 0.8% agarose gel. The DNA sample was aliquoted for library preparation on two different platforms: Illumina HiSeq4000 and Oxford Nanopore Technologies (ONT).

Library preparation and sequencing

A. Paired-End library preparation and sequencing

Whole genome sequencing (WGS) libraries were prepared with Illumina-compatible NEXTflex DNA sequencing kit (BIOO Scientific, Austin, TX, USA). Approximately, 1 μg of genomic DNA was sheared using Covaris S2 sonicator (Covaris, Woburn, MA, USA) to generate approximate fragment size distribution from 300 - 600 basepair (bp). The fragment size distribution was checked on Agilent 2200 Tape Station with D1000 DNA screen tapes and reagents (Agilent Technologies, Palo Alto, CA, USA) and subsequently purified using HighPrep magnetic beads (Magbio Genomics Inc, USA). The purified fragments were end-repaired, adenylated and ligated to Illumina multiplex barcode adaptors as per NEXTflex DNA sequencing kit protocol (BIOO Scientific, Austin, TX, USA).

The adapter-ligated DNA was purified with HighPrep beads (MagBio Genomics, Inc, Gaithersburg, MD, USA) and then size selected on 2% low melting agarose gel and cleaned using MinElute column (QIAGEN). The resultant fragments were amplified for 10 cycles of PCR using Illumina-compatible primers provided in the NEXTFlex DNA sequencing kit. The final PCR product (sequencing library) was purified with HighPrep beads, followed by library quality control check. The Illumina-compatible sequencing library was initially quantified by Qubit fluorometer (Thermo Fisher Scientific, MA, USA) and its fragment size distribution was analyzed on Agilent TapeStation. Finally, the sequencing library was accurately quantified by quantitative PCR using Kapa Library Quantification Kit (Kapa Biosystems, Wilmington, MA, USA). The qPCR-quantified library was subjected to sequencing on an Illumina sequencer for 150 bp paired-end chemistry.

The Illumina-compatible sequencing library for the samples has a fragment size range between 275 - 425 bp for Paired-End Short Insert (PE-SI) and 350 - 650 bp for Paired-End Long Insert (PE-LI). As the combined adapter size is approximately 120 bp, the effective user-defined insert size is 155 - 305 bp and 230 - 530 bp for PE-SI and PE-LI, respectively. Libraries were sequenced in Illumina HiSeq platform with 150 PE chemistry.

B. Mate-Pair library preparation and sequencing

Mate Pair sequencing library was prepared with Illumina-compatible Nextera Mate Pair Sample Preparation Kit (Illumina Inc., Austin, TX, USA). Approximately, 4 ug of genomic DNA was simultaneously fragmented and tagged with Mate Pair adapters in a transposon-based tagmentation step. Tagmented DNA was then purified using AMPure XP Magnetic beads (Beckman Coulter Life Sciences, Indianapolis, IN, USA) followed by strand displacement to fill gaps in the tagmented DNA. Strand displaced DNA was further purified with AMPure XP beads before size-selecting the 3 - 5 kilobases (kb), 5 - 7 kb & 7 - 10 kb fragments on low melting agarose gel. The fragments were circularized in an overnight blunt-end intra-molecular ligation step, which will result in circularization of DNA with the insert mate pair adapter junction. The circularized DNA was sheared using Covaris S220 sonicator (Covaris, Woburn, MA, USA) to generate approximate fragment size distribution from 300 - 1000 bp. The sheared DNA was purified to collect the mate pair junction positive fragments using Dynabeads M-280 Streptavidin Magnetic beads (Thermo Fisher Scientific, Waltham, MA, USA). The purified fragments were end-repaired, adenylated and ligated to Illumina multiplex barcode adaptors as per Nextera Mate Pair Sample Preparation Kit protocol.

The adapter-ligated DNA was then amplified for 15 cycles of PCR using Illumina-compatible primers. The final PCR product (sequencing library) was purified with AMPure XP beads,

followed by library quality control check. The Illumina compatible sequencing library was initially quantified by Qubit fluorometer (Thermo Fisher Scientific, MA, USA), and its fragment size distribution was analyzed on Agilent TapeStation. Finally, the sequencing library was accurately quantified by quantitative PCR using Kapa Library Quantification Kit (Kapa Biosystems, Wilmington, MA, USA). The qPCR quantified libraries were pooled in equimolar amounts to create a final multiplexed library pool for sequencing on an Illumina sequencer.

C. Oxford Nanopore MinION library preparation and sequencing

Genomic DNA (1.5μg) was end-repaired (NEBnext ultra II end repair kit, New England Biolabs, MA, USA), cleaned up with 1x AmPure beads (Beckmann Coulter, USA). Adapter ligations were performed for 20 minutes using NEB blunt/TA ligase (New England Biolabs, MA, USA). Library mix were cleaned up using 0.4X AmPure beads (Beckmann Coulter, USA) and eluted in 25 μl of elution buffer. Eluted library was used for sequencing. Whole genome library were prepared by using ligation sequencing SQK-LSK108 Oxford Nanopore sequencing kit (ONT, Oxford, UK). Sequencing was performed on MinION Mk1b (ONT, Oxford, UK) using SpotON flow cell (FLO-MIN106) in a 48 hour sequencing protocol on MinKNOW (1.1.20 from ONT).

Raw data quality control and processing

A. Illumina raw data quality control and processing

The Illumina reads were de-multiplexed using Illumina bcl2fastq. The Illumina generated raw data for genomic libraries was quality checked using FastQC (http://www.bioinformatics.babraham.ac.uk/projects/fastqc/) (Andrews, S., 2010). The paired-end Illumina reads were processed for clipping the adapter and low-quality bases

using customized script which retains minimum 70% bases/reads with Phred score (Q≥30 in each base position) with a read length of 50 bp. The MP libraries were trimmed for adapter and low-quality base trimming from the 3'-end using PLATANUS internal trimmer (http://platanus.bio.titech.ac.jp/) [11].

B. Oxford Nanopore reads base calling and processing

The raw data were then base-called with the cloud-based Metrichor workflow 2D Basecalling plus Barcoding by Metrichor (V.2.43.1 from ONT, https://nanoporetech.com/products/metrichor). The Oxford Nanopore reads were processed using Poretools [12] for converting fast5 files to fasta format. For further quantification and analysis the 2D reads or 1D high quality reads were selected for further assembly.

***De novo* genome assembly and genome size estimation**

The quality checked Oxford Nanopore reads were error-corrected using Illumina PE reads. For error-correction the Illumina PE-reads were aligned to the Nanopore reads by using BWA aligner [13]. The paired-end reads were assembled using Abyss [14] followed by contig extension using Oxford Nanopore reads using SSPACE-LongRead [15]. Super scaffolding of the assembled scaffold was performed using SSPACE [16] and PLATANUS (http://platanus.bio.titech.ac.jp/) using the Oxford Nanopore and Matepair data. Final draft genome resulted after gap closure by GAPCLOSER (http://sourceforge.net/projects/soapdenovo2/files/GapCloser/) and PLATANUS gap_close tool (http://platanus.bio.titech.ac.jp/) using Illumina data. The genome size was estimated using a k-mer distribution plot using JELLYFISH [17]. The assembly and annotation workflow has been represented in Figure 2.

Identification of repetitive elements and SSR markers

Repetitive elements, retrotransposons and DNA transposons were identified in the draft genome and was hard masked by using reference genomic repeats of *G. gallus* using Repeatmasker tool (www.repeatmasker.org/). Final assembled scaffolds were analysed for Simple Sequence Repeats (SSR) identification. SSRs like the di, tri, tetra, penta and hexa-nucleotide repeats in the genome were obtained using MISA (Version 1.0.0) (http://pgrc.ipk-gatersleben.de/misa/).

Annotation of the draft genome

Gene models were predicted on a hard-masked draft genome using AUGUSTUS (http://augustus.gobics.de/) with *G. gallus* (red junglefowl the chicken) as a reference model. The predicted proteins were annotated by using BLASTP [18] against the NCBI NR (non-redundant) database with default parameters at E-value cutoff of 1E-5.

The predicted proteins were searched against the KEGG-KAAS server (http://www.genome.jp/tools/kaas/) for pathway analysis [19]. *G. gallus*, *M. gallopavo* (turkey), *Taeniopygia guttata* (zebra finch), *Falco peregrinus* (peregrine falcon) were used as reference organism for pathway identification. The EuKaryotic Orthologous Groups (KOGs) (https://genome.jgi.doe.gov/portal/help/kogbrowser.jsf) were predicted using homology-based approach.

Prediction of protein domains

Predicted proteins from peacock, chicken and turkey with sequence length greater than 100 amino acids were considered for protein domain analysis. All the protein sequences from each organism were searched against Pfam-A database (http://pfam.sanger.ac.uk/) using

Pfam scan (https://www.ebi.ac.uk/seqdb/confluence/display/THD/PfamScan) for protein domain identification.

Identification of avian protein families

A total of 748,544 protein sequences from 49 avian species (including peacock proteins from this study) and others were downloaded from http://avian.genomics.cn/en/jsp/database.shtml. Sequences greater than 100 amino acids from all the avian genomes were selected and concatenated to a single fasta file. These sequences were clustered using CD-HIT [20] with 70% alignment coverage for the shorter sequence with a length difference cutoff of 0.7. The single copy gene family orthologs present across all avian species and not clustered peacock proteins were annotated.

Phylogenetic tree construction

For phylogenetic tree construction we considered single copy gene clusters present as single copy in all the avian species. These protein sequences from each species were concatenated and were further aligned by multiple sequence alignment tool Clustalw (http://www.clustal.org/clustal2). The poorly aligned positions and divergent regions were removed using Gblock tool (http://molevol.cmima.csic.es/castresana/Gblocks.html). The fasta format sequences were converted to phylip format using Phylip tool (http://evolution.genetics.washington.edu/phylip/getme-new1.html). Phylogenetic trees were constructed using IQ-TREE version 1.5.6 (www.iqtree.org). The parameters used for phylogenetic tree construction were ultrafast boostrap (UFBoot, using the –bb option of 1000 replicates), and a standard substitution model (-st AA –m TEST) and alrt 1000 -nt AUTO was given for tree generation. The generated trees from IQ-TREE tool were visualized using Figtree (http://tree.bio.ed.ac.uk/software/figtree/) and the Brach-support values were recorded

from the output ".treefile". The trees were modified for better visualization under Trees section an increasing order nodes were applied.

Genome conservation analysis

Draft chromosome visualizations were constructed by aligning the assembled peacock genome against the *G. gallus* with the Chromosomer tool (https://github.com/gtamazian/chromosomer). The reordered assembled genome was aligned against the chicken genome using LAST aligner (http://last.cbrc.jp/) with NEAR (finding short-and-strong [near-identical] similarities) parameter allowing for substitution and gap frequencies, leading to the identification of orthologs. These query-mapped regions were filtered with a greater than 1% of the maximum length for visualization using Circos (http://circos.ca/).

Results

Genome sequencing assessment

A total of five libraries from Illumina HiSeq technology of 150 bp paired-end were generated. The short-insert reads of 489,114,747 accounted to genome coverage of 146.7X and long-insert reads of 302,884,819 sequences was about 90.9X coverage with a total coverage of 237.6X. Sequencing of three mate-pairs of 3-5Kb, 5-7Kb of and 7-10Kb yielded 72,915,033, 47,440,144 and 36,464,628 reads, respectively with an approximate coverage of 21.9X, 14.2X and 10.9X, respectively, with a grand total of 156 million mate-pair reads of 47X coverage. Oxford Nanopore technology was used to generate 366,323 long reads having of 2,398,560,283 bp with coverage of 2.3X. The complete genome sequencing was generated to a depth of ~287X from both Illumina and Oxford Nanopore platform (Table 1). The coverage was based on the assumption that the peacock genome size of about 1 Gb.

Genome assembly

The first assembly was performed on Illumina reads with Abyss *de novo* assembler that resulted in ~932 Mb (mega base) of genome with an N50 of 1639 bp. The extension of the contigs were performed with Oxford Nanopore reads, which generated scaffolds with N50 of 14,748 bp. Super scaffolding of the assembled scaffold was performed using SSPACE and PLATANUS with MP libraries that generated ~916 Mb genome with the N50 value of 168,140 bp. The final gap closer was executed using GAPCLOSER program with MP and PE-LI libraries, which generated a draft genome of 1.02 GB (giga base). The draft genome assembly of *Pavo cristatus* consists of 179,346 bp scaffolds, with a N50 of 189,886 bp with 37 scaffolds, having sequence length >=1 Mbp. Contigs above 5000 bp have covered a genome of ~0.915 Mb with N50 0.23 Mb. In the assembled genome there were ~0.4% of non-ATGC characters (Table 2).

Repetitive genome elements and SSR markers

A total of 75,315,566 bp (7.33%) of the peacock genome was estimated to consist of repeat sequences (Table S1). In the genome about 56,511,635 bp (5.5%) of retrotransposons (class I) were identified as the NON-LTR elements (LINEs (4.7%), SINEs (0.08%) and LTR elements (0.72%). Subsequently, the DNA transposons (class II) of 7,277,390 bp (0.71%) and unclassified elements of about 467,719 (0.05%) were identified (Table S1). The median percentages of LINEs, SINEs, LTR, DNA, unknown and total masked bases of other avian birds were 3.94, 0.11, 1.31, 0.22, 0.85 and 6.93, respectively (Table S2).

A total of 399,493 SSRs were obtained from the peacock genome assembly. The largest fraction of SSRs identified were mono-nucleotide (60.04%), followed by tetra-nucleotide

(26%), di-nucleotide (8.51%), tri-nucleotide (4.31%), penta-nucleotide (1.03%) and finally hexa-nucleotide (0.13%). Among the SSRs identified, A (49.2%) and T (44.9%) accounted for 94.1% of the mono-nucleotide repeats. AT (23.8%), TA (16.5%), TG (13.7%), AC (10.6%) and CA (10.32%) accounted for 75% of the di-nucleotide repeats, whereas TTG (9.9%), AAT (9.6%), AAC (9.4%), TTA (7.1%), ATT (4.5%), TAA (3.5%), CAA (3.1%) and GGA (2.69%) accounted for 49.7% of the tri-nucleotide repeats (Table S3).

Gene prediction and annotation

A total of 23,153 proteins were predicted from the assembled draft peacock genome using AUGUSTUS. Among them, 21,854 (94.4%) predicted proteins showed homology to other sequences from the NCBI NR database (Fig. 3). The top four organisms where the peacock proteins showed homology belonged to the *G. gallus* with 11,398 proteins, *M. gallopavo* with 4059 proteins, *Amazona aestiva* (blue-fronted Amazon parrot) with 1352 proteins and *Anas platyrhynchos* (mallard the duck) with 849 proteins. The detail annotations of all the proteins are available in Table S4.

Gene Ontology (GO) descriptions were assigned for a total of 18,294 (79%) peacock proteins. Among them, 14,489 proteins have molecular function; 11,678 have biological process and 13,735 proteins have cellular component as functional categories (Table S4). A total of 4091 (17.7%) peacock proteins were found to have pathway information from the KEGG database (Table S5), whereas a total of 20,937 (88.1%) peacock proteins found a similarity against the KOG annotations (Table S6). The peacock proteins when searched against the Human proteins showed expansions in cell morphogenesis, neuronal projection and development and GTPases (Table S7 and Fig. S3).

Analysis of avian protein families

A total of 748,544 protein sequences from 49 avian species have 653,497 protein sequences of length above 100 amino acids (Table S8A). Based on the level of identity CD-HIT clustered all the proteins into a total of 114,121 gene clusters. Among them, 68 highly homologous gene clusters were present as single copy in all the 49 avian species (Table S8B and Table S8C). We also observed 13,860 protein clusters of peacock species not clustered with other species (Table S8D).

Phylogenetic analysis

The phylogenetic analysis of 48 avian species along with peacock genome showed clustering of the *P. cristatus* species in a clade of *G. gallus* (chicken), *M. gallopavo* (turkey), *A. platyrhynchos* (mallard the duck), *Tinamus guttatus* (white-throated tinamou) and *Struthio camelus* (ostrich). This is the largest clade with six species having a bootstrap support of a 100. In the aforementioned clade, except the mallard species all belong to flightless or low flying birds. The bootstrap support between *P. cristatus* and *G. gallus* were 96, followed by *M. gallopavo*of 100 bootstrap support (Fig. 4).

Comparison with other species and databases

Predicted proteins from peacock, chicken and turkey when searched for the conserved Pfam protein domains showed about 81% of the domains that were common among these three species (Fig. 5, Table S9). In comparison with the total Pfam domains from all the three species, 94%, 98.4% and 99.7% Pfam domains were present in peacock, chicken and turkey, respectively. However, 255, 69 and 14 Pfam domains were absent among the species comparisons, respectively (Table S9H).

There were 15,470 (78%), 12,794 (85%) and 11,745 (85%) of the peacock, chicken and turkey proteins found to contain a match to Pfam domains, respectively (Table S9). The domain comparisons among the species showed gene family expansions such as kinases, Zn finger proteins, GTPases and others in either one of the species (Fig. 6). Commonly, a total of 9974 peacock proteins were found to have annotation in all the four databases NCBI-NR, KOG, Pfam and GO (Fig. 7). The assembled peacock genome when reordered for pseudo chromosomes generation against the masked 1.21 GB chicken genome [21] showed a 597 MB reordered peacock genome (Fig. 8). There are around 60 different avian species that have been sequenced by using various sequencing technologies (Table S10). The sequencing depth varies from as low as 6x to maximum of 390x coverage. The result obtained from different bioinformatics methods to assemble the sequencing data are measured as scaffold N50 that is, from 30 kb to 14 Mb.

Conclusions

A rapid surge in de-novo genome sequence assembly of diverse species is seen in recent years. This is essentially driven largely due to an affordable cost per base sequencing along with the development of smarter algorithms refined and equipped to handle large data sets. The challenge of newer genome analysis pipeline lies in generating assembly with lower contig numbers and longer contigs per genome. To achieve this, technologies that generate longer reads or greater read depths are found to be very helpful; but most importantly combination of different sequencing technologies play a significant role in improving genome assemblies (Table S10). Libraries generated using different chemistry have been found to be superior on improving assemblies. Further, a combination of different sequencing platform like Illumina when used in combination with other technologies like Sanger sequencing, Pacbio and ONT have shown to reduce the number of scaffolds even with very low coverage. Thus, we need to consider combination of sequencing technologies, along with using

different bioinformatics software to obtain assembly with fewer number or scaffolds or closer to chromosome-level.

In comparison with other avian genomes [22], the 290X sequencing depth generated for peacock is one of the highest. The final draft genome assembly of peacock resulted in N50 of 0.23 MB. Inclusion of 2.3X of reads from Oxford Nanopore helped the assembly to improve by 26.2% reduction in the number of scaffolds and about 50.7% and 115% increase in the scaffold and contig N50, respectively. The draft assembly contained less than 0.4% of unknown nucleotides, which is very low for a draft assembly. Thus, we have shown for the first time in avian genomics how the low-cost third generation sequencing data from Oxford Nanopore can play a significant role in improving the genomes draft assembly. Assemblies with longer scaffolds will further benefit in understanding the organisms with structurally complex regions, repeat elements and isoforms in the genome [23].

Comparisons of the genome features of peacock against other species in different genomic databases have shown about 95% homology (Fig. 7). The genome sequence also gives insights on its genetic lineage and evolution with relation to the other avian members. The estimated median divergence time of *P. cristatus* from *G. gallus* is of about 35 million years ago (MYA), whereas between *G. gallus* and *M. gallopavo* is about 37 MYA [24]. The huge gap of other avians to peacock is due to non-availability of genome sequences from other avians. The gap can be by sequencing other avian species. Among the vertebrates, it has been observed that the variations in TEs among avians are very low [25] (Table S8). The genome complexities of a species are influenced by the transposable elements (TE) that are believed to play a crucial role [26]. In this peacock genome assembly, inclusion of Oxford Nanopore long read sequences has significantly improved the assembly, thus, helping in resolving across the repetitive regions in genome. Their roles in development and evolution of the peacocks need to be further explored.

The genome information of peacock can be valued and explored by avian enthusiasts to further understand about the avian world. Though not yet critically endangered in India, peafowl population

is surely at a declining trend in the wild due to massive deforestation, habitat loss [27] and increased poaching for meat and feathers. Our genome sequencing initiative of *Pavo cristatus* is not only valuable from a conservational viewpoint, but also to preserve a heritage associated with this bird that runs through centuries and that bears a strong attachment to the national psyche.

Availability of supporting data

Supplementary data contains, read statistics, annotation, repeats identification, orthology analysis, assembly and annotation. Figures, Gene ontology and annotations. Additional data are available from https://biit.cs.ut.ee/supplementary/peacock/

Raw Data and genome assembly in SRA

Raw reads (Illumina and Oxford Nanopore) are available in the Sequence Read Archive (SRA), and the Whole Genome Shotgun project has been deposited at GenBank under SRA Submission ID: SUB3108024, Bioproject: PRJNA413288 and Biosamples SUB3108018/SAMN07739105 : SKPea2016_SI, SUB3108017/SAMN07739104 : SKPea2016_LI, SUB3107930/SAMN07739101 : FPL_3_5KB, SUB3108015/SAMN07739102 : FPL_5_7KB, SUB3108016/SAMN07739103 : FPL_7_10KB and SUB3108020/SAMN07739107 : FPL_Nano (Table 1). The *de novo* genome assembly can be accessed under SUB4504869/ SAMN07739105.

Competing interests

The author(s) declare that they have no competing interests.

Authors contributions

RD, AS, KP performed wet lab experiments; RD designed work plan, experiments and logistics; SS, VR, KP SG IM and AR assisted with the work; RS provided samples from bird; BR, SK performed data analysis and interpretation; BR, SK drafted the manuscript and SK overseen the whole project.

Acknowledgements

Department of Biochemistry, AIIMS, New Delhi for providing space and infrastructure to carry on the work. RD for providing partial funding. Raja, Sudha from Genotypic Technology and their team Deepti, Shalini, Nagu, Abhi and Manish for providing sequencing services. BR acknowledges IUT 34-4.

Tables

Table 1. Raw data statistics of Illumina HiSeq and Nanopore reads of the peacock genome.

Sample	Platform	Library and chemistry	Number of reads	Coverage	SRA ID
SO_6221_SKPea2016_SI	HiSeq	PE – SI (150 * 2)	489114747	146.73	SUB3108018, SAMN07739105
SO_6221_SKPea2016_LI	HiSeq	PE – LI (150 * 2)	302884819	90.87	SUB3108017, SAMN07739104
SO_6221_FPL_3_5KB	HiSeq	MP (150 * 2)	72915033	21.87	SUB3107930, SAMN07739101
SO_6221_FPL_5_7KB	HiSeq	MP (150 * 2)	47440144	14.23	SUB3108015, SAMN07739102
SO_6221_FPL_7_10KB	HiSeq	MP (150 * 2)	36464628	10.94	SUB3108016, SAMN07739103
SO_6221_NP	Nanopore	5 - 341124	366323	2.3	SUB3108020, SAMN07739107

Abbreviations used, PE = Paired end, SI = Short Insert, LI = Long insert, MP = Mate pair, NP = Nano pore and KB = Kilo Bases

Table 2. *De novo* assembly statistics of the peacock genome.

Description	Contigs	Nanopore Scaffold	Super Scaffolds	GapClosed	>1000 Kb	>5000 Kb
Contigs	685,241	281,272	179,346	179,332	34,178	15,025
Maximum Length	49,159	251,510	2,390,121	2,488,982	2,488,982	2,488,982
Minimum Length	300	5	265	265	1000	5000
Average Length	1360	3250	5111	5729	-	-
Total Length	932,162,464	914,363,908	916,720,956	1,027,510,962	954,449,349	915,342,012
Length >= 100 bp	685,241	281,271	179,346	179,332	34,178	15,025
Length >= 200 bp	685,241	281,271	179,346	179,332	34,178	15,025
Length >= 500 bp	616,120	186,433	93,727	93,718	34,178	15,025
Length >= 1 Kbp	363,428	104,479	34,168	34,178	34,178	15,025
Length >= 10 Kbp	1591	24,748	9249	10,310	10,310	10,310
Length >= 1 Mbp	0	0	27	37	37	37
Non-ATGC #	350,325	42,696,911	49,169,831	4,043,129	4,040,790	3,986,487
Non-ATGC %	0.038	4.67	5.36	0.393	0.423	0.436
N50 value	1639	14,748	168,140	190,304	218,023	232,312

Figure legend

Figure 1. The beautiful and charismatic photo of Indian blue peacock (*Pavo cristatus)* bird.

Figure 2. Detailed workflow for *de novo* whole genome assembly and annotation.

Figure 3. Peacock proteins showing homology**.** Pie chart showing significant similarity scores of peacock proteins against the NR database.

Figure 4. Phylogenetic tree generated from homologous proteins from 49 different avian species.

Figure 5. Venn diagram showing common and not present protein family domains (Pfam) between peacock, chicken and turkey proteins.

Figure 6. Heatmap showing protein family (Pfam) distributed in peacock, chicken or turkey species. The number represents the Pfam domain count predicted from the protein sequences. Pfam domains of 50 and above identified in any one of the species are compared in the heatmap.

Figure 7. Venn diagram showing peacock proteins showing significant homology to NR database, KOG, Pfam and GO ontologies.

Figure 8. Circular image of the assembled peacock genome aligned against the *G. gallus* genome using Chromosomer tool. Draft chromosomes were generated by similarity between scaffolds that were arranged on the reference chicken genome. Circos was used for visualization. The right side of the image represents the reference chicken genome and left side of the image represents the peacock genome.

References:

1. Brickle, N. 2002. Habitat use, predicted distribution and conservation of green peafowl (*Pavo muticus*) in Dak Lak Province, Vietnam. Biological Conservation, 105: 189-197.

2. Jackson, C. 2006. Peacock. London: Reaktion Books Ltd.

3. Gadagkar, R., 2003. Is the peacock merely beautiful or also honest?. Current Science, 85(7), pp.1012-1020.

4. Kushwaha, S., and Kumar, A. 2016. A Review on Indian Peafowl (*Pavo cristatus*) Linnaeus, 1758. Journal of Wildlife and Research, 4, 42-59.

5. Kadgaonkar, Shivendra B. 1993. The peacock in ancient Indian art and literature. Bulletin of the Deccan College Research Institute, vol. 53, pp. 95–115. JSTOR, www.jstor.org/stable/42936434.

6. Hillier LW, Miller W, Birney E, Warren W, International Chicken Genome Sequencing Consortium et al. 2004 Sequence and comparative analysis of the chicken genome provide unique perspectives on vertebrate evolution. Nature 432:695–716

7. Zhang, G., Jarvis, E. D., and Gilbert, M. T. P. 2014. A flock of genomes. Science 346, 1308–1309.

8. Bejerano, G., Pheasant, M., Makunin, I., Stephen, S., Kent, W.J., Mattick, J.S. and Haussler, D., 2004. Ultraconserved elements in the human genome. Science, 304(5675), pp.1321-1325.

9. Burt, D.W., 2007. Emergence of the chicken as a model organism: implications for agriculture and biology. Poultry science, 86(7), pp.1460-1471.

10. Furlong, R.F., 2005. Insights into vertebrate evolution from the chicken genome sequence. Genome biology, 6(2), p.207.

11. Kajitani, R., Toshimoto, K., Noguchi, H., Toyoda, A., Ogura, Y., Okuno, M., Yabana, M., Harada, M., Nagayasu, E., Maruyama, H. and Kohara, Y., 2014. Efficient de novo assembly of highly heterozygous genomes from whole-genome shotgun short reads. Genome research, 24(8), pp.1384-1395.

12. Loman, N. J. and Quinlan, A. R. 2014. Poretools: a toolkit for analyzing nanopore sequence data. Bioinformatics, 30(23), 3399-3401.

13. Li H. and Durbin R. 2009. Fast and accurate short read alignment with Burrows-Wheeler Transform. Bioinformatics, 25:1754-60.

14. Birol, I., Jackman, S.D., Nielsen, C.B., Qian, J.Q., Varhol, R., Stazyk, G., Morin, R.D., Zhao, Y., Hirst, M., Schein, J.E. and Horsman, D.E., 2009. De novo transcriptome assembly with ABySS. Bioinformatics, 25(21), pp.2872-2877.

15. Boetzer, Marten, and Walter Pirovano. 2014. SSPACE-LongRead: scaffolding bacterial draft genomes using long read sequence information. BMC bioinformatics 15.1: 211

16. Boetzer, M., Henkel, C.V., Jansen, H.J., Butler, D. and Pirovano, W., 2010. Scaffolding pre-assembled contigs using SSPACE. Bioinformatics, 27(4), pp.578-579.

17. Marcais, G and Kingsford, C. 2011. A fast, lock-free approach for efficient parallel counting of occurrences of k-mers. Bioinformatics 27(6): 764-770.

18. Altschul, S.F., Gish, W., Miller, W., Myers, E.W. and Lipman, D.J., 1990. Basic local alignment search tool. Journal of molecular biology, 215(3), pp.403-410.

19. Moriya, Y., Itoh, M., Okuda, S., Yoshizawa, A.C. and Kanehisa, M., 2007. KAAS: an automatic genome annotation and pathway reconstruction server. Nucleic acids research, 35(suppl_2), pp.W182-W185.

20. Fu, L., Niu, B., Zhu, Z., Wu, S. and Li, W., 2012. CD-HIT: accelerated for clustering the next-generation sequencing data. Bioinformatics, 28(23), pp.3150-3152.

21. Warren, W.C., Hillier, L.W., Tomlinson, C., Minx, P., Kremitzki, M., Graves, T., Markovic, C., Bouk, N., Pruitt, K.D., Thibaud-Nissen, F. and Schneider, V., 2016. A new chicken genome assembly provides insight into avian genome structure. G3: Genes, Genomes, Genetics, pp.g3-116.

22. Zhang, G., Li, C., Li, Q., Li, B., Larkin, D.M., Lee, C., Storz, J.F., Antunes, A., Greenwold, M.J., Meredith, R.W. and Ödeen, A., 2014. Comparative genomics reveals insights into avian genome evolution and adaptation. Science, 346(6215), pp.1311-1320.

23. Goodwin, S., McPherson, J.D. and McCombie, W.R., 2016. Coming of age: ten years of next-generation sequencing technologies. Nature Reviews Genetics, 17(6), p.333.

24. Kumar S, Stecher G, Suleski M, Hedges SB., 2017. TimeTree: A Resource for Timelines, Timetrees, and Divergence Times. Mol Biol Evol 34 (7): 1812-1819

25. Sotero-Caio, C.G., Platt, R.N., Suh, A. and Ray, D.A., 2017. Evolution and diversity of transposable elements in vertebrate genomes. Genome biology and evolution, 9(1), pp.161-177.

26. Kapusta, A. and Suh, A., 2017. Evolution of bird genomes—a transposon's-eye view. Annals of the New York Academy of Sciences, 1389(1), pp.164-185.

27. Ramesh, K. and McGowan, P., 2009. On the current status of Indian peafowl Pavo cristatus (Aves: Galliformes: Phasianidae): keeping the common species common. Journal of Threatened Taxa, 1(2), pp.106-108.

Figure 1

Click here to access/download;Figure;Fig 1. Peacock bird.jpg

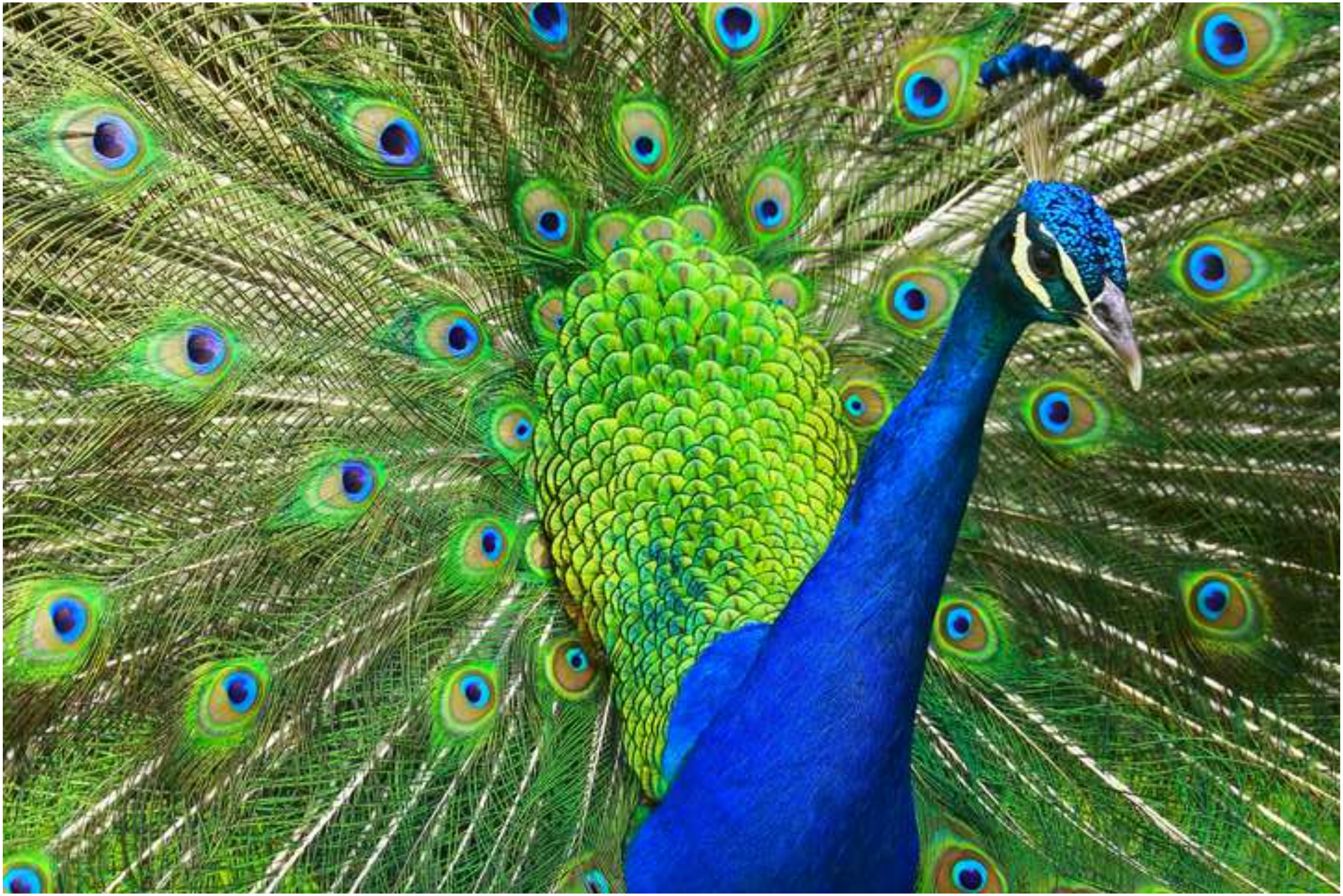

Figure 2 Click here to access/download;Figure;Fig 2. Workflow.jpeg

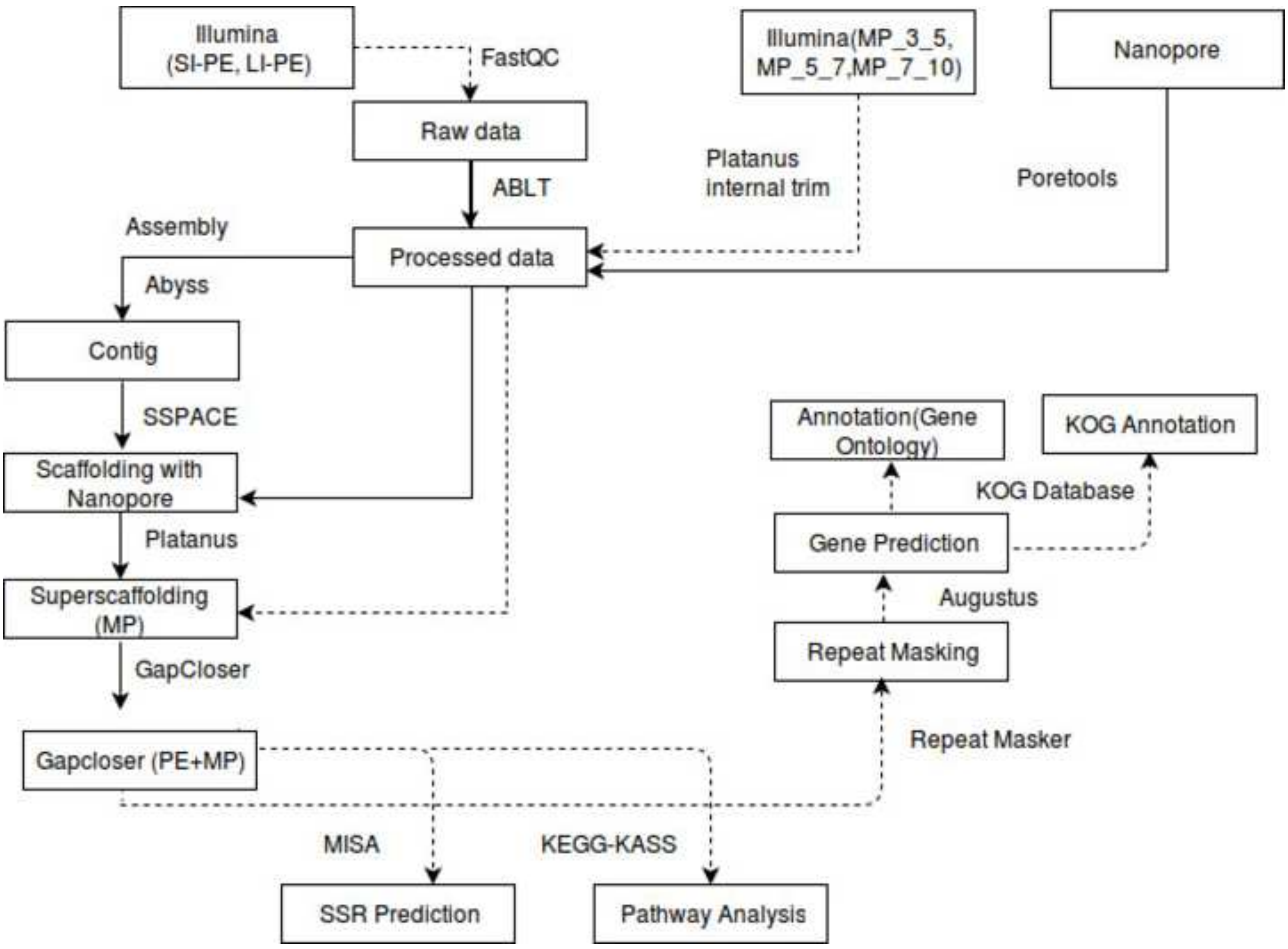

Figure 3

Click here to access/download;Figure;Fig 3. Similarity scores against the Uniprot database.pdf

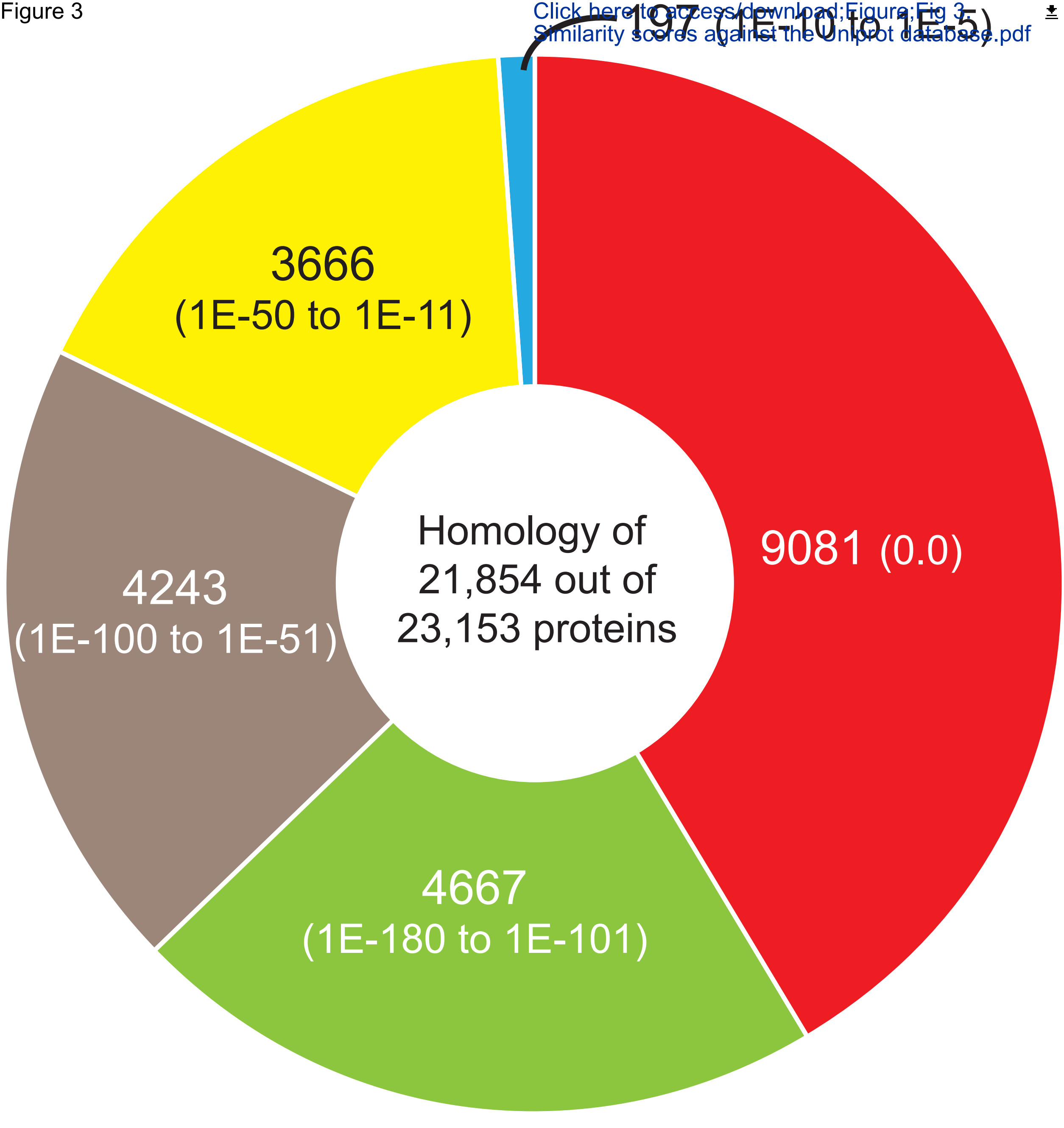

Figure 4

Click here to access/download;Figure;Fig 4. Phylogeny of avian species.pdf

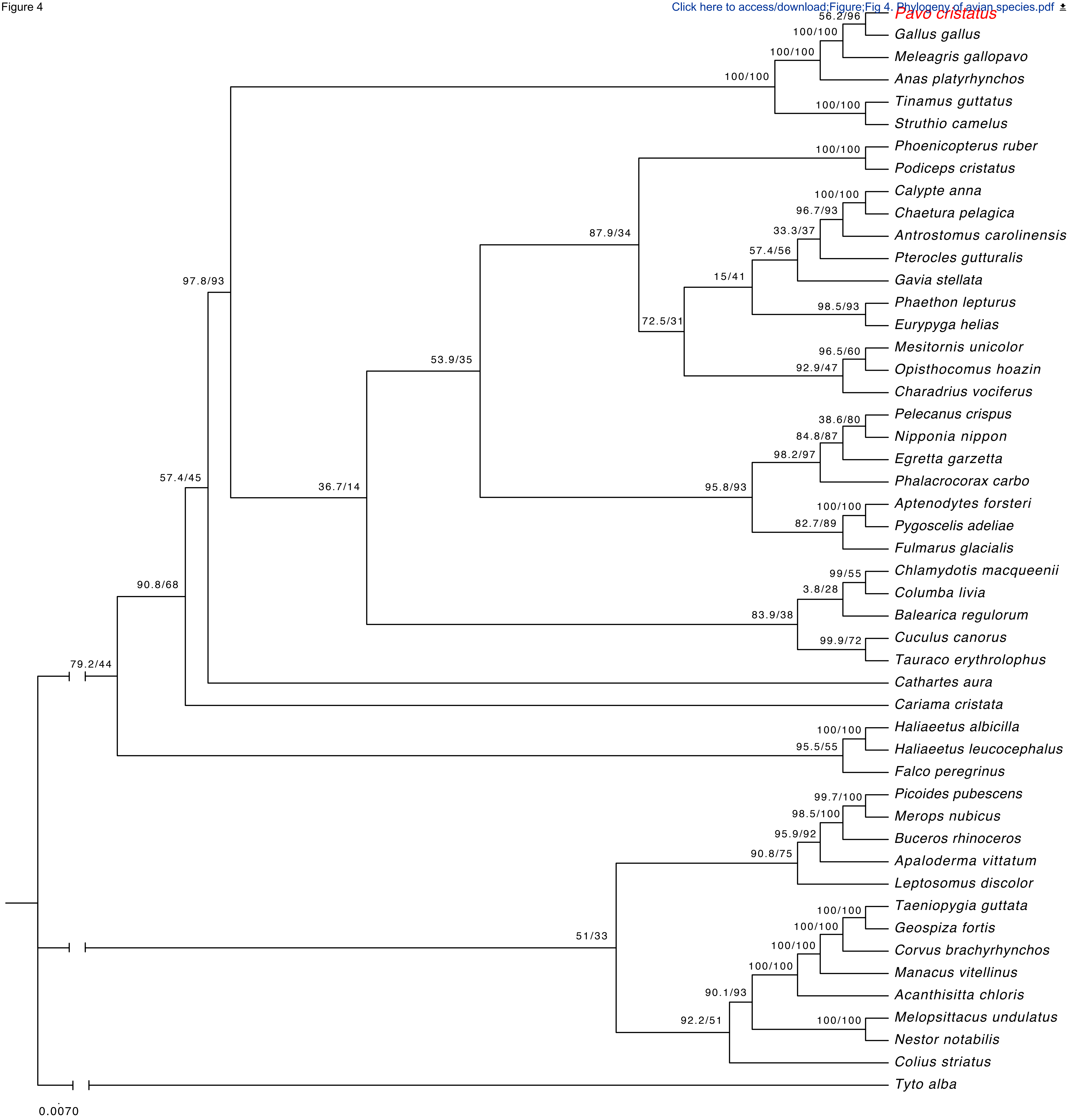

Figure 5

Click here to access/download;Figure;Fig 5. Unique Pfam domains common with chicken

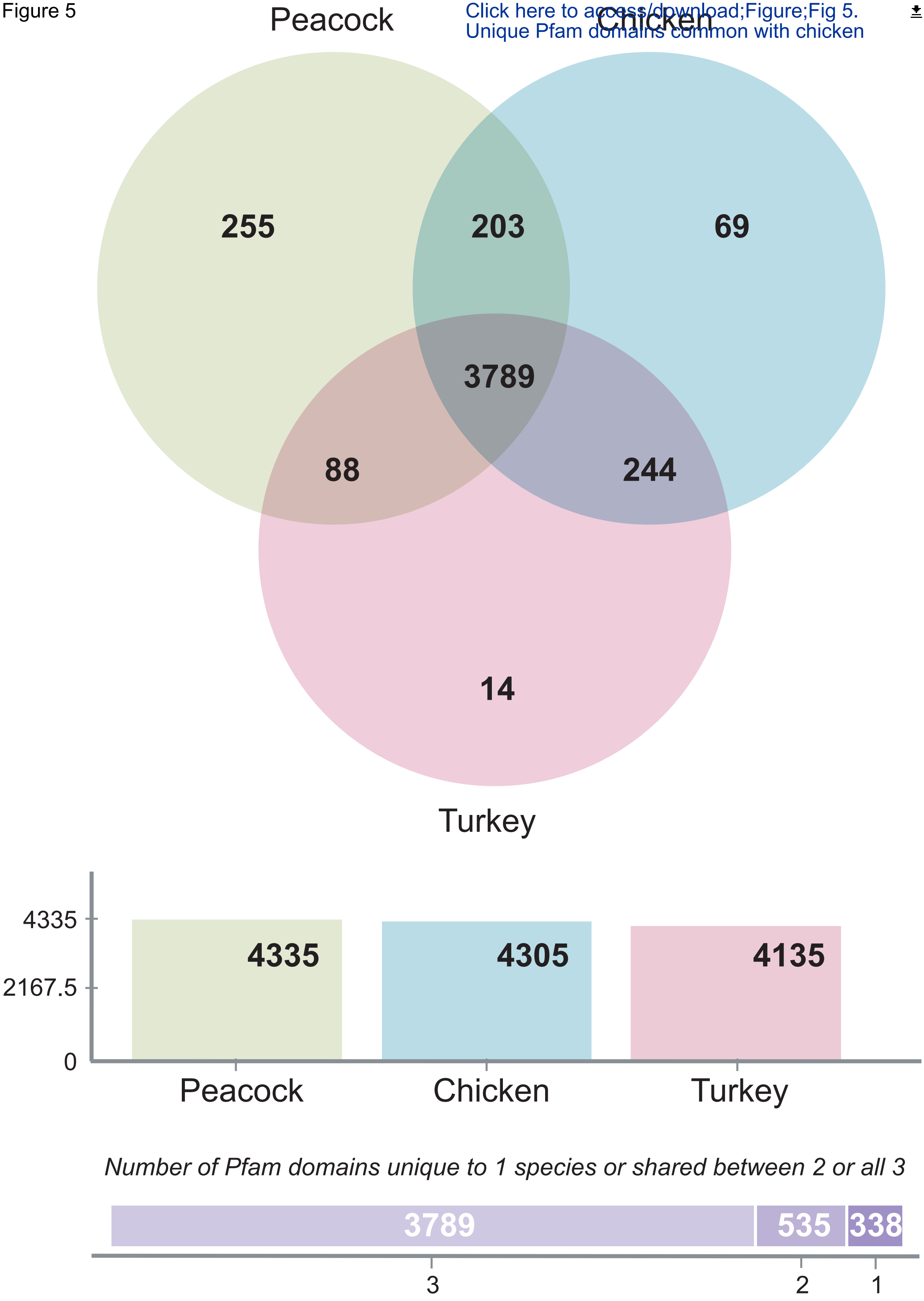

Figure 6

Click here to access/download;Figure;Fig 6. Heatmap

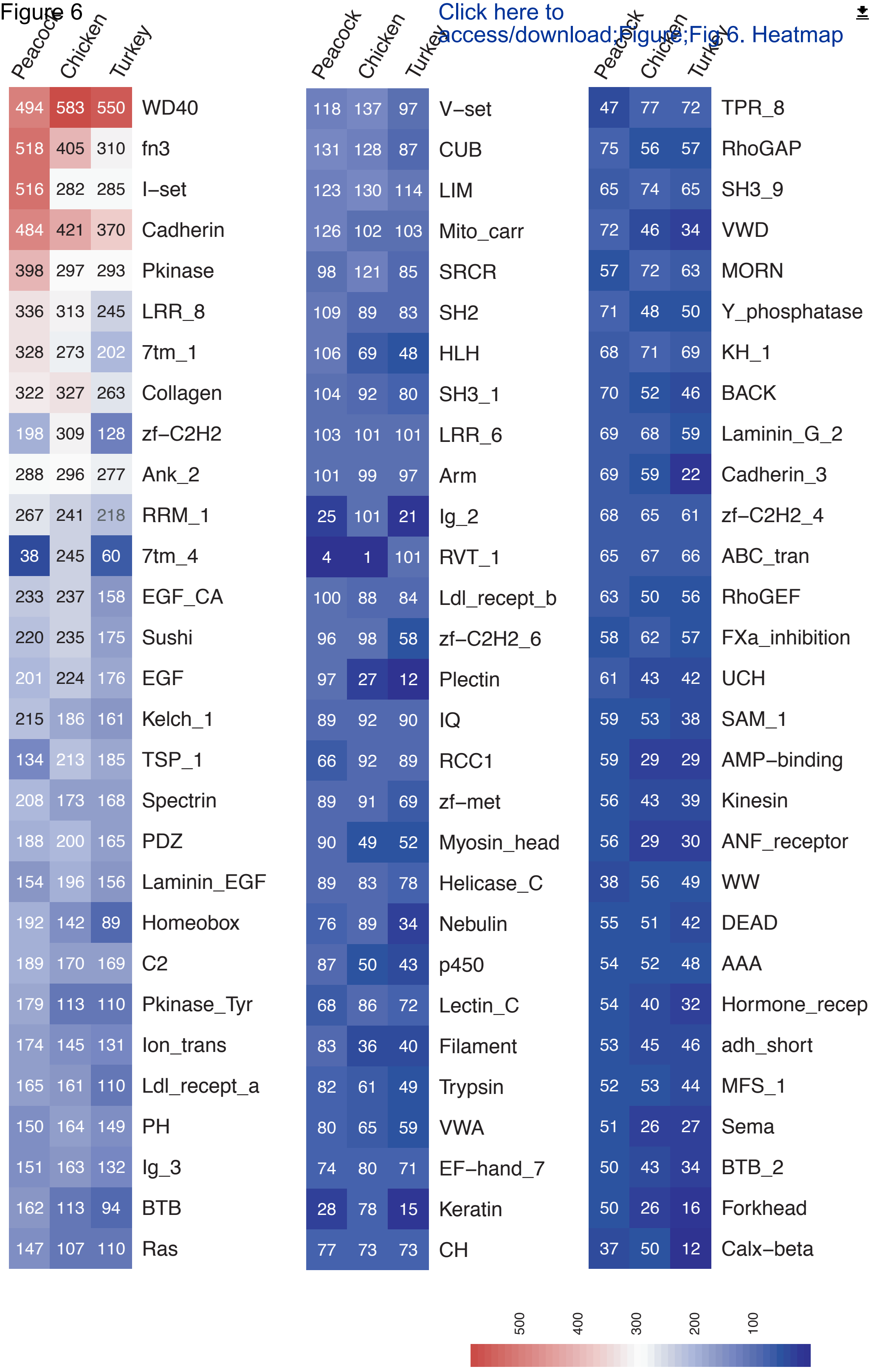

Figure 7

Click here to access/download;Figure;Fig 7. Venn of annotations.pdf

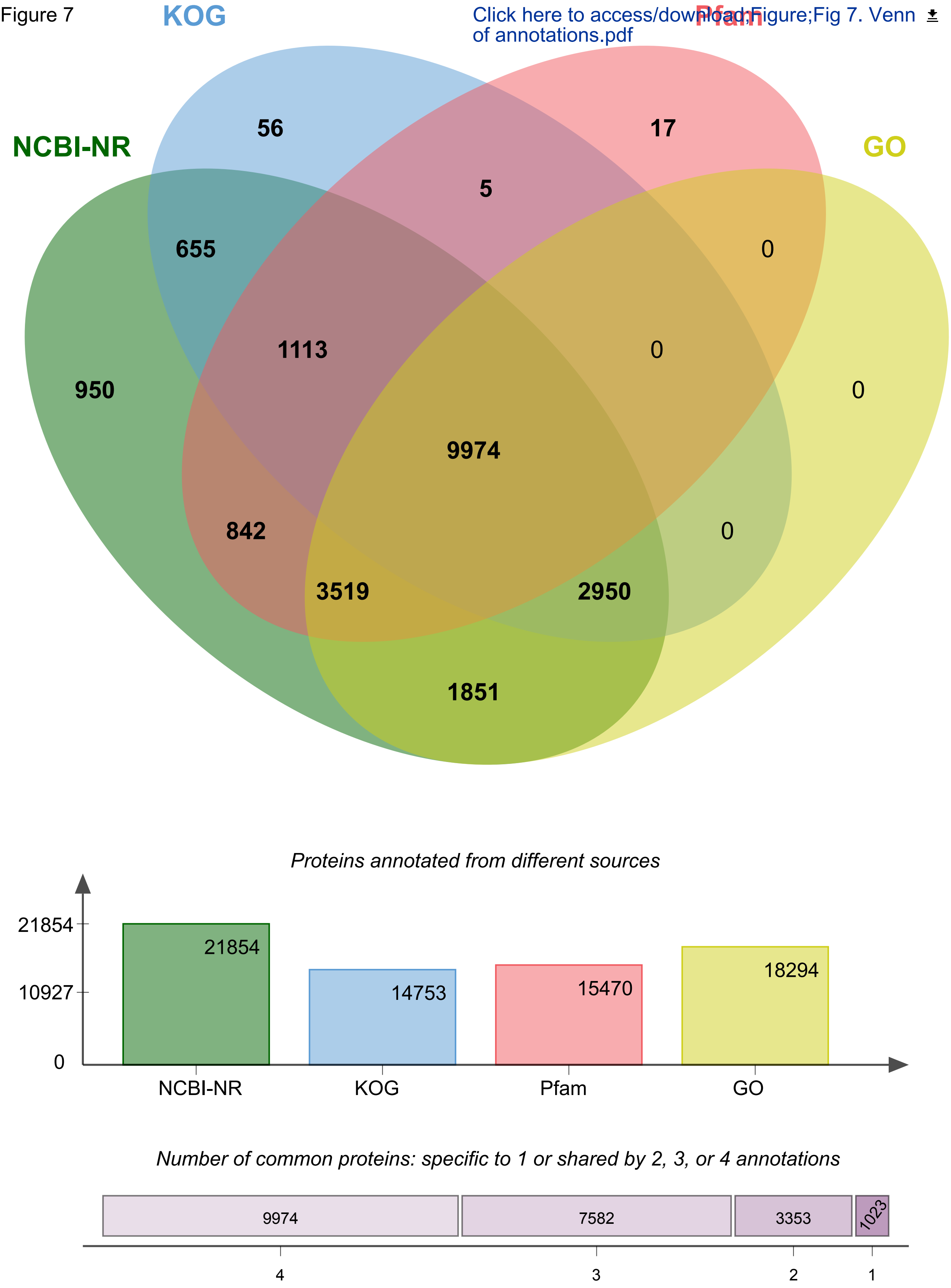

Figure 8

Click here to access/download;Figure;Fig 8. Peacock scaffolds against Gallus circular synteny.png

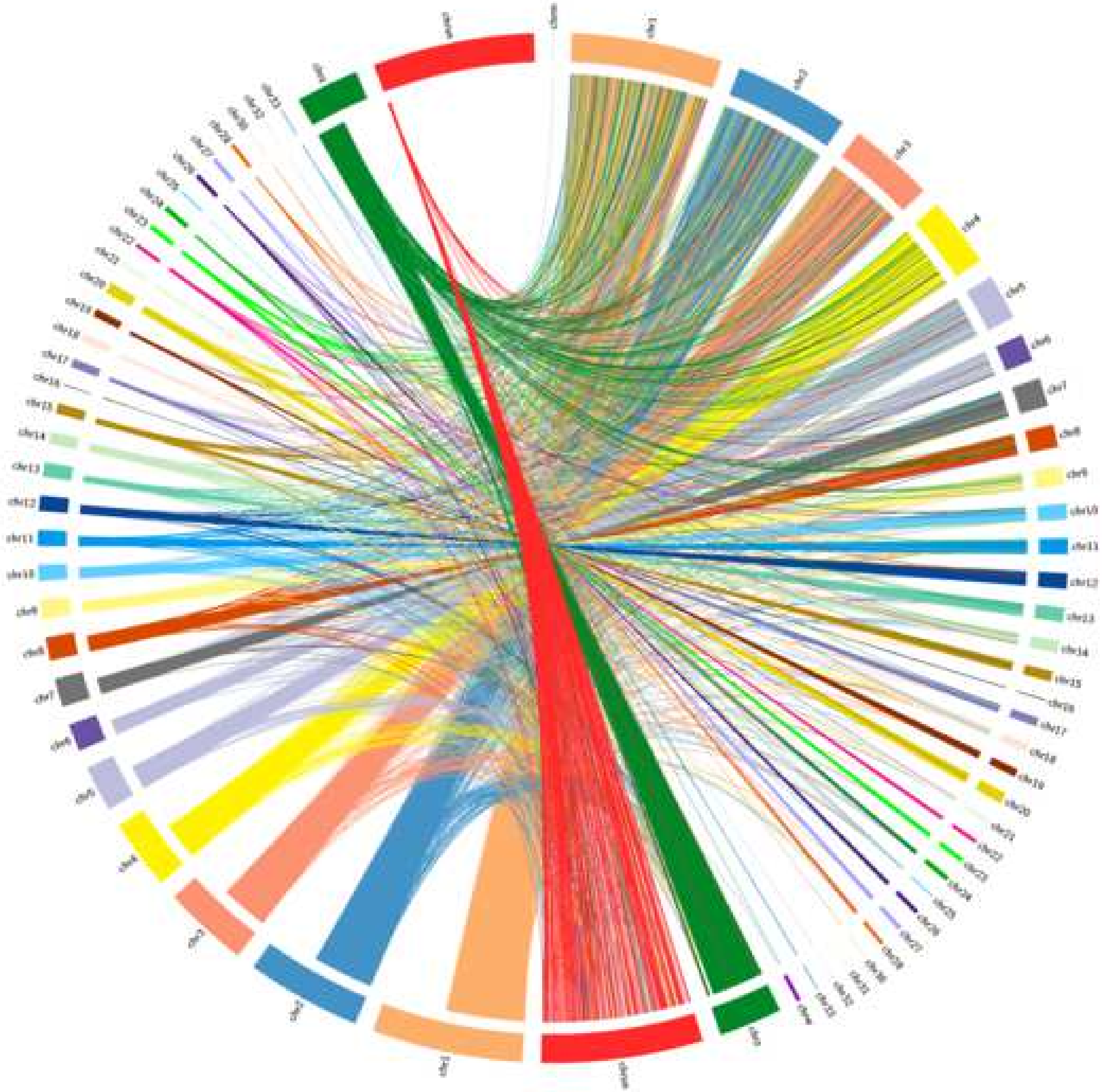

Click here to access/download
Supplementary Material
Supplementary_Description of all the tables and figures.docx

Click here to access/download
Supplementary Material
Table_S1_ReadStats_Table_S2_TEs.xlsx

Supplementary Material: Table S3

Click here to access/download

Supplementary Material

Table_S3_Repeats.xlsx

Supplementary Material: Table S4

Click here to access/download
Supplementary Material
Table_S4_Gene_annotations_of_peacock_proteins.xlsx

Supplementary Material: Table S5

Click here to access/download
Supplementary Material
Table_S5_KEGG_annotation.xlsx

Supplementary Material: Table S6

Click here to access/download
Supplementary Material
Table_S6_KOG_annotation.xlsx

Click here to access/download
Supplementary Material
Table_S7_BlastVsHumanProteins.xlsx

Supplementary Material: Table S8

Click here to access/download
Supplementary Material
Table_S8_Orthologous_proteins

Supplementary Material: Table S9

Click here to access/download
Supplementary Material
Table_S9_Pfam_Analysis.xlsx

Click here to access/download
Supplementary Material
Table_S10_Bird_Species_with_counts.xlsx

Click here to access/download
Supplementary Material
Fig S1. Proteins showing similarity to Pfam domains.pdf

Supplementary Material: Figure S2

Click here to access/download
Supplementary Material
Fig S2. Gene Ontololgy of top 10 WGS.png

Supplementary Material: Figure S3

Click here to access/download
Supplementary Material
Fig S3.Peacock vs Human_GO.pdf
