# Supplementary material for: De novo assembly of the Indian blue peacock (Pavo cristatus) genome using Oxford Nanopore technology and Illumina sequencing
Source: Gigascience. 2019 May 11;8(5):giz038. doi: 10.1093/gigascience/giz038 (PMC6511069; doi:10.1093/gigascience/giz038)
Supplement: GIGA-D-18-00280_Revision_3.pdf [file giz038_giga-d-18-00280_revision_3.pdf]

GigaScience

De novo genome assembly of the Indian Blue Peacock (Pavo cristatus), from Oxford Nanopore and Illumina sequencing

--Manuscript Draft--

**Manuscript Number:**	GIGA-D-18-00280R3
**Full Title:**	De novo genome assembly of the Indian Blue Peacock (Pavo cristatus), from Oxford Nanopore and Illumina sequencing
**Article Type:**	Data Note
**Funding Information:**	
**Abstract:**	Background<br>Pavo cristatus, the Indian peafowl are located in natural habitats of South Asia. The male blue peacock bird is known for its elegance, majestic looks and beauty. Since prehistoric times they have been described in Indian culture and has been adopted as the national bird of India. In this study, we present the first draft genome sequence of the peacock using Illumina and Oxford Nanopore technologies (ONT).<br><br>Findings<br>ONT sequencing resulted in approximately 2.3-fold sequencing coverage, whereas Illumina generated 150 bp paired-end sequence data at 284.6-fold sequencing coverage from five libraries. Subsequently, we generated de-novo genome assembly of the peacock genome with a 0.915 Gigabases (Gb) with a scaffold N50 of 0.23 Megabases (Mb). We also predicted that the peacock genome contains 23,153 protein-coding genes and 75.3 Mb (7.33%) of repetitive sequences.<br><br>Conclusions<br>We report a high-quality genome assembly of the peacock using a hybrid assembly generated from Illumina and ONT sequencing platforms. Long read chemistry generated from ONT was found to be useful in addressing challenges related to de-novo assembly particularly at regions containing repetitive sequences that span longer than the read length, and which cannot be resolved using only short-read-based assembly. The contig assembly on the short reads from Illumina resulted in an N50 of 1639 bases, whereas using 2.3x coverage from ONT increased the N50 by nine fold to 14,749 bases. The initial contig assembly based on Illumina sequencing reads alone resulted in total of 685,241 contigs. Further scaffolding on assembled contigs using both Illumina and ONT sequencing reads resulted in a final assembly having 15,025 super scaffolds with a N50 of about 0.23 Mb. The completeness of our genome assembly was verified with the fact that 95% of proteins predicted by homology were matched to those submitted in public repository. Further in concordance with other phylogenetic studies, the avian phylogeny on the conserved genes showed P. cristatus being closest with Gallus gallus followed by Meleagris gallopavo and Anas platyrhynchos.
**Corresponding Author:**	Subhradip Karmakar, PhD<br>All India Institute of Medical Sciences<br>New Delhi, Delhi INDIA
**Corresponding Author Secondary Information:**	
**Corresponding Author's Institution:**	All India Institute of Medical Sciences
**Corresponding Author's Secondary Institution:**	
**First Author:**	Ruby Dhar
**First Author Secondary Information:**	
**Order of Authors:**	Ruby Dhar
	Ashikh Seethy

Powered by Editorial Manager® and ProduXion Manager® from Aries Systems Corporation

	Karthikeyan Pethusamy
	Vishwajeet Rohil
	Sunil Singh
	Kakali Purkayastha
	Sandeep Goswami
	Rakesh Singh
	Indrani Mukherjee
	Ankita Raj
	Tryambak Srivastava
	Sovon Acharya
	Balaji Rajashekhar, Ph.D
	Subhradip Karmakar, PhD
**Order of Authors Secondary Information:**	
**Response to Reviewers:**	Date: 6 Feb. 2019<br><br>Dear Dr. Scott,<br>Thank you for suggesting improvements in the article. We have addressed all the reviewer's comments. Below are point-by-point response for the corrections raised by the reviewers for the manuscript titled<br>"De-novo genome assembly of the Indian Blue Peacock (Pavo cristatus), from Oxford Nanopore and Illumina sequencing"<br><br>Page 2<br>1.Line 6-9: Remove the two sentences: "The findings from avian genomics …" to "next generation sequencing technologies"<br>Reply: The two sentences from "The findings from avian genomics …" to "next generation sequencing technologies" are removed.<br><br>2.Lines 13: Recommend remove the sentence: "For the first time in avian genomics", since it is redundant with the sentence immediate preceding it.<br>Reply: The sentence starting from "For the first time in avian genomics" is removed.<br><br>3.Line 19: Recommend change "75,315,566" bp to "75.3 Mb"<br>Reply: The text is changed from "75,315,566" bp to "75.3 Mb"<br><br>Page 3<br>4.Line 6: Replace "reliability" with "completeness"<br>Reply: The word is replaced from "reliability" with "completeness"<br><br>5.Line 8-10: This sentence should be re-written to comment on the significance of this result. Is this expected or unexpected?<br>Reply: The sentence is modified as "Further in concordance with other phylogenetic studies, the avian phylogeny on the conserved genes showed P. cristatus being closest with Gallus gallus followed by Meleagris gallopavo and Anas platyrhynchos"<br><br>Page 4<br>6.Line 5: Need citation(s) for peacock references in ancient Indian literatures. Probably secondary scholarly works and not primary (ancient Indian) literature references.<br>Reply: Reference Kadgoankar, 1993 have been added here.<br><br><br>7.Line 14: In the version that I reviewing there is an empty line between the paragraph that ends, "within the orthologous regions [8]", and the paragraph that begins, "Despite the wealth of information".<br>Reply: The sentence got removed unintentionally in our previous version, we have included the missing sentence in this version.

Powered by Editorial Manager® and ProduXion Manager® from Aries Systems Corporation

8.Line 20: Replace “construction” with “sequencing” or “assembly”
Reply: The word “construction” is replaced with “assembly”

Page 5
9.Line 10: Change to “A ReliaPrep™”.
Reply: changed to “A ReliaPrep™”

Page 6
10.Line 21: Remove “accurately”
Reply: The word “accurately” is removed

Page 7
11.Line 1-5: This paragraph needs to be rewritten in the past tense to match the rest of the section. “had” instead of “has”, “was” instead of “is”.
Reply: In the paragraph lines 1-5 the sentences are modified in past tense.

Page 8
12.Line 13: “The library mix”
Reply: The sentence modified as “The library mix”

13.Line 14: “The eluted library”
Reply: The sentence modified as “The eluted library”

14.Line 15: “The whole genome library was prepared” or “The whole genome libraries were prepared”
Reply: The sentences is modified as “The whole genome libraries were prepared”

15.Line 22: “bcl2fastq (Illumina)”
Reply: The text is modified to bcl2fastq (Illumina)

16.Line 24: The citation, “(Andrews, S., 2010)” is in the wrong style.
Reply: “(Andrews, S., 2010)” is changed to citation [10] and in reference the following citation is added “Andrews, S., 2010. FastQC: a quality control tool for high throughput sequence data.”

Page 9
17.The phrase “Oxford Nanopore” needs to be replaced with “ONT” or whichever abbreviation the authors choose to use.
Reply: the text “Oxford Nanopore” is changed to “ONT”

Page 10
18.Line 3: “hard masked with the G. gallus repeat library using Repeatmasker (www.repeatmasker.org/).” Proper citation for Repeatmasker is found here: http://repeatmasker.org/faq.html#faq3.
Reply: The reference “Smit, AFA, Hubley, R and Green, P. RepeatMasker Open-4.0. 2013-2015 http://www.repeatmasker.org” is modified

19.Line 6: Replace “obtained” with “identified”
Reply: The word “obtained” is replaced with “identified”

Powered by Editorial Manager® and ProduXion Manager® from Aries Systems Corporation

20.Line 10: Proper citations for Augustus found here:
http://augustus.gobics.de/references
Reply: the reference is replaced with "[]Stanke, M., Diekhans, M., Baertsch, R. and Haussler, D., 2008. Using native and syntenically mapped cDNA alignments to improve de novo gene finding. Bioinformatics, 24(5), pp.637-644. (http://augustus.gobics.de/)"

21.Line 18: Proper citation for JGI portal https://genome.jgi.doe.gov/pages/citeUs.jsf
Reply: the reference is replaced with "Nordberg, H., Cantor, M., Dusheyko, S., Hua, S., Poliakov, A., Shabalov, I., Smirnova, T., Grigoriev, I.V. and Dubchak, I., 2013. The genome portal of the Department of Energy Joint Genome Institute: 2014 updates. Nucleic acids research, 42(D1), pp.D26-D31."

22.Line 24: The URLs for Pfam-A database and Pfam scan tools are out of date. Proper citation for Pfam is at the bottom of this page: http://pfam.xfam.org/.
Reply: the reference is replaced with "El-Gebali, S., Mistry, J., Bateman, A., Eddy, S.R., Luciani, A., Potter, S.C., Qureshi, M., Richardson, L.J., Salazar, G.A., Smart, A. and Sonnhammer, E.L.L., 2018. The Pfam protein families database in 2019. Nucleic Acids Research, 47(D1), pp.D427-D432."

Page 11
23.Line 6: There are two papers at the top of this web page (http://avian.genomics.cn/en/jsp/database.shtml) that should be cited as sources for this data.
Reply: Replaced as "avian phylogenomics project []"
And the following citation added
"Zhang, G., Li, B., Li, C., Gilbert, M.T.P., Jarvis, E.D. and Wang, J., 2014. Comparative genomic data of the Avian Phylogenomics Project. GigaScience, 3(1), p.26.
Jarvis, E.D., Mirarab, S., Aberer, A.J., Li, B., Houde, P., Li, C., Ho, S.Y., Faircloth, B.C., Nabholz, B., Howard, J.T. and Suh, A., 2015. Phylogenomic analyses data of the avian phylogenomics project. GigaScience, 4(1), p.4.
"

24.Lines 17,18,20,21 There are papers that should be cited for clustal, Gblock, Phylip and IQ-tree. I don't see them cited here. The papers are listed on the tools' websites.
Reply: Following citation were added,
"Larkin, M.A., Blackshields, G., Brown, N.P., Chenna, R., McGettigan, P.A., McWilliam, H., Valentin, F., Wallace, I.M., Wilm, A., Lopez, R. and Thompson, J.D., 2007. Clustal W and Clustal X version 2.0. bioinformatics, 23(21), pp.2947-2948.
Talavera, G. and Castresana, J., 2007. Improvement of phylogenies after removing divergent and ambiguously aligned blocks from protein sequence alignments. Systematic biology, 56(4), pp.564-577.
Felsenstein, J. 1989. PHYLIP - Phylogeny Inference Package (Version 3.2). Cladistics 5: 164-166.
L.-T. Nguyen, H.A. Schmidt, A. von Haeseler, B.Q. Minh, 2015. IQ-TREE: A fast and effective stochastic algorithm for estimating maximum likelihood phylogenies. Mol. Biol. Evol., 32:268-274."

Page 12
25.Line 8: Papers to cite when using LAST: http://last.cbrc.jp/doc/last-papers.html
Reply: Following citation was added, "Frith, M.C. and Kawaguchi, R., 2015. Split-alignment of genomes finds orthologies more accurately. Genome biology, 16(1), p.106." is added

26.Line 12: Paper to cite when using Circos: If you are using Circos, please cite us: Krzywinski, M. et al. Circos: an Information Aesthetic for Comparative Genomics. Genome Res (2009) 19:1639-1645 | download citation
Reply: Following citation was added "Krzywinski, M.I., Schein, J.E., Birol, I., Connors, J., Gascoyne, R., Horsman, D., Jones, S.J. and Marra, M.A., 2009. Circos: an

Powered by Editorial Manager® and ProduXion Manager® from Aries Systems Corporation

information aesthetic for comparative genomics. Genome research, 19(9):1639-45."

Page 13
27.Line 4: Remove "(mega base)".
Reply: The text "(mega base)" is removed.
28.Line 9: Change "1.02 GB (giga base)" to "1.02 Gb", remove the "(giga base)".
Reply: The text "1.02 GB (giga base)" is changed to "1.02 Gb"

29.Line 11: Change ">=1 Mbp" to ">= 1 Mb".
Reply: The text ">=1 Mbp" is changed to ">= 1 Mb".

30.Line 16: In accordance with my prior comment, change "75,315,566 bp" to "75 Mb".
Reply: The text is changed from "75,315,566 bp" to "75 Mb"

31.Line 17: Change 56,511,635 bp to "56 Mb".
Reply: The text is changed from "56,511,635 bp" to "56 Mb".

Page 14
32.Line 14: Change to "The detailed annotations".
Reply: The text is changed to "The detailed annotations"

33.Line 23: "humans" not "Humans"
Reply: The word is changed to "human"

Page 15
Page 16
34.Line 3: "Zn" to "zinc"
Reply: The text is changed from "Zn" to "zinc"

35.Line 16: "de-novo", is italicized and not hyphenated elsewhere in the manuscript, except for Line 19 of Page 4. Needs to be consistent. Probably should use "de novo".
Reply: We have used "de-novo" instead of "de novo" in the manuscript.

36.Line 16-26: This whole paragraph needs some citations, especially for the claim about different technologies improving genome assemblies. Even though you have data that supports this claim and demonstrate ONT's use in bird genomes for the first time, this idea has been discussed before and has been the basis of at least one genome assembler in the past (eg AllPaths LG) and the topic of several reviews (see Metzker, Nature Reviews Genetics, 2010).
Reply: Citations relevant to the sentences have been included in this paragraph.

Page 17
37.Line 15: "95% homology" implies that 95% of the nucleotides match between sequences, which I don't is demonstrated in this figure. If the claim is that 95% of the annotated (or predicted; it's not clear in the text which set of peacock genes is meant) had a match then that should be made clearer. Looking in the abstract, I see the sentence, "The reliability of our genome assembly was verified with the fact that 95% of proteins predicted by homology were matched to those submitted in public repository." That claim matches more closely the message communicated by Figure 7, so I would re-write this sentence to match the abstract.
Reply: The sentence is now modified as "The confidence on the predicted peacock proteins got strengthened when about 95% of them showed significant homology to various genomic features from different databases (Fig. 7)."

Powered by Editorial Manager® and ProduXion Manager® from Aries Systems Corporation

	Page 18<br>38.Line 8: “Figures, Gene ontology and annotations”. This sentence fragment needs to be re-written.<br>Reply: The section is rewritten as “Additional figures included are the Peacock, Chicken and Turkey proteins showing similarity to Pfam domains, top ten Gene ontology annotations in Biological process; Cellular component and Molecular function from the Peacock proteins, and Peacock homologous proteins in humans.”<br><br>Page 21<br>39.Lines 16-20: This figure caption needs to be shortened, since it is partially a re-write of this section from methods. Could be re-written as “Circular image of the assembled peacock genome aligned against the G. gallus genome. The right side of the image represents the reference chicken genome and left side of the image represents the peacock genome.”<br>Reply: The text has been shortened as suggested.<br><br>Page 22<br>40.Line 13: Reference 5 here, Kadgoankar, 1993, is not used in the text and provides the information that I said was missing on Page 4, Line 5.<br>Reply: The missing citation has been added.<br><br>41.In the reference list in general, “p” or “pp” is missing from several references (reference 12, 13, 17, maybe others) and should be made consistent throughout.<br>Reply: We have used reference manager software to make all references consistent.
**Additional Information:**	
**Question**	**Response**
Are you submitting this manuscript to a special series or article collection?	No
**Experimental design and statistics**<br><br>Full details of the experimental design and statistical methods used should be given in the Methods section, as detailed in our Minimum Standards Reporting Checklist. Information essential to interpreting the data presented should be made available in the figure legends.<br><br>Have you included all the information requested in your manuscript?	Yes
**Resources**<br><br>A description of all resources used, including antibodies, cell lines, animals and software tools, with enough information to allow them to be uniquely identified, should be included in the Methods section. Authors are strongly	Yes

encouraged to cite Research Resource Identifiers (RRIDs) for antibodies, model organisms and tools, where possible.<br><br>Have you included the information requested as detailed in our Minimum Standards Reporting Checklist?	
**Availability of data and materials**<br><br>All datasets and code on which the conclusions of the paper rely must be either included in your submission or deposited in publicly available repositories (where available and ethically appropriate), referencing such data using a unique identifier in the references and in the “Availability of Data and Materials” section of your manuscript.<br><br>Have you have met the above requirement as detailed in our Minimum Standards Reporting Checklist?	Yes

Powered by Editorial Manager® and ProduXion Manager® from Aries Systems Corporation

Manuscript

Click here to access/download;Manuscript;Manuscript_revision_06022019_

Click here to view linked References

De novo genome assembly of the Indian Blue Peacock (*Pavo cristatus*), from Oxford Nanopore and Illumina sequencing

Authors: Ruby Dhar[1], Ashikh Seethy[1], Karthikeyan Pethusamy[1], Vishwajeet Rohil[2], Sunil Singh[1], Kakali Purkayastha[2], Sandeep Goswami[1], Rakesh Singh[3], Indrani Mukherjee[1], Ankita Raj[1], Tryambak Srivastava[1], Sovon Acharya[1], Balaji Rajashekhar[4,5,]* and Subhradip Karmakar[1,]*

Affiliation: [1]Department of Biochemistry, AIIMS, New Delhi, India. [2]Vallabhbhai Patel Chest Institute (VPCI), New Delhi, India. [3]Kanpur Zoo, Kanpur, India. [4]Celixa, Bangalore, India. and [5]Institute of Computer Science, University of Tartu, 50409 Tartu, Estonia

*Corresponding Authors email: balaji@ut.ee, subhradip.k@aiims.edu

Running Title: De novo Genome Assembly of the Peacock Bird

Key words: Peacock, *Pavo cristatus*, Indian National Bird, Genome Assembly, Oxford Nanopore.

Abstract

Background

Pavo cristatus, the Indian peafowl are located in natural habitats of South Asia. The male blue peacock bird is known for its elegance, majestic looks and beauty. Since prehistoric times they have been described in Indian culture and has been adopted as the national bird of India. In this study, we present the first draft genome sequence of the peacock using Illumina and Oxford Nanopore technologies (ONT).

Findings

ONT sequencing resulted in approximately 2.3-fold sequencing coverage, whereas Illumina generated 150 bp paired-end sequence data at 284.6-fold sequencing coverage from five libraries. Subsequently, we generated de novo genome assembly of the peacock genome with a 0.915 Gigabases (Gb) with a scaffold N50 of 0.23 Megabases (Mb). We also predicted that the peacock genome contains 23,153 protein-coding genes and 75.3 Mb (7.33%) of repetitive sequences.

Conclusions

We report a high-quality genome assembly of the peacock using a hybrid assembly generated from Illumina and ONT sequencing platforms. Long read chemistry generated from ONT was found to be useful in addressing challenges related to de novo assembly particularly at regions containing repetitive sequences that span longer than the read length, and which cannot be resolved using only short-read-based assembly. The contig assembly on the short reads from Illumina resulted in an N50 of 1639 bases, whereas using 2.3x coverage from ONT increased the N50 by nine fold to 14,749 bases. The initial contig assembly based on Illumina sequencing reads alone resulted in total of 685,241 contigs. Further scaffolding on assembled contigs using both Illumina and ONT sequencing reads resulted in a final

assembly having 15,025 super scaffolds with a N50 of about 0.23 Mb. The completeness of our genome assembly was verified with the fact that 95% of proteins predicted by homology were matched to those submitted in public repository. Further in concordance with other phylogenetic studies, the avian phylogeny on the conserved genes showed *P. cristatus* being closest with *Gallus gallus* followed by *Meleagris gallopavo* and *Anas platyrhynchos*.

Introduction

Pavo cristatus commonly known as the Indian blue peafowl are native to South Asian countries. Apart from the wild, they are usually found as exhibits in park and zoo, besides being raised for breeding and conservation purposes [1, 2] (Fig. 1). The peacock has been widely referred in ancient Indian literatures [3]. They have been found to be closely associated with the life and culture of the people from South East Asia, symbolizing beauty, love, grace and pride [4, 5]. Owing to these, the peacock obtained the status as the National Bird of India in 1963.

Genome sequencing of the avian model organism *Gallus gallus* (red junglefowl the chicken) [6], as well as variety of other avian species [7] have provided a novel perspective on vertebrate genome evolution. This enabled us to understand the genome structure better and annotate the mammalian genome. Genome studies of *G. gallus* with respect to the human have revealed an extremely high level of conservation within the orthologous regions [8] thus promising of being a good candidate for studies on developmental biology, immunology and vertebrate genome architecture [9, 10].

Despite the wealth of information from the existing avian genome sequencing projects, it is still important to sequence genome of other new species to add value, both into avian and vertebrate genomics. For the first time in avian genomics, Oxford Nanopore technology (ONT or Nanopore) has been used to sequence a bird genome presented in this study. Long reads have been helpful during the de novo assembly of the genome especially in the GC rich repeat regions which invariably poses serious challenges in genome assembly. Comparative genomics with other birds will help in understanding the uniqueness of peacock genome, development of this species, complex plumage pigmentation, sexual dimorphism and its evolutionary relationships with other birds. The characterization of the genes and association

with specific function will provide better understanding of the peafowl species. The protein comparisons among the peacock, chicken and *Meleagris gallopavo* (domestic turkey) will reveal conserved domains and functional annotations that are common and absent among these species.

Materials and methods

Sample collection and extraction of DNA

The whole blood of male peacock was collected from Kanpur zoo, India after obtaining the necessary ethical and institutional approval. Approximately, 20 µl of proteinase K (PK) solution was taken into a 1.5 ml microcentrifuge tube, 200 µl of blood was added and briefly mixed. Furthermore, 200 µl of cell lysis buffer was added to the tube, mixed by vortexing for 10 seconds, incubated at 56°C for 10 minutes. ReliaPrep™ Binding Column was placed into an empty collection tube. Furthermore, 250 µl of Binding Buffer (BBA) was added to the tube, and mixed by vortexing for 10 seconds with a vortex mixer. Contents of the tube were added to the A ReliaPrep™ binding column, capped and placed in a refrigerated microcentrifuge. These were then centrifuged for 1 minute at maximum speed and flow through was discarded. Binding column was placed into a fresh collection tube. In addition, 500 µl of column wash solution was added to the column and centrifuged for 3 minutes at maximum speed; flow through was again discarded. Column washing is repeated thrice. Columns were then placed in a nuclease free clean 1.5 ml microcentrifuge tube. Furthermore, 100 µl of Nuclease-Free Water was then added to the column and centrifuged for an additional 1 minute at maximum speed. Column was discarded and elute was saved. The concentration and purity of the extracted DNA was evaluated using Nanodrop Spectrophotometer (Thermo Scientific) and Qubit flurometer and integrity was checked on a

0.8% agarose gel. The DNA sample was aliquoted for library preparation on two different platforms: Illumina HiSeq4000 and Oxford Nanopore Technologies (ONT).

Library preparation and sequencing

A. Paired-End library preparation and sequencing

Whole genome sequencing (WGS) libraries were prepared with Illumina-compatible NEXTflex DNA sequencing kit (BIOO Scientific, Austin, TX, USA). Approximately, 1 μg of genomic DNA was sheared using Covaris S2 sonicator (Covaris, Woburn, MA, USA) to generate approximate fragment size distribution from 300 - 600 basepair (bp). The fragment size distribution was checked on Agilent 2200 Tape Station with D1000 DNA screen tapes and reagents (Agilent Technologies, Palo Alto, CA, USA) and subsequently purified using HighPrep magnetic beads (Magbio Genomics Inc, USA). The purified fragments were end-repaired, adenylated and ligated to Illumina multiplex barcode adaptors as per NEXTflex DNA sequencing kit protocol (BIOO Scientific, Austin, TX, USA).

The adapter-ligated DNA was purified with HighPrep beads (MagBio Genomics, Inc, Gaithersburg, MD, USA) and then size selected on 2% low melting agarose gel and cleaned using MinElute column (QIAGEN). The resultant fragments were amplified for 10 cycles of PCR using Illumina-compatible primers provided in the NEXTFlex DNA sequencing kit. The final PCR product (sequencing library) was purified with HighPrep beads, followed by library quality control check. The Illumina-compatible sequencing library was initially quantified by Qubit fluorometer (Thermo Fisher Scientific, MA, USA) and its fragment size distribution was analyzed on Agilent TapeStation. Finally, the sequencing library was quantified by quantitative PCR using Kapa Library Quantification Kit (Kapa Biosystems,

Wilmington, MA, USA). The qPCR-quantified library was subjected to sequencing on an Illumina sequencer for 150 bp paired-end chemistry.

The Illumina-compatible sequencing library for the samples had a fragment size range between 275 - 425 bp for Paired-End Short Insert (PE-SI) and 350 - 650 bp for Paired-End Long Insert (PE-LI). As the combined adapter size was approximately 120 bp, the effective user-defined insert size was 155 - 305 bp and 230 - 530 bp for PE-SI and PE-LI, respectively. Libraries were sequenced in Illumina HiSeq platform with 150 PE chemistry.

B. Mate-Pair library preparation and sequencing

Mate Pair sequencing library was prepared with Illumina-compatible Nextera Mate Pair Sample Preparation Kit (Illumina Inc., Austin, TX, USA). Approximately, 4 ug of genomic DNA was simultaneously fragmented and tagged with Mate Pair adapters in a transposon-based tagmentation step. Tagmented DNA was then purified using AMPure XP Magnetic beads (Beckman Coulter Life Sciences, Indianapolis, IN, USA) followed by strand displacement to fill gaps in the tagmented DNA. Strand displaced DNA was further purified with AMPure XP beads before size-selecting the 3 - 5 kilobases (Kb), 5 - 7 Kb & 7 - 10 Kb fragments on low melting agarose gel. The fragments were circularized in an overnight blunt-end intra-molecular ligation step, which will result in circularization of DNA with the insert mate pair adapter junction. The circularized DNA was sheared using Covaris S220 sonicator (Covaris, Woburn, MA, USA) to generate approximate fragment size distribution from 300 - 1000 bp. The sheared DNA was purified to collect the mate pair junction positive fragments using Dynabeads M-280 Streptavidin Magnetic beads (Thermo Fisher Scientific, Waltham, MA, USA). The purified fragments were end-repaired, adenylated and ligated to Illumina multiplex barcode adaptors as per Nextera Mate Pair Sample Preparation Kit protocol.

The adapter-ligated DNA was then amplified for 15 cycles of PCR using Illumina-compatible primers. The final PCR product (sequencing library) was purified with AMPure XP beads, followed by library quality control check. The Illumina compatible sequencing library was initially quantified by Qubit fluorometer (Thermo Fisher Scientific, MA, USA), and its fragment size distribution was analyzed on Agilent TapeStation. Finally, the sequencing library was accurately quantified by quantitative PCR using Kapa Library Quantification Kit (Kapa Biosystems, Wilmington, MA, USA). The qPCR quantified libraries were pooled in equimolar amounts to create a final multiplexed library pool for sequencing on an Illumina sequencer.

C. Oxford Nanopore MinION library preparation and sequencing

Genomic DNA (1.5μg) was end-repaired (NEBnext ultra II end repair kit, New England Biolabs, MA, USA), cleaned up with 1x AmPure beads (Beckmann Coulter, USA). Adapter ligations were performed for 20 minutes using NEB blunt/TA ligase (New England Biolabs, MA, USA). The library mix were cleaned up using 0.4X AmPure beads (Beckmann Coulter, USA) and eluted in 25 μl of elution buffer. The eluted library was used for sequencing. The whole genome libraries were prepared by using ligation sequencing SQK-LSK108 Oxford Nanopore sequencing kit (ONT, Oxford, UK). Sequencing was performed on MinION Mk1b (ONT, Oxford, UK) using SpotON flow cell (FLO-MIN106) in a 48 hour sequencing protocol on MinKNOW (1.1.20 from ONT).

Raw data quality control and processing

A. Illumina raw data quality control and processing

The Illumina reads were de-multiplexed using bcl2fastq (Illumina). The Illumina generated raw data for genomic libraries was quality checked using FastQC (http://www.bioinformatics.babraham.ac.uk/projects/fastqc/) [11]. The paired-end Illumina reads were processed for clipping the adapter and low-quality bases using customized script which retains minimum 70% bases/reads with Phred score (Q≥30 in each base position) with a read length of 50 bp. The MP libraries were trimmed for adapter and low-quality base trimming from the 3'-end using PLATANUS internal trimmer (http://platanus.bio.titech.ac.jp/) [12].

B. Oxford Nanopore reads base calling and processing

The raw data were then base-called with the cloud-based Metrichor workflow 2D Basecalling plus Barcoding by Metrichor (V.2.43.1 from ONT, https://nanoporetech.com/products/metrichor). The Oxford Nanopore reads were processed using Poretools [13] for converting fast5 files to fasta format. For further quantification and analysis the 2D reads or 1D high quality reads were selected for further assembly.

De novo genome assembly and genome size estimation

The quality checked Oxford Nanopore reads were error-corrected using Illumina PE reads. For error-correction the Illumina PE-reads were aligned to the Nanopore reads by using BWA aligner [14]. The paired-end reads were assembled using Abyss [15] followed by contig extension using ONT reads using SSPACE-LongRead [16]. Super scaffolding of the assembled scaffold was performed using SSPACE [17] and PLATANUS (http://platanus.bio.titech.ac.jp/) using the Oxford Nanopore and Matepair data. Final draft genome resulted after gap closure by GAPCLOSER (http://sourceforge.net/projects/soapdenovo2/files/GapCloser/) and PLATANUS gap_close

tool (http://platanus.bio.titech.ac.jp/) using Illumina data. The genome size was estimated using a k-mer distribution plot using JELLYFISH [18]. The assembly and annotation workflow has been represented in Figure 2.

Identification of repetitive elements and SSR markers

Repetitive elements, retrotransposons and DNA transposons were identified in the draft genome and was hard masked by using reference genomic repeats of *G. gallus* using Repeatmasker tool [19]. Final assembled scaffolds were analysed for Simple Sequence Repeats (SSR) identification. SSRs like the di, tri, tetra, penta and hexa-nucleotide repeats in the genome were identified using MISA (Version 1.0.0) (http://pgrc.ipk-gatersleben.de/misa/).

Annotation of the draft genome

Gene models were predicted on a hard-masked draft genome using AUGUSTUS [20] with *G. gallus* as a reference model. The predicted proteins were annotated by using BLASTP [21] against the NCBI NR (non-redundant) database with default parameters at E-value cutoff of 1E-5.

The predicted proteins were searched against the KEGG-KAAS server (http://www.genome.jp/tools/kaas/) for pathway analysis [22]. *G. gallus*, *M. gallopavo*, *Taeniopygia guttata* (zebra finch), *Falco peregrinus* (peregrine falcon) were used as reference organism for pathway identification. The EuKaryotic Orthologous Groups (KOGs) [23] were predicted using homology-based approach.

Prediction of protein domains

Predicted proteins from peacock, chicken and turkey with sequence length greater than 100 amino acids were considered for protein domain analysis. All the protein sequences from each organism were searched against Pfam-A database using Pfam scan [24] for protein domain identification.

Identification of avian protein families

A total of 748,544 protein sequences from 49 avian species (including peacock proteins from this study) and others were downloaded from avian phylogenomics project [25, 26]. Sequences greater than 100 amino acids from all the avian genomes were selected and concatenated to a single fasta file. These sequences were clustered using CD-HIT [27] with 70% alignment coverage for the shorter sequence with a length difference cutoff of 0.7. The single copy gene family orthologs present across all avian species and not clustered peacock proteins were annotated.

Phylogenetic tree construction

For phylogenetic tree construction we considered single copy gene clusters present as single copy in all the avian species. These protein sequences from each species were concatenated and were further aligned by multiple sequence alignment tool Clustalw [28]. The poorly aligned positions and divergent regions were removed using Gblock tool [29]. The fasta format sequences were converted to phylip format using Phylip tool [30]. Phylogenetic trees were constructed using IQ-TREE version 1.5.6 [31]. The parameters used for phylogenetic tree construction were ultrafast boostrap (UFBoot, using the –bb option of 1000 replicates), and a standard substitution model (-st AA –m TEST) and alrt 1000 -nt AUTO was given for tree generation. The generated trees from IQ-TREE tool were visualized using Figtree (http://tree.bio.ed.ac.uk/software/figtree/) and the Brach-support values were recorded from

the output ".treefile". The trees were modified for better visualization under Trees section an increasing order nodes were applied.**Genome conservation analysis**

Draft chromosome visualizations were constructed by aligning the assembled peacock genome against the *G. gallus* with the Chromosomer tool (https://github.com/gtamazian/chromosomer). The reordered assembled genome was aligned against the chicken genome using LAST aligner [32] with NEAR (finding short-and-strong [near-identical] similarities) parameter allowing for substitution and gap frequencies, leading to the identification of orthologs. These query-mapped regions were filtered with a greater than 1% of the maximum length for visualization using Circos [33].

Results

Genome sequencing assessment

A total of five libraries from Illumina HiSeq technology of 150 bp paired-end were generated. The short-insert reads of 489,114,747 accounted to genome coverage of 146.7X and long-insert reads of 302,884,819 sequences was about 90.9X coverage with a total coverage of 237.6X. Sequencing of three mate-pairs of 3-5 Kb, 5-7 Kb of and 7-10 Kb yielded 72,915,033, 47,440,144 and 36,464,628 reads, respectively with an approximate coverage of 21.9X, 14.2X and 10.9X, respectively, with a grand total of 156 million mate-pair reads of 47X coverage. Oxford Nanopore technology was used to generate 366,323 long reads having of 2,398,560,283 bp with coverage of 2.3X. The complete genome sequencing was generated to a depth of ~287X from both Illumina and Oxford Nanopore platform (Table 1). The coverage was based on the assumption that the peacock genome size of about 1 Gb.

Genome assembly

The first assembly was performed on Illumina reads with Abyss de novo assembler that resulted in ~932 Mb of genome with an N50 of 1639 bp. The extension of the contigs were performed with Oxford Nanopore reads, which generated scaffolds with N50 of 14,748 bp. Super scaffolding of the assembled scaffold was performed using SSPACE and PLATANUS with MP libraries that generated ~916 Mb genome with the N50 value of 168,140 bp. The final gap closer was executed using GAPCLOSER program with MP and PE-LI libraries, which generated a draft genome of 1.02 Gb. The draft genome assembly of *Pavo cristatus* consists of 179,346 bp scaffolds, with a N50 of 189,886 bp with 37 scaffolds, having sequence length >=1 Mb. Contigs above 5000 bp have covered a genome of ~0.915 Mb with N50 0.23 Mb. In the assembled genome there were ~0.4% of non-ATGC characters (Table 2).

Repetitive genome elements and SSR markers

A total of 75 Mb (7.33%) of the peacock genome was estimated to consist of repeat sequences (Table S1). In the genome about 56 Mb (5.5%) of retrotransposons (class I) were identified as the NON-LTR elements (LINEs (4.7%), SINEs (0.08%) and LTR elements (0.72%). Subsequently, the DNA transposons (class II) of 7,277,390 bp (0.71%) and unclassified elements of about 467,719 (0.05%) were identified (Table S1). The median percentages of LINEs, SINEs, LTR, DNA, unknown and total masked bases of other avian birds were 3.94, 0.11, 1.31, 0.22, 0.85 and 6.93, respectively (Table S2).A total of 399,493 SSRs were obtained from the peacock genome assembly. The largest fraction of SSRs identified were mono-nucleotide (60.04%), followed by tetra-nucleotide (26%), di-nucleotide (8.51%), tri-nucleotide (4.31%), penta-nucleotide (1.03%) and finally hexa-nucleotide (0.13%). Among the SSRs identified, A (49.2%) and T (44.9%) accounted for 94.1% of the mono-nucleotide repeats. AT (23.8%), TA (16.5%), TG (13.7%), AC (10.6%) and CA

(10.32%) accounted for 75% of the di-nucleotide repeats, whereas TTG (9.9%), AAT (9.6%), AAC (9.4%), TTA (7.1%), ATT (4.5%), TAA (3.5%), CAA (3.1%) and GGA (2.69%) accounted for 49.7% of the tri-nucleotide repeats (Table S3).

Gene prediction and annotation

A total of 23,153 proteins were predicted from the assembled draft peacock genome using AUGUSTUS. Among them, 21,854 (94.4%) predicted proteins showed homology to other sequences from the NCBI NR database (Fig. 3). The top four organisms where the peacock proteins showed homology belonged to the *G. gallus* with 11,398 proteins, *M. gallopavo* with 4059 proteins, *Amazona aestiva* (blue-fronted Amazon parrot) with 1352 proteins and *Anas platyrhynchos* (mallard the duck) with 849 proteins. The detailed annotations of all the proteins are available in Table S4.

Gene Ontology (GO) descriptions were assigned for a total of 18,294 (79%) peacock proteins. Among them, 14,489 proteins have molecular function; 11,678 have biological process and 13,735 proteins have cellular component as functional categories (Table S4). A total of 4091 (17.7%) peacock proteins were found to have pathway information from the KEGG database (Table S5), whereas a total of 20,937 (88.1%) peacock proteins found a similarity against the KOG annotations (Table S6). The peacock proteins when searched against the human proteins showed gene family expansions (in cell morphogenesis, neuronal projection and development and GTPases (Table S7 and Fig. S3).

Analysis of avian protein families

A total of 748,544 protein sequences from 49 avian species have 653,497 protein sequences of length above 100 amino acids (Table S8A). Based on the level of identity CD-HIT

clustered all the proteins into a total of 114,121 gene clusters. Among them, 68 highly homologous gene clusters were present as single copy in all the 49 avian species (Table S8B and Table S8C). We also observed 13,860 protein clusters of peacock species not clustered with other avian species (Table S8D).

Phylogenetic analysis

The phylogenetic analysis of 48 avian species and the peacock proteins showed clustering of the *P. cristatus* species in a clade of *G. gallus*, *M. gallopavo*, *A. platyrhynchos*, *Tinamus guttatus* (white-throated tinamou) and *Struthio camelus* (ostrich). This is the largest clade with six species having a bootstrap support of a 100. In the aforementioned clade, except the mallard species all belong to flightless or low flying birds. The bootstrap support between *P. cristatus* and *G. gallus* were 96, followed by *M. gallopavo*of 100 bootstrap support (Fig. 4).

Comparison with other species and databases

Predicted proteins from peacock, chicken and turkey when searched for the conserved Pfam protein domains showed about 81% of the domains that were common among these three species (Fig. 5, Table S9). In comparison with the total Pfam domains from all the three species, 94%, 98.4% and 99.7% Pfam domains were present in peacock, chicken and turkey, respectively. However, 255, 69 and 14 Pfam domains were absent among the species comparisons, respectively (Table S9H).

There were 15,470 (78%), 12,794 (85%) and 11,745 (85%) of the peacock, chicken and turkey proteins found to contain a match to Pfam domains, respectively (Table S9). The domain comparisons among the species showed gene family expansions such as kinases, zinc finger proteins, GTPases and others in either one of the species (Fig. 6). Commonly, a total of 9974 peacock proteins were found to have annotation in all the four databases NCBI-NR,

KOG, Pfam and GO (Fig. 7). The assembled peacock genome when reordered for pseudo chromosomes generation against the masked 1.21 Gb chicken genome [34] showed a 597 MB reordered peacock genome (Fig. 8). There are around 60 different avian species that have been sequenced by using various sequencing technologies (Table S10). The sequencing depth varies from as low as 6x to maximum of 390x coverage. The result obtained from different bioinformatics methods to assemble the sequencing data are measured as scaffold N50 that is, from 30 Kb to 14 Mb.

Conclusions

A rapid surge in de novo genome sequence assembly of diverse species is seen in recent years [35]. This is essentially driven largely due to an affordable cost per base sequencing along with the development of smarter algorithms refined and equipped to handle large data sets [36-38]. The challenge of newer genome analysis pipeline lies in generating assembly with lower contig numbers and longer contigs per genome. To achieve this, technologies that generate longer reads or greater read depths are found to be very helpful [39]; but most importantly combination of different sequencing technologies play a significant role in improving genome assemblies [40] (Table S10). Libraries generated using different chemistry have been found to be superior on improving assemblies [41]. Further, a combination of different sequencing platform like Illumina when used in combination with other technologies like Sanger sequencing, Pacbio and ONT have shown to reduce the number of scaffolds even with very low coverage. Thus, we need to consider combination of sequencing technologies, along with using different bioinformatics software to obtain assembly with fewer number or scaffolds or closer to chromosome-level [42].

In comparison with other avian genomes [43], the 290X sequencing depth generated for peacock is one of the highest. The final draft genome assembly of peacock resulted in N50 of 0.23 MB. Inclusion of 2.3X of reads from Oxford Nanopore helped the assembly to improve by 26.2% reduction in the number of scaffolds and about 50.7% and 115% increase in the scaffold and contig N50, respectively. The draft assembly contained less than 0.4% of unknown nucleotides, which is very low for a draft assembly. Thus, we have shown for the first time in avian genomics how the low-cost third generation sequencing data from Oxford Nanopore can play a significant role in improving the genomes draft assembly. Assemblies with longer scaffolds will further benefit in understanding the organisms with structurally complex regions, repeat elements and isoforms in the genome [37].

The confidence on the predicted peacock proteins got strengthened when about 95% of them showed significant homology to various genomic features from different databases (Fig. 7). The phylogeny based on the conserved proteins across the avians showed that the peacock being closest with chicken followed by turkey and duck, the grouping correlated to the previous mitochondrial phylogeny [44]. Thus the genome sequence further gives insights on its genetic lineage and evolution with relation to the other avian members. The estimated median divergence time of *P. cristatus* from *G. gallus* is of about 35 million years ago (MYA), whereas between *G. gallus* and *M. gallopavo* is about 37 MYA [45]. The huge gap of other avians to peacock is due to non-availability of genome sequences from other avians. The gap can be by sequencing other avian species. Among the vertebrates, it has been observed that the variations in TEs among avians are very low [46] (Table S8). The genome complexities of a species are influenced by the transposable elements (TE) that are believed to play a crucial role [47]. In this peacock genome assembly, inclusion of Oxford Nanopore long read sequences has significantly improved the assembly, thus, helping in resolving

across the repetitive regions in genome. Their roles in development and evolution of the peacocks need to be further explored.

The genome information of peacock can be valued and explored by avian enthusiasts to further understand about the avian world. Though not yet critically endangered in India, peafowl population is surely at a declining trend in the wild due to massive deforestation, habitat loss [48] and increased poaching for meat and feathers. Our genome sequencing initiative of *Pavo cristatus* is not only valuable from a conservational viewpoint, but also to preserve a heritage associated with this bird that runs through centuries and that bears a strong attachment to the national psyche.

Availability of supporting data

Supplementary data contains, read statistics, annotation, repeats identification, orthology analysis, assembly and annotation. Additional figures included are the peacock, chicken and turkey proteins showing similarity to Pfam domains, top ten Gene ontology annotations in Biological process; Cellular component and Molecular function from the Peacock proteins, and Peacock homologous proteins in humans. Additional data are available from https://biit.cs.ut.ee/supplementary/peacock/

Raw Data and genome assembly in SRA

Raw reads (Illumina and Oxford Nanopore) are available in the Sequence Read Archive (SRA), and the Whole Genome Shotgun project has been deposited at GenBank under SRA Submission ID: SUB3108024, Bioproject: PRJNA413288 and Biosamples SUB3108018/SAMN07739105 : SKPea2016_SI, SUB3108017/SAMN07739104 : SKPea2016_LI, SUB3107930/SAMN07739101 : FPL_3_5KB,

SUB3108015/SAMN07739102 : FPL_5_7KB, SUB3108016/SAMN07739103 : FPL_7_10KB and SUB3108020/SAMN07739107 : FPL_Nano (Table 1). The de novo genome assembly can be accessed under SUB4504869/ SAMN07739105.

Competing interests

The author(s) declare that they have no competing interests.

Authors contributions

RD, AS, KP performed wet lab experiments; RD designed work plan, experiments and logistics; SS, VR, KP SG IM and AR assisted with the work; RS provided samples from bird; BR, SK performed data analysis and interpretation; BR, SK drafted the manuscript and SK overseen the whole project.

Acknowledgements

Department of Biochemistry, AIIMS, New Delhi for providing space and infrastructure to carry on the work. RD for providing partial funding. Genotypic Technology and their team for providing sequencing services. BR acknowledges IUT 34-4.

Tables

Table 1. Raw data statistics of Illumina HiSeq and Nanopore reads of the peacock genome.

Sample	Platform	Library and chemistry	Number of reads	Coverage	SRA ID
SO_6221_SKPea2016_SI	HiSeq	PE – SI (150 * 2)	489114747	146.73	SUB3108018, SAMN07739105
SO_6221_SKPea2016_LI	HiSeq	PE – LI (150 * 2)	302884819	90.87	SUB3108017, SAMN07739104
SO_6221_FPL_3_5KB	HiSeq	MP (150 * 2)	72915033	21.87	SUB3107930, SAMN07739101
SO_6221_FPL_5_7KB	HiSeq	MP (150 * 2)	47440144	14.23	SUB3108015, SAMN07739102
SO_6221_FPL_7_10KB	HiSeq	MP (150 * 2)	36464628	10.94	SUB3108016, SAMN07739103
SO_6221_NP	Nanopore	5 - 341124	366323	2.3	SUB3108020, SAMN07739107

Abbreviations used, PE = Paired end, SI = Short Insert, LI = Long insert, MP = Mate pair, NP = Nano pore and KB = Kilo Bases

Table 2. De novo assembly statistics of the peacock genome.

Description	Contigs	Nanopore Scaffold	Super Scaffolds	GapClosed	>1000 Kb	>5000 Kb
Contigs	685,241	281,272	179,346	179,332	34,178	15,025
Maximum Length	49,159	251,510	2,390,121	2,488,982	2,488,982	2,488,982
Minimum Length	300	5	265	265	1000	5000
Average Length	1360	3250	5111	5729	-	-
Total Length	932,162,464	914,363,908	916,720,956	1,027,510,962	954,449,349	915,342,012
Length >= 100 bp	685,241	281,271	179,346	179,332	34,178	15,025
Length >= 200 bp	685,241	281,271	179,346	179,332	34,178	15,025
Length >= 500 bp	616,120	186,433	93,727	93,718	34,178	15,025
Length >= 1 Kb	363,428	104,479	34,168	34,178	34,178	15,025
Length >= 10 Kb	1591	24,748	9249	10,310	10,310	10,310
Length >= 1 Mb	0	0	27	37	37	37
Non-ATGC #	350,325	42,696,911	49,169,831	4,043,129	4,040,790	3,986,487
Non-ATGC %	0.038	4.67	5.36	0.393	0.423	0.436
N50 value	1639	14,748	168,140	190,304	218,023	232,312

Figure legend

Figure 1. The beautiful and charismatic photo of Indian blue peacock (*Pavo cristatus)* bird.

Figure 2. Detailed workflow for de novo whole genome assembly and annotation.

Figure 3. Peacock proteins showing homology**.** Pie chart showing significant similarity scores of peacock proteins against the NR database.

Figure 4. Phylogenetic tree generated from homologous proteins from 49 different avian species.

Figure 5. Venn diagram showing common and not present protein family domains (Pfam) between peacock, chicken and turkey proteins.

Figure 6. Heatmap showing protein family (Pfam) distributed in peacock, chicken or turkey species. The number represents the Pfam domain count predicted from the protein sequences. Pfam domains of 50 and above identified in any one of the species are compared in the heatmap.

Figure 7. Venn diagram showing peacock proteins showing significant homology to NR database, KOG, Pfam and GO ontologies.

Figure 8. Circular image of the assembled peacock genome aligned against the *G. gallus* genome. The right side of the image represents the reference chicken genome and left side of the image represents the peacock genome.

References:

1. Brickle NW. Habitat use, predicted distribution and conservation of green peafowl (Pavo muticus) in Dak Lak Province, Vietnam. Biological Conservation. 2002;105:189–97.

2. Jackson CE. Peacock. London: Reaktion, 2006.

3. Kadgaonkar, Shivendra B. The peacock in ancient Indian art and literature. Bulletin of the Deccan College Research Institute. 1993;53:95–115.

4. Gadagkar R. Is the peacock merely beautiful or also honest? Current Science. 2003;85:1012–20.

5. Kushwaha S, Kumar A. A Review on Indian Peafowl (Pavo cristatus) Linnaeus, 1758. Journal of Wildlife Research. 2016;4:42–59.

6. Hillier LW, Miller W, Birney E et al. Sequence and comparative analysis of the chicken genome provide unique perspectives on vertebrate evolution. International Chicken Genome Sequencing Consortium (ed.). Nature. 2004;432:695–716.

7. Zhang G, Jarvis ED, Gilbert MTP. A flock of genomes. Science. 2014;346:1308–9.

8. Bejerano G, Pheasant M, Makunin I et al. Ultraconserved elements in the human genome. Science. 2004;304:1321–5.

9. Burt DW. Emergence of the Chicken as a Model Organism: Implications for Agriculture and Biology. Poultry Science. 2007;86:1460–71.

10. Furlong RF. Insights into vertebrate evolution from the chicken genome sequence. Genome Biology. 2005;6:207.

11. Andrews S. FastQC A Quality Control tool for High Throughput Sequence Data. Babraham.ac.uk 2010.

12. Kajitani R, Toshimoto K, Noguchi H et al. Efficient de novo assembly of highly heterozygous genomes from whole-genome shotgun short reads. Genome Research. 2014;24:1384–95.

13. Loman NJ, Quinlan AR. Poretools: a toolkit for analyzing nanopore sequence data. Bioinformatics. 2014;30:3399–401.

14. Li H, Durbin R. Fast and accurate short read alignment with Burrows-Wheeler transform. Bioinformatics. 2009;25:1754–60.

15. Birol I, Jackman SD, Nielsen CB et al. De novo transcriptome assembly with ABySS. Bioinformatics. 2009;25:2872–7.

16. Boetzer M, Pirovano W. SSPACE-LongRead: scaffolding bacterial draft genomes using long read sequence information. BMC Bioinformatics. 2014;15:211.

17. Marçais G, Kingsford C. A fast, lock-free approach for efficient parallel counting of occurrences of k-mers. Bioinformatics. 2011;27:764–70.

18. Smit A, Hubley R, Green P. RepeatMasker Open-4.0. 2013-2015. www.repeatmasker.org.

19. Stanke M, Diekhans M, Baertsch R et al. Using native and syntenically mapped cDNA alignments to improve de novo gene finding. Bioinformatics. 2008;24:637–44.

20. Boetzer M, Henkel CV, Jansen HJ et al. Scaffolding pre-assembled contigs using SSPACE. Bioinformatics. 2010;27:578–9.

21. Altschul SF, Gish W, Miller W et al. Basic local alignment search tool. Journal of molecular biology. 1990;215:403–10.

22. Moriya Y, Itoh M, Okuda S et al. KAAS: an automatic genome annotation and pathway reconstruction server. Nucleic Acids Research. 2007;35:W182–5.

23. Nordberg H, Cantor M, Dusheyko S et al. The genome portal of the Department of Energy Joint Genome Institute: 2014 updates. Nucleic Acids Research. 2013;42:D26–31.

24. El-Gebali S, Mistry J, Bateman A et al. The Pfam protein families database in 2019. Nucleic Acids Research. 2018;47:D427–32.

25. Zhang G, Li B, Li C et al. Comparative genomic data of the Avian Phylogenomics Project. GigaScience. 2014;3:26.

26. Jarvis ED, Mirarab S, Aberer AJ et al. Phylogenomic analyses data of the avian phylogenomics project. GigaScience. 2015;4:4.

27. Fu L, Niu B, Zhu Z et al. CD-HIT: accelerated for clustering the next-generation sequencing data. Bioinformatics. 2012;28:3150–2.

28. Larkin MA, Blackshields G, Brown NP et al. Clustal W and Clustal X version 2.0. Bioinformatics. 2007;23:2947–8.

29. Talavera G, Castresana J. Improvement of Phylogenies after Removing Divergent and Ambiguously Aligned Blocks from Protein Sequence Alignments. Systematic Biology. 2007;56:564–77.

30. Felsenstein J. PHYLIP (Phylogeny Inference Package) Version 3.57c. http://evolution.genetics.washington.edu/phylip.html 1993.

31. Nguyen L-T, Schmidt HA, von Haeseler A et al. IQ-TREE: A Fast and Effective Stochastic Algorithm for Estimating Maximum-Likelihood Phylogenies. Molecular Biology and Evolution. 2014;32:268–74.

32. Frith MC, Kawaguchi R. Split-alignment of genomes finds orthologies more accurately. Genome Biology. 2015;16:106.

33. Krzywinski M, Schein J, Birol I et al. Circos: an information aesthetic for comparative genomics. Genome research. 2009;19:1639–45.

34. Warren WC, Hillier LW, Tomlinson C et al. A New Chicken Genome Assembly Provides Insight into Avian Genome Structure. G3: Genes, Genomes, Genetics. 2016;7:109–17.

35. Peona V, Weissensteiner MH, Suh A. How complete are “complete” genome assemblies?-An avian perspective. Molecular Ecology Resources 2018;18:1188–95.

36. Muir P, Li S, Lou S et al. The real cost of sequencing: scaling computation to keep pace with data generation. Genome Biology. 2016;17:53.

37. Goodwin S, McPherson JD, McCombie WR. Coming of age: ten years of next-generation sequencing technologies. Nature Reviews Genetics. 2016;17:333–51.

38. Levy SE, Myers RM. Advancements in Next-Generation Sequencing. Annual Review of Genomics and Human Genetics. 2016;17:95–115.

39. Lu H, Giordano F, Ning Z. Oxford Nanopore MinION Sequencing and Genome Assembly. Genomics, Proteomics & Bioinformatics. 2016;14:265–79.

40. Rice ES, Green RE. New Approaches for Genome Assembly and Scaffolding. Annual Review of Animal Biosciences. 2018;7.

41. Weissensteiner MH, Pang AWC, Bunikis I et al. Combination of short-read, long-read, and optical mapping assemblies reveals large-scale tandem repeat arrays with population genetic implications. Genome Research. 2017;27:697–708.

42. Sohn J, Nam J-W. The present and future of de novowhole-genome assembly. Briefings in Bioinformatics. 2016:bbw096.

43. Zhang G, Li C, Li Q et al. Comparative genomics reveals insights into avian genome evolution and adaptation. Science. 2014;346:1311–20.

44. Dalloul RA, Long JA, Zimin AV et al. Multi-Platform Next-Generation Sequencing of the Domestic Turkey (Meleagris gallopavo): Genome Assembly and Analysis. Roberts RJ (ed.). PLoS Biology 2010;8:e1000475.

45. Kumar S, Stecher G, Suleski M et al. TimeTree: A Resource for Timelines, Timetrees, and Divergence Times. Molecular Biology and Evolution. 2017;34:1812–9.

46. Sotero-Caio CG, Platt RN, Suh A et al. Evolution and Diversity of Transposable Elements in Vertebrate Genomes. Genome Biology and Evolution. 2017;9:161–77.

47. Kapusta A, Suh A. Evolution of bird genomes-a transposon's-eye view. Annals of the New York Academy of Sciences. 2016;1389:164–85.

48. Ramesh K, McGowan P. On the current status of Indian Peafowl Pavo cristatus (Aves: Galliformes: Phasianidae): keeping the common species common. Journal of Threatened Taxa. 2009;1:106–8.

Figure 1 Click here to access/download;Figure;Fig 1. Peacock bird.jpg

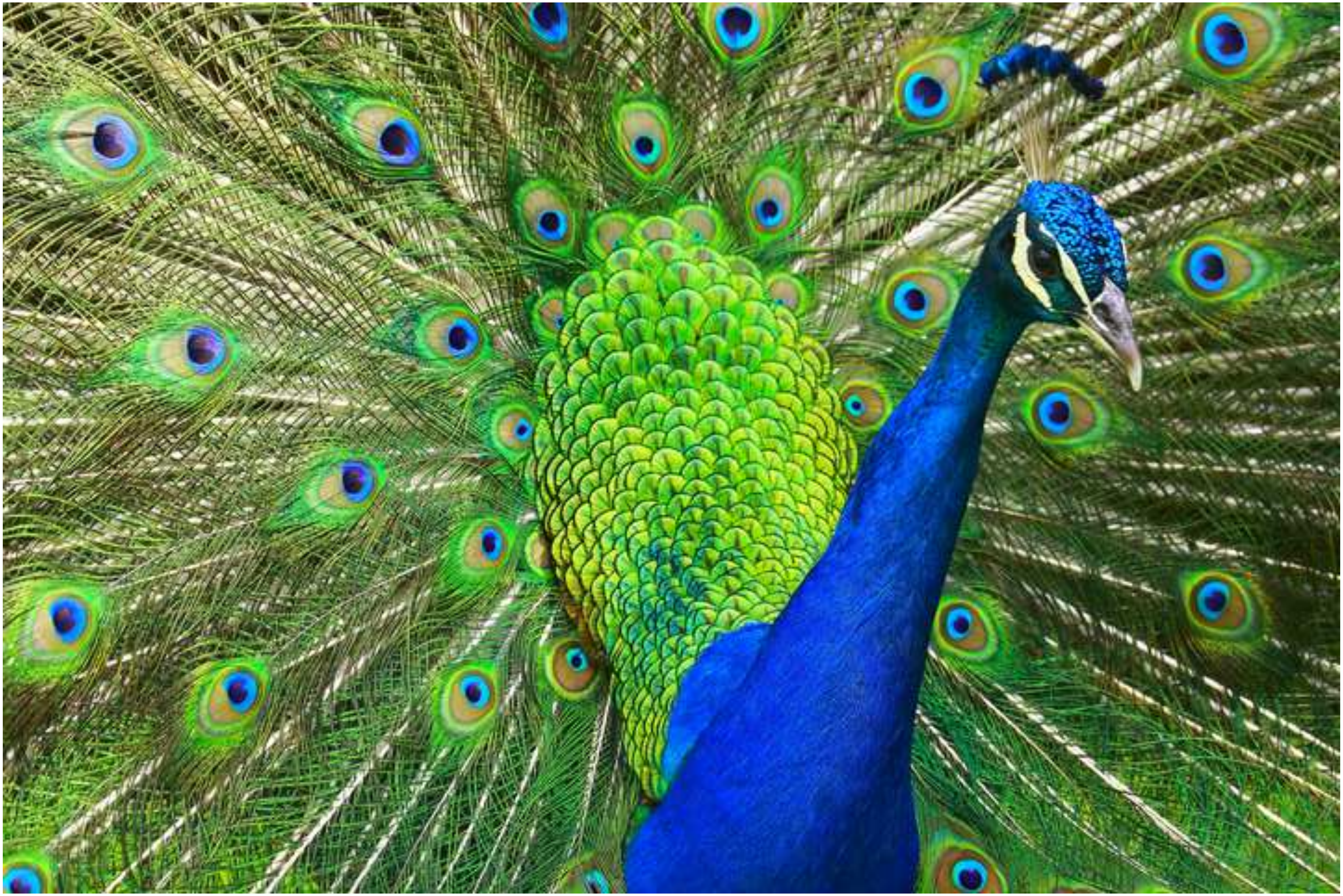

Figure 2 Click here to access/download;Figure;Fig 2. Workflow.jpeg

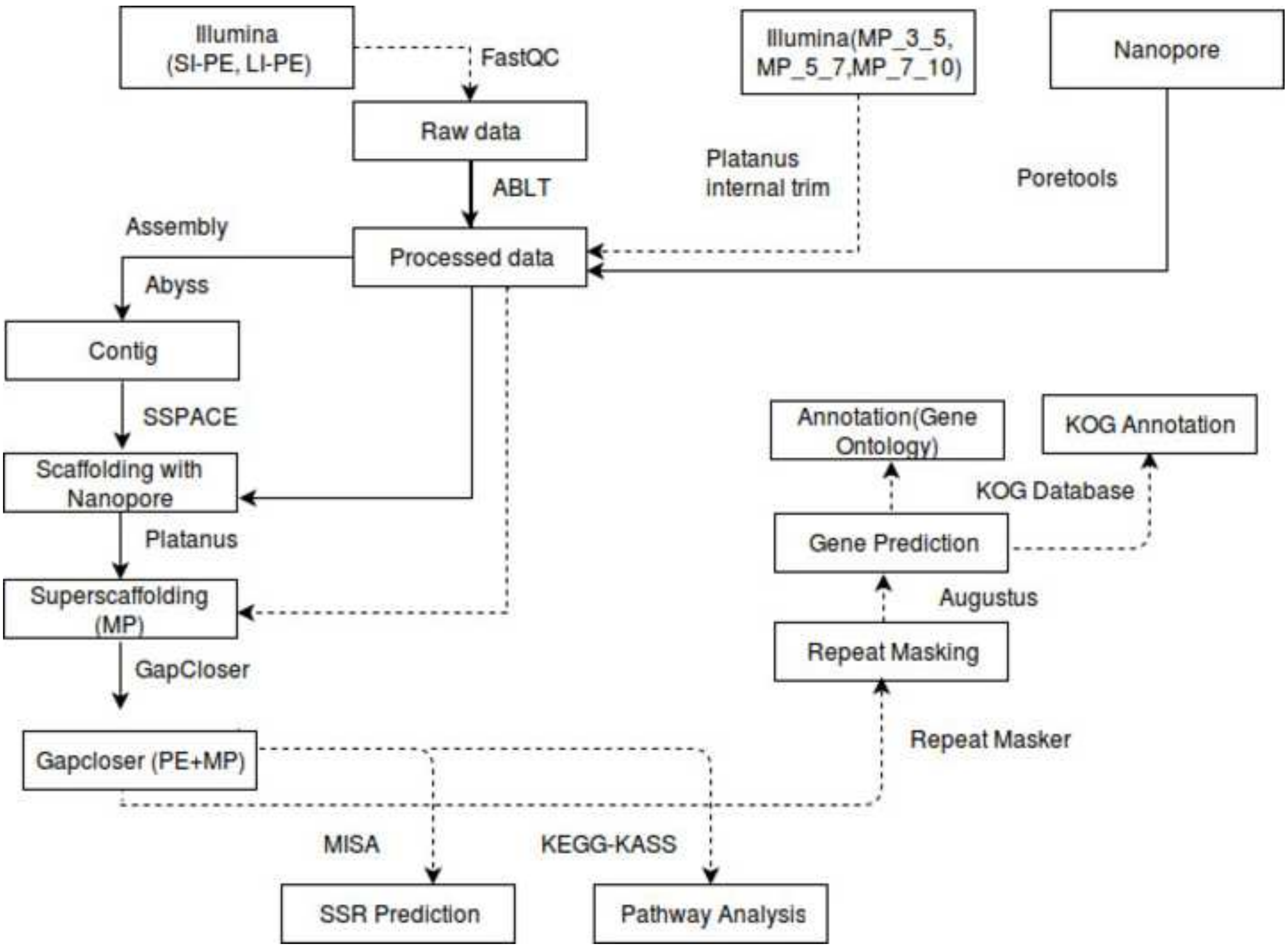

Figure 3

Click here to access/download;Figure;Fig 3 Similarity scores against the Uniprot database.pdf

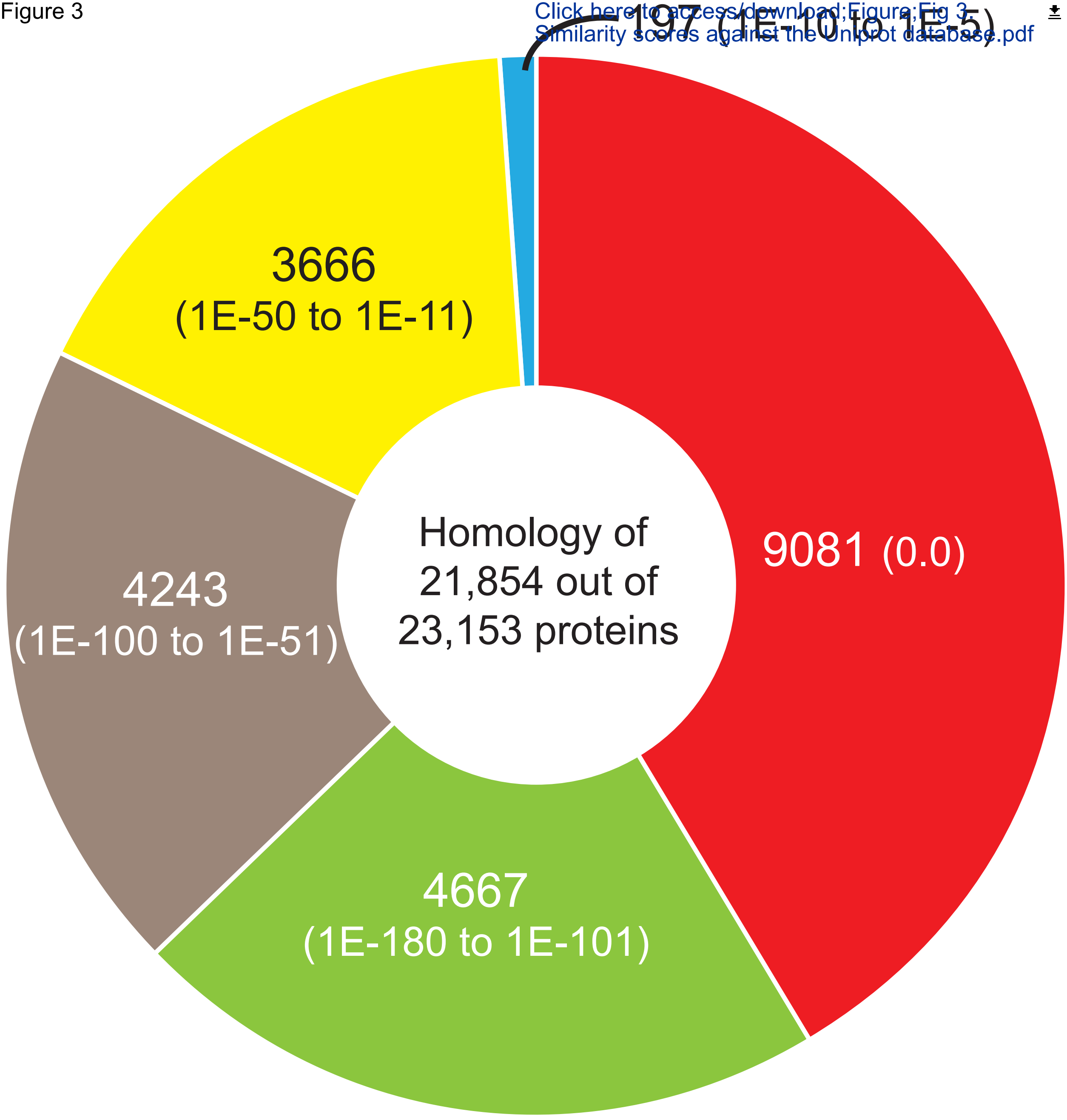

Figure 4

Click here to access/download;Figure;Fig 4. Phylogeny of avian species.pdf

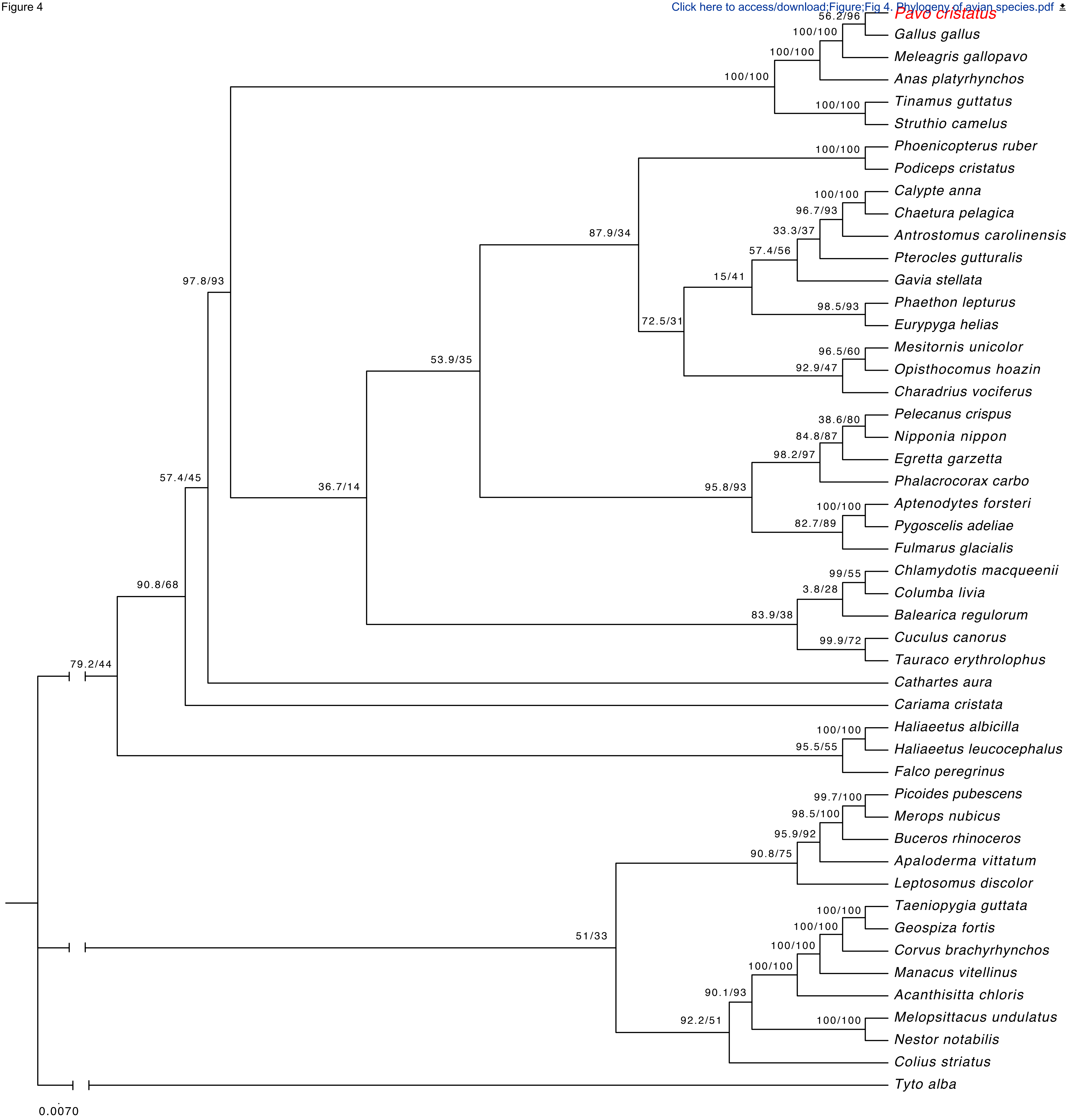

Figure 5

Click here to access/download;Figure;Fig 5. Unique Pfam domains common with chicken

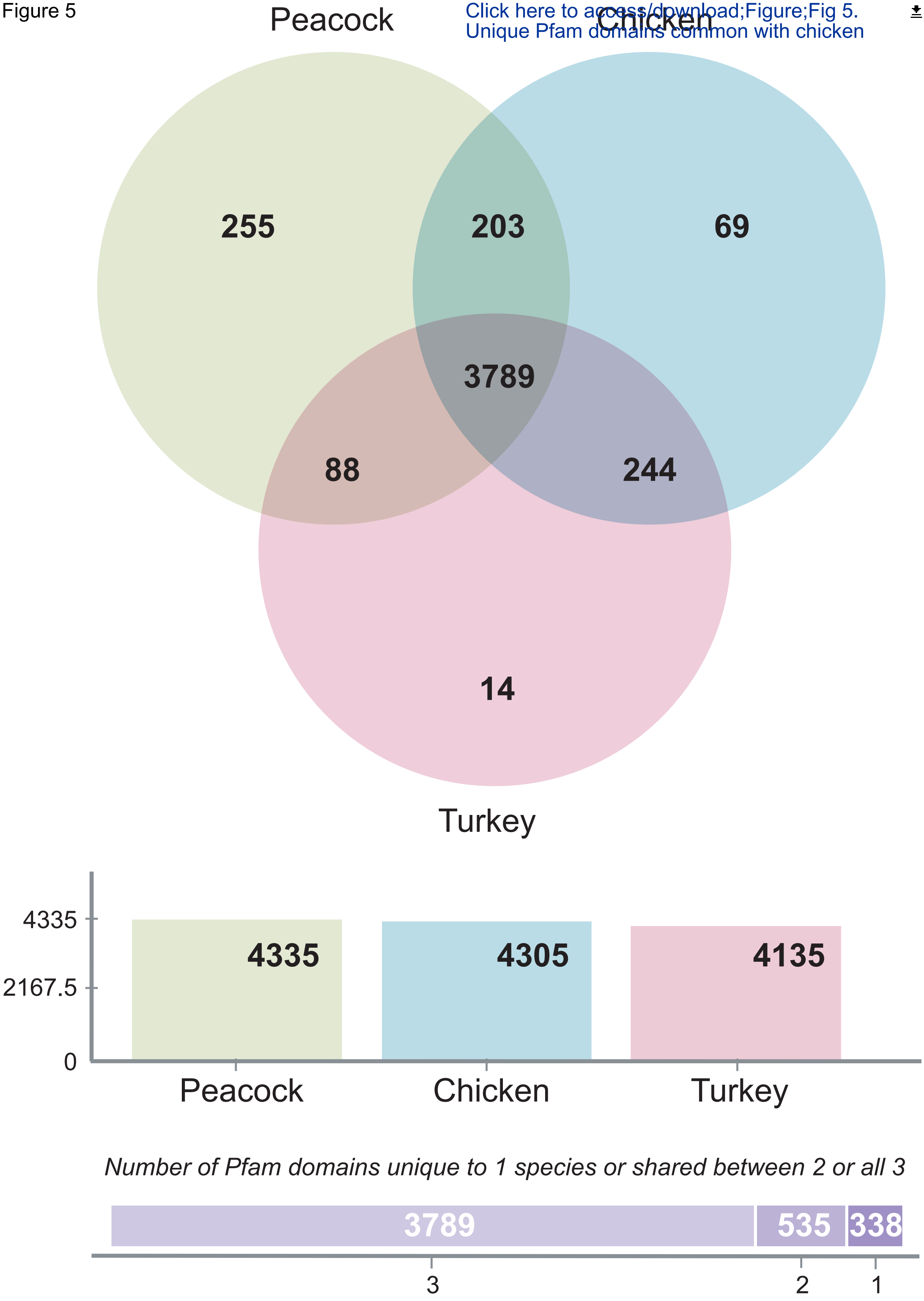

Click here to access/download;Figure;Fig 6. Heatmap

Figure 6

Peacock	Chicken	Turkey	
494	583	550	WD40
518	405	310	fn3
516	282	285	I–set
484	421	370	Cadherin
398	297	293	Pkinase
336	313	245	LRR_8
328	273	202	7tm_1
322	327	263	Collagen
198	309	128	zf–C2H2
288	296	277	Ank_2
267	241	218	RRM_1
38	245	60	7tm_4
233	237	158	EGF_CA
220	235	175	Sushi
201	224	176	EGF
215	186	161	Kelch_1
134	213	185	TSP_1
208	173	168	Spectrin
188	200	165	PDZ
154	196	156	Laminin_EGF
192	142	89	Homeobox
189	170	169	C2
179	113	110	Pkinase_Tyr
174	145	131	Ion_trans
165	161	110	Ldl_recept_a
150	164	149	PH
151	163	132	Ig_3
162	113	94	BTB
147	107	110	Ras

Peacock	Chicken	Turkey	
118	137	97	V–set
131	128	87	CUB
123	130	114	LIM
126	102	103	Mito_carr
98	121	85	SRCR
109	89	83	SH2
106	69	48	HLH
104	92	80	SH3_1
103	101	101	LRR_6
101	99	97	Arm
25	101	21	Ig_2
4	1	101	RVT_1
100	88	84	Ldl_recept_b
96	98	58	zf–C2H2_6
97	27	12	Plectin
89	92	90	IQ
66	92	89	RCC1
89	91	69	zf–met
90	49	52	Myosin_head
89	83	78	Helicase_C
76	89	34	Nebulin
87	50	43	p450
68	86	72	Lectin_C
83	36	40	Filament
82	61	49	Trypsin
80	65	59	VWA
74	80	71	EF–hand_7
28	78	15	Keratin
77	73	73	CH

Peacock	Chicken	Turkey	
47	77	72	TPR_8
75	56	57	RhoGAP
65	74	65	SH3_9
72	46	34	VWD
57	72	63	MORN
71	48	50	Y_phosphatase
68	71	69	KH_1
70	52	46	BACK
69	68	59	Laminin_G_2
69	59	22	Cadherin_3
68	65	61	zf–C2H2_4
65	67	66	ABC_tran
63	50	56	RhoGEF
58	62	57	FXa_inhibition
61	43	42	UCH
59	53	38	SAM_1
59	29	29	AMP–binding
56	43	39	Kinesin
56	29	30	ANF_receptor
38	56	49	WW
55	51	42	DEAD
54	52	48	AAA
54	40	32	Hormone_recep
53	45	46	adh_short
52	53	44	MFS_1
51	26	27	Sema
50	43	34	BTB_2
50	26	16	Forkhead
37	50	12	Calx–beta

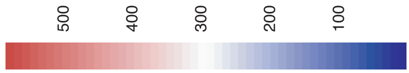

Figure 7

Click here to access/download;Figure;Fig 7. Venn of annotations.pdf

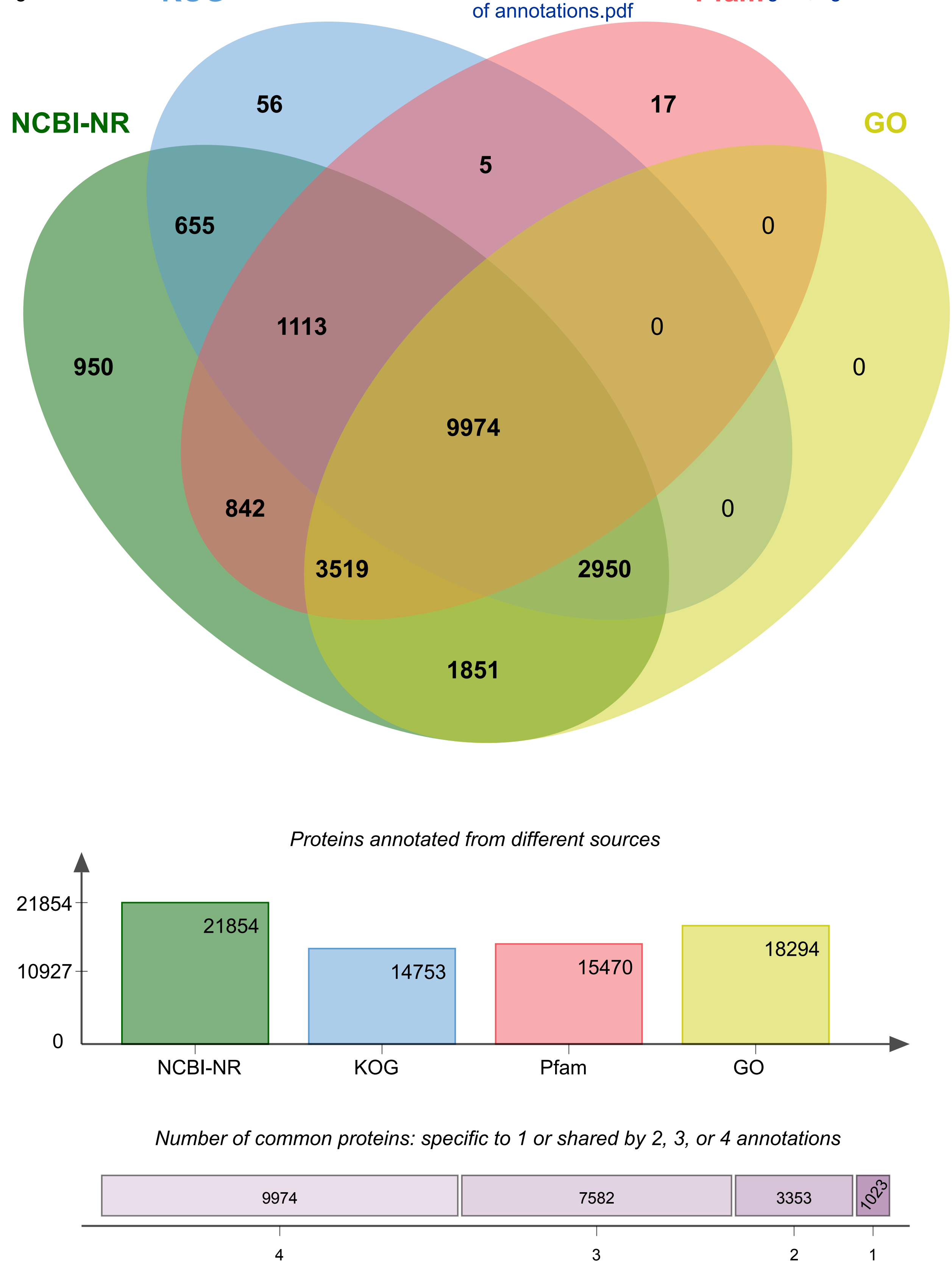

Figure 8

Click here to access/download;Figure;Fig 8. Peacock scaffolds against Gallus circular synteny.png

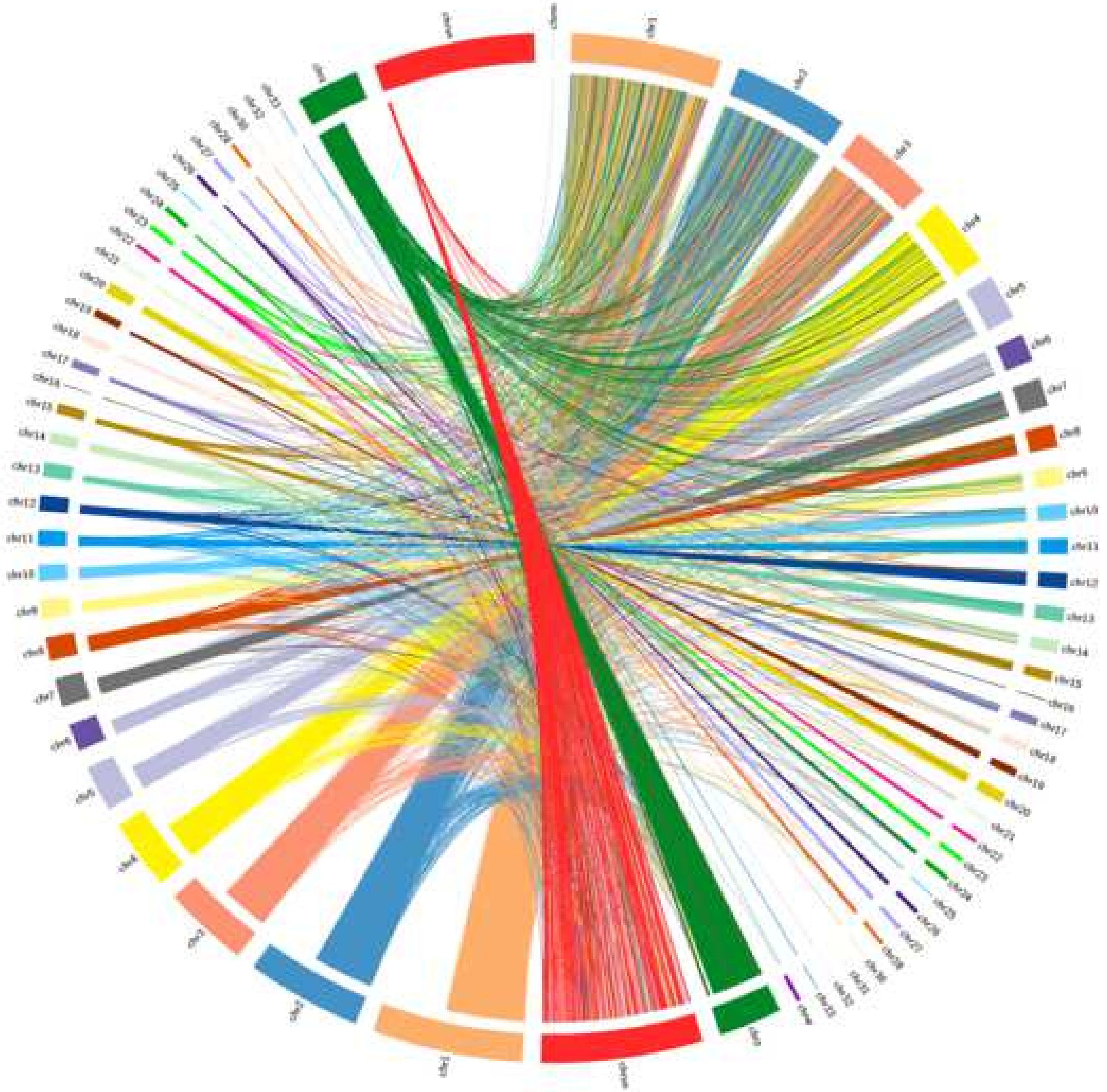

Click here to access/download
Supplementary Material
Supplementary_Description of all the tables and figures.docx

Click here to access/download
Supplementary Material
Table_S1_ReadStats_Table_S2_TEs.xlsx

Supplementary Material: Table S3

Click here to access/download
Supplementary Material
Table_S3_Repeats.xlsx

Click here to access/download
Supplementary Material
Table_S4_Gene_annotations_of_peacock_proteins.xlsx

Click here to access/download
Supplementary Material
Table_S5_KEGG_annotation.xlsx

Supplementary Material: Table S6

Click here to access/download
Supplementary Material
Table_S6_KOG_annotation.xlsx

Click here to access/download
Supplementary Material
Table_S7_BlastVsHumanProteins.xlsx

Supplementary Material: Table S8

Click here to access/download
Supplementary Material
Table_S8_Orthologous_proteins

Supplementary Material: Table S9

Click here to access/download
Supplementary Material
Table_S9_Pfam_Analysis.xlsx

Supplementary Material: Table S10

Click here to access/download
Supplementary Material
Table_S10_Bird_Species_with_counts.xlsx

Supplementary Material: Figure S1

Click here to access/download
Supplementary Material
Fig S1. Proteins showing similarity to Pfam domains.pdf

Supplementary Material: Figure S2

Click here to access/download

Supplementary Material

Fig S2. Gene Ontololgy of top 10 WGS.png

Supplementary Material: Figure S3

Click here to access/download
Supplementary Material
Fig S3.Peacock vs Human_GO.pdf
