# Supplementary material for: De novo assembly of the Indian blue peacock (Pavo cristatus) genome using Oxford Nanopore technology and Illumina sequencing
Source: Gigascience. 2019 May 11;8(5):giz038. doi: 10.1093/gigascience/giz038 (PMC6511069; doi:10.1093/gigascience/giz038)
Supplement: GIGA-D-18-00280_Revision_4.pdf [file giz038_giga-d-18-00280_revision_4.pdf]

GigaScience

De novo assembly of the Indian Blue Peacock (Pavo cristatus) genome using Oxford Nanopore Technology and Illumina sequencing

--Manuscript Draft--

**Manuscript Number:**	GIGA-D-18-00280R4
**Full Title:**	De novo assembly of the Indian Blue Peacock (Pavo cristatus) genome using Oxford Nanopore Technology and Illumina sequencing
**Article Type:**	Data Note
**Funding Information:**	
**Abstract:**	Background: The Indian peafowl (Pavo cristanus) is native to South Asia and is the national bird of India. Here we present a draft genome sequence of the male blue peacock using Illumina and Oxford Nanopore Technology (ONT).<br>Results: ONT sequencing gave approximately 2.3-fold sequencing coverage, whereas Illumina generated 150-bp paired-end sequence data at 284.6-fold coverage from five libraries. Subsequently, we generated a 0.915-Gb de novo assembly of the peacock genome with a scaffold N50 of 0.23 Mb. We predict that the peacock genome contains 23,153 protein-coding genes and 75.3 Mb (7.33%) of repetitive sequences.<br>Conclusions: We report a high-quality assembly of the peacock genome using a hybrid approach of sequences generated by both Illumina and ONT. The long-read chemistry generated by ONT was useful for addressing challenges related to de novo assembly, particularly at regions containing repetitive sequences spanning longer than the read length, and which could not be resolved with only short-read-based assembly. Contig assembly of Illumina short reads gave an N50 of 1,639 bases, whereas with ONT, the N50 increased by more than nine-fold to 14,749 bases. The initial contig assembly based on Illumina sequencing reads alone gave 685,241 contigs. Further scaffolding on assembled contigs using both Illumina and ONT sequencing reads resulted in a final assembly of 15,025 super-scaffolds, with an N50 of about 0.23 Mb. Ninety-five per cent of proteins predicted by homology matched with those in a public repository, verifying the completeness of our assembly. Like other phylogenetic studies of avian conserved genes, we found P. cristatus to be most closely related to Gallus gallus, followed by Meleagris gallopavo and Anas platyrhynchos. Compared with the recently published peacock genome assembly, the current, superior, hybrid assembly has greater sequencing depth, fewer non-ATGC sequences, and fewer scaffolds.
**Corresponding Author:**	Subhradip Karmakar, PhD<br>All India Institute of Medical Sciences<br>New Delhi, Delhi INDIA
**Corresponding Author Secondary Information:**	
**Corresponding Author's Institution:**	All India Institute of Medical Sciences
**Corresponding Author's Secondary Institution:**	
**First Author:**	Ruby Dhar
**First Author Secondary Information:**	
**Order of Authors:**	Ruby Dhar
	Ashikh Seethy
	Karthikeyan Pethusamy
	Vishwajeet Rohil
	Sunil Singh
	Kakali Purkayastha
	Indrani Mukherjee

Powered by Editorial Manager® and ProduXion Manager® from Aries Systems Corporation

	Sandeep Goswami
	Rakesh Singh
	Ankita Raj
	Tryambak Srivastava
	Sovon Acharya
	Balaji Rajashekhar, Ph.D
	Subhradip Karmakar, PhD
**Order of Authors Secondary Information:**	
**Response to Reviewers:**	Dear Dr. Scott,<br>Kindly find our response to the minor corrections in the manuscript.<br>All the comments have been replied and corrected in the manuscript, figure 2 and supplementary table and figure description.<br><br>Thank you for all the help and review provided.<br>Regards,
**Additional Information:**	
**Question**	**Response**
Are you submitting this manuscript to a special series or article collection?	No
**Experimental design and statistics**<br><br>Full details of the experimental design and statistical methods used should be given in the Methods section, as detailed in our Minimum Standards Reporting Checklist. Information essential to interpreting the data presented should be made available in the figure legends.<br><br>Have you included all the information requested in your manuscript?	Yes
**Resources**<br><br>A description of all resources used, including antibodies, cell lines, animals and software tools, with enough information to allow them to be uniquely identified, should be included in the Methods section. Authors are strongly encouraged to cite Research Resource Identifiers (RRIDs) for antibodies, model organisms and tools, where possible.<br><br>Have you included the information	Yes

requested as detailed in our Minimum Standards Reporting Checklist?	
**Availability of data and materials**<br><br>All datasets and code on which the conclusions of the paper rely must be either included in your submission or deposited in publicly available repositories (where available and ethically appropriate), referencing such data using a unique identifier in the references and in the “Availability of Data and Materials” section of your manuscript.<br><br>Have you have met the above requirement as detailed in our Minimum Standards Reporting Checklist?	Yes

Powered by Editorial Manager® and ProduXion Manager® from Aries Systems Corporation

Manuscript
Click here to access/download;Manuscript;Manuscript_revision_13032019_f
Click here to view linked References

De novo assembly of the Indian Blue Peacock (*Pavo cristatus*) genome using Oxford Nanopore Technology and Illumina sequencing

Ruby Dhar[1], Ashikh Seethy[1], Karthikeyan Pethusamy[1], Sunil Singh[1], Vishwajeet Rohil[2], Kakali Purkayastha[2], Indrani Mukherjee[1], Sandeep Goswami[1], Rakesh Singh[3], Ankita Raj[1], Tryambak Srivastava[1], Sovon Acharya[1], Balaji Rajashekhar[4,5*], Subhradip Karmakar[1*]

[1]Department of Biochemistry, Room No 3020, AIIMS, New Delhi, India

[2]Vallabhbhai Patel Chest Institute (VPCI), Delhi University, New Delhi, India

[3]Kanpur Zoo, Hastings Ave, Azad Nagar, Nawabganj, Kanpur, Uttar Pradesh 208002, India India

[4]Institute of Computer Science, University of Tartu, 50409 Tartu, Estonia

[5]Celixa, Bangalore, 560020, India

*Correspondence address: Balaji Rajashekar, Institute of Computer Science, University of Tartu, 50409 Tartu, Estonia; Tel: +91-9844677993; Email: balaji@ut.ee

Subhradip Karmakar, Department of Biochemistry, Room No 3020, AIIMS, New Delhi, India; Tel: +91-9999612564; Email: subhradip.k@aiims.edu

ORCID IDs: Ruby Dhar: 0000-0003-3600-6554; Ashikh Seethy: 0000-0001-6825-5753; Tryambak Pratap Srivastava: 0000-0002-7903-5876; Balaji Rajashekar: 0000-0002-1665-5584; Subhradip Karmakar: 0000-0002-4757-8729

Abstract

Background: The Indian peafowl (*Pavo cristanus*) is native to South Asia and is the national bird of India. Here we present a draft genome sequence of the male blue peacock using Illumina and Oxford Nanopore Technology (ONT).

Results: ONT sequencing gave approximately 2.3-fold sequencing coverage, whereas Illumina generated 150-bp paired-end sequence data at 284.6-fold coverage from five libraries. Subsequently, we generated a 0.915-Gb de novo assembly of the peacock genome with a scaffold N50 of 0.23 Mb. We predict that the peacock genome contains 23,153 protein-coding genes and 75.3 Mb (7.33%) of repetitive sequences.

Conclusions: We report a high-quality assembly of the peacock genome using a hybrid approach of sequences generated by both Illumina and ONT. The long-read chemistry generated by ONT was useful for addressing challenges related to de novo assembly, particularly at regions containing repetitive sequences spanning longer than the read length, and which could not be resolved with only short-read-based assembly. Contig assembly of Illumina short reads gave an N50 of 1,639 bases, whereas with ONT, the N50 increased by more than nine-fold to 14,749 bases. The initial contig assembly based on Illumina sequencing reads alone gave 685,241 contigs. Further scaffolding on assembled contigs using both Illumina and ONT sequencing reads resulted in a final assembly of 15,025 super-scaffolds, with an N50 of about 0.23 Mb. Ninety-five per cent of proteins predicted by homology matched with those in a public repository, verifying the completeness of our assembly. Like other phylogenetic studies of avian conserved genes, we found *P. cristatus* to be most closely related to *Gallus gallus*, followed by *Meleagris gallopavo* and *Anas platyrhynchos*. Compared with the recently published peacock genome assembly, the current, superior, hybrid assembly has greater sequencing depth, fewer non-ATGC sequences, and fewer scaffolds.

Keywords: Peacock, *Pavo cristatus*, Indian National Bird, Genome Assembly, Oxford Nanopore.

Data description

Background

Pavo cristatus, commonly known as the Indian Blue Peafowl, is native to South Asian countries. Apart from the wild, they are usually found as park and zoo exhibits, or are raised for breeding and conservation purposes [1, 2] (Fig. 1). Peafowl have been widely referred to in ancient Indian literature [3] and are closely associated with the life and culture of Southeast Asian, symbolizing beauty, love, grace and pride [4, 5]. For these reasons, the peafowl – specifically the peacock – was chosen to be the national bird of India in 1963.

Genome sequencing of the avian model organism *Gallus gallus* (the red junglefowl, or chicken) [6] and other avian species [7] has provided novel perspectives on vertebrate genome evolution, such as a better understanding of genome structure and annotating the mammalian genome. Genome studies of *G. gallus* have revealed high conservation within orthologous regions of the human genome [8], thus showing promise as a good candidate for studies on developmental biology, immunology and vertebrate genome architecture [9, 10].

Despite a wealth of information from existing avian genome sequencing projects, it remains important to sequence the genomes of other species to add value to avian and vertebrate genomics. Here, we use Oxford Nanopore Technology (ONT) to sequence a bird genome for the first time. The long reads generated from this sequencing technology were helpful during the de novo assembly of this genome, especially in the GC-rich repeat regions, which invariably pose serious challenges. By comparing this genome with those of other birds, we will understand more about the uniqueness of the peacock genome; the development of this species, its complex plumage pigmentation and sexual dimorphism; and its evolutionary

relationships with other birds. Characterization of genes and their specific functions will facilitate better understanding of the peafowl species. By comparing proteins between the peacock, chicken and *Meleagris gallopavo* (domestic turkey), conserved domains and functional annotations may be revealed.

Methods

Sample collection and extraction of DNA

Blood was collected from an Indian male peacock (Figure 1) at Kanpur Zoo, India, after obtaining the necessary ethical and institutional approvals.

DNA from blood was prepared for sequencing as follows: firstly, 200 µl of blood was added to a 1.5-ml microcentrifuge tube containing approximately 20 µl of proteinase K (PK) solution, and briefly mixed. Cell lysis buffer (200 µl) was added to the tube, which was mixed by vortexing for 10 seconds, then incubated at 56°C for 10 minutes. Then, 250 µl of binding buffer (BBA) was added to the tube, which was mixed by vortexing again for 10 seconds. The contents of the tube were added to a ReliaPrep™ (Promega, Madison, USA) binding column, which had been placed into an empty collection tube, then capped and placed in a refrigerated microcentrifuge. The binding column and tube were then centrifuged for 1 minute at 12000 rpm and flow-through was discarded. The binding column was placed into a fresh collection tube, 500 µl of column wash solution was added, and then centrifuged for 3 minutes at maximum speed, again discarding flow-through. Column washing was repeated three times. Columns were then placed in a clean, nuclease-free 1.5-ml microcentrifuge tube. Nuclease-free water (100 µl) was then added to the column and centrifuged for 1 minute more at maximum speed before discarding the column and saving the elute.

The concentration and purity of the extracted DNA was evaluated using a Nanodrop 2000 spectrophotometer (Thermo Fisher Scientific, MA, USA) and a Qubit fluorometer (Thermo Fisher Scientific, MA, USA), and integrity was checked on 0.8% agarose gel. The DNA sample was aliquoted for library preparation on two different platforms: Illumina HiSeq 2000 (Illumina, CA, USA) and Oxford Nanopore Technology (ONT) (Oxford, UK) MinION sequencing platform. The genome sequencing was performed by Genotypic Technology, Bengaluru, India in accordance to standard protocols.

Library preparation and sequencing

Paired-end library preparation and sequencing

Whole genome sequencing (WGS) libraries were prepared with an Illumina-compatible NEXTflex DNA sequencing kit (BIOO Scientific, Austin, TX, USA). Approximately 1 μg of genomic DNA was sheared using a Covaris S2 sonicator (Covaris, Woburn, MA, USA) to generate fragment sizes of approximately 300–600 bp. The fragment size distribution was checked using an Agilent 2200 TapeStation system with D1000 DNA screen tapes and reagents (Agilent Technologies, Palo Alto, CA, USA), and subsequently purified using HighPrep magnetic beads (Magbio Genomics Inc, USA). The purified fragments were end-repaired, adenylated and ligated to Illumina multiplex barcode adaptors, as per the NEXTflex DNA sequencing kit protocol (BIOO Scientific, Austin, TX, USA).

The adapter-ligated DNA was purified with HighPrep beads (MagBio Genomics, Inc, Gaithersburg, MD, USA), then size selected on 2% low melting agarose gel, and cleaned using a MinElute column (QIAGEN). The resulting fragments were amplified for 10 cycles of polymerase chain reaction (PCR) using the Illumina-compatible primers provided in the NEXTFlex DNA sequencing kit. The final PCR product (sequencing library) was purified with HighPrep beads, followed by a library quality control check. The Illumina-compatible

sequencing library was initially quantified using a Qubit fluorometer (Thermo Fisher Scientific, MA, USA), and fragment size distribution was analyzed on an Agilent TapeStation. Finally, the sequencing library was quantified by quantitative PCR (qPCR) using the Kapa Library Quantification Kit (Kapa Biosystems, Wilmington, MA, USA). The qPCR-quantified library was sequenced on an Illumina sequencer for 150-bp paired-end chemistry.

For each sample, the Illumina-compatible sequencing library had a fragment size range of 275–425 bp for paired-end short inserts (PE-SI), and 350–650 bp for paired-end long inserts (PE-LI). As the combined adapter size was approximately 120 bp, the effective user-defined insert size was 155–305 bp and 230–530 bp for PE-SI and PE-LI, respectively. Libraries were sequenced using the Illumina HiSeq platform [11] with 150 PE chemistry.

Mate-pair library preparation and sequencing

The mate-pair sequencing library was prepared using the Illumina-compatible NextEra Mate Pair Sample Preparation Kit (Illumina Inc., Austin, TX, USA). Approximately 4 µg of genomic DNA was simultaneously fragmented and tagged with mate-pair adapters in a transposon-based tagmentation step. Tagmented DNA was then purified using AMPure XP magnetic beads (Beckman Coulter Life Sciences, Indianapolis, IN, USA), followed by strand displacement to fill gaps in the tagmented DNA. Strand-displaced DNA was further purified with AMPure XP beads before size-selecting fragments of 3–5 Kb, 5–7 Kb and 7–10 Kb on low melting agarose gel. The fragments were circularized in an overnight blunt-end intra-molecular ligation step, which resulted in circularization of DNA with the insert mate-pair adapter junction. Circularized DNA was sheared using a Covaris S220 sonicator (Covaris, Woburn, MA, USA) to generate approximate fragment sizes of 300–1000 bp. The sheared DNA was purified to collect the mate-pair junction-positive fragments using Dynabeads M-

280 streptavidin magnetic beads (Thermo Fisher Scientific, Waltham, MA, USA). The purified fragments were end-repaired, adenylated and ligated to Illumina multiplex barcode adaptors, as per the NextEra Mate Pair Sample Preparation Kit protocol.

The adapter-ligated DNA was then amplified for 15 cycles of PCR using Illumina-compatible primers. The final PCR product (sequencing library) was purified with AMPure XP beads, followed by a library quality control check. The Illumina compatible sequencing library was initially quantified using a Qubit fluorometer (Thermo Fisher Scientific, MA, USA), and its fragment size distribution was analyzed with an Agilent TapeStation. Finally, the sequencing library was accurately quantified by qPCR using the Kapa Library Quantification Kit (Kapa Biosystems, Wilmington, MA, USA). The qPCR-quantified libraries were pooled in equimolar amounts to create a final multiplexed library pool for sequencing on an Illumina sequencer.

ONT MinION library preparation and sequencing

Genomic DNA (1.5 μg) was end-repaired using the NEBnext Ultra II End Repair kit (New England Biolabs, MA, USA), and cleaned up with 1x AmPure beads (Beckmann Coulter, USA). Adapter ligations were performed for 20 minutes using NEB blunt/TA ligase (New England Biolabs, MA, USA). The library mixtures were cleaned up using 0.4X AmPure beads (Beckmann Coulter, USA), and eluted in 25 μl of elution buffer. The eluted library was used for sequencing. Whole genome libraries were prepared using the ligation sequencing SQK-LSK108 Oxford Nanopore sequencing kit (ONT, Oxford, UK). Sequencing was performed on a MinION Mk1b (ONT, Oxford, UK) using SpotON flow cell (FLO-MIN106) in a 48-hour sequencing protocol on MinKNOW (version 1.1.20, ONT, Oxford, UK).

Raw data quality control and processing

Illumina raw data: quality control and processing

Illumina reads were de-multiplexed using bcl2fastq (Illumina). Raw genomic library data generated by Illumina was quality-checked using FastQC (FastQC, RRID:SCR_014583) [12]. Paired-end Illumina reads were processed for clipping adapter and low quality bases using a customized script that retains a minimum of 70% bases/reads with Phred score (Q≥30 in each base position) with a read length of 50 bp. Mate-pair libraries were trimmed for adapter sequences and low-quality bases, trimming from the 3-end using the PLATANUS internal trimmer (Platanus version 1.2.4, RRID:SCR_015531)[13].

ONT reads: base calling and processing

Raw data were base-called with the cloud-based Metrichor workflow 2D Basecalling plus Barcoding (Metrichor version 2.43.1, ONT, Oxford, UK [14]. ONT reads were processed using Poretools [15] to convert fast5 files to fasta format. The 2D reads or 1D high quality reads were selected for further assembly.

De novo genome assembly and genome size estimation

Quality-checked ONT reads were error-corrected using Illumina PE reads. For error-correction, the Illumina PE reads were aligned to the ONT reads using BWA aligner (BWA , RRID:SCR_010910) [16]. Paired-end reads were assembled using Abyss (ABySS, RRID:SCR_010709) [17], followed by contig extension using ONT reads using SSPACE-LongRead [18]. Super-scaffolding of the assembled scaffold was performed using SSPACE (SSPACE, RRID:SCR_005056) [19] and PLATANUS on the ONT and mate-pair data. A final draft genome resulted after gap closure using GAPCLOSER (GapCloser, RRID:SCR_015026) [20] and the PLATANUS gap_close tool, with Illumina data. The

genome size was estimated with a k-mer distribution plot using JELLYFISH (Jellyfish, RRID:SCR_005491) [21]. The assembly and annotation workflow is shown in Figure 2.

Identification of repetitive elements and simple sequence repeat (SSR) markers

Repetitive elements, retrotransposons and DNA transposons were identified in the draft genome, and hard-masked by using reference genomic repeats of *G. gallus* using Repeatmasker (RRID:SCR_012954) [22]. Final assembled scaffolds were analyzed to identify simple sequence repeats (SSRs). SSRs, such as di-, tri-, tetra-, penta- and hexa-nucleotide repeats in the genome, were identified using MISA (version 1.0.0) [23].

Annotation of the draft genome

Gene models were predicted on a hard-masked draft genome using AUGUSTUS (RRID:SCR_008417) [24], with *G. gallus* as a reference model. Predicted proteins were annotated using BLASTP (RRID:SCR_001010) [25] against the National Centerfor Biotechnology Information (NCBI)'s NR (non-redundant) database, with default parameters at an E-value cutoff of 1E-5.

Predicted proteins were searched against the Kyoto Encyclopedia of Genes and Genomes' Automatic Annotation Server (KEGG-KAAS) for pathway analysis [26]. *G. gallus, M. gallopavo*, *Taeniopygia guttata* (zebra finch), and *Falco peregrinus* (peregrine falcon) were used as reference organisms for pathway identification. EuKaryotic Orthologous Groups (KOGs) [27] were predicted using a homology-based approach.

Prediction of protein domains

Predicted proteins from peacock, chicken and turkey, with sequence lengths greater than 100 amino acids, were considered for protein domain analysis. All protein-coding sequences from each organism were searched against the Pfam-A database using Pfam scan [28].

Identification of avian protein families

A total of 748,544 protein sequences from 49 avian species (including peacock proteins from this study) and others were downloaded from the Avian Phylogenomics Project [29, 30]. Sequences with lengths greater than 100 amino acids from all the avian genomes were selected and concatenated to a single fasta file. These sequences were clustered using CD-HIT [31], with 70% alignment coverage for the shorter sequences, with a length difference cutoff of 0.7. Single-copy gene family orthologs present across all avian species, and not clustered peacock proteins, were annotated.

Phylogenetic tree construction

To construct a phylogenetic tree, we considered single-copy gene clusters present as single copies in all the avian species analyzed. These protein sequences from each species were concatenated and further aligned using the multiple sequence alignment tool Clustalw [32]. Poorly aligned positions and divergent regions were removed using Gblock [33]. Sequences in fasta format were converted to phylip format using Phylip [34]. Phylogenetic trees were constructed using IQ-TREE (version 1.5.6) [35]. The parameters used to construct the phylogenetic tree were ultrafast bootstrap (UFBoot, using the –bb option of 1000 replicates), and a standard substitution model (–st AA –m TEST), and alrt 1000 –nt AUTO was given to generate the tree. Trees generated from IQ-TREE were visualized using FigTree [36], and the branch-support values were recorded from the output '.treefile'. For better visualization, trees were modified under the 'Trees' section, and increasing order nodes were applied.

Genome conservation analysis

Draft chromosome visualizations were constructed by aligning the assembled peacock genome against that for *G. gallus* using the Chromosomer tool [37]. The reordered, assembled genome was aligned to the chicken genome using LAST aligner [38], with NEAR (finding short-and-strong [near-identical] similarities) parameters to allow for substitution and gap frequencies, leading to the identification of orthologs. For visualization, these query-mapped regions were filtered for >1% of the maximum length using Circos [39].

Results

Genome sequencing assessment

Five libraries were generated from 150-bp paired-end Illumina sequences. Short-insert reads (489,114,747) represented genome coverage of 146.7x, and 302,884,819 long-insert reads represented about 90.9x coverage, with a total coverage of 237.6x. Sequencing of three mate-pairs of 3–5 Kb, 5–7 Kb and 7–10 Kb yielded 72,915,033, 47,440,144 and 36,464,628 reads, respectively, with an approximate coverage of 21.9x, 14.2x and 10.9x, respectively, and a grand total of 156 million mate-pair reads representing 47x coverage.

ONT was used to generate 366,323 long reads, having 2,398,560,283 bp and coverage of 2.3x. The complete genome was sequenced to a depth of ~287x, using both Illumina and ONT platforms (Table 1). Coverage was based on the assumption that the peacock genome is 1 Gb in size.

Genome assembly

The first assembly was based on Illumina reads only, using the Abyss de novo assembler, which resulted in a genome size of ~932 Mb and an N50 of 1639 bp. Contig extension was

performed using ONT-generated reads, which gave scaffolds with an N50 of 14,748 bp. SSPACE AND PLATANUS were used to super-scaffold the assembled scaffold with mate-pair libraries, which generated a genome size of ~916 Mb and an N50 of 168,140 bp. Finally, gaps were closed using GAPCLOSER with mate-pair and PE-LI libraries, which generated a draft genome size of 1.02 Gb.

The draft genome assembly of *P. cristatus* comprises 179,346-bp scaffolds, with an N50 of 189,886 bp with 37 scaffolds, having a sequence length ≥1 Mb. Contigs greater than 5000 bp in length covered a genome of ~0.915 Mb, with an N50 of 0.23 Mb. In the assembled genome, there were ~0.4% non-ATGC characters (Table 2).

Repetitive genome elements and SSR markers

It was estimated that 75 Mb (7.33%) of the peacock genome consisted of repeat sequences (Table S1). About 56 Mb (5.5%) of class I retrotransposons were identified (long interspersed nuclear elements [LINEs], 4.7%; short interspersed nuclear elements [SINEs], 0.08%; and total LTR elements, 0.72%). Subsequently, 7,277,390 bp (0.71%) class II DNA transposons and 467,719 (0.05%) unclassified elements were identified (Table S1). The median percentages of LINEs, SINEs, LTR, DNA, unknown and total masked bases of other avian birds were 3.94, 0.11, 1.31, 0.22, 0.85 and 6.93, respectively (Table S2). A total of 399,493 SSRs were obtained from the peacock genome assembly. The largest fraction of SSRs identified were mononucleotides (60.04%), followed by tetranucleotides (26%), dinucleotides (8.51%), trinucleotides (4.31%), pentanucleotides (1.03%), and hexanucleotides (0.13%). Among these SSRs, A (49.2%) and T (44.9%) accounted for 94.1% of the mononucleotide repeats. AT (23.8%), TA (16.5%), TG (13.7%), AC (10.6%) and CA (10.32%) accounted for 75% of the dinucleotide repeats, whereas TTG (9.9%), AAT (9.6%), AAC (9.4%), TTA

(7.1%), ATT (4.5%), TAA (3.5%), CAA (3.1%) and GGA (2.69%) accounted for 49.7% of the trinucleotide repeats (Table S3).

Gene prediction and annotation

A total of 23,153 proteins were predicted from the assembled draft peacock genome using AUGUSTUS. Of these, 21,854 (94.4%) predicted proteins showed homology to other sequences from the NCBI NR database (Fig. 3). The top four organisms with which peacock proteins showed homology were *G. gallus* (11,398 proteins), *M. gallopavo* (4,059 proteins), *Amazona aestiva* (blue-fronted Amazon parrot; 1352 proteins), and *Anas platyrhynchos* (mallard duck; 849 proteins). Detailed annotations of all proteins are available in Table S4.

Gene Ontology (GO) descriptions were assigned for 18,294 (79%) peacock proteins. Of these, 14,489 proteins were identified as having molecular function; 11,678 as biological processes, and 13,735 proteins as cellular components (Table S4).

A total of 4,091 (17.7%) peacock proteins had pathway information from the KEGG database (Table S5), whereas 20,937 (88.1%) peacock proteins were similar to KOG annotations (Table S6). When peacock proteins were searched against human proteins, gene family expansions were found in cell morphogenesis, neuronal projection and development and GTPases (Table S7 and Fig. S3).

Analysis of avian protein families

From a total of 748,544 protein sequences from 49 avian species, 653,497 protein sequences were found to have a length of 100 amino acids or greater (Table S8A). Based on their level of identity, CD-HIT clustered the proteins into 114,121 gene clusters. Of these, 68 highly homologous gene clusters were present as single copies in all the 49 avian species (Table

S8B and Table S8C). We also observed 13,860 peacock protein clusters that were not clustered with other avian species (Table S8D).

Phylogenetic analysis

Phylogenetic analysis of 48 avian species and peacock proteins showed *P. cristatus* to be clustered in a clade with *G. gallus, M. gallopavo, A. platyrhynchos, Tinamus guttatus* (white-throated tinamou), and *Struthio camelus* (ostrich). This is the largest clade, with six species, having bootstrap support of 100. All species within this clade, except the mallard duck, are flightless or low flying birds. Bootstrap support between *P. cristatus* and *G. gallus* was 96, followed by *M. gallopavo*, with bootstrap support of 100 (Fig. 4).

Comparison with other species and databases

When searching Pfam for conserved protein domains between the predicted proteins from peacock, chicken and turkey, it was revealed that about 81% of domains were common to these three species (Fig. 5, Table S9). Compared with the total number of Pfam domains from these three species, 94%, 98.4% and 99.7% Pfam domains were present in peacock, chicken and turkey, respectively, but 255, 69 and 14 Pfam domains were absent between the species comparisons, respectively (Table S9H).

There were 15,470 (78%), 12,794 (85%) and 11,745 (85%) of the peacock, chicken and turkey proteins were found to match with Pfam domains, respectively (Table S9). Domain comparisons between these species showed gene family expansions such as kinases, zinc finger proteins, GTPases, and others, in either one of the species (Fig. 6).

A total of 9,974 peacock proteins were annotated in all four databases (NCBI NR, KOG, Pfam and GO) (Fig. 7). When reordered for the generation of pseudo-chromosomes, 597 Mb

of the assembled peacock genome was reordered peacock genome compared with the 1.21-Gb masked chicken genome [40] (Fig. 8).

Around 60 different avian species have been sequenced using various sequencing technologies (Table S10). The depth of these sequences varies, from as low as 6x to as high as 390x coverage. These results, which were obtained using different bioinformatics methods to assemble the sequencing data, are measured as scaffold N50; i.e., from 30 Kb to 14 Mb.

Discussion and conclusions

In recent years, there has been a rapid surge in the de novo genome sequence assembly of diverse species [41]. This surge has largely been driven by a more affordable cost per base sequencing, and the development of smarter algorithms that have been refined and equipped to handle large datasets [42–44]. The challenge for newer genome analysis pipeline is to generate assemblies with lower contig numbers and longer contigs per genome. To achieve this, technologies that generate longer reads or greater read depths are very helpful [45]; but the use of combinations of different sequencing technologies also plays a significant role in improving genome assemblies [46] (Table S10). Libraries generated using more than one type of chemistry have been found to generate superior assemblies [47], and have been shown to reduce the number of scaffolds – even with very low coverage. Thus, we need to consider combinations of sequencing technologies, along with the use of different bioinformatics software programs, to obtain assemblies with fewer numbers of scaffolds, or which are closer to chromosome-level sequencing [48].

Compared with other avian genomes [49], the sequencing depth of 290x that we achieved for the peacock is one of the highest. The final draft peacock genome assembly resulted in an N50 of 0.23 MB. Including 2.3x of reads generated by ONT helped to improve the assembly

by reducing the number of scaffolds by 26.2% and increasing the scaffold and contig N50s by about 50.7% and 115%, respectively.

The draft assembly contained fewer than 0.4% unknown nucleotides, which is very low for a draft assembly. Our hybrid peacock assembly outperforms the currently available draft peacock assembly (Table S11) by sequencing six different libraries, including long reads from ONT, and 2.1-fold increased sequencing data generation. Greater sequencing depth and the use of multiple libraries enabled us to obtain a better assembly with 6.6-fold fewer scaffolds and an improvement in N50 length by 9.1-fold. The longest scaffold in our assembly is 8.7-fold longer than in the previously published draft assembly, and has a 5-fold lower percentage of non-ATGC. Thus, for the first time in avian genomics, we have demonstrated how low-cost, third-generation sequencing data generated by ONT can help to improve draft genome assembly. Assemblies with longer scaffolds will further help us to understand more about organisms with structurally complex genomic regions, repeat elements and isoforms [39].

Our confidence in the peacock proteins predicted from our assembly was strengthened when we discovered that about 95% of them showed significant homology to various genomic features from different databases (Fig. 7). Based on proteins conserved across the avian species, our phylogenetic analysis revealed that the peacock is most closely related to the chicken, followed by turkey and duck. This concurs with previous data based on mitochondrial phylogeny [50]. Thus, our genome sequence provides further insight into the peacock's genetic lineage and evolution with respect to other avian species. The estimated median divergence time of *P. cristatus* from *G. gallus* is about 35 million years ago (MYA), whereas the divergence time estimated between *G. gallus* and *M. gallopavo* is about 37 MYA [51]. The large gap between peacock and other avians may be attributed to the non-

availability of other avian genome sequences. The gap may be closed by sequencing other avian species.

Among the vertebrates, it has been observed that variations in transposable elements (TEs) between avians are very low [52] (Table S8). The genome complexities of a species are influenced by the TEs that are believed to play a crucial role [53]. In this peacock genome assembly, inclusion of ONT long-read sequences has significantly improved the assembly, thus helping to resolve repetitive regions across the genome. The roles of TEs in the development and evolution of the peacock should be further explored.

Information about the peacock genome will be valued, and may be explored, by avian enthusiasts to further understand the avian world. Though not yet critically endangered in India, the wild peafowl population is declining because of massive deforestation, habitat loss [54], and increased poaching for their meat and feathers. Our *P. cristatus* genome sequencing initiative is not only valuable from a conservational viewpoint, but also to preserve the history and heritage that is associated with this bird, which bears a strong attachment to the national psyche.

Availability of supporting data

The data sets supporting the results of this article are available on the study website [55] and the *GigaScience* GigaDB repository [58].

Raw reads (Illumina and ONT) are available in the Sequence Read Archive (SRA) database, and the whole genome shotgun project has been deposited at GenBank under SRA submission ID SUB3108024, Bioproject PRJNA413288, and biosamples SUB3108018/SAMN07739105:SKPea2016_SI, SUB3108017/SAMN07739104:SKPea2016_LI, SUB3107930/SAMN07739101:FPL_3_5KB, SUB3108015/SAMN07739102:FPL_5_7KB,

SUB3108016/SAMN07739103:FPL_7_10KB, and SUB3108020/SAMN07739107:FPL_Nano (Table 1).

The de novo genome assembly can be accessed under SUB4504869/SAMN07739105.

Declarations

List of abbreviations

bp, base pair; Gb, Gigabase pairs; Kb, Kilobase pairs; LINE, Long interspersed nuclear elements; LTR, Long terminal repeat; Mb, Megabase pairs; ONT, Oxford Nanopore Technology; Pfam, Protein families; SINE, Short interspersed nuclear elements; SSR, Simple sequence repeat; WGS, whole genome sequencing.

Ethics approval and consent to participate

Not applicable.

Consent for publication

Not applicable

Competing interests

The authors declare that they have no competing interests.

Funding

R.D. provided partial funding. This work was also supported by Estonian Research Council ETAG grants IUT 34–4 for B.R.

Authors' contributions

R.D., A.S. and K.P. performed the wet lab experiments; R.D. designed the work plan, experiments and logistics; S.S., V.R., K.P., S.G., I.M. and A.R. assisted with the work; R.S. provided bird samples; B.R. and S.K. analyzed and interpreted the data, and drafted the manuscript. S.K. oversaw the whole project. All authors read and approved the final version of the manuscript.

Acknowledgements

We acknowledge the Department of Biochemistry, AIIMS, New Delhi, India, for providing space and infrastructure to carry on the work. We would like to thank Genotypic Technology for providing sequencing services.

Tables

Table 1. Raw data statistics of peacock genome reads generated by Illumina HiSeq and Oxford Nanopore Technology

Sample	Platform	Library and chemistry	Number of reads	Coverage	SRA ID
SO_6221_SKPea2016_SI	HiSeq	PE – SI (150 * 2)	489114747	146.73	SUB3108018, SAMN07739105
SO_6221_SKPea2016_LI	HiSeq	PE – LI (150 * 2)	302884819	90.87	SUB3108017, SAMN07739104
SO_6221_FPL_3_5KB	HiSeq	MP (150 * 2)	72915033	21.87	SUB3107930, SAMN07739101
SO_6221_FPL_5_7KB	HiSeq	MP (150 * 2)	47440144	14.23	SUB3108015, SAMN07739102
SO_6221_FPL_7_10KB	HiSeq	MP (150 * 2)	36464628	10.94	SUB3108016, SAMN07739103
SO_6221_NP	ONT	5 - 341124	366323	2.3	SUB3108020, SAMN07739107

Abbreviations: KB, kilobases; LI, long insert; MP, mate-pair; ONT, Oxford Nanopore Technology; PE, paired-end; SI, short insert; SRA, Sequence Read Archive

Table 2. De novo assembly statistics of the peacock genome

Description	Contigs	ONT scaffolds	Super-scaffolds	GapClosed	>1000 Kb	>5000 Kb
Contigs	685,241	281,272	179,346	179,332	34,178	15,025
Maximum length	49,159	251,510	2,390,121	2,488,982	2,488,982	2,488,982
Minimum length	300	5	265	265	1,000	5,000
Average length	1,360	3,250	5,111	5,729	-	-
Total length	932,162,464	914,363,908	916,720,956	1,027,510,962	954,449,349	915,342,012
Length ≥ 100 bp	685,241	281,271	179,346	179,332	34,178	15,025
Length ≥ 200 bp	685,241	281,271	179,346	179,332	34,178	15,025
Length ≥ 500 bp	616,120	186,433	93,727	93,718	34,178	15,025
Length ≥ 1 Kb	363,428	104,479	34,168	34,178	34,178	15,025
Length ≥ 10 Kb	1,591	24,748	9,249	10,310	10,310	10,310
Length ≥ 1 Mb	0	0	27	37	37	37
Non-ATGC #	350,325	42,696,911	49,169,831	4,043,129	4,040,790	3,986,487
Non-ATGC %	0.038	4.67	5.36	0.393	0.423	0.436
N50 value	1,639	14,748	168,140	190,304	218,023	232,312

Figure legends

Figure 1. Photograph of the Indian blue peacock (*Pavo cristatus*).

Figure 2. Detailed workflow for de novo whole genome assembly and annotation.

Figure 3. Peacock proteins showing homology. Pie chart showing significant similarity scores of peacock proteins against the National Center for Biotechnology Information Non-Redundant (NCBI NR) database. The pie chart colors are grouped based on the E-value scores from most significant E-value of 0.0 (red) going clockwise to least significant of about 1E-5 (blue).

Figure 4. Phylogenetic tree generated from homologous proteins from 49 different avian species.

Figure 5. Venn diagram showing common and absent protein family domains (Pfam) between peacock, chicken and turkey proteins.

Figure 6. Heatmap showing protein families (Pfam) distributed in peacock, chicken or turkey species. The number represents the Pfam domain count predicted from the protein sequences. Pfam domains of 50 and above identified in any one of the species are compared in the heatmap.

Figure 7. Venn diagram showing peacock proteins with significant homology to the NCBI NR database, the EuKaryotic Orthologous Groups (KOG) database, and protein family (Pfam) and Gene Ontology (GO) ontologies.

Figure 8. Circular image of the assembled peacock genome, aligned against the *Gallus gallus* genome. The right side of the image represents the reference chicken genome; left side represents the peacock genome.

References

1. Brickle NW. Habitat use, predicted distribution and conservation of green peafowl (*Pavo muticus*) in Dak Lak Province, Vietnam. *Biological Conservation*. 2002;105:189–97.

2. Jackson CE. Peacock. London: *Reaktion*, 2006.

3. Kadgaonkar, Shivendra B. The peacock in ancient Indian art and literature. *Bulletin of the Deccan College Research Institute*. 1993;53:95–115.

4. Gadagkar R. Is the peacock merely beautiful or also honest? *Current Science*. 2003;85:1012–20.

5. Kushwaha S, Kumar A. A Review on Indian Peafowl (*Pavo cristatus*) Linnaeus, 1758. *Journal of Wildlife Research*. 2016;4:42–59.

6. Hillier LW, Miller W, Birney E et al. Sequence and comparative analysis of the chicken genome provide unique perspectives on vertebrate evolution. International Chicken Genome Sequencing Consortium (ed.). *Nature*. 2004;432:695–716.

7. Zhang G, Jarvis ED, Gilbert MTP. A flock of genomes. *Science*. 2014;346:1308–9.

8. Bejerano G, Pheasant M, Makunin I et al. Ultraconserved elements in the human genome. *Science*. 2004;304:1321–5.

9. Burt DW. Emergence of the Chicken as a Model Organism: Implications for Agriculture and Biology. *Poultry Science*. 2007;86:1460–71.

10. Furlong RF. Insights into vertebrate evolution from the chicken genome sequence. *Genome Biology*. 2005;6:207.

11. Edmunds S. Hiseq 4000 Sequencing protocol v1 (protocols.io.q58dy9w). *protocols.io* 2018, https://dx.doi.org/10.17504/protocols.io.q58dy9w

12. Andrews S. FastQC A Quality Control tool for High Throughput Sequence Data. 2015. http://www.bioinformatics.babraham.ac.uk/projects/fastqc

13. Kajitani R, Toshimoto K, Noguchi H et al. Efficient de novo assembly of highly heterozygous genomes from whole-genome shotgun short reads. *Genome Research*. 2014;24:1384–95.

14. Metrichor. https://nanoporetech.com/products/metrichor Accessed 1st Feb 2019

15. Loman NJ, Quinlan AR. Poretools: a toolkit for analyzing nanopore sequence data. *Bioinformatics*. 2014;30:3399–401.

16. Li H, Durbin R. Fast and accurate short read alignment with Burrows-Wheeler transform. *Bioinformatics*. 2009;25:1754–60.

17. Birol I, Jackman SD, Nielsen CB et al. De novo transcriptome assembly with ABySS. *Bioinformatics*. 2009;25:2872–7.

18. Boetzer M, Pirovano W. SSPACE-LongRead: scaffolding bacterial draft genomes using long read sequence information. *BMC Bioinformatics*. 2014;15:211.

19. Boetzer M, Henkel CV, Jansen HJ et al. Scaffolding pre-assembled contigs using SSPACE. *Bioinformatics* 2010;27:578–9.

20. Luo R, Liu B, Xie Y et al. SOAPdenovo2: an empirically improved memory-efficient short-read de novo assembler. *GigaScience* 2012;1(1):18

21. Marçais G, Kingsford C. A fast, lock-free approach for efficient parallel counting of occurrences of k-mers. *Bioinformatics*. 2011;27:764–70.

22. Smit A, Hubley R, Green P. RepeatMasker Open-4.0. 2013-2015. www.repeatmasker.org. Accessed 1st Feb 2019

23. Thiel T. MISA - MIcroSAtellite identification tool. 2012. http://pgrc.ipk-gatersleben.de/misa/ Accessed 1st Feb 2019

24. Stanke M, Diekhans M, Baertsch R et al. Using native and syntenically mapped cDNA alignments to improve de novo gene finding. *Bioinformatics*. 2008;24:637–44.

25. Altschul SF, Gish W, Miller W et al. Basic local alignment search tool. *Journal of molecular biology*. 1990;215:403–10.

26. Moriya Y, Itoh M, Okuda S et al. KAAS: an automatic genome annotation and pathway reconstruction server. *Nucleic Acids Research*. 2007;35:W182–5.

27. Nordberg H, Cantor M, Dusheyko S et al. The genome portal of the Department of Energy Joint Genome Institute: 2014 updates. *Nucleic Acids Research*. 2013;42:D26–31.

28. El-Gebali S, Mistry J, Bateman A et al. The Pfam protein families database in 2019. *Nucleic Acids Research*. 2018;47:D427–32.

29. Zhang G, Li B, Li C et al. Comparative genomic data of the Avian Phylogenomics Project. *GigaScience*. 2014;3:26.

30. Jarvis ED, Mirarab S, Aberer AJ et al. Phylogenomic analyses data of the avian phylogenomics project. *GigaScience*. 2015;4:4.

31. Fu L, Niu B, Zhu Z et al. CD-HIT: accelerated for clustering the next-generation sequencing data. *Bioinformatics*. 2012;28:3150–2.

32. Larkin MA, Blackshields G, Brown NP et al. Clustal W and Clustal X version 2.0. *Bioinformatics*. 2007;23:2947–8.

33. Talavera G, Castresana J. Improvement of Phylogenies after Removing Divergent and Ambiguously Aligned Blocks from Protein Sequence Alignments. *Systematic Biology*. 2007;56:564–77.

34. Felsenstein J. PHYLIP (Phylogeny Inference Package) Version 3.57c. 1993 http://evolution.genetics.washington.edu/phylip.html Accessed 1st Feb 2019

35. Nguyen L-T, Schmidt HA, von Haeseler A et al. IQ-TREE: A Fast and Effective Stochastic Algorithm for Estimating Maximum-Likelihood Phylogenies. *Molecular Biology and Evolution.* 2014;32:268–74.

36. FigTree, a graphical viewer of phylogenetic trees. http://tree.bio.ed.ac.uk/software/figtree/ Accessed 1st Feb 2019

37. Tamazian G, Dobrynin P, Krasheninnikova K et al. Chromosomer: a reference-based genome arrangement tool for producing draft chromosome sequences. *GigaScience* 2016;5(1):38.

38. Frith MC, Kawaguchi R. Split-alignment of genomes finds orthologies more accurately. *Genome Biology*. 2015;16:106.

39. Krzywinski M, Schein J, Birol I et al. Circos: an information aesthetic for comparative genomics. *Genome research*. 2009;19:1639–45.

40. Warren WC, Hillier LW, Tomlinson C et al. A New Chicken Genome Assembly Provides Insight into Avian Genome Structure. *G3: Genes, Genomes, Genetics*. 2016;7:109–17.

41. Peona V, Weissensteiner MH, Suh A. How complete are “complete” genome assemblies?-An avian perspective. *Molecular Ecology Resources*. 2018;18:1188–95.

42. Muir P, Li S, Lou S et al. The real cost of sequencing: scaling computation to keep pace with data generation. *Genome Biology*. 2016;17:53.

43. Goodwin S, McPherson JD, McCombie WR. Coming of age: ten years of next-generation sequencing technologies. *Nature Reviews Genetics*. 2016;17:333–51.

44. Levy SE, Myers RM. Advancements in Next-Generation Sequencing. *Annual Review of Genomics and Human Genetics*. 2016;17:95–115.

45. Lu H, Giordano F, Ning Z. Oxford Nanopore MinION Sequencing and Genome Assembly. *Genomics, Proteomics & Bioinformatics*. 2016;14:265–79.

46. Rice ES, Green RE. New Approaches for Genome Assembly and Scaffolding. *Annual Review of Animal Biosciences*. 2018;7.

47. Weissensteiner MH, Pang AWC, Bunikis I et al. Combination of short-read, long-read, and optical mapping assemblies reveals large-scale tandem repeat arrays with population genetic implications. *Genome Research*. 2017;27:697–708.

48. Sohn J, Nam J-W. The present and future of de novowhole-genome assembly. *Briefings in Bioinformatics*. 2016:bbw096.

49. Zhang G, Li C, Li Q et al. Comparative genomics reveals insights into avian genome evolution and adaptation. *Science*. 2014;346:1311–20.

50. Dalloul RA, Long JA, Zimin AV et al. Multi-Platform Next-Generation Sequencing of the Domestic Turkey (Meleagris gallopavo): Genome Assembly and Analysis. Roberts RJ (ed.). *PLoS Biology*. 2010;8:e1000475.

51. Kumar S, Stecher G, Suleski M et al. TimeTree: A Resource for Timelines, Timetrees, and Divergence Times. *Molecular Biology and Evolution*. 2017;34:1812–9.

52. Sotero-Caio CG, Platt RN, Suh A et al. Evolution and Diversity of Transposable Elements in Vertebrate Genomes. *Genome Biology and Evolution*. 2017;9:161–77.

53. Kapusta A, Suh A. Evolution of bird genomes-a transposon's-eye view. *Annals of the New York Academy of Sciences*. 2016;1389:164–85.

54. Ramesh K, McGowan P. On the current status of Indian Peafowl Pavo cristatus (Aves: Galliformes: Phasianidae): keeping the common species common. *Journal of Threatened Taxa*. 2009;1:106–8.

55. Additional data for De novo genome assembly of the peacock. https://biit.cs.ut.ee/supplementary/peacock/ Accessed 1st Feb 2019

56. Reimand J, Arak T, Adler P et al. g:Profiler—a web server for functional interpretation of gene lists (2016 update). *Nucleic Acids Research.* 2016;44:W83–9.

57. Kolde R, Vilo J. GOsummaries: an R Package for Visual Functional Annotation of Experimental Data. *F1000Research.* 2015, DOI: 10.12688/f1000research.6925.1.

58. Dhar R, Seethy A, Pethusamy K, Singh S, Rohil V, Purkayastha K, Mukherjee I, Goswami S, Singh R, Raj A, Srivastava T, Acharya S, Rajashekhar B and Karmakar S (2019): Supporting data for "De novo genome assembly of the Indian Blue Peacock (Pavo cristatus), from Oxford Nanopore and Illumina sequencing" *GigaScience Database*. http://dx.doi.org/10.5524/100559

Figure 1

Click here to access/download;Figure;Fig 1. Peacock bird.jpg

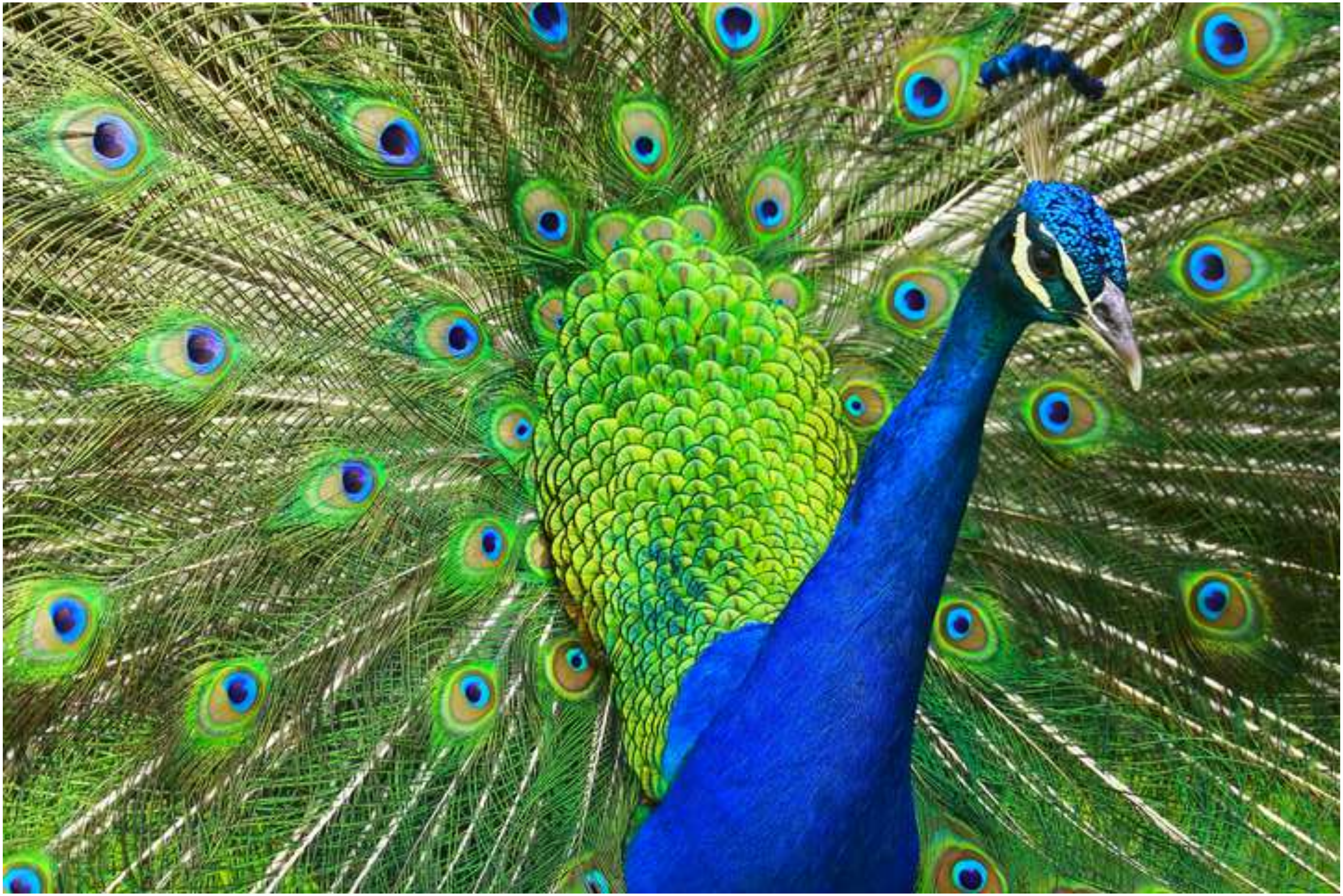

Click here to access/download;Figure;Fig 2_Workflow.pdf

Figure 2

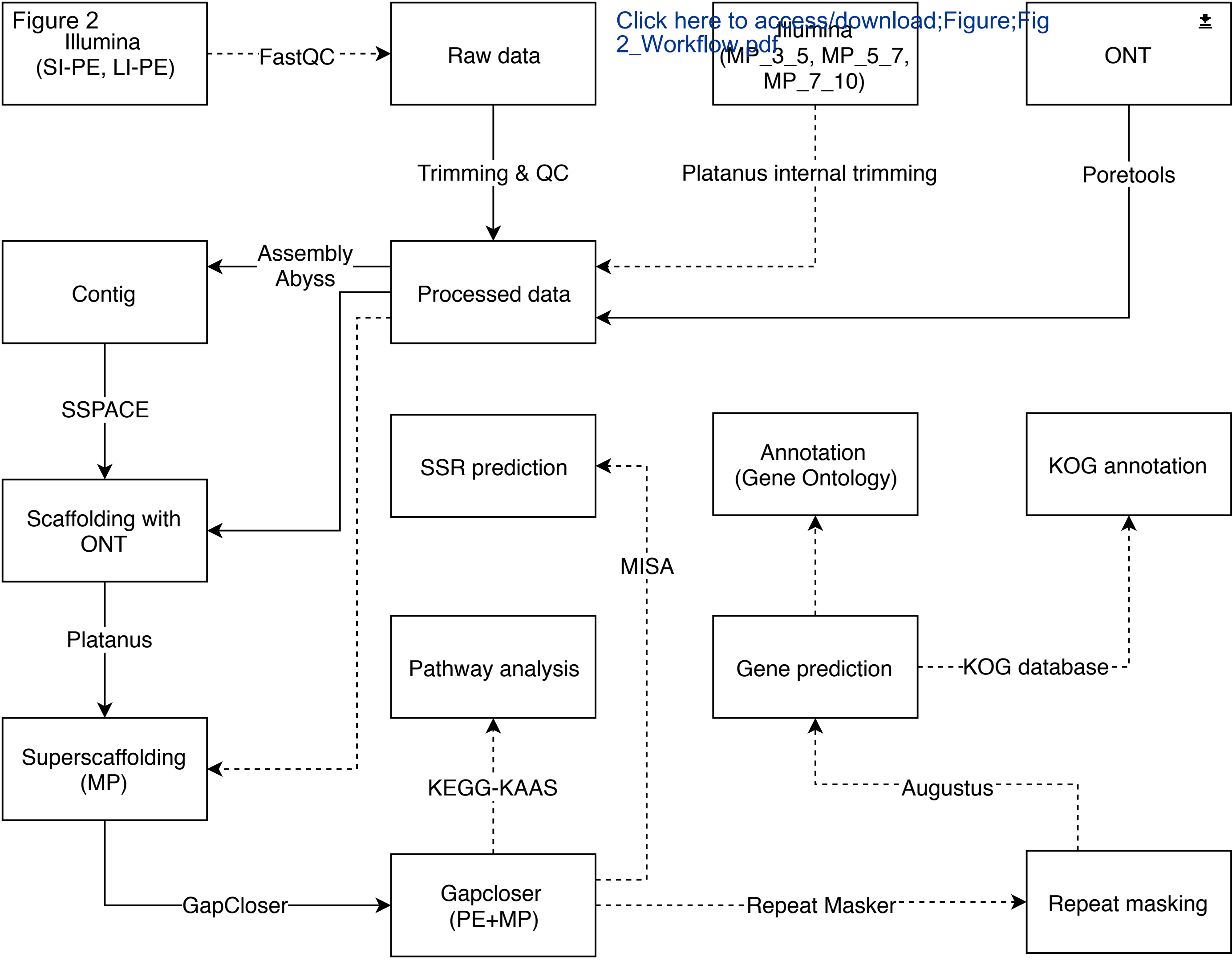

Figure 3

Click here to access/download;Figure;Fig 3 Similarity scores against the Uniprot database.pdf

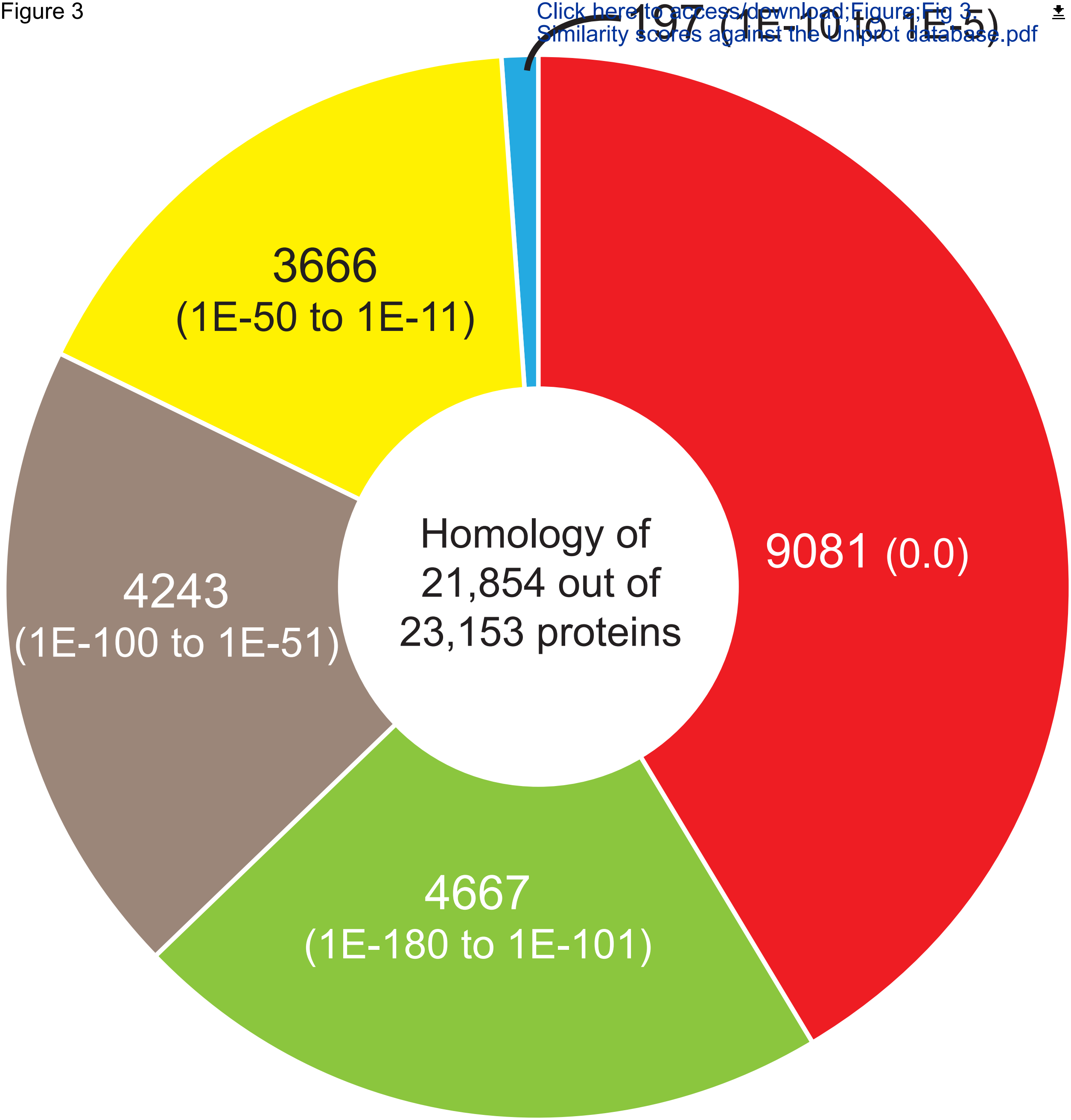

Figure 4

Click here to access/download;Figure;Fig 4. Phylogeny of avian species.pdf

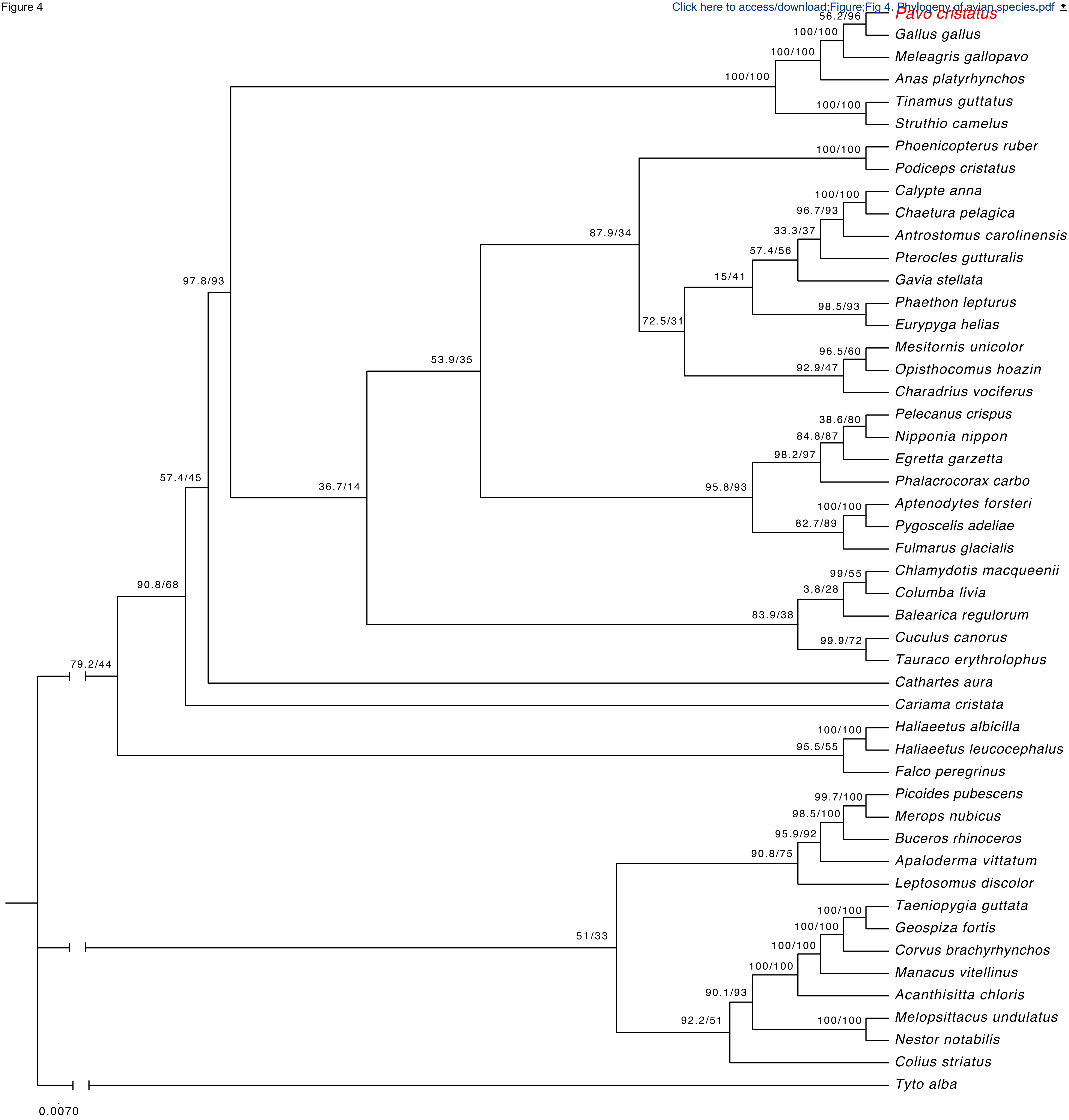

Figure 5

Click here to access/download;Figure;Fig 5. Unique Pfam domains common with chicken

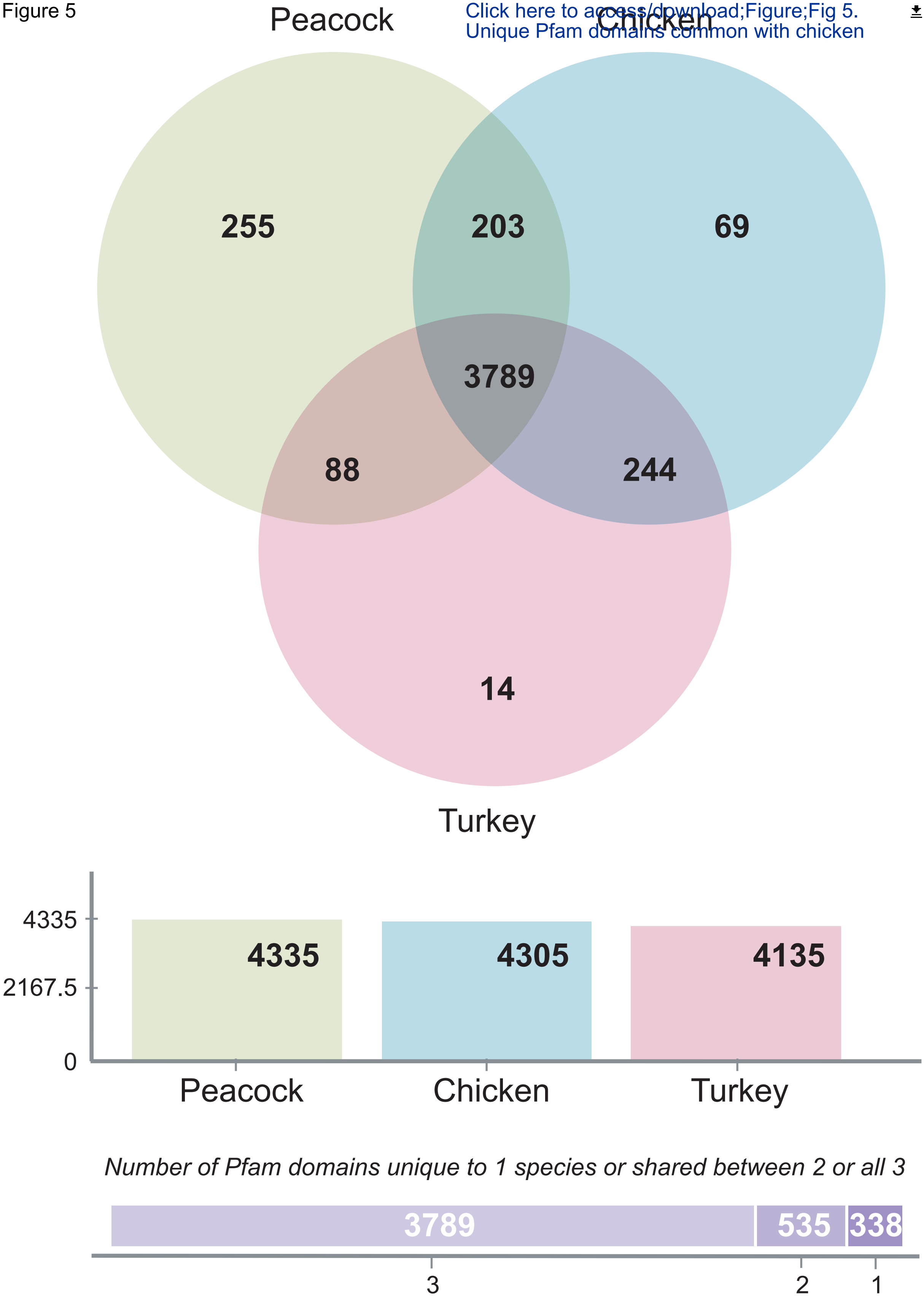

Figure 6

Click here to access/download;Figure;Fig6. Heatmap

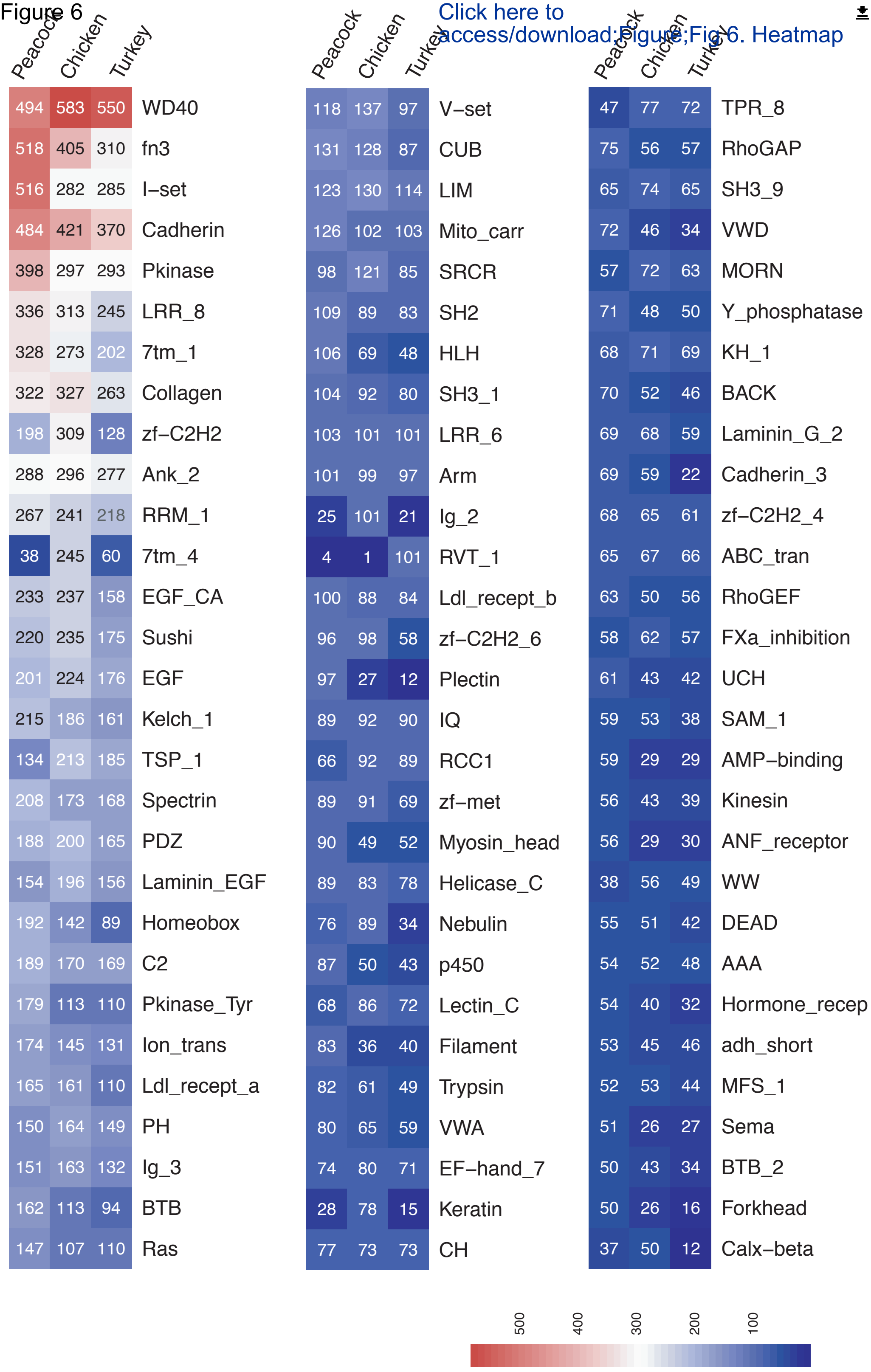

Figure 7

Click here to access/download;Figure;Fig 7. Venn of annotations.pdf

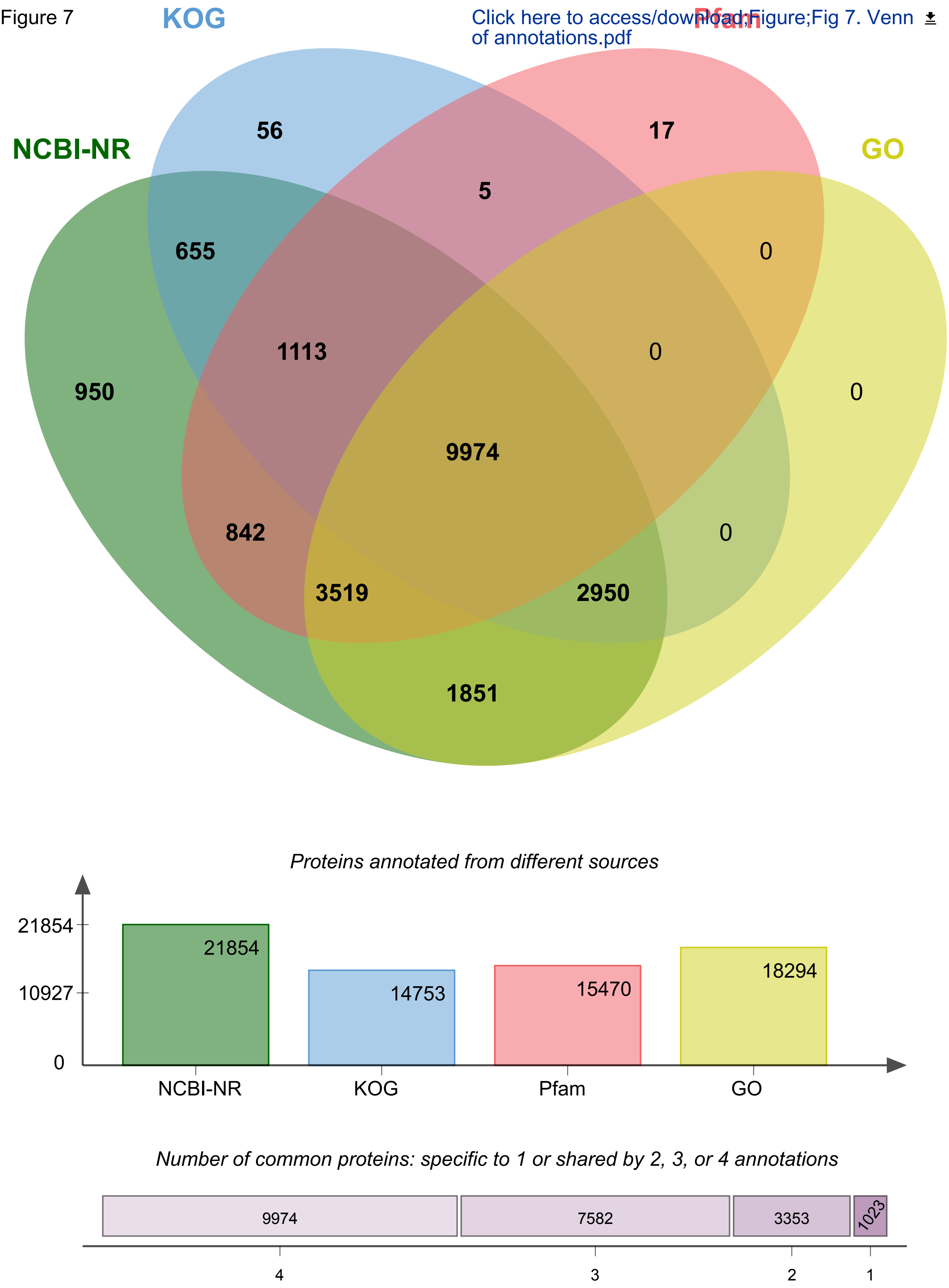

Figure 8

Click here to access/download;Figure;Fig 8. Peacock scaffolds against Gallus circular synteny.png

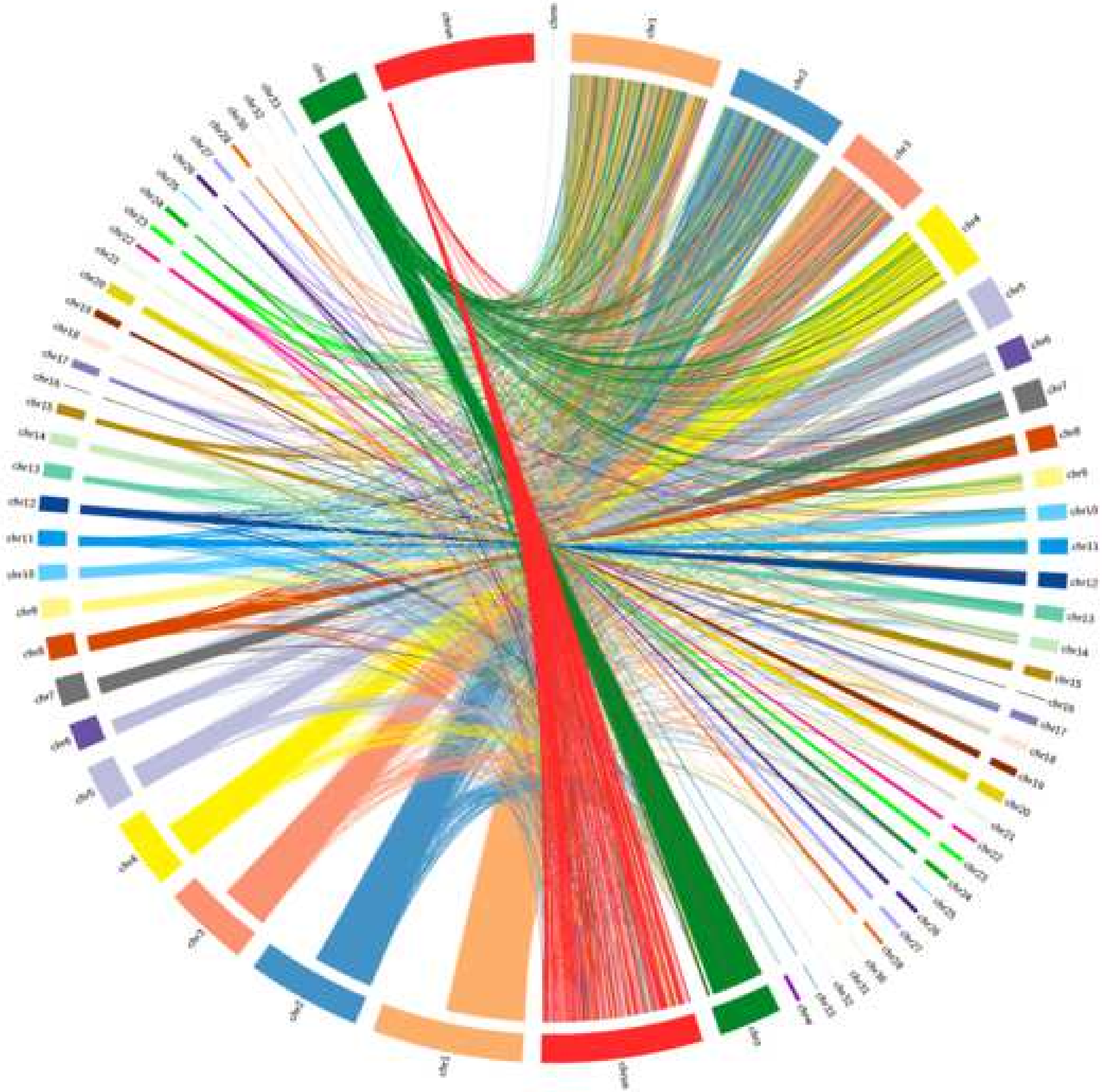

Click here to access/download
Supplementary Material
Table_S1_ReadStats_Table_S2_TEs.xlsx

Supplementary Material: Table S3

Click here to access/download
Supplementary Material
Table_S3_Repeats.xlsx

Click here to access/download
Supplementary Material
Table_S4_Gene_annotations_of_peacock_proteins.xlsx

Supplementary Material: Table S5

Click here to access/download

Supplementary Material

Table_S5_KEGG_annotation.xlsx

Click here to access/download
Supplementary Material
Table_S6_KOG_annotation.xlsx

Click here to access/download
Supplementary Material
Table_S7_BlastVsHumanProteins.xlsx

Supplementary Material: Table S8

Click here to access/download
Supplementary Material
Table_S8_Orthologous_proteins

Supplementary Material: Table S9

Click here to access/download
Supplementary Material
Table_S9_Pfam_Analysis.xlsx

Supplementary Material: Table S10

Click here to access/download
Supplementary Material
Table_S10_Bird_Species_with_counts.xlsx

Click here to access/download
Supplementary Material
Fig S1. Proteins showing similarity to Pfam domains.pdf

Supplementary Material: Figure S2

Click here to access/download

Supplementary Material

Fig S2. Gene Ontololgy of top 10 WGS.png

Click here to access/download
Supplementary Material
Fig S3.Peacock vs Human_GO.pdf

Supplementary Material: Table S11

Click here to access/download
Supplementary Material
Table_S11_Peacock_assembly_comparison.xlsx

Supplementary Material: Table S10

Click here to access/download
Supplementary Material
Table_S10_Bird_Species_with_counts.xlsx

Supplementary Material description

Click here to access/download
Supplementary Material
Supplementary_Description of tables and figures.docx
