# Supplementary material for: De novo assembly of the Indian blue peacock (Pavo cristatus) genome using Oxford Nanopore technology and Illumina sequencing
Source: Gigascience. 2019 May 11;8(5):giz038. doi: 10.1093/gigascience/giz038 (PMC6511069; doi:10.1093/gigascience/giz038)
Supplement: Supplemental Files [file giz038_supplemental_files.zip › Supplementary_Description of tables and figures.docx]

**Supplementary tables and figures**

**Tables**

**Table S1.** Statistical analysis of repeat masking for the peacock genome

**Table S2.** Statistical analysis of repeats in the peacock genome compared with other bird species

**Table S3A.** Overview of simple sequence repeats (SSRs)

**Table S3B.** Simple sequence repeats (SSRs) identified in the peacock genome

**Table S4.** Protein database annotations for predicted peacock genes, using BLAST software

**Table S5.** Kyoto Encyclopedia of Genes and Genomes (KEGG) annotations for all peacock proteins

**Table S6.** EuKaryotic Orthologous Groups (KOG) annotations for all peacock proteins

**Table S7.** Peacock proteins showing remote orthologous sequence relationships with human proteins, using BLAST software

**Table S8A.** Count of proteins in different bird species

**Table S8B.** Orthology, clustering and annotation of proteins from different bird species

**Table S8C.** Single-copy orthologous proteins present in all bird species

**Table S8D.** Proteins uniquely present in peacock

**Table S9A-G.** Protein family (Pfam) protein domains identified in peacock, chicken and turkey

**Table S10.** Raw sequencing data, total scaffolds with N50 obtained for different avian species

**Table S11.** Comparison of two different peacock assemblies

**Figures**

**Fig S1.** Number of proteins showing similarity to protein family (Pfam) domains

**Fig S2.** Distribution of top ten gene ontologies in biological process, cellular component and molecular function categories represented as a pie chart.

**Fig S3.** Comparison of peacock and human protein orthologs and their Gene Ontology annotations, represented as a word cloud to show significant ontology descriptors.

Geneids were converted to Ensembl Ids using G:Convert [56] and gene ontologies were evaluated using GO:Summaries [57].
