# Supplementary material for: De novo assembly of the Indian blue peacock (Pavo cristatus) genome using Oxford Nanopore technology and Illumina sequencing
Source: Gigascience. 2019 May 11;8(5):giz038. doi: 10.1093/gigascience/giz038 (PMC6511069; doi:10.1093/gigascience/giz038)
Supplement: Response_to_Reviewer_Comments_Original_Submission.pdf [file giz038_response_to_reviewer_comments_original_submission.pdf]

Author's Response To Reviewer Comments

Close

Reply to reviewer’s comments (see also attached response letter).

We thank the editors and the reviewer for reviewing our manuscript titled “ De-novo genome assembly of Indian Blue peacock (Pavo Cristatus) Oxford Nanopore and Illumina sequencing . Following reviewers comments and suggestions, we have modified the manuscript incorporating all the necessary changes. Additional figures are incorporated as per the reviewer suggestions while non relevant items are removed. File containing a point by point reply to reviewers questions was also attached. We also uploaded the raw data at NCBI SRA as requested by one
of the reviewer.

1: “of the Indian Blue Peacock”
Response: The above has been changed

3: [remove “reads”, unnecessary]
Response: Reads have been removed from the title

26-27: “are native to South Asia”
Response: The authors accepted this suggestion and the same has been modified in the article.

27-28: “The peacock has been described as one of the most elegant, majestic, and beautiful bird species.”
Response: The authors thank the reviewers for suggesting this change and as per their expert suggestions, the above sentence has been included in the article.

38,40: standardizing how you refer to Oxford Nanopore sequencing would be helpful. “Oxford Nanopore technology” vs. “long read Nanopore technologies”
Response: Authors thank the reviewer for raising the concern and we have now used “Oxford Nanopore technology” in the article. Further a detail discussion of how ONT (Oxford Nanopore technology) long read chemistry was helpful to improve the genome assembly is discussed in the conclusion section. The authors want to humbly state that a hybrid approach of genome assembly using short reads along with long reads seems to improve genome quality that otherwise might not be achieved using just only one of the type. This could be due to repetitive elements in the genome.

60: comparing peacock proteins to human seems like much less informative than comparisons to chick and turkey.
Response: When we submitted the manuscript under research category previously one of the reviewers suggested to do a comparison of peacock proteins to human. Hence we included the results of comparison against human proteins. In this present manuscript, we have included the comparisons to Chicken and Turkey.

73-84: I’m not certain that the review of avian genomics is helpful. This could be condensed to a couple of sentences with appropriate citations.
Response: Authors agree with the reviewer’s suggestion. This section has been condensed and references have been included.

89-90: “The long read chemistry …” I think this sentence is supposed to end with “repeat rich regions of the genome”.
Response: Authors agree with the reviewer’s suggestion. This has been modified in the manuscript as per reviewers comment.

90-91: remove, redundant with the rest of the paragraph or replace as the first sentence in the paragraph

Response: Authors thanks the reviewers for his comments and this has been corrected in the manuscript.

91-92: “Comparative genomics”
Response: This has been modified.

93-95: How will knowledge of the sex determination genes aid in selective breeding? I’m not certain this
Response: Authors agree with the reviewers suggestions and taking into duly consideration of their concern, this sentence has been modified in the updated manuscript.

97: “should improve”
Response: Authors assure the reviewer that this has been duly considered and modified in the manuscript

106: “10 seconds” (add space)
Response: Space has been added.

107: “10 minutes” (add space)
Response: Space has been added.

108: It’s not clear whether the Binding Buffer was added to the collection tube with the ReliaPrep column or the tube that contained the sample mixture.
Response: Authors confirmed the working protocol from the concerned investigators and concluded that binding buffer was added to the collection tube and the entire sample preparation was carried out strictly adhering to the manufacturers protocol. The same has been incorporated in the text.

126: approximate not approx.. Abbreviation is unnecessary here.
Response: Abbreviation has been removed from the manuscript

205-206: This is a run on sentence. Should end with “Metrichor V.2.43.1” followed by a citation or URL for the software.
Response: The authors agree with the concern raised and sentence has been modified with URL included.

224-227: This section should be simplified to one sentence and combined with the prior paragraph.
Response: The paragraph is merged and modified.

229-234: Citations needed for repeatmasker tool, augustus, and Uniprot protein database. Half of this paragraph is a repeat of a prior section and could be combined there.
Response: The paragraph is merged and references have been included.

253: URL or citation needed
Response: URL included.

257: I think you mean “selected” here, not “filtered”. To my understanding, “filtered” implies exclusion.
Response: The authors agree with the reviewer that the original sentence was misleading and as per their valued suggestions necessary correction has been made in the text. We thank the reviewer for this.

269: Citation needed for Pfam
Response: Citation is included.

282-284: This should be one sentence: “Draft chromosomes were constructed by aligning the assembled draft genome against the G. gallus with the Chromosomer tool” with a citation or URL for the Chromosomer tool.
Response: The authors agree with the reviewer on this and sentences are now merged as per their recommendation. Further URL for the tool is included in the text for readers.

304-315: Citations needed for Abyss, SSPACE, PLATANUS, GAPCLOSER tools.
Response: The tools have already been cited in 214-218.

318-320: Citation needed for the previously published peacock mitogenome.
Response: Authors want to state that information /data on Peacock mitogenome has not been included in thus present manuscript .

322-326: This section needs a thorough rewrite for clarity.
Response: Authors have seriously taken the positive feedback of the reviewers comments and this section has been rewritten and one more table has been included for comparison with other bird species for better clarity. We thank the reviewers for this critical suggestion.

329-334: This data could be easier presented in a table. The very few homologous genes identified with blast hits between the peacock and parrot and mallard genomes suggest that a too stringent blast search was used.
Response: Authors agree with the reviewers comment. New figures and tables have been included in the manuscript.

334: “Thirteen species had about 100-400 annotated proteins”. This is a misstatement of these results. The authors did not annotated genes in the other bird genomes. They identified homologous genes using a very stringent requirement of similarity. Again, this data would be better presented as a table or figure, ideally as a histogram with the various bird species binned by the number of blast hits. identified between each species and the genes from the peacock genome.
Response: The authors agree on this and appreciate the reviewers concerns. This section have been rewritten and modified in this updated manuscript. The significant results are represented as pie and venn chart, histograms with complete details in tables.

337: “overrepresented” It isn’t clear what criteria or method was used for overrepresentation here.
Response: The authors want to state that this was based on the count, now this section is modified.

346-350: The interpretation of the “overrepresented” categories here isn’t clear either.
Response: This section is modified.

374: If the majority of peacock genes (15K out of 23K) clustered by themselves (ie found no homolog in any of the 49 avian proteomes used here), then probably too stringent a blast search or clustering criteria were used for this analysis to be generally useful. This is supported by the fact that clustering the 750K protein sequences resulted in ~250K gene cluster, or about 3 genes per cluster. An alternate interpretation is that a large number of those 15K unique peacock genes are mis-annotations of some kind, and the reason they have no known homology is that they do not represent actual genic sequences. This is supported by the fact that a very low percentage of the annotated peacock genes were found to have Pfam domains (4335 out of 23000 or ~19% of annotated genes with a Pfam domain, see Fig. 1 in Holt and Yandell, 2011).
Response: The authors understood the reviewers concerns and addressed the necessary changes in the reviewed manuscript. These have been modified and the new figures have been included. We used CD-Hit to cluster the proteins with 70% similarity, we have tried different similarity cut-offs and below 70% CD-hit showed errors in clustering hence we had to report results at the above mentioned cutoff. CD-Hit clusters the sequences assuming there will be 70% continuous sequence similarity. If there are mutations between the sequences like substitutions, insertions, deletions this will fall outside the cluster. The approach of this method was to work on those proteins that are present in all bird genomes and make a phylogeny on the conserved pool of orthologs. Blast similarity approaches will yield different results but we may end up with shorter orthologs and the results may be completely different. In this article we present CD-Hit based clustering approach to instead of BLAST approach to avoid false positives clustering.

393-395: This sentence is hard to follow.
Response: The authors agree with the reviewers suggestions and hence the sentence is modified.

398: I don’t think you can say that this assembly is “improved” if it is the first published assembly for this species.
Response: Your statement of understanding is correct, hence the word improved have been removed from the sentence.

410-432: The last sentence in this paragraph is missing a period. There should be some analysis of the Kit and FGF proteins that the authors point to here. Are they conserved or divergent from chicken, from guinea fowl? The fact that they are present in the genome isn’t surprising or notable, since large number

of proteins share homology across large taxonomic distances. The first paragraph here, which discusses sexual selection is too long, and needs to be reduced to one or two sentences to highlight the peacock's historic role in the development of the theory of sexual selection.
Response: This paragraph have been removed since the literature talks about some other proteins and we have to investigate more about all these proteins and the transcriptome data will be better to reveal more about the coloration in the peacock bird.

437: "closeness" is hard to interpret here.
Response: This has been modified and made more clear for the readers. Authors want to thank reviewer for their suggestions.

445: citation for population decline and conservation status of the Indian peafowl population.
Response: The citation have been provided in the manuscript.

Close
