# Supplementary material for: De novo assembly of the Indian blue peacock (Pavo cristatus) genome using Oxford Nanopore technology and Illumina sequencing
Source: Gigascience. 2019 May 11;8(5):giz038. doi: 10.1093/gigascience/giz038 (PMC6511069; doi:10.1093/gigascience/giz038)
Supplement: Response_to_Reviewer_Comments_Revision_1.pdf [file giz038_response_to_reviewer_comments_revision_1.pdf]

Author's Response To Reviewer Comments

Close

Dear Dr. Scott,
We have addressed all the reviewer's comments and have made significant additional revisions as required by you and both the reviewers. We have removed content on the sexual selection from the manuscript. Below are point-by-point response for the queries raised by the reviewers.

1. Abstract: Page 2, 12-13: "Its length …" The level of detail in this sentence is inappropriate for an abstract, and it should be removed.

Reply: the sentence below is completely removed from abstract
"Its length varies from 92-125 centimeter (without train), weighing about 4-8 Kilograms and lives up to 20 years in the wild."

2. Page 3, 15: "Observation from our study showed…", rewrite as "Our study showed…"

Reply: The sentence has been corrected as suggested.

3. Page 3, 19: "Further a comparative genomics …" this sentence is grammatically incorrect

Reply: this sentence is modified to "Further predicted peacock proteins when compared with"

4. Page 3, 32: "amongst the clade of birds based on their ability to fly". I think you should just indicate the clade with which Pavo was grouped.

Reply: The sentence is modified to "Further, the phylogentic tree on the conserved genes from the avian species showed the P. cristatus amongst in the clade of G. gallus, M. gallopavo and A. platyrhynchos (mallard),"

5. Page 4, 27: "The avian genomics began …" This paragraph is still too much introduction and too general to be helpful for the paper. The phrase "The avian genomics" is grammatically incorrect.

Reply: The first 2 sentences are completely removed now the new paragraph starts as "The genome sequencing of the model organism Gallus gallus species (Chicken) (Hillier et al. 2004) and wide variety of avian species (Zhang et al. 2014). have provided a novel perspective on vertebrate genome evolution in better understanding number of distinct characteristics and the annotation of mammalian genomic regions."

6. Page 4, 55: "aves" I think this should be italicized.

Reply: In other published articles "aves" is used as "Aves". To keep in standard format we have changed to Aves without italics.

7. Page 5, 10-15: "We have unraveled …" I don't think that these are demonstrated in the results ("genomic signatures", "gene pools"). I think that "gene pools" is used incorrectly here.

Reply: The sentence is changed from "We have unraveled some of the genomic signatures and thus have reported unique gene pools of this bird by performing comparative genomics."
to
"The protein comparisons between the peacock, chicken and turkey will reveal proteins, conserved

domains and functional annotations common and absent between the species."

8. Page 9, 32-33: "The raw data were then base-called …" This reads like it is directly following the MinION library preparation and sequencing section, which it doesn't. It should either be re-written to fix this or the paragraphs should be re-ordered.

Reply: To resolve the issues the paragraphs are arranged under two new broad sections
Library preparation and sequencing
Raw data quality control and processing

9. Page 10, 41-46: "Gene models were predicted on a hard masked draft genome and further genes were predicted using AUGUSTUS" This sounds like gene models were predicted twice (once on the hard-masked genome and once using AUGUSTUS).

Reply: This sentence was corrected to
"Gene models were predicted on a hard masked draft genome using AUGUSTUS"

10. Page 14, 59: "Significant gene Ontology (GO)" Significance in this context implies statistical significance, but no statistical tests are presented. Throughout this section, sometimes results are presented as percentages or counts inconsistently.

Reply: Now the paragraph starts from Gene ontology. In the whole section the total protein numbers (% in brackets) are mentioned. This has been represented uniformly in this paragraph.

11. Page 15, 4-5: The meaning of the phrase "unique proteins" is unclear here, since you're just talking about the set of predicted proteins from the peacock genome.

Reply: The unique protein is changed to "peacock specific proteins" "absent between" or "not clustered with other species" in the entire manuscript

12. Page 15, 12-13: "showed expansions in ontologies" should be "showed expansions in GO categories".

Reply: The sentence has been modified as suggested.

13. Page 15, 12-13: Fig. S4 appears to be missing from the attachments in this document. I don't know why Table S7 is referenced here. Table S7 doesn't have GO terms or any other functional annotation information.

Reply: Fig. S4 was removed from the manuscript after previous revision. We have removed the Fig. S4 from the manuscript.

14. Page 15, 29-32: "With the stringent cutoff" This makes it sound like there were two cutoffs ¬– a stringent one and a lenient one. This result (13,860 genes unique to peacock) still seems to point to over-prediction in the peacock genes than actual unique genes.

Reply: The cutoff parameters for clustering were 70% alignment coverage and length difference of 0.7. With above cutoff we obtained 13860 clusters not clustering with other avian proteins. This could be due to the sensitivity of the CD-Hit tool to identify highly conserved proteins in avian species. BLAST similarity and further clustering them may result in less number of unique proteins. This will allow short sequences clustered with complete long sequences resulting in false positive results. Due to very low coverage of sequencing of some avian species which may have resulted in incomplete ORF predictions.

15. Page 17, 24-25: The timetree URL isn't the correct way to reference the tool.
http://www.timetree.org/faqs#q7

Reply: The following reference have been included in the references section of the manuscript
Kumar S, Stecher G, Suleski M, Hedges SB (2017) TimeTree: A Resource for Timelines, Timetrees, and Divergence Times. Mol Biol Evol 34 (7): 1812-1819

16. Page 17, 51-52: It isn't clear which methods or results section Fig. 8 is connected to. These results should be addressed before the Conclusions section of the paper.

Reply: This sentence has been moved to the results sections under Pfam.
"The domain comparisons between the species showed gene family expansions such as Kinases, Zn finger proteins, GTPases and others in either one of the aforementioned species (Fig. 6)."
The other figure order and also legends have been modified

17. Page 18, 5-6: This paragraph needs to be shortened to one or two sentences pointing out the importance of tail feathers in the biology of the peacock and relevant literature regarding genetic control of plumage that might inform future studies. The discussion of sexual selection is irrelevant to the results presented in this paper. This point has been repeatedly addressed by reviewers in the past two rounds of revision.

Reply: The following paragraph regarding sexual selection have been removed from the manuscript,
One of the most important task will be to characterize the genes involved in the coloration of the tail feather plumage in P. cristatus (Roulin et al. 2013). The peacock feathers have played a significant role in the mating and sexual selection. Peacock seems to defy the Darwinian laws of natural selection. These concern were raised by no other than Darwin himself. Hence, he proposed the theory of the sexual selection where the female can choose for a male with a certain phenotypic feature such as brilliant color or a long tail (Burgess 2001). Peacock's brilliantly colored long tail feathers seems to evolve at the cost of finding its female partner thereby contributing its beneficial genes, even at the cost of making itself vulnerable to predators. A female peafowl in turn tends to choose the mate with the largest and decorated plumage, which indirectly reflects its healthiness and capacity to wade off potential competitors. Thus understanding the formation of beautiful feathers from the genomic context will help in resolving several evolutionary theories on sexual selection that have been discussed on this species.
And is modified into
The section is now reduced to three new sentences.

18. Page 18, 36-37: "peacock Though" missing period here.

Reply: The period has been included between the sentences.

19. Page 18, 44: suggest replacing "just" with "valuable" here.

Reply: The alternative work have been replaced in the manuscript

20. Page 22, 14-15: I don't think indicating the flight status of birds is helpful in Fig. 4.

Reply: The figure is modified, the flightless and low flying have been removed from the figure and the figure legend.

Additional notes from letter
Your manuscript "De novo genome assembly of the Indian Blue Peacock (Pavo cristatus), from Oxford Nanopore and Illumina sequencing" (GIGA-D-18-00280R1) has been re-reviewed by our reviewers. Although it is of interest, we are unable to consider it for publication in its current form as significant additional revisions are required. The reviewers have raised a number of points which we believe would improve the manuscript and may allow a revised version to be published in GigaScience so we are giving you one final chance to address these otherwise we cannot keep considering this paper. It is a shame you ignored some of the previously raised significant revisions that need to be made and have been brought up before, specifically the irrelevant discussion of Darwin and sexual selection. In the final version these and the many other speculative discussions need to be removed to just focus on the data

and its validation (including the comparisons of the builds of the many bird genomes currently available).

Reply : We have completely removed sections on Darwin and sexual selection. Significant additional revisions as suggested have been made and the details of each correction are described above.

Please include a point-by-point within the 'Response to Reviewers' box in the submission system. Please ensure you describe additional experiments that were carried out and include a detailed rebuttal of any criticisms or requested revisions that you disagreed with. Please also ensure that your revised manuscript conforms to the journal style, which can be found in the Instructions for Authors on the journal homepage.

Reply : https://academic.oup.com/gigascience/pages/instructions_to_authors

The due date for submitting the revised version of your article is 20 Jan 2019.

Reviewer #1: The manuscript entitled "De novo assembly of Indian Blue Peacock (Pavo cristatus), from Oxford Nanopore and Illumina sequencing" details the results from sequencing and assembling the peacock genome. The manuscript is very much improved and should be ready for publication with only minor revisions.

I think this manuscript lacks one very important point. How does this hybrid assembly compare to other avian genome assemblies? For example, the turkey genome used two different genome sequencers while the original chicken genome made use of Sanger sequencing. Furthermore, many of the 48 bird genomes (Jarvis et al.; Zhang et al, 2014) only used Illumina sequencing at different sequencing depths. I think a comparison between these builds (N50, etc.) should be included in this manuscript. This will aid future researchers who are trying to decide the best sequencing strategy for their favorite bird/organism.

Reply :

The abstract and introduction contain several awkward sentences that impede the reader's understanding. For example, the second to last sentence (lines19-22) of the Abstract Background needs to be rewritten.

Reply : We have changed the second last sentence in the Abstract Background section.

Reviewer #2: The manuscript is much improved over prior versions, but still needs significant revisions.

1) The abstract includes too much detail about the general biology of the peacock and can be shortened for clarity and to focus on the results of the manuscript.

Reply : We have modified our abstract for clarity and have aligned with the other accepted articles in giga science. The biology is completely removed and we have focusedon the key results and the importance of Nanopore long reads.

2) Citations are not numbered in the text and in some cases do not cite the tool or resource correctly (see my note about timetree.org)

Reply : The citations are numbered in the text, the timetree.org reference is now correctly cited.

3) A supplementary figure (Fig. S4) is missing from the text and the table referenced at the same point of the many script doesn't contain relevant data.

Reply : This has been corrected, see above point 13 for details.

4) The Conclusions section includes a largely irrelevant section about sexual selection that needs to be removed.

Reply : Sections related to sexual selection has been completely removed from the manuscript.

5) The Conclusions includes a first reference of a figure that doesn't seem to be referenced in the Methods or Results sections.

Reply : This figures is now referenced in results section “Comparison with other species and databases” in the last paragraph as Fig. 7.

Close
