# Supplementary material for: De novo assembly of the Indian blue peacock (Pavo cristatus) genome using Oxford Nanopore technology and Illumina sequencing
Source: Gigascience. 2019 May 11;8(5):giz038. doi: 10.1093/gigascience/giz038 (PMC6511069; doi:10.1093/gigascience/giz038)
Supplement: Response_to_Reviewer_Comments_Revision_2.pdf [file giz038_response_to_reviewer_comments_revision_2.pdf]

Author's Response To Reviewer Comments

Close

Date: 6 Feb. 2019

Dear Dr. Scott,
Thank you for suggesting improvements in the article. We have addressed all the reviewer's comments. Below are point-by-point response for the corrections raised by the reviewers for the manuscript titled "De-novo genome assembly of the Indian Blue Peacock (Pavo cristatus), from Oxford Nanopore and Illumina sequencing"

Page 2
1. Line 6-9: Remove the two sentences: "The findings from avian genomics …" to "next generation sequencing technologies"
Reply: The two sentences from "The findings from avian genomics …" to "next generation sequencing technologies" are removed.

2. Lines 13: Recommend remove the sentence: "For the first time in avian genomics", since it is redundant with the sentence immediate preceding it.
Reply: The sentence starting from "For the first time in avian genomics" is removed.

3. Line 19: Recommend change "75,315,566" bp to "75.3 Mb"
Reply: The text is changed from "75,315,566" bp to "75.3 Mb"

Page 3
4. Line 6: Replace "reliability" with "completeness"
Reply: The word is replaced from "reliability" with "completeness"

5. Line 8-10: This sentence should be re-written to comment on the significance of this result. Is this expected or unexpected?
Reply: The sentence is modified as "Further in concordance with other phylogenetic studies, the avian phylogeny on the conserved genes showed P. cristatus being closest with Gallus gallus followed by Meleagris gallopavo and Anas platyrhynchos"

Page 4
6. Line 5: Need citation(s) for peacock references in ancient Indian literatures. Probably secondary scholarly works and not primary (ancient Indian) literature references.
Reply: Reference Kadgoankar, 1993 have been added here.

7. Line 14: In the version that I reviewing there is an empty line between the paragraph that ends, "within the orthologous regions [8]", and the paragraph that begins, "Despite the wealth of information".
Reply: The sentence got removed unintentionally in our previous version, we have included the missing sentence in this version.

8. Line 20: Replace "construction" with "sequencing" or "assembly"
Reply: The word "construction" is replaced with "assembly"

Page 5
9. Line 10: Change to "A ReliaPrep™".
Reply: changed to "A ReliaPrep™"

Page 6
10. Line 21: Remove “accurately”
Reply: The word “accurately” is removed

Page 7
11. Line 1-5: This paragraph needs to be rewritten in the past tense to match the rest of the section. “had” instead of “has”, “was” instead of “is”.
Reply: In the paragraph lines 1-5 the sentences are modified in past tense.

Page 8
12. Line 13: “The library mix”
Reply: The sentence modified as “The library mix”

13. Line 14: “The eluted library”
Reply: The sentence modified as “The eluted library”

14. Line 15: “The whole genome library was prepared” or “The whole genome libraries were prepared”
Reply: The sentences is modified as “The whole genome libraries were prepared”

15. Line 22: “bcl2fastq (Illumina)”
Reply: The text is modified to bcl2fastq (Illumina)

16. Line 24: The citation, “(Andrews, S., 2010)” is in the wrong style.
Reply: “(Andrews, S., 2010)” is changed to citation [10] and in reference the following citation is added “Andrews, S., 2010. FastQC: a quality control tool for high throughput sequence data.”

Page 9
17. The phrase “Oxford Nanopore” needs to be replaced with “ONT” or whichever abbreviation the authors choose to use.
Reply: the text “Oxford Nanopore” is changed to “ONT”

Page 10
18. Line 3: “hard masked with the G. gallus repeat library using Repeatmasker (www.repeatmasker.org/).” Proper citation for Repeatmasker is found here: http://repeatmasker.org/faq.html#faq3.
Reply: The reference “Smit, AFA, Hubley, R and Green, P. RepeatMasker Open-4.0. 2013-2015 http://www.repeatmasker.org” is modified

19. Line 6: Replace “obtained” with “identified”
Reply: The word “obtained” is replaced with “identified”

20. Line 10: Proper citations for Augustus found here: http://augustus.gobics.de/references
Reply: the reference is replaced with “[]Stanke, M., Diekhans, M., Baertsch, R. and Haussler, D., 2008. Using native and syntenically mapped cDNA alignments to improve de novo gene finding. Bioinformatics, 24(5), pp.637-644. (http://augustus.gobics.de/)”

21. Line 18: Proper citation for JGI portal https://genome.jgi.doe.gov/pages/citeUs.jsf
Reply: the reference is replaced with “Nordberg, H., Cantor, M., Dusheyko, S., Hua, S., Poliakov, A., Shabalov, I., Smirnova, T., Grigoriev, I.V. and Dubchak, I., 2013. The genome portal of the Department of Energy Joint Genome Institute: 2014 updates. Nucleic acids research, 42(D1), pp.D26-D31.”

22. Line 24: The URLs for Pfam-A database and Pfam scan tools are out of date. Proper citation for Pfam is at the bottom of this page: http://pfam.xfam.org/.
Reply: the reference is replaced with "El-Gebali, S., Mistry, J., Bateman, A., Eddy, S.R., Luciani, A., Potter, S.C., Qureshi, M., Richardson, L.J., Salazar, G.A., Smart, A. and Sonnhammer, E.L.L., 2018. The Pfam protein families database in 2019. Nucleic Acids Research, 47(D1), pp.D427-D432."

Page 11
23. Line 6: There are two papers at the top of this web page (http://avian.genomics.cn/en/jsp/database.shtml) that should be cited as sources for this data.
Reply: Replaced as "avian phylogenomics project []"
And the following citation added
"Zhang, G., Li, B., Li, C., Gilbert, M.T.P., Jarvis, E.D. and Wang, J., 2014. Comparative genomic data of the Avian Phylogenomics Project. GigaScience, 3(1), p.26.
Jarvis, E.D., Mirarab, S., Aberer, A.J., Li, B., Houde, P., Li, C., Ho, S.Y., Faircloth, B.C., Nabholz, B., Howard, J.T. and Suh, A., 2015. Phylogenomic analyses data of the avian phylogenomics project. GigaScience, 4(1), p.4.
"

24. Lines 17,18,20,21 There are papers that should be cited for clustal, Gblock, Phylip and IQ-tree. I don't see them cited here. The papers are listed on the tools' websites.
Reply: Following citation were added,
"Larkin, M.A., Blackshields, G., Brown, N.P., Chenna, R., McGettigan, P.A., McWilliam, H., Valentin, F., Wallace, I.M., Wilm, A., Lopez, R. and Thompson, J.D., 2007. Clustal W and Clustal X version 2.0. bioinformatics, 23(21), pp.2947-2948.
Talavera, G. and Castresana, J., 2007. Improvement of phylogenies after removing divergent and ambiguously aligned blocks from protein sequence alignments. Systematic biology, 56(4), pp.564-577.
Felsenstein, J. 1989. PHYLIP - Phylogeny Inference Package (Version 3.2). Cladistics 5: 164-166.
L.-T. Nguyen, H.A. Schmidt, A. von Haeseler, B.Q. Minh, 2015. IQ-TREE: A fast and effective stochastic algorithm for estimating maximum likelihood phylogenies. Mol. Biol. Evol., 32:268-274."

Page 12
25. Line 8: Papers to cite when using LAST: http://last.cbrc.jp/doc/last-papers.html
Reply: Following citation was added, "Frith, M.C. and Kawaguchi, R., 2015. Split-alignment of genomes finds orthologies more accurately. Genome biology, 16(1), p.106." is added

26. Line 12: Paper to cite when using Circos: If you are using Circos, please cite us: Krzywinski, M. et al. Circos: an Information Aesthetic for Comparative Genomics. Genome Res (2009) 19:1639-1645 | download citation
Reply: Following citation was added "Krzywinski, M.I., Schein, J.E., Birol, I., Connors, J., Gascoyne, R., Horsman, D., Jones, S.J. and Marra, M.A., 2009. Circos: an information aesthetic for comparative genomics. Genome research, 19(9):1639-45."

Page 13
27. Line 4: Remove "(mega base)".
Reply: The text "(mega base)" is removed.
28. Line 9: Change "1.02 GB (giga base)" to "1.02 Gb", remove the "(giga base)".
Reply: The text "1.02 GB (giga base)" is changed to "1.02 Gb"

29. Line 11: Change ">=1 Mbp" to ">= 1 Mb".
Reply: The text ">=1 Mbp" is changed to ">= 1 Mb".

30. Line 16: In accordance with my prior comment, change "75,315,566 bp" to "75 Mb".
Reply: The text is changed from "75,315,566 bp" to "75 Mb"

31. Line 17: Change 56,511,635 bp to "56 Mb".

Reply: The text is changed from “56,511,635 bp” to “56 Mb”.

Page 14
32. Line 14: Change to “The detailed annotations”.
Reply: The text is changed to “The detailed annotations”

33. Line 23: “humans” not “Humans”
Reply: The word is changed to “human”

Page 15
Page 16
34. Line 3: “Zn” to “zinc”
Reply: The text is changed from “Zn” to “zinc”

35. Line 16: “de-novo”, is italicized and not hyphenated elsewhere in the manuscript, except for Line 19 of Page 4. Needs to be consistent. Probably should use “de novo”.
Reply: We have used “de-novo” instead of “de novo” in the manuscript.

36. Line 16-26: This whole paragraph needs some citations, especially for the claim about different technologies improving genome assemblies. Even though you have data that supports this claim and demonstrate ONT’s use in bird genomes for the first time, this idea has been discussed before and has been the basis of at least one genome assembler in the past (eg AllPaths LG) and the topic of several reviews (see Metzker, Nature Reviews Genetics, 2010).
Reply: Citations relevant to the sentences have been included in this paragraph.

Page 17
37. Line 15: “95% homology” implies that 95% of the nucleotides match between sequences, which I don’t is demonstrated in this figure. If the claim is that 95% of the annotated (or predicted; it’s not clear in the text which set of peacock genes is meant) had a match then that should be made clearer. Looking in the abstract, I see the sentence, “The reliability of our genome assembly was verified with the fact that 95% of proteins predicted by homology were matched to those submitted in public repository.” That claim matches more closely the message communicated by Figure 7, so I would re-write this sentence to match the abstract.
Reply: The sentence is now modified as “The confidence on the predicted peacock proteins got strengthened when about 95% of them showed significant homology to various genomic features from different databases (Fig. 7).”

Page 18
38. Line 8: “Figures, Gene ontology and annotations”. This sentence fragment needs to be re-written.
Reply: The section is rewritten as “Additional figures included are the Peacock, Chicken and Turkey proteins showing similarity to Pfam domains, top ten Gene ontology annotations in Biological process; Cellular component and Molecular function from the Peacock proteins, and Peacock homologous proteins in humans.”

Page 21
39. Lines 16-20: This figure caption needs to be shortened, since it is partially a re-write of this section from methods. Could be re-written as “Circular image of the assembled peacock genome aligned against the G. gallus genome. The right side of the image represents the reference chicken genome and left side of the image represents the peacock genome.”
Reply: The text has been shortened as suggested.

Page 22
40. Line 13: Reference 5 here, Kadgoankar, 1993, is not used in the text and provides the information that I said was missing on Page 4, Line 5.

Reply: The missing citation has been added.

41. In the reference list in general, “p” or “pp” is missing from several references (reference 12, 13, 17, maybe others) and should be made consistent throughout.
Reply: We have used reference manager software to make all references consistent.

Close
