# Supplementary material for: De novo assembly of the Indian blue peacock (Pavo cristatus) genome using Oxford Nanopore technology and Illumina sequencing
Source: Gigascience. 2019 May 11;8(5):giz038. doi: 10.1093/gigascience/giz038 (PMC6511069; doi:10.1093/gigascience/giz038)
Supplement: Response_to_Reviewer_Comments_Revision_3.pdf [file giz038_response_to_reviewer_comments_revision_3.pdf]

Author's Response To Reviewer Comments

Close

Dear Dr. Scott,
Kindly find our response to the minor corrections in the manuscript.
All the comments have been replied and corrected in the manuscript, figure 2 and supplementary table and figure description.

Thank you for all the help and review provided.
Regards,

Close
