# Supplementary material for: De novo assembly of the Indian blue peacock (Pavo cristatus) genome using Oxford Nanopore technology and Illumina sequencing
Source: Gigascience. 2019 May 11;8(5):giz038. doi: 10.1093/gigascience/giz038 (PMC6511069; doi:10.1093/gigascience/giz038)
Supplement: Reviewer_1_Report_Original_Submission -- Matthew Greenwold — 8/24/2018 Reviewed [file giz038_reviewer_1_report_original_submission.pdf]

Reviewer Report

Title: De novo assembly of the Indian Blue Peacock (Pavo cristatus) genome using Oxford Nanopore Technology and Illumina sequencing

Version: Original Submission **Date:** 8/24/2018

Reviewer name: Matthew Greenwold

Reviewer Comments to Author:

The manuscript entitled "De novo assembly of Indian Blue Peacock (Pavo cristatus), from Oxford Nanopore and Illumina sequencing reads" details the results from sequencing and assembling the peacock genome. This manuscript, as a data note, is appropriate. However, there are several ways it can be improved.
1) The focus of the manuscript should be on how different sequencing platforms improved the assembly of the peacock genome. However, the benefits (or downside) to using Nanopore technology are not at all addressed. This should be the main point of the manuscript. Perhaps, they can compare an assembly using only Illumina data to that with which they present in the manuscript using both Nanopore and Illumina sequencing data.
2) The authors detail the genome assembly but only supply the SRA data for the public. I know it is not standard for many genome studies, but I think the actual assembled genome should be made public.
3) Generally, the manuscript would benefit from an English speaker going through it thoroughly and correcting the grammar and awkward sentences.
4) The citations are not numbered in the manuscript.
5) I don't know why they reconstructed the mitochondrial phylogeny. It seems out of focus for a data note type manuscript.
6) The conclusion sections of the Abstract and main manuscript are inappropriate and generally should be condensed.
The abstract's "Conclusions" section needs to be completely rewritten. I don't think they addressed any of it in the manuscript except vaguely in the main manuscript conclusions (see below).
The main manuscript Conclusions should be condensed. The first and last paragraphs are the only paragraphs that could be retained. The other paragraphs are more of a discussion nature. Furthermore, they do not discuss the benefits or downside of Nanopore sequencing combined with Illumina sequencing for genome assembly.

Level of Interest

Please indicate how interesting you found the manuscript: Choose an item.

Quality of Written English

Please indicate the quality of language in the manuscript: Choose an item.

Declaration of Competing Interests

Please complete a declaration of competing interests, considering the following questions:

- Have you in the past five years received reimbursements, fees, funding, or salary from an organisation that may in any way gain or lose financially from the publication of this manuscript, either now or in the future?
- Do you hold any stocks or shares in an organisation that may in any way gain or lose financially from the publication of this manuscript, either now or in the future?
- Do you hold or are you currently applying for any patents relating to the content of the manuscript?
- Have you received reimbursements, fees, funding, or salary from an organization that holds or has applied for patents relating to the content of the manuscript?
- Do you have any other financial competing interests?
- Do you have any non-financial competing interests in relation to this paper?

If you can answer no to all of the above, write 'I declare that I have no competing interests' below. If your reply is yes to any, please give details below.

I declare that I have no competing interests.

I agree to the open peer review policy of the journal. I understand that my name will be included on my report to the authors and, if the manuscript is accepted for publication, my named report including any attachments I upload will be posted on the website along with the authors' responses. I agree for my report to be made available under an Open Access Creative Commons CC-BY license (http://creativecommons.org/licenses/by/4.0/). I understand that any comments which I do not wish to be included in my named report can be included as confidential comments to the editors, which will not be published.

I agree to the open peer review policy of the journal

To further support our reviewers, we have joined with Publons, where you can gain additional credit to further highlight your hard work (see: https://publons.com/journal/530/gigascience). On publication of this paper, your review will be automatically added to Publons, you can then choose whether or not to claim your Publons credit. I understand this statement.

Yes Choose an item.
