# Supplementary material for: De novo assembly of the Indian blue peacock (Pavo cristatus) genome using Oxford Nanopore technology and Illumina sequencing
Source: Gigascience. 2019 May 11;8(5):giz038. doi: 10.1093/gigascience/giz038 (PMC6511069; doi:10.1093/gigascience/giz038)
Supplement: Reviewer_1_Report_Revision_1 -- Matthew Greenwold — 10/18/2018 Reviewed [file giz038_reviewer_1_report_revision_1.pdf]

Reviewer Report

Title: De novo assembly of the Indian Blue Peacock (Pavo cristatus) genome using Oxford Nanopore Technology and Illumina sequencing

Version: Revision 1 **Date:** 10/18/2018

Reviewer name: Matthew Greenwold

Reviewer Comments to Author:

The manuscript entitled "De novo assembly of Indian Blue Peacock (Pavo cristatus), from Oxford Nanopore and Illumina sequencing" details the results from sequencing and assembling the peacock genome. The manuscript is very much improved and should be ready for publication with only minor revisions.
I think this manuscript lacks one very important point. How does this hybrid assembly compare to other avian genome assemblies? For example, the turkey genome used two different genome sequencers while the original chicken genome made use of Sanger sequencing. Furthermore, many of the 48 bird genomes (Jarvis et al.; Zhang et al, 2014) only used Illumina sequencing at different sequencing depths. I think a comparison between these builds (N50, etc.) should be included in this manuscript. This will aid future researchers who are trying to decide the best sequencing strategy for their favorite bird/organism.
The abstract and introduction contain several awkward sentences that impede the reader's understanding. For example, the second to last sentence (lines19-22) of the Abstract Background needs to be rewritten.

Level of Interest

Please indicate how interesting you found the manuscript: Choose an item.

Quality of Written English

Please indicate the quality of language in the manuscript: Choose an item.

Declaration of Competing Interests

Please complete a declaration of competing interests, considering the following questions:

- Have you in the past five years received reimbursements, fees, funding, or salary from an organisation that may in any way gain or lose financially from the publication of this manuscript, either now or in the future?
- Do you hold any stocks or shares in an organisation that may in any way gain or lose financially from the publication of this manuscript, either now or in the future?

- Do you hold or are you currently applying for any patents relating to the content of the manuscript?
- Have you received reimbursements, fees, funding, or salary from an organization that holds or has applied for patents relating to the content of the manuscript?
- Do you have any other financial competing interests?
- Do you have any non-financial competing interests in relation to this paper?

If you can answer no to all of the above, write 'I declare that I have no competing interests' below. If your reply is yes to any, please give details below.

I declare that I have no competing interests.

I agree to the open peer review policy of the journal. I understand that my name will be included on my report to the authors and, if the manuscript is accepted for publication, my named report including any attachments I upload will be posted on the website along with the authors' responses. I agree for my report to be made available under an Open Access Creative Commons CC-BY license (http://creativecommons.org/licenses/by/4.0/). I understand that any comments which I do not wish to be included in my named report can be included as confidential comments to the editors, which will not be published.

I agree to the open peer review policy of the journal

To further support our reviewers, we have joined with Publons, where you can gain additional credit to further highlight your hard work (see: https://publons.com/journal/530/gigascience). On publication of this paper, your review will be automatically added to Publons, you can then choose whether or not to claim your Publons credit. I understand this statement.

Yes Choose an item.
