# Supplementary material for: De novo assembly of the Indian blue peacock (Pavo cristatus) genome using Oxford Nanopore technology and Illumina sequencing
Source: Gigascience. 2019 May 11;8(5):giz038. doi: 10.1093/gigascience/giz038 (PMC6511069; doi:10.1093/gigascience/giz038)
Supplement: Reviewer_2_Report_Original_Submission -- Daniel Ence — 8/27/2018 Reviewed [file giz038_reviewer_2_report_original_submission.pdf]

Reviewer Report

Title: De novo assembly of the Indian Blue Peacock (Pavo cristatus) genome using Oxford Nanopore Technology and Illumina sequencing

Version: Original Submission **Date:** 8/27/2018

Reviewer name: Daniel Ence

Reviewer Comments to Author:

The pdf file that I received included two copies of the manuscript text. The second copy begins on page 30 of the pdf file and appears to be identical to the first. I restricted my comments and review to the first copy of the manuscript.

The manuscript is much improved in most aspects. The introduction and conclusion are much improved although the discussion of sexual selection still needs to be reduced. The analysis of Gene Ontology (GO) terms and gene homology across birds are incorrect though. Gene ontology terms are divided into three domains, Biological Process, Cell Component, and Molecular Function. These domains are separate from each other in GO terms but the manuscript (in the results and discussion sections and the supplementary Fig3a-c) treat them as if one gene can only be in one of those three domains. Most analyses just focus on one of those three domains, usually Biological Process or Molecular function.

The claims of overrepresented gene ontology terms aren't tied to any comparisons that I can see in the manuscript, so it isn't clear how any such claim can be made. Claims of overrepresentation should also be backed up by statistical tests for significance. Either the claims of overrepresentation should be removed or analysis should be presented to backup those claims. There are tools that work with GO term annotation to identify overrepresented terms, account for the hierarchy, and test for statistical significance. Two such tools are ClueGO and BinGO which are plugins to the Cytoscape tool.

The mere presence of FGF and KIT proteins in the peacock genome isn't surprising since they are probably present in a large number of other animals' genomes too. Some analysis (even a count of homologs, or some sort of comparative alignment analysis) should be presented.

The results in the "Avian Protein Family" section also need to be re-examined, specifically the finding that ~15,000 out of the ~23,000 peacock genes have no ortholog in other bird genomes. Either a majority of peacock genes are misannotations of some kind, or the CD-HIT clustering was too stringent to identify their homologs.

Level of Interest

Please indicate how interesting you found the manuscript: Choose an item.

Quality of Written English

Please indicate the quality of language in the manuscript: Choose an item.

Declaration of Competing Interests

Please complete a declaration of competing interests, considering the following questions:

- Have you in the past five years received reimbursements, fees, funding, or salary from an organisation that may in any way gain or lose financially from the publication of this manuscript, either now or in the future?
- Do you hold any stocks or shares in an organisation that may in any way gain or lose financially from the publication of this manuscript, either now or in the future?
- Do you hold or are you currently applying for any patents relating to the content of the manuscript?
- Have you received reimbursements, fees, funding, or salary from an organization that holds or has applied for patents relating to the content of the manuscript?
- Do you have any other financial competing interests?
- Do you have any non-financial competing interests in relation to this paper?

If you can answer no to all of the above, write 'I declare that I have no competing interests' below. If your reply is yes to any, please give details below.

I declare that I have no competing interests

I agree to the open peer review policy of the journal. I understand that my name will be included on my report to the authors and, if the manuscript is accepted for publication, my named report including any attachments I upload will be posted on the website along with the authors' responses. I agree for my report to be made available under an Open Access Creative Commons CC-BY license (http://creativecommons.org/licenses/by/4.0/). I understand that any comments which I do not wish to be included in my named report can be included as confidential comments to the editors, which will not be published.

I agree to the open peer review policy of the journal

To further support our reviewers, we have joined with Publons, where you can gain additional credit to further highlight your hard work (see: https://publons.com/journal/530/gigascience). On publication of this paper, your review will be automatically added to Publons, you can then choose whether or not to claim your Publons credit. I understand this statement.

Yes Choose an item.
